# Safety and immunogenicity of DNA omicron booster Alveavax-v1.2 in Ad26.COV2.S-vaccinated adults

**DOI:** 10.1016/j.isci.2025.113970

**Published:** 2025-11-10

**Authors:** Maximilian Schons, Tobias Odendahl, James A. Smith, Kyle Fish, Anemone Franz, Miti Saksena, Sonia Sutherland, Vaughan Reed, Phumzile Mhlongo, Madeli Kruger, Zinhle Zwane, Kgoete Dimakatso, Carol Crowther, Penny Moore, Veronique De Jager, Ethan Alley, Grigory Khimulya

**Affiliations:** 1 Alvea LLC, Cambridge, MA, USA; 2 Micron Research, Ely, Cambridgeshire, UK; 3 Ubuntu Clinical Research, Lenasia, Johannesburg, South Africa; 4 Setshaba Research Centre, Soshanguve, Pretoria, South Africa; 5 AIRU, Sandrigham, Johannesburg, South Africa; 6 University of the Witwatersrand, Sandringham, Johannesburg, South Africa; 7 TASK Brooklyn, Stanberry Street, Brooklyn, Cape Town, South Africa

**Keywords:** Realth sciences, Medicine;Medical specialty, Immunology

## Abstract

A phase I study assessed the safety and immunogenicity of the naked DNA Omicron BA.2 booster vaccine, Alveavax-v1.2, in 130 South African adults previously vaccinated with Janssen Ad26.COV2.S. Participants received varying doses intradermally or subcutaneously, or a Janssen booster as a control. The vaccine was well tolerated, with mild to moderate adverse events across all groups and no serious events linked to vaccination. However, the antibody response was modest, with no significant increases in Omicron BA.2 titers observed in any group. The vaccine remained shelf stable for over six months at room temperature.

## Highlights

• DNA booster is safe and well tolerated at all dose levels
• No significant increase in Omicron BA.2 antibody titers
• High baseline immunity likely due to prior infection
• Vaccine stable at room temperature for over 6 months

## Graphical abstract

**Figure:**
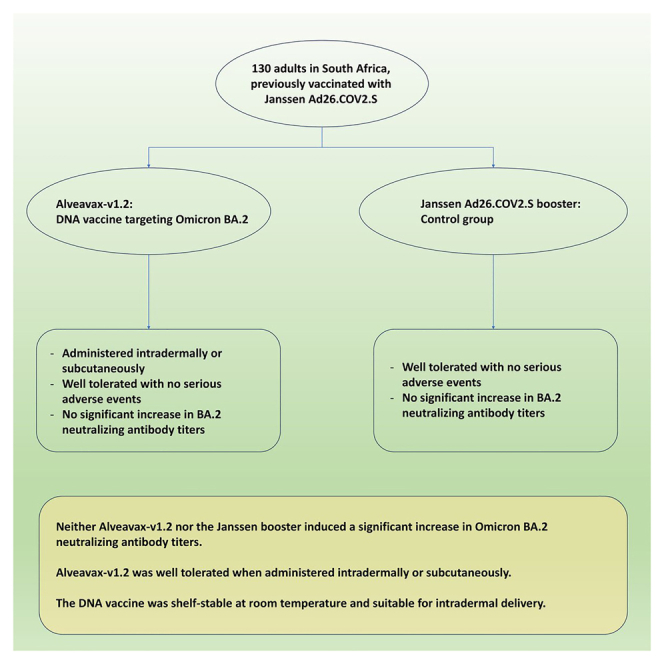

## Teaser

Health sciences; Medicine;Medical specialty; Immunology

## Introduction

Naked DNA vaccine platforms are of particular interest due to their shelf stability and low manufacturing complexity. Like mRNA vaccines, DNA vaccines can be designed quickly and therefore have great potential to address priority pathogens during public health emergencies.^1^ One naked DNA SARS-CoV-2 vaccine (ZyCoV-D) has received emergency use authorization (EUA) in India,^2^ and several other DNA vaccine candidates were in development during the COVID-19 pandemic.^3^

The SARS-CoV-2 Omicron variant (B.1.1.529 lineage) was reported by the WHO in November 2021 as a novel variant of concern with a number of immune evasive mutations.^4^ Besides high cost and limited dose availability, the worldwide distribution of mRNA vaccine candidates for booster shots to resource constrained settings was hampered by the requirement for cold chain storage and shipment.^5^ At the point of design of Alveavax-v1.2, 15 months after the EUA of the first COVID-19 vaccine, more than five billion people worldwide (>60%) had received at least one dose of a COVID-19 vaccine – but only 13·7% of people in low-income countries.^6^

To address vaccine delivery challenges and ensure equitable and rapid access to COVID-19 booster vaccines, Alvea LLC developed a plasmid DNA booster vaccine, Alveavax-v1.2. The vaccine comprises double-stranded plasmid DNA carrying the gene for the SARS-CoV-2 spike protein containing Omicron/BA.2-specific mutations, as well as K986P and V987P (“2P”) proline pre-fusion conformation mutations.^7^ Alveavax-v1.2 uses the well-known pVAX1 backbone.^8^ The plasmid is formulated in preservative-free, sterile phosphate buffer saline (PBS) at a concentration of 5 ± 0·5 mg/mL and administered intradermally (0·1 mL or 0·4 mL). At release, >95% of the plasmids were circular, >90% supercoiled, with stability at room temperature for at least six months based on an acceptance criterion of ≥80% supercoiled. To simplify manufacturing processes and supply chain bottlenecks in resource constrained settings, no additional adjuvants were used, analogous to the ZyCoV-D vaccine. Three unpublished preclinical studies in mice demonstrated neutralizing antibody responses against SARS-CoV-2 BA.2 after the intradermal injection of Alveavax-v1.2 (see Supplements for animal study reports). The aim of the clinical phase I study was to evaluate the safety and tolerability of Alveavax-v1.2 in healthy participants, compared with a control booster vaccine (the Janssen Ad26.COV2.S COVID-19 vaccine), as a booster vaccine against SARS-CoV-2.

## Results

### Trial flow and randomization

**Figure 1:**
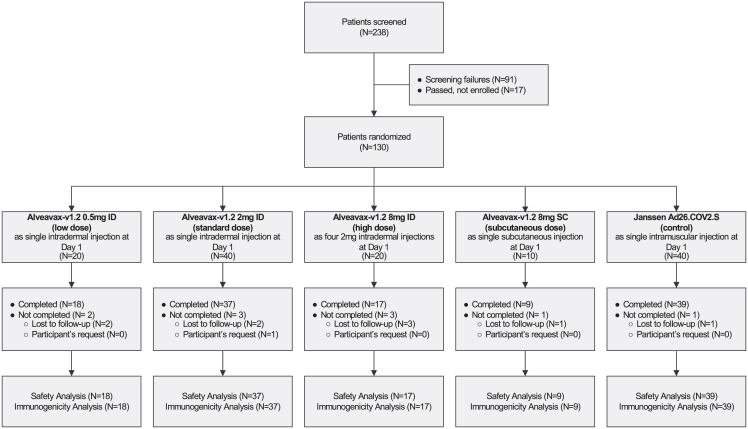
Trial flow diagram Flow diagram for participants in the trial showing screening, group allocation, follow-up, and analysis groups.

**Figure 2:**
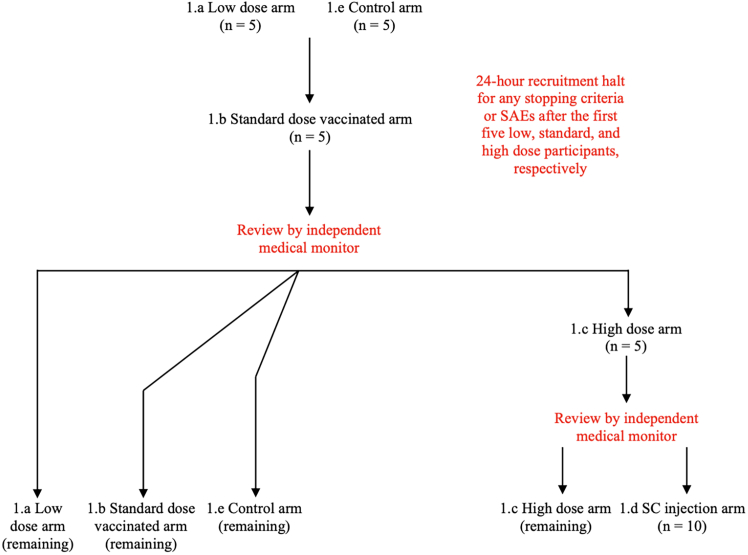
Study design and randomization schema Randomization flow of the study. Red text represents safety events before opening up additional arms. Study groups were as follows. low dose: 0·5 mg intradermal (ID); standard dose: 2 mg ID; high dose: 8 mg as four ID injections of 2 mg each; subcutaneous (SC) injection: 8 mg as a single SC injection; control arm - Janssen Ad26.COV2.S booster as a single intramuscular (IM) injection.

Between 30th June 2022 and 14th September 2022, 238 patients were screened, 91 did not meet eligibility criteria during screening, and 17 met the criteria but were not enrolled in the study. A total of 130 participants were enrolled and randomized, and followed up until 28th February 2023 (see Figures 1 and 2). Nine enrolled participants were lost to follow-up, one withdrew from participation, and three participants did not have a Day 28 immunogenicity result.

### Baseline and demographic characteristics

The mean age (SD) was 32·9 (11·21) years for the Alveavax-v1.2 groups combined, 33·3 (11·82) for the Janssen control arm, mean BMI (SD) was 23·9 (4·23) and 23·6 (3·97), respectively. 39/90 female participants (43·3%) were enrolled across the Alveavax-v1.2 arms and 15/40 (37·5%) were enrolled in the control arm. The study cohort predominantly comprised individuals of Black African descent, with a smaller proportion identifying as Southern African-Colored or Mixed Race. These classifications were based on self-reported data, as per the study’s inclusion criteria. Table 1 details the baseline demographics of the participants across arms.

**Table 1:** Baseline and demographic characteristics

	Alveavax	Alveavax-	Alveavax	Alveavax-	Alveavax-	Janssen
	-v1.2 0·5	v1.2 2 mg	-v1.2 8	v1.2 8 mg SC	v1.2 all	Ad26.COV2.S
	mg ID (low dose) *N* = 20	ID (standard dose) *N* = 40	mg ID (high dose) *N* = 20	(subcutaneo us dose) *N* = 10	participants (combined) *N* = 90	(Control) *N* = 40
**Gender n (%)**						

Male	11 (55·0%)	26 (65·0%)	10 (50·0%)	4 (40·0%)	51 (56·7%)	25 (62·5%)
Female	9 (45·0%)	14 (35·0%)	10 (50·0%)	6 (60·0%)	39 (43·3%)	15 (37·5%)

**Age (y)**						

Mean (SD)	35·5 (13·30)	32·2 (11·00)	31·0 (9·19)	34·2 (11·83)	32·9 (11·21)	33·3 (11·82)
Median	34·5	29·5	31·0	36·5	31·0	29·5
Range	19·0–61·0	18·0–58·0	19·0–57·0	21·0–57·0	18·0–61·0	19·0–60·0

**Race n (%)**						

Black African	16 (80·0%)	39 (97·5%)	18 (90·0%)	8 (80·0%)	81 (90·0%)	37 (92·5%)
Mixed race- Colored	1 (5·0%)	0 (0·0)	0 (0·0)	0 (0·0)	1 (1·1%)	0 (0·0)
Southern African- Colored	3 (15·0%)	1 (2·5%)	2 (10·0%)	2 (20·0%)	8 (8·9%)	3 (7·5%)

**Height (cm)**						

Mean (SD)	165·3 (9·36)	166·3 (7·84)	165·1 (8·21)	165·4 (8·65)	165·7 (8·24)	165·7 (9·31)
Median	167·5	166·5	161·9	164·2	165·9	167·5
Range	145·5–176·2	147·0–182·4	146·4–182·0	155·0–178·4	145·5–182·4	145·0–181·0

**Weight (kg)**						

Mean (SD)	68·2 (11·67)	65·1 (10·69)	65·6 (11·13)	60·8 (11·14)	65·4 (11·06)	64·7 (12·14)
Median	68·1	67·3	62·4	57·2	65·9	61·6
Range	48·9–86·0	43·1–87·0	46·3–85·8	45·3–79·9	43·1–87·0	42·4–90·0

**BMI (kg/m**^**2**^**)**						

Mean (SD)	25·0 (4·19)	23·6 (4·07)	24·1 (4·03)	22·5 (5·30)	23·9 (4·23)	23·6 (3·97)
Median	24·9	23·0	22·6	20·9	23·2	22·6
Range	19·1–32·0	17·7–31·6	19·8–32·9	17·5–31·6	17·5–32·9	18·1–31·5

ID = intradermal, SC = subcutaneous.

### Adverse reactions

**Figure 3:**
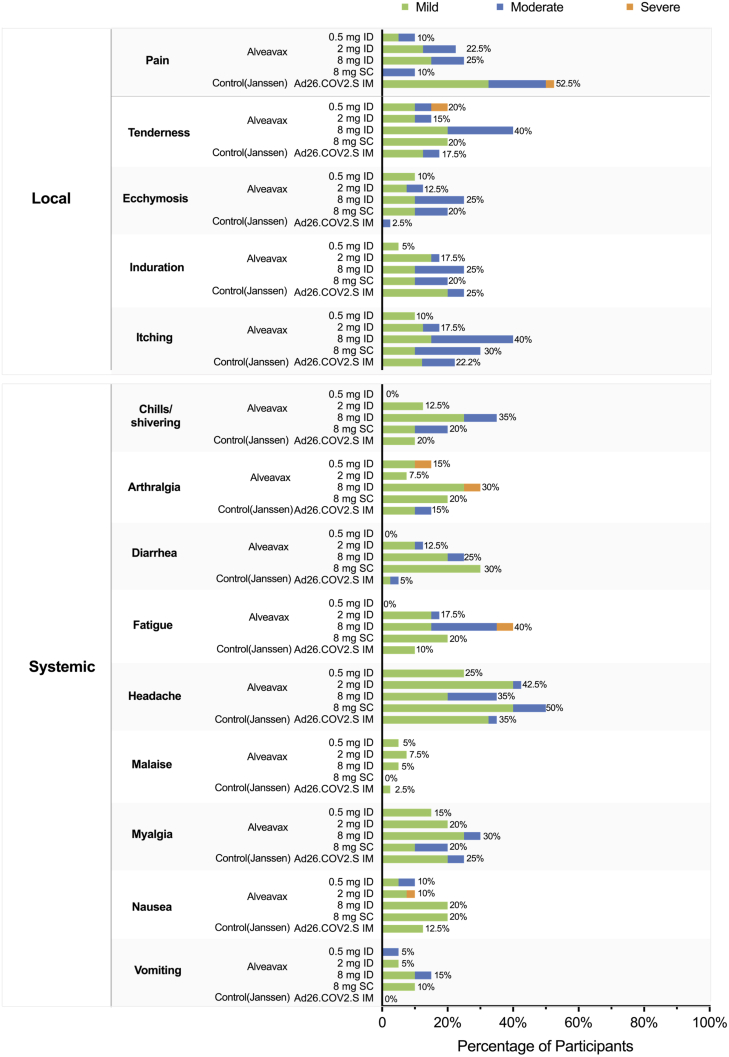
Solicited local and systemic adverse reactions Shown are the percentages of participants in whom solicited local or systemic adverse reactions occurred within seven days of the vaccine dose administered in the trial. (20, 40, 20, and 10 participants in the Alveavax-v1.2 0·5 mg [low], 2 mg [standard], 8 mg [high], and 8 mg subcutaneous [subcutaneous] group; 40 participants in the Janssen Ad26.COV2.S [control] group). Grade 1 (Mild): transient or mild discomfort (<48 h); no medical intervention/therapy required. Grade 2 (Moderate): mild to moderate limitation in activity - some assistance may be needed; no or minimal medical intervention/therapy required Grade 3 (severe): marked limitation in activity, some assistance usually required; medical intervention/therapy required, hospitalization possible or medically significant but not immediately life-threatening; hospitalization or prolongation of hospitalization indicated; disabling; limiting self-care activities of daily living.

The medical exams and history confirmed a healthy participant population without significant prior or current illness. None of the laboratory and vital sign values post vaccination were deemed clinically significant. Solicited AEs reported within the week after vaccination included mostly mild to moderate signs (see Figure 3). Mild to moderate injection pain on the day of injection was present in 12/80 (15%) for ID, 1/10 (10%) for SC, and 17/40 (42·5%) for IM injections, and lasted up to Day 6 in three Alveavax-v1.2 and one control participant. Individual participants experienced severe arthralgia, fatigue, nausea, or local tenderness in the Alveavax-v1.2 groups and severe pain in the Janssen control arm. Symptoms resolved quickly for most participants. Moderate to severe fatigue was experienced by a subset of participants in the Alveavax-v1.2 high dose group at Day 5 and Day 6 (moderate *N* = 2; severe, *N* = 1). Two participants in the high dose arm and two in the subcutaneous arm showed clinically significant abnormal findings at the physical examination at Day 7 (Skin and head, eyes, ears, nose, and throat [HEENT] and HEENT and lymphatic, respectively), which in the further course returned to normal.

Unsolicited AEs up until Day 28 were experienced by 8/20 (40%), 21/40 (52·5%) (16/20 (80%), 7/10 (70%), and 29/40 (72·5%), for low, standard, high, subcutaneous, and control, respectively. Common events, both across Alveavax-v1.2 and control, included aforementioned injection site reactions, fatigue (14/90 [15·6%], 1/40 [2·5%]), and headache (24/90 [26·7%], 7/40 [17·5%]). Three SAEs judged unrelated to vaccination were recorded for a single Alveavax-v1.2 participant in the high dose group (a severe lower respiratory tract infection requiring hospitalization, a fecaloma that resolved, and a pregnancy that resulted in a complication-free vaginal birth with a healthy newborn). There were no AESIs or deaths during the study. A table summarizing all adverse events classified by organ system can be found in the supplemental information (Table S1 in the Supplements).

### Vaccine administration

The administration of the full dose of the vaccine candidate was successful in 136/140 (97·1%) intradermal injections, 9/10 (90%) subcutaneous injections, and 40/40 (100%) intramuscular injections. The median bleb size for intradermal injections was 6·9 mm (SD 2·44) and 10·7 mm in diameter (SD 2·66, see Tables 2 and 3) for 0·1 mL and 0·4 mL, respectively.

**Table 2:** Administration success of the vaccine

	Alveavax-v1.2 0·5 mg ID (low dose) *N* = 20	Alveavax -v1.2 2 mg ID (standard dose) *N* = 40	Alveava x-v1.2 8 mg ID (high dose) *N* = 20	Alveavax-v1.2 8 mg SC (subcutan eous dose) *N* = 10	Alveavax- v1.2 all participant s (combined) *N* = 90	Janssen Ad26.C OV2.S (Control) *N* = 40
**Was the full dose administered**						

n	20	40	80	10	150	40
Yes	20 (100%)	39 (97·5%)	77 (96·3%)	9 (90·0%)	145 (96·7%)	40 (100%)
No	0	1 (2·5%)	3 (3·8%)	1 (10·0%)	5 (3·3%)	0

The denominator for the number of vaccinations and the number administered a full dose is the total number of vaccinations not the number of participants.

**Table 3:** Bleb size following intradermal vaccination

	Alveav ax-v1.2 0·5 mg ID (low dose) *N* = 20	Alveava x-v1.2 2 mg ID (standar d dose) *N* = 40	Alveavax -v1.2 8 mg ID (high dose) Injection 1 *N* = 20	Alveavax -v1.2 8 mg ID (high dose) Injection 2 *N* = 20	Alveavax -v1.2 8 mg ID (high dose) Injection 3 *N* = 20	Alveavax -v1.2 8 mg ID (high dose) Injection 4 *N* = 20
**Size of Bleb (mm) when it Lasted 20 s or more**						

n	19	39	20	20	20	20
Mean (SD)	6·9 (2·44)	10·7 (2·66)	10·7 (2·30)	11·1 (2·10)	10·7 (2·25)	10·6 (1·70)
Median	8·0	10·0	12·0	12·0	11·0	10·0
Min-Max	3·0 to 10·0	7·0 to 16·0	6·0 to 14·0	7·0 to 14·0	7·0 to 14·0	7·0 to 14·0

Note: only two participants had blebs lasting less than 20 s. They are not included in the table.

### Immune response

**Figure 4:**
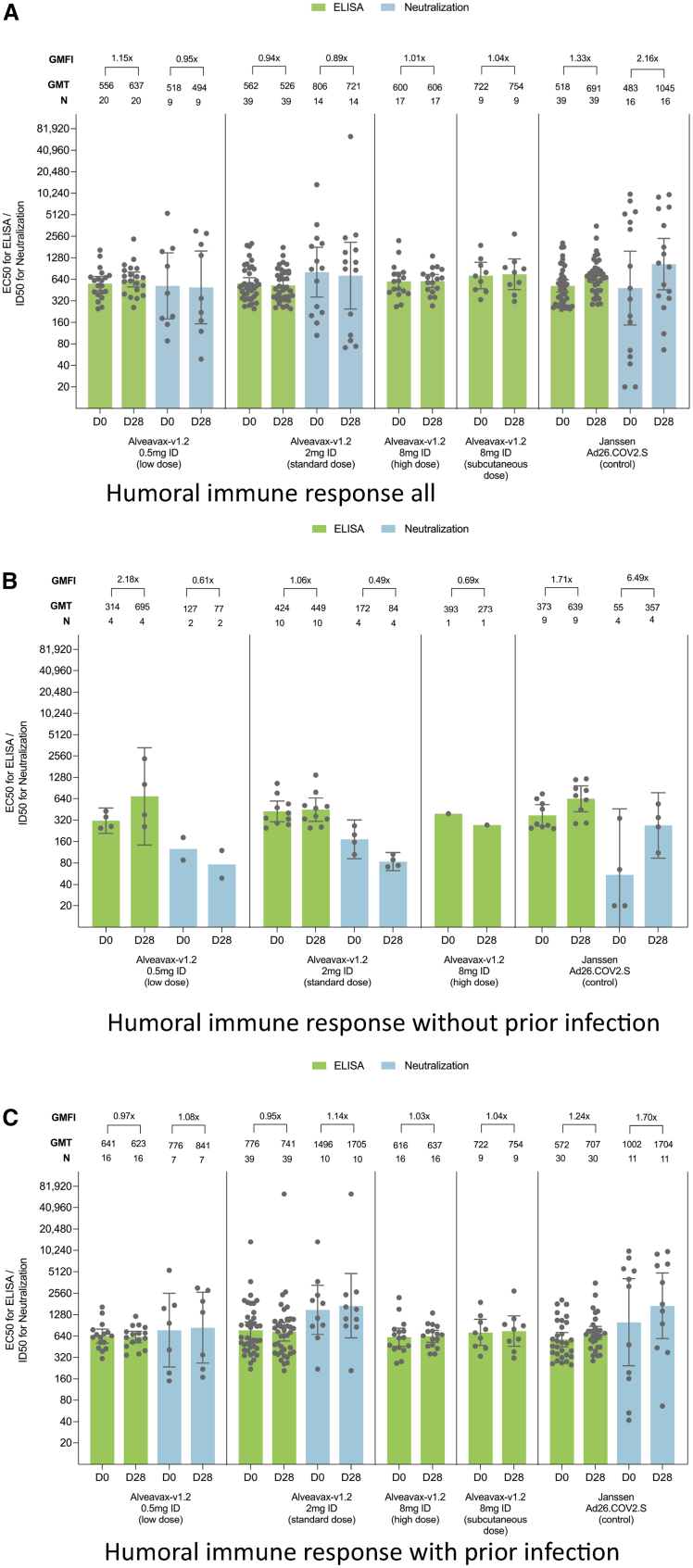
Humoral immune response ELISA and neutralization data on Day 0, Day 28, and Day 84 measured by geometric mean titer (GMT) of anti-spike protein (S) immunoglobulin G antibody for SARS-CoV-2 BA.2/Omicron are shown. (A) All participants pooled. (B) Subset of participants without prior infection based on nucleocapsid antibody titers. (C) Subset of participants with prior infection based on nucleocapsid antibody titers.

GMT for BA.2 spike at baseline assessed by ELISA for low, standard, high, subcutaneous and control was 556 (95% CI, 441 to 701), 562 (95% CI, 467 to 676), 600 (95% CI, 451 to 798), 722 (95% CI, 472 to 1107), 518 (95% CI, 425 to 632) (see Figure 4A). ELISA BA.2 GMT fold increase in the mITT population based on Day 28 ELISA data for low, standard, high, subcutaneous, and control was, 1·15 (95% CI, 0·72 to 1·83), 0·94 (95% CI, 0·66 to 1·33), 1·01 (95% CI, 0·61 to 1·66), 1·04 (95% CI, 0·41 to 2·62), and 1·33 (95% CI, 0·91 to 1·95), respectively. A subset of Day 84 participant samples was tested and confirm the magnitude of geometric mean fold increase (GMFI). ELISA fold rises were validated via neutralizing antibodies for Omicron BA.2 in a subset of 27/130 (20·8%) participants of the low dose, standard dose, and control arm. Figure 4B plots the BA.2 antibodies of the subgroups of participants 25/130 (19%) who were not positive for SARS-CoV-2 nucleocapsid antibodies at baseline, Figure 4C plots those who had a previous SARS-CoV-2 infection prior to the administration of the vaccine. Baseline GMTs for the group of uninfected participants were lower when compared to the infected population and GMFI tended to be higher for the low dose and the Janssen control arm. Only one participant in the standard group experienced a COVID-19 infection during the study; thus, no statistical analysis using the WHO clinical progression scale was performed.

## Discussion

The review of Shafaati et al. identified eleven DNA based vaccine candidates for primary vaccination in clinical testing.^3^ To our knowledge, this phase I safety, tolerability, and immunogenicity study represents the first report of a DNA COVID-19 booster vaccine candidate evaluated in humans. Searches for “DNA COVID-19/SARS-CoV-2 vaccine booster/omicron clinical trial” in PubMed, Medline, Google Scholar, and clinicaltrials.gov yielded three registered DNA based SARS-CoV-2 booster vaccine clinical trials, which have not yet reported their results (NCT05182567, NCT05171946, NCT05904054). DNA vaccines for primary SARS-CoV-2 vaccination were able to elicit both humoral and cellular immune responses in SARS-CoV-2 naive populations when administered IM or ID.^3^ Typical dose levels per vaccination are in the 1–3 mg range and included up to three administrations for primary vaccination and GMTs of 952·7 based on ELISA at Day 84.^8^

This study provided a single boost with a BA.2 optimized naked DNA vaccine to healthy adults with a single primary Janssen Ad26.COV2.S vaccination. Approximately 80% of those randomized had also previously experienced an infection, which is in accordance with South African seroprevalence studies conducted end of 2021.^9^ It was hypothesized that the potency of a single DNA shot could be sufficient in the context of preimmunized participants, most of whom have hybrid immunity from past infections. Preclinical experiments before and after the clinical trial with single- and escalating dose regimens similar to HIV vaccines^10^^,^^11^ showed potent development of neutralizing antibodies against various SARS-CoV-2 strains (see Supplements). The candidate vaccine was randomized against Janssen’s Ad26.COV2.S to provide comparative data about the expected effectiveness at a very early stage of clinical development and to inform future sample size calculations.^12^ Despite active comparisons with approved vaccines in early clinical trials being common for other conditions,^13^^,^^14^ randomization against approved SARS-CoV-2 vaccines has been astonishingly rare for COVID-19 vaccine candidates.^15^

In alignment with the broad safety literature already available for DNA vaccines,^1^ Alveavax-v1.2 was safe and well tolerated among all treatment groups, with frequency and intensity of solicited and unsolicited AEs matching those of the comparator, DNA primary series^8^ and mRNA booster vaccine programs.^16^ Both the Janssen comparator as well as Alveavax-v1.2 were not able to elicit meaningful GMT fold rises against Omicron/BA.2 on top of the already high antibody titers. This is noticeable, as there was a 16x dose difference between the low and high dose Alveavax-v1.2 arms and the control, the Janssen Ad26.COV2 vaccine was a known effective vaccine. It is possible that there were limitations in the design and conduct of the study, whereby the baseline antibody titers may have been too high to show a titer increase after treatment. The analysis of the small subgroup of uninfected participants with lower baseline titers and slightly higher GMFIs is in agreement with this hypothesis. The overall baseline titers of the study’s South African population were comparable to post-boost titers (606 and 896 for age groups 18–55 and >55, respectively) of Pfizer’s BA4/5 trial.^17^ Zhou et al. collected neutralization data across multiple SARS-CoV-2 variants, showing that the geometric mean factor increase is generally substantially greater in individuals without previous infection compared to individuals with previous infection, even at the fourth booster dose.^18^ DNA vaccine neutralizing humoral immune responses have been small even in primary vaccination.^8^ While a lack of humoral response does not imply absence of additional protection - for instance, via cellular response mechanisms^19^ - it still led to a hold on further exploratory testing and the clinical program for this drug candidate. Participants with low titers were informed, and additional vaccination with a licensed vaccine was recommended.

Further study limitations were that the study population had a potential lack of heterogeneity in the study population recruited only from South Africa, and that they were a healthy population that excluded patients with HIV. The results may not be transferable to other vaccination regimens, as only people who had received the Janssen vaccine as a first vaccination and had not received an mRNA-based vaccine as primary vaccination were examined. Alternative ways of administration other than ID with needles were not studied, due to the widespread availability of needles even in resource limited contexts. Reliance on ID injections with needles makes direct immunogenicity comparison to other DNA vaccine trials difficult, where proprietary needle-free injection systems or electroporation were studied to improve the immune response.^20^

The strengths of this study included the multicenter and the active controlled study design. The total and per arm population was relatively large for a phase I study, and a representative region for low- and middle income countries was selected. Alveavax-v1.2 was targeting the dominant Omicron strain at the time.

To our knowledge, for the first time, an ID dosing up to 8 mg and SC administration routes in DNA vaccines were evaluated in humans.

In spite of not attaining the desired immunological endpoints, it is a noteworthy accomplishment that a drug development initiative guided by principles of vaccine equity has showcased arguably the most expeditious transition of a pharmaceutical entity to the clinical phase in comparison to analogous trials. This contrasts with the prevailing public perception that the drug development process is marked by languor, bureaucratic impediments, and inaccessibility for nascent enterprises.^21^ A mere 174 days subsequent to the establishment of Alvea at the advent of the Omicron wave, the human trial of Alveavax-v1.2 started, without compromising on best practices. This reinforces the imperativeness of persistent investments in broadly protective and equitable medical countermeasure platforms. Furthermore, it confirms how operational excellence and financial risk-taking can enable clinical trials to be realized in the shortest possible time.

### Limitations of the study

• Baseline antibody titers in participants may have been too high to detect a meaningful post-vaccination increase. This could have limited the observed immunogenicity of the booster. A small subgroup of uninfected participants with lower baseline titers showed slightly higher geometric mean fold increases (GMFIs), supporting this hypothesis.
• The study population was limited to healthy adults in South Africa, excluding individuals with HIV or other comorbidities. This lack of heterogeneity may restrict the generalizability of the findings to broader or more diverse populations. Additionally, the study only included participants previously vaccinated with the Janssen Ad26.COV2.S vaccine and did not assess responses in individuals primed with mRNA-based vaccines.
• Alternative administration methods beyond intradermal injection with needles were not explored, though such methods may be relevant for broader deployment. The choice was based on the widespread availability of needles, even in resource-limited settings.

## Resource availability

### Lead contact

Further information and requests for resources, data, and reagents should be directed to and will be fulfilled by the lead contact, Tobias Odendahl (email: alvea@infozelt.de).

### Materials availability

The vaccine candidate Alveavax-v1.2 is a proprietary product developed by Alvea Holdings, LLC. It is not currently commercially available but may be made available upon reasonable request and subject to a material transfer agreement.

### Data and code availability

• **Data**: All individual participant data collected during the trial, after de-identification, along with the study protocol, statistical analysis plan, informed consent form, and clinical study report, are publicly available and licensed under **CC BY 4.0**. These materials are accessible via **Mendeley Data**: Schons, Maximilian (2023), *Phase I clinical trial dataset: naked DNA SARS-CoV-2 Omicron BA.2 booster vaccine Alveavax-v1.2 against Janssen Ad26.COV2.S comparator,* NCT05844202, Mendeley Data, V1. https://data.mendeley.com/datasets/pjrs3rrnfc/1, https://doi.org/10.17632/pjrs3rrnfc.1. There are no restrictions on access or reuse of these data and documents, which are available indefinitely for any purpose.
• **Code**: Not applicable.
• **Other items:** The trial is registered at ClinicalTrials.gov: NCT05844202.

## Acknowledgments

The study was funded by the sponsor, Alvea Holdings, LLC. Penny Moore and her lab are supported by the South African Research Chairs Initiative of the Department of Science and Innovation and the National Research Foundation of South Africa, and the SA Medical Research Council program.

## Author contributions

We want to acknowledge the following individuals who have made substantial contributions to the execution of this clinical trial: Sashkia Balla, Angela Cai, Christopher Da Costa, Ryan Duncombe, Kevin Esvelt, Connor Flexman, Sumen Govender, Cate Hall, Dang Khanh Ngan Ho, Alwyna Holtzhausen, Stephanie Koehl, Madeleine Lourens, Phumzile Promise Mhlongo, Zanele Makhado, Thandeka Moyo-Gwete, Portia Mutevedzi, Brent Packer, Georg Petrick, Sarah Robinson, Patricia Rutherfoord, Kenza Samlali, Pascal Scheven, Jannik Stemler, Subrahmanian Tarakkad Krishnaji, Adam Trotman, Roland van Rensburg, Brian Wang, and Kyle Webster.

## Declaration of interests

Maximilian Schons received grants from Open Philanthropy and Lightspeed Grants. James Smith received grants from the European Commission, Open Philanthropy, Lightspeed Grants, NIHR Oxford Biomedical Research Center, and the Long-term future fund. He received consulting fees from OneDaySooner. Ethan Alley is the inventor of several patents related to nucleic acid vaccines.

## STAR★Methods

### Key resources table

**Table:** 

REAGENT or RESOURCE	SOURCE	IDENTIFIER
**Software and algorithms**		

SAS® software (version 9.4 or higher)	SAS Institute Inc., USA	https://www.sas.com
MedDRA (version 25.1)	MedDRA MSSO (Maintenance and Support Services Organization)	https://www.meddra.org

### Experimental model and study participant details

#### Human participants

A total of 238 individuals were screened, and eligible healthy adult volunteers aged 18 to 65 years (inclusive) were enrolled across seven non-hospital clinical research sites in South Africa. Sex was self-reported as “male,” “female,” or “undifferentiated.” Participants were eligible if they had previously received a single dose of the Janssen Ad26.COV2.S COVID-19 vaccine at least 60 days before enrollment. Individuals were excluded if they had received any other SARS-CoV-2 vaccine or planned to receive additional SARS-CoV-2 vaccination within 90 days following study vaccine administration.

#### Ethical approvals

All participants provided written informed consent before any study procedures. The study was approved by the South African Health Products Regulatory Authority and the South African Medical Association Research Ethics Committee. It was registered in the South African National Clinical Trials Registry (Identifier: DOH-27-062022-5157) and on ClinicalTrials.gov (Identifier: NCT05844202).

#### Randomization and study arms

The study used a randomized, open-label design. Randomization was performed centrally using a computer-generated fixed sequence, without blocking, and participants were assigned sequentially. Enrolled participants were randomized into one of five groups.

• **Low dose**: 0.5 mg Alveavax-v1.2 intradermally (ID); 20 participants
• **Standard dose**: 2 mg ID; 40 participants
• **High dose**: 8 mg as four 2 mg ID injections; 20 participants
• **Subcutaneous (SC)**: 8 mg as a single SC injection; 10 participants
• **Control**: Janssen Ad26.COV2.S booster as a single intramuscular (IM) injection; 40 participants

#### Vaccination and follow-up

Vaccines were administered during a single Day 1 enrollment visit. Participants were observed on-site for local or systemic reactions for either 4 h (early safety cohorts) or 30 min (remaining participants) after vaccination. Follow-up included a phone call on Day 3 and in-person visits on Days 7, 14, 28, 84, and 168 for safety monitoring, laboratory assessments, and reporting of pregnancy, COVID-19 infections, or additional vaccinations.

### Method details

#### Study design and procedures

An open-label, active-controlled, randomized safety and dose-finding study was chosen to evaluate Alveavax-v1.2. Participants were recruited from seven non-hospital study sites in South Africa. Approval by the South African Health Products Regulatory Authority and South African Medical Association Research Ethics Committee were obtained (South African National Clinical Trials Registry Identifier: DOH-27-062022-5157. ClinicalTrials.gov Identifier: NCT05844202). The study protocol can be found in the supplemental information.

The main inclusion criteria were healthy volunteers (sex self-reported with the options “male”, “female”, and “undifferentiated”) between the age of 18 and 65 years (inclusive) having received a single primary Janssen Ad26.COV2.S COVID-19 vaccine ≥60 days prior to receiving the study vaccine. Volunteers were excluded when they had received any other form of SARS-CoV-2 vaccination or planned to receive any additional SARS-CoV-2 vaccination within 90 days after the study vaccine administration. The full list of eligibility criteria in the study protocol and the informed consent forms can be found in the supplements. A participant was considered a screen failure if the informed consent form was signed but they were ineligible at the screening visit or withdrew before receiving the study vaccine.

The arms of the study were (see Figure 1).

• Low dose – 0·5 mg intradermal (ID), 20 participants;
• Standard dose – 2 mg ID, 40 participants;
• High dose – 8 mg as four ID injections of 2 mg each, 20 participants;
• Subcutaneous (SC) injection – 8 mg as a single SC injection, 10 participants;
• Control – Janssen Ad26.COV2.S booster as a single intramuscular (IM) injection, 40 participants.

The vaccine candidate was stored in temperature controlled refrigerators at 2°C–8°C. Each Alveavax-v1.2 vial contained sufficient volume for two standard doses (2 mg, 0·4 mL), with 30% excess fill volume. The Janssen Ad26.COV2.S vaccine was stored and administered according to the package leaflet. After successful screening, participants had one nrolment and vaccine administration visit. Local staff were provided with video training of correct intradermal administration and measurement of intradermal bleb size. No additional training for subcutaneous or intramuscular injections was provided. After administration of the study vaccine on Day 1 participants were monitored on-site for local or systemic reactions to the vaccine either 4 h (early safety cohorts) or 0·5 h (remaining study participants). Subsequent visits included a telephone call on Day 3 and in person follow-up visits on days 7, 14, 28, 84 and 168. Each visit included safety and laboratory assessments, in addition to inquiries regarding pregnancy and COVID-19 infections or vaccinations. The full schedule of events can be found in the study protocol in the supplements.

#### Randomisation and masking

Initially, participants were randomized in two sequential safety cohorts and the remaining cohorts were opened for randomization thereafter. An illustration of the randomization flow is presented in Figure 2. The randomization lists and specifications are provided in the supplements.

The first 10 participants were randomized into the low dose arm (1.a) and the control arm (1.e). No more than five participants were vaccinated on the first day, and subsequent recruitment of the study groups or escalation between dose levels was allowed only after an independent medical monitor had reviewed at least 24-h post-dose safety data.

Then, the remaining participants assigned to the low and standard dose (1.b) cohorts, in addition to those assigned to the control arm, were recruited. In parallel, the first five participants of the high dose (1.c) arm were enrolled. Further recruitment and the subcutaneous injection arm (1.d) were started after an independent medical monitor reviewed 24-h safety data of the high dose arm.

A central interactive response technology (IRT) randomization system generated the sequence and allocated participants into the respective arms of the trial. Block sizes ranged between one and five. An open-label design with no blinding for study site staff or participants was selected. Safety and immunogenicity laboratory personnel were blinded.

### Quantification and statistical analysis

#### Outcomes

Primary endpoints for the assessment of safety were solicited local and systemic adverse events (AEs) within seven days of dose administration via patient diary cards, unsolicited AEs within 28 days of vaccination, serious adverse events (SAEs), adverse events of special interest (AESIs), and AEs leading to withdrawal during the 6 months post vaccination follow-up period. Secondary endpoints included humoral immune response on Day 1 and Day 28 measured by geometric mean titer (GMT) and geometric mean fold rise (GMFR) of anti-spike protein (S) immunoglobulin G (IgG) antibody for SARS-CoV-2 BA.2/Omicron, clinical efficacy measured using the WHO clinical progression scale for COVID-19 on Day 7/14/28/84/168, and the success rate of ID injections as measured by the absolute number and fraction of ID injections that generated a clearly demarcated bleb, of ≥1 mm and ≥7 mm in diameter, clearly visible for at least 20 s, for 0·5 mg (0·1 mL) and 2 mg (0·4 mL) Alveavax-v1.2, respectively. All humoral assays were performed by the National Institute for Communicable Diseases (NICD) in Johannesburg, South Africa. Details on the assays are described in the supplements. Additional exploratory endpoints listed in the protocol, including cellular immune responses, were not performed.

#### Statistical analysis

As this was a phase I study, all data were analyzed descriptively without a formal statistical hypothesis and sample sizes were not based on a statistical power calculation. Figure 1 illustrates the participants included in the safety and immunogenicity analyses.

The safety population was the set of all study participants who were administered with a dose of the vaccine candidate. Participants were grouped as treated. All enrolled participants who received a study vaccine and experienced at least one post-baseline immunogenicity readout comprised the modified intent-to-treat (mITT) population. Missing or non-evaluable measurements were not replaced. The mITT and Safety populations were identical. The mITT population was used instead of the per protocol population for the immunogenicity analyses as the differences to the Janssen control were small and the former included more participants.

Categorical variables were summarized as frequencies and percentages. Continuous variables were summarized using descriptive statistics (number of participants with an observation [n], mean, standard deviation [SD], median, and range). Where partial dates (missing day or missing day and month) were recorded on the electronic case report form (CRF) and where these could not be resolved by queries, dates were estimated for the purpose of calculating durations. Statistical analysis was performed using SAS software (version 9.4 or higher; SAS Institute Inc., USA). AEs were coded using MedDRA version 25.1. Coding included the system organ class and preferred term. The statistical analysis plan as well as the code conducted to run the analysis are included in the supplements.

### Additional resources

#### Role of the funding source

There was no funding source for this study other than the sponsor, Alvea Holdings, LLC. The funding source was involved in the study design, collection of data, analysis, interpretation of data, writing of the study report, writing of this paper and the decision to submit the paper for publication.

## Supplemental information

Document S1. Table S1

Data S1. Study report

Data S2. Randomization codes

Methods S1. Study protocol

Methods S2. Statistical analysis plan

Methods S3. Informed consent sample storage

Methods S4. Informed consent HIV test

Methods S5. Informed consent core

Methods S6. Informed consent screening
