# Supplementary material for: Safety and immunogenicity of DNA omicron booster Alveavax-v1.2 in Ad26.COV2.S-vaccinated adults
Source: iScience. 2025 Nov 10;28(12):113970. doi: 10.1016/j.isci.2025.113970 (PMC12704268; doi:10.1016/j.isci.2025.113970)
Supplement: Document S1. Table S1 [file mmc1.pdf]

iScience, Volume *28*

Supplemental information

Safety and immunogenicity of DNA omicron booster Alveavax-v1.2 in Ad26.COV2.S-vaccinated adults

Maximilian Schons, Tobias Odendahl, James A. Smith, Kyle Fish, Anemone Franz, Miti Saksena, Sonia Sutherland, Vaughan Reed, Phumzile Mhlongo, Madeli Kruger, Zinhle Zwane, Kgoete Dimakatso, Carol Crowther, Penny Moore, Veronique De Jager, Ethan Alley, and Grigory Khimulya

Table 4.1: All Adverse Events Classified by System Organ Class and Preferred Term – Safety Population

By System Organ Class and Preferred Term	Low Dose N= 20	Standard N= 40	High Dose N= 20	SC Injection N= 10	Combined N= 90	Control N= 40
**Number (%) of Participants Reporting any TEAE**	**n=8 (40.0%) [30]**	**n=21 (52.5%) [97]**	**n=16 (80.0%) [94]**	**n=7 (70.0%) [33]**	**n=52 (57.8%) [254]**	**n=29 (72.5%) [82]**
**GENERAL DISORDERS AND ADMINISTRATION SITE CONDITIONS**	5 (25.0%) [13]	10 (25.0%) [44]	13 (65.0%) [57]	6 (60.0%) [14]	34 (37.8%) [128]	16 (40.0%) [40]
**Injection site reaction**	4 (20.0%) [10]	8 (20.0%) [31]	12 (60.0%) [48]	5 (50.0%) [9]	29 (32.2%) [98]	16 (40.0%) [35]
**Chills**	0	2 (5.0%) [3]	3 (15.0%) [3]	2 (20.0%) [2]	7 (7.8%) [8]	2 (5.0%) [2]
**Fatigue**	1 (5.0%) [1]	6 (15.0%) [8]	5 (25.0%) [5]	2 (20.0%) [2]	14 (15.6%) [16]	1 (2.5%) [1]
**Influenza like illness**	0	0	0	1 (10.0%) [1]	1 (1.1%) [1]	1 (2.5%) [1]
**Malaise**	1 (5.0%) [1]	2 (5.0%) [2]	0	0	3 (3.3%) [3]	0
**Pyrexia**	1 (5.0%) [1]	0	1 (5.0%) [1]	0	2 (2.2%) [2]	1 (2.5%) [1]
**GASTROINTESTINAL DISORDERS**	2 (10.0%) [4]	4 (10.0%) [5]	7 (35.0%) [11]	4 (40.0%) [6]	17 (18.9%) [26]	5 (12.5%) [6]
**Diarrhea**	1 (5.0%) [1]	1 (2.5%) [2]	3 (15.0%) [3]	2 (20.0%) [3]	7 (7.8%) [9]	2 (5.0%) [3]
**Abdominal pain**	0	1 (2.5%) [1]	1 (5.0%) [1]	1 (10.0%) [1]	3 (3.3%) [3]	0
**Nausea**	2 (10.0%) [2]	1 (2.5%) [1]	2 (10.0%) [2]	1 (10.0%) [1]	6 (6.7%) [6]	2 (5.0%) [2]
**Vomiting**	1 (5.0%) [1]	0	1 (5.0%) [1]	1 (10.0%) [1]	3 (3.3%) [3]	0
**Constipation**	0	0	1 (5.0%) [1]	0	1 (1.1%) [1]	0
**Dental caries**	0	1 (2.5%) [1]	0	0	1 (1.1%) [1]	0
**Dyspepsia**	0	0	0	0	0	1 (2.5%) [1]
**Fecaloma**	0	0	1 (5.0%) [1]	0	1 (1.1%) [1]	0

By System Organ Class and Preferred Term	Low Dose N= 20	Standard N= 40	High Dose N= 20	SC Injection N= 10	Combined N= 90	Control N= 40
**Number (%) of Participants Reporting any TEAE**	**n=8 (40.0%) [30]**	**n=21 (52.5%) [97]**	**n=16 (80.0%) [94]**	**n=7 (70.0%) [33]**	**n=52 (57.8%) [254]**	**n=29 (72.5%) [82]**
**Gastroesophageal reflux disease**	0	0	1 (5.0%) [1]	0	1 (1.1%) [1]	0
**Peptic ulcer**	0	0	1 (5.0%) [1]	0	1 (1.1%) [1]	0
**NERVOUS SYSTEM DISORDERS**	4 (20.0%) [4]	12 (30.0%) [22]	6 (30.0%) [6]	4 (40.0%) [6]	26 (28.9%) [38]	7 (17.5%) [9]
**Headache**	3 (15.0%) [3]	11 (27.5%) [19]	6 (30.0%) [6]	4 (40.0%) [6]	24 (26.7%) [34]	7 (17.5%) [9]
**Dizziness**	1 (5.0%) [1]	3 (7.5%) [3]	0	0	4 (4.4%) [4]	0
**INFECTIONS AND INFESTATIONS**	3 (15.0%) [3]	8 (20.0%) [13]	5 (25.0%) [7]	3 (30.0%) [3]	19 (21.1%) [26]	10 (25.0%) [11]
**Gastroenteritis**	0	1 (2.5%) [1]	1 (5.0%) [1]	1 (10.0%) [1]	3 (3.3%) [3]	0
**Upper respiratory tract infection**	0	2 (5.0%) [3]	1 (5.0%) [1]	1 (10.0%) [1]	4 (4.4%) [5]	0
**Urinary tract infection**	0	0	0	1 (10.0%) [1]	1 (1.1%) [1]	0
**COVID-19**	0	1 (2.5%) [1]	1 (5.0%) [1]	0	2 (2.2%) [2]	0
**Influenza**	1 (5.0%) [1]	3 (7.5%) [6]	1 (5.0%) [1]	0	5 (5.6%) [8]	3 (7.5%) [3]
**Lower respiratory tract infection**	0	0	1 (5.0%) [1]	0	1 (1.1%) [1]	0
**Nasopharyngitis**	1 (5.0%) [1]	0	1 (5.0%) [1]	0	2 (2.2%) [2]	1 (2.5%) [1]
**Orchitis**	0	0	0	0	0	1 (2.5%) [1]
**Otitis media**	0	0	1 (5.0%) [1]	0	1 (1.1%) [1]	0
**Pharyngitis**	0	1 (2.5%) [1]	0	0	1 (1.1%) [1]	2 (5.0%) [2]
**Rhinitis**	1 (5.0%) [1]	0	0	0	1 (1.1%) [1]	0
**Sinusitis**	0	0	0	0	0	2 (5.0%) [2]
**Subcutaneous abscess**	0	1 (2.5%) [1]	0	0	1 (1.1%) [1]	0
**Tonsillitis**	0	0	0	0	0	2 (5.0%) [2]

By System Organ Class and Preferred Term	Low Dose N= 20	Standard N= 40	High Dose N= 20	SC Injection N= 10	Combined N= 90	Control N= 40
**Number (%) of Participants Reporting any TEAE**	**n=8 (40.0%) [30]**	**n=21 (52.5%) [97]**	**n=16 (80.0%) [94]**	**n=7 (70.0%) [33]**	**n=52 (57.8%) [254]**	**n=29 (72.5%) [82]**
**INJURY- POISONING AND PROCEDURAL COMPLICATIONS**	1 (5.0%) [1]	1 (2.5%) [1]	1 (5.0%) [1]	1 (10.0%) [1]	4 (4.4%) [4]	1 (2.5%) [1]
**Injury**	0	0	0	1 (10.0%) [1]	1 (1.1%) [1]	0
**Face injury**	0	1 (2.5%) [1]	0	0	1 (1.1%) [1]	0
**Joint injury**	0	0	0	0	0	1 (2.5%) [1]
**Ligament sprain**	0	0	1 (5.0%) [1]	0	1 (1.1%) [1]	0
**Thermal burn**	1 (5.0%) [1]	0	0	0	1 (1.1%) [1]	0
**MUSCULOSKELETAL AND CONNECTIVE TISSUE DISORDERS**	1 (5.0%) [2]	5 (12.5%) [7]	4 (20.0%) [6]	1 (10.0%) [3]	11 (12.2%) [18]	6 (15.0%) [9]
**Arthralgia**	1 (5.0%) [1]	1 (2.5%) [1]	2 (10.0%) [2]	1 (10.0%) [1]	5 (5.6%) [5]	4 (10.0%) [4]
**Myalgia**	1 (5.0%) [1]	4 (10.0%) [5]	3 (15.0%) [4]	1 (10.0%) [2]	9 (10.0%) [12]	5 (12.5%) [5]
**Soft tissue injury**	0	1 (2.5%) [1]	0	0	1 (1.1%) [1]	0
**BLOOD AND LYMPHATIC SYSTEM DISORDERS**	0	2 (5.0%) [2]	0	0	2 (2.2%) [2]	0
**Neutropenia**	0	2 (5.0%) [2]	0	0	2 (2.2%) [2]	0
**EAR AND LABYRINTH DISORDERS**	0	0	1 (5.0%) [1]	0	1 (1.1%) [1]	0
**Ear pain**	0	0	1 (5.0%) [1]	0	1 (1.1%) [1]	0
**IMMUNE SYSTEM DISORDERS**	1 (5.0%) [1]	0	0	0	1 (1.1%) [1]	0
**Hypersensitivity**	1 (5.0%) [1]	0	0	0	1 (1.1%) [1]	0
**INVESTIGATIONS**	0	0	1 (5.0%) [1]	0	1 (1.1%) [1]	2 (5.0%) [2]
**Aspartate aminotransferase increased**	0	0	0	0	0	1 (2.5%) [1]
**Platelet count decreased**	0	0	1 (5.0%) [1]	0	1 (1.1%) [1]	0

By System Organ Class and Preferred Term	Low Dose N= 20	Standard N= 40	High Dose N= 20	SC Injection N= 10	Combined N= 90	Control N= 40
**Number (%) of Participants Reporting any TEAE**	**n=8 (40.0%) [30]**	**n=21 (52.5%) [97]**	**n=16 (80.0%) [94]**	**n=7 (70.0%) [33]**	**n=52 (57.8%) [254]**	**n=29 (72.5%) [82]**
White blood cell count decreased	0	0	0	0	0	1 (2.5%) [1]
METABOLISM AND NUTRITION DISORDERS	0	0	1 (5.0%) [1]	0	1 (1.1%) [1]	0
Dehydration	0	0	1 (5.0%) [1]	0	1 (1.1%) [1]	0
PREGNANCY-PUERPERIUM AND PERINATAL CONDITIONS	0	0	1 (5.0%) [1]	0	1 (1.1%) [1]	0
Pregnancy	0	0	1 (5.0%) [1]	0	1 (1.1%) [1]	0
RESPIRATORY-THORACIC AND MEDIASTINAL DISORDERS	1 (5.0%) [1]	3 (7.5%) [3]	1 (5.0%) [1]	0	5 (5.6%) [5]	2 (5.0%) [2]
Allergic sinusitis	0	1 (2.5%) [1]	0	0	1 (1.1%) [1]	0
Cough	0	0	0	0	0	1 (2.5%) [1]
Dysphonia	1 (5.0%) [1]	0	0	0	1 (1.1%) [1]	0
Nasal congestion	0	1 (2.5%) [1]	0	0	1 (1.1%) [1]	0
Oropharyngeal pain	0	1 (2.5%) [1]	0	0	1 (1.1%) [1]	0
Rhinitis allergic	0	0	0	0	0	1 (2.5%) [1]
Throat irritation	0	0	1 (5.0%) [1]	0	1 (1.1%) [1]	0
SKIN AND SUBCUTANEOUS TISSUE DISORDERS	1 (5.0%) [1]	0	1 (5.0%) [1]	0	2 (2.2%) [2]	1 (2.5%) [1]
Eczema	0	0	1 (5.0%) [1]	0	1 (1.1%) [1]	0
Pruritus	1 (5.0%) [1]	0	0	0	1 (1.1%) [1]	0
Solar dermatitis	0	0	0	0	0	1 (2.5%) [1]
VASCULAR DISORDERS	0	0	0	0	0	1 (2.5%) [1]
Hypertension	0	0	0	0	0	1 (2.5%) [1]

SAS Program: V_ae01 Date: 13APR2023 Unique Number: 8779

By System Organ Class and Preferred Term	Low Dose N= 20	Standard N= 40	High Dose N= 20	SC Injection N= 10	Combined N= 90	Control N= 40
**Number (%) of Participants Reporting any TEAE**	**n=8 (40.0%) [30]**	**n=21 (52.5%) [97]**	**n=16 (80.0%) [94]**	**n=7 (70.0%) [33]**	**n=52 (57.8%) [254]**	**n=29 (72.5%) [82]**

The number in square brackets [x] is the number of individual occurrences of a TEAE

If a participant reports the same event more than once it counts as a single participant event

Where a participant reports two or more events in the same SOC group both events are recorded but only one record is counted in the SOC group
