# Supplementary material for: Safety and immunogenicity of DNA omicron booster Alveavax-v1.2 in Ad26.COV2.S-vaccinated adults
Source: iScience. 2025 Nov 10;28(12):113970. doi: 10.1016/j.isci.2025.113970 (PMC12704268; doi:10.1016/j.isci.2025.113970)
Supplement: Data S1. Study report [file mmc2.pdf]

Data S1: Study Report

CLINICAL STUDY REPORT

A PHASE 1 OPEN-LABEL, ACTIVE-CONTROLLED, RANDOMIZED DOSE-FINDING STUDY TO EVALUATE SAFETY, TOLERABILITY, AND IMMUNOGENICITY OF INTRADERMAL AND SUBCUTANEOUS APPLICATION OF THE PLASMID DNA SARS-COV-2 OMICRON BA.2 VACCINE ALVEAVAX-V1.2 IN PRIMARY AD26.COV2.S VACCINATED HEALTHY INDIVIDUALS.

Study/protocol no.	Alvea-VAX-P00001
Study design	A first-in-human, open-label, active-controlled, randomized dose-finding study to evaluate safety, tolerability, and immunogenicity of intradermal and subcutaneous application of the plasmid DNA SARS-CoV-2 Omicron BA.2 vaccine Alveavax-v1.2 in primary Ad26.COV2.S vaccinated healthy individuals.
Test Product	Alveavax-v1.2
Development phase	1
Trial Registry number(s)	South African National Clinical Trials Registry Identifier: DOH-27-062022-5157<br>ClinicalTrials.gov Identifier: **NCT05844202**
Indication	SARS-CoV-2 BA.2/Omicron
Date of first enrollment	27 June 2022
Date that last participant completed study	28 February 2023
Sponsor	Alvea, LLC. and Telis Bioscience Inc.<br>19 Blackstone St, Cambridge MA, 02139. USA
Medical Monitor	Sriharsha Munnamgi and Drishti Pahuja (Bioclinica) from study initiation to 30 September 2022.<br>Tobias Odendahl (Alvea, LLC.) and Madeleine Lourens (Micron Research Limited) from 01 October 2022 to study close.
Sponsor's Responsible Medical Officer	Maximilian Schons (Alvea, LLC.)
Date of issue	14-June-2023, Final

The study was *conducted in accordance with the protocol of the ICH Good Clinical Practice (GCP) guidelines ethical principles, which have their origin in the Declaration of Helsinki, 2008 version, US Investigational New Drug (IND) regulations (21 Code of Federal Regulations [CFR] 56), or all local regulations (as applicable).*

1 SYNOPSIS

Name of Sponsor/Company (and Scientific and Public Contact Points, if applicable):
Alvea, LLC. and Telis Bioscience Inc., 19 Blackstone St, Cambridge, MA, 02139. USA

Name of finished product:
Alveavax-v1.2

Name of active ingredient:
BA.2 Omicron SARS-CoV-2 spike protein encoded in plasmid DNA

Title of study:
A Phase 1 open-label, active-controlled, randomized dose-finding study to evaluate safety, tolerability, and immunogenicity of intradermal and subcutaneous application of the plasmid DNA SARS-CoV-2 Omicron BA.2 vaccine Alveavax-v1.2 in primary Ad26.COV2.S vaccinated healthy individuals.

Study/protocol number:
Alvea-VAX-P00001

Trial registry name and number:
South African National Clinical Trials Registry Identifier: DOH-27-062022-5157
ClinicalTrials.gov Identifier: NCT05844202

Pediatric investigation plan (PIP) number:
Not applicable

Principal/coordinating Investigators:
7 investigators (see Table 5.1 and refer to Appendix 15.1.4 for a listing of investigators)

Study center(s):
7 (± 3) sites in South Africa with access to local or centralized laboratories for protocol-mandated safety and immunogenicity evaluation (for a list of investigative sites, refer to Appendix 15.1.4)

Publication (reference) (if any):
No publications have resulted from this study to date

**Study period (years):**	**Phase of development:** 1
First participant enrolled: 27 June 2022<br>*Last participant completed*: 28 February 2023	

Reporting period:
First data collection date: 30 June 2022
Last data collection date: 31 March 2023

Background and rationale for the study:
In 2019, severe acute respiratory syndrome coronavirus 2 (SARS-CoV-2) rapidly spread around the world, resulting in the ongoing coronavirus disease 2019 (COVID-19) pandemic. In 2021, the SARS-CoV-2 Omicron variant (B.1.1.529 lineage) was reported as a novel variant of concern. Two descendants of the Omicron lineage, BA.1 and BA.2 are in current circulation and have spread globally. There are currently no licensed vaccines optimized for either Omicron variant. Alvea, LLC. has developed a plasmid deoxyribonucleic acid vaccine, Alveavax-v1.2, intended to prevent symptomatic disease caused by these dominant circulating Omicron variants, when used as a booster in previously immunized people.

Objectives:
As this was first-in-human, Phase 1, dose-finding trial, the primary objective was to report the endpoints for safety, followed by the secondary objective to report on the immunogenicity and efficacy endpoints.

Name of Sponsor/Company (and Scientific and Public Contact Points, if applicable):
Alvea, LLC. and Telis Bioscience Inc., 19 Blackstone St, Cambridge, MA, 02139. USA

Name of finished product:
Alveavax-v1.2

Name of active ingredient:
BA.2 Omicron SARS-CoV-2 spike protein encoded in plasmid DNA

Primary (Safety)

- To evaluate the safety and tolerability of Alveavax-v1.2 in healthy participants, compared to a control booster vaccine.

Secondary (Immunogenicity and Efficacy)

- To evaluate the immunogenicity as humoral immune response against SARS-CoV-2 BA.2/Omicron after a booster dose of Alveavax-v1.2
- To evaluate the clinical efficacy against SARS-CoV-2 after a booster dose of Alveavax-v1.2
- To evaluate success rate of intradermal (ID) injections

Exploratory

- To evaluate the cell-mediated immune response against SARS-CoV-2 BA.2/Omicron after a booster dose of Alveavax-v1.2
- To evaluate constant fragment (Fc) effector functions against SARS-CoV-2 after a booster dose of Alveavax-v1.2
- To evaluate the humoral immune response against additional SARS-CoV-2 variants and sub-lineages after a booster dose of Alveavax-v1.2
- To correlate clinical efficacy with neutralizing antibody response
- To correlate clinical efficacy and immunogenicity with anti-nucleocapsid protein antibodies

Methodology:

Open-label, active-controlled, randomized dose-finding study in primary Janssen Ad26.COV2.S vaccinated healthy individuals. Participants were randomly allocated to one of five treatment arms to receive Alveavax-v1.2 or a Janssen Ad26.COV2.S control booster vaccine:

1.a: Low dose: 0.5 mg Alveavax-v1.2 in one ID injection

1.b: Standard dose: 2 mg Alveavax-v1.2 in one ID injection

1.c: High dose: 8 mg Alveavax-v1.2 as four ID injections of 2 mg

1.d: Subcutaneous (SC) injection: 8 mg Alveavax-v1.2 as a single SC injection

1.e: Control: Janssen Ad26.COV2.S control booster as a single intramuscular (IM) injection

Each participant was administered a booster vaccine on Day 1 of the study and monitored afterwards for local or systemic reactions to the vaccine. The first 10 participants were randomized into the low dose arm (1.a) and the control arm (1.e). No more than five participants were vaccinated on the first day, and subsequent recruitment of the study groups or escalation between dose levels was allowed only after an independent medical monitor had reviewed at least 24-hour post-dose safety data.

Then, the remaining participants assigned to the low and standard dose (1.b) cohorts, in addition to those assigned to the control arm were recruited. In parallel, the first five participants of the high dose (1.c) arm were enrolled. Further recruitment and the subcutaneous injection arm (1.d) were started after an independent medical monitor reviewed 24-hour safety data of the high dose arm.

Participants measured the diameter of local reactions using a measurement device for erythema or swelling, and for the first seven days, monitored temperature daily, and recorded any local pain at the injection site and the size of swelling or erythema. Any other adverse events were also recorded, and the severity measured.

Name of Sponsor/Company (and Scientific and Public Contact Points, if applicable):
Alvea, LLC. and Telis Bioscience Inc., 19 Blackstone St, Cambridge, MA, 02139. USA

Name of finished product:
Alveavax-v1.2

Name of active ingredient:
BA.2 Omicron SARS-CoV-2 spike protein encoded in plasmid DNA

Assessed points were Study Day 7, Day 14, Day 28, Day 84, and Day 168.

Number of participants (planned and analyzed):
A total of 130 in 5 groups with 10–40 participants each

Diagnosis and main criteria for inclusion and exclusion:

Inclusion criteria

- Healthy adult male and female volunteers between 18 and 65 years of age, inclusive.
- Participants who received a primary Janssen Ad26.COV2.S COVID-19 vaccine against SARS-CoV-2.≥ 60 days prior to receiving the study vaccine on Day 1 in this study.

Exclusion criteria

- Received any SARS-CoV-2 vaccination other than a single Janssen Ad26.COV2.S COVID-19 vaccine or plans to receive any additional SARS-CoV-2 vaccination within 90 days after the study vaccine administration (Day 1).

Test product, dose and mode of administration, batch number:
Alveavax-v1.2, filled at 5 ± 0.5 mg/mL, as an ID or SC injection at doses of 0.5 mg. 2 mg, or 8 mg
Lot/Batch number: Alveavax-v1.2.22E010

Duration of treatment:
Single dose

Control product, dose and mode of administration, batch number(s):
Janssen Ad26.COV2.S, 8.92 log_{10} infectious units/0.5 mL, in one IM injection
Batch: XE496

Endpoints:

Primary (Safety) at Day 1, Day 7, Day 28, after 3 months and/or up to 6 months:

1. Number of participants with solicited local and systemic adverse events (AEs) within 7 days of dose administration
2. Number of participants with unsolicited AEs within 28 days of vaccination
3. Number of participants with any serious adverse events (SAEs), adverse events of special interest (AESIs), and AEs leading to withdrawal during the entire period of study

Secondary (Immunogenicity and Efficacy)

- Characterization of the humoral immune response of booster vaccinations against SARS-CoV-2 BA.2/Omicron measured at Baseline (pre-vaccination; except for Point 1 below) and on Day 28, using the following:
    1. Change in geometric mean titer (GMT) of anti-spike protein (S) immunoglobulin G (IgG) antibody
    2. GMT of anti-spike protein (S) IgG antibody
    3. Geometric mean fold rise (GMFR) of anti-spike protein (S) IgG antibody
- clinical efficacy measured on Day 7, Day 14, Day 28, Day 84, and Day 168, using the WHO clinical progression scale for COVID-19

Name of Sponsor/Company (and Scientific and Public Contact Points, if applicable):
Alvea, LLC. and Telis Bioscience Inc., 19 Blackstone St, Cambridge, MA, 02139. USA

Name of finished product:
Alveavax-v1.2

Name of active ingredient:
BA.2 Omicron SARS-CoV-2 spike protein encoded in plasmid DNA

- the success rate of ID injections as measured by the absolute number and fraction of ID injections that generated a clearly demarcated bleb, of ≥ 1 mm and ≥ 7 mm in diameter, clearly visible for at least 20 seconds, for 0.5 mg and 2 mg Alveavax-v1.2, respectively

Exploratory (see also **Table 7.1**)

- Characterization of cellular immune response of booster vaccinations against SARS-CoV-2 ancestral and BA.2/Omicron:
    1. Spike-specific CD4+ T-cell response
    2. Change in spike-specific CD4+ T-cell response
    3. Spike-specific CD8+ T-cell response
    4. Change in spike-specific CD8+ T-cell response
- Fc effector functions against Omicron/BA.2, ancestral variants (such as Alpha, Beta, Delta), and additional Omicron subvariants (such as BA.1):
    1. Change in relative light units (RLU), and
    2. RLU of:
        a. antibody dependent cellular cytotoxicity
        b. antibody dependent cellular phagocytosis
        c. antibody dependent cellular trogocytosis
        d. complement deposition
        e. Fc dimer receptor binding
- Humoral immune response of booster vaccinations against ancestral, existing and yet to be defined variants (such as Alpha, Beta, Delta), and existing and yet to be defined sub-lineage (such as Omicron BA.1) SARS-CoV-2 strains:
    1. Change in GMT
    2. GMT
    3. GMFR of:
        a. serum anti-SARS-CoV-2 neutralizing antibodies
        b. serum anti-S IgG antibodies
        c. serum anti-S-RBD IgG antibodies
- Correlation of clinical efficacy with neutralizing antibody response:
    1. For each neutralizing antibody type:
        a. Percentage of participants with neutralization IC_{50} >64 IU/mL against the respective strain
        b. Percentage of participants with neutralization IC_{50} >128 IU/mL against the respective strain
- Correlation of clinical efficacy and immunogenicity with anti-nucleocapsid protein antibodies:
    1. Serologic change in GMT and GMFR between baseline and other blood samples for anti-nucleocapsid protein (N) IgG antibodies

Statistical methods:

Name of Sponsor/Company (and Scientific and Public Contact Points, if applicable):
Alvea, LLC. and Telis Bioscience Inc., 19 Blackstone St, Cambridge, MA, 02139. USA

Name of finished product:
Alveavax-v1.2

Name of active ingredient:
BA.2 Omicron SARS-CoV-2 spike protein encoded in plasmid DNA

As the study was a Phase 1 study primarily assessing safety, all data were analyzed descriptively without a formal statistical hypothesis.

Primary (Safety) endpoints

- The overall number of participants and incidence proportion (number of participants experiencing any AEs by the total number of participants) of any solicited local and systemic AEs within seven days of dose administration were presented. The incidence of each solicited AE (at MedDRA system organ class and preferred term level) to also be shown.
- The overall number and incidence proportion of unsolicited AEs within 28 days of vaccination were analyzed in a similar fashion.
- SAEs, AESIs, and AEs leading to participant discontinuation were presented in the form of a listing, with number and proportion for each one. In addition, the overall incidence proportion of these categories of AE were shown.
- For participant level tabulation a maximum intensity and highest relationship to investigational drug were presented in separate tables.

Secondary (Immunogenicity and Efficacy) endpoints

- Description of GMT, change in GMT, and GMFR of serum anti-spike protein (S) IgG antibody and the change in GMT and GFMR of anti-N protein (N) IgG antibody at Day 28.
- The number and percentage of participants found to be SARS-CoV-2 positive by external testing (score 1-10), number and percentage of participants with ambulatory mild disease (score 1-3), number and percentage of participants hospitalized with moderate disease (score 4-5), number and percentage of participants hospitalized with severe disease (score 6-9), number and percentage of participants who died (score 10) while being SARS-CoV-2 positive with the exact 95% CI were also presented separately for each Study Day 7, Day 14, Day 28, Day 84, Day 168.

Exploratory analysis

- Characterization of humoral antibody immune response of booster vaccinations against ancestral and Variant (Alpha, Beta, Delta, BA.1, and any newly discovered variant of concern [VOC]) SARS-CoV-2 Strains measured using GMT of anti-SARS-CoV-2 neutralizing antibody at each timepoint (Pre-vaccination, Day 7, Day 14, Day 28, Day 84, and Day 168) were made using the approach described for the primary endpoint analyses.
- Characterization of the cellular immune response following booster vaccinations against SARS-CoV-2 BA.2/Omicron measured using cellular immune responses CD4+ and CD8+ T-cell response.
- The RLU and change in RLU of serum antibody-dependent cell-mediated cytotoxicity (ADCC) against existing and yet to be defined variants and sub-lineages at the defined time points were presented unadjusted in tabular form.

Summary of Results and Conclusions:

Participant disposition

130 Participants were enrolled in the study and vaccinated with either the Alveavax-v1.2 vaccine or the Janssen Ad26.COV2.S vaccine. The study groups and number of participants were as follows:

- Group 1.a: Low dose group, 0.5 mg Alveavax-v1.2 in one ID injection (20 participants)

**Name of Sponsor/Company (and Scientific and Public Contact Points, if applicable):**<br>Alvea, LLC. and Telis Bioscience Inc., 19 Blackstone St, Cambridge, MA, 02139. USA
**Name of finished product:**<br>Alveavax-v1.2
**Name of active ingredient:**<br>BA.2 Omicron SARS-CoV-2 spike protein encoded in plasmid DNA

- Group 1.b: Standard dose group, 2 mg Alveavax-v1.2 in one ID injection (40 participants)
- Group 1.c: High dose group, 8 mg Alveavax-v1.2 as four ID injections of 2 mg (20 participants)
- Group 1.d: SC injection group, 8 mg Alveavax-v1.2 as a single SC injection (10 participants)
- Group 1.e: Control booster group, Janssen Ad26.COV2.S, 8.92 log_{10} infectious units/0.5 mL, as a single IM injection (40 participants)

The analyzed study populations were as follows:

- modified Intent-To-Treat (mITT) and Safety populations: N = 120
  - 10 of the 130 enrolled participants did not complete the study due to being lost to follow-up (N = 9) or withdrawal by the participant (N = 1).
- The Per Protocol (PP) population: N = 116
  - 7 of the 130 enrolled participants were excluded from the PP population for protocol deviations: 6 participants were from the Alveax-1.2 dose groups and one participant received the control. Four participants did not receive the full dose on vaccination and three participants did not have a Day 28 immunogenicity result. The PP population was used only for the immunogenicity analysis.

Demography and baseline characteristics

There were no noteworthy differences in the demography or baseline characteristics between the Alveavax-v1.2 dose groups or control group. The treatment groups were similar for all parameters.

Primary (Safety) results

- Included data were from AEs, a laboratory screen, vital signs, and symptom severity resulting from vaccination.
- There was a total of 3 SAEs, all recorded for a single participant (a severe lower respiratory infection requiring hospitalization, a fecaloma which resolved, and a pregnancy which was ongoing at the end of the study). None of the events started within the first seven days following vaccination.
- There were no AESIs or deaths during the study.
- The hematology data were similar between baseline and Study Day 7, with the majority of participants within the normal range at Day 7.
- A small number of participants had raised liver function tests above normal (ALT, N = 7; or AST, N = 10) at baseline but there was no indication of any further increase following treatment.
- There were no significant changes in any vital signs between baseline and Day 7, following vaccination.
- From Day 1 (vaccination) to Day 6 post-vaccination, most participants recorded only mild to moderate signs and symptoms which readily resolved.
- Mild to moderate pain lasted up to Day 6 in a small number of participants (mild, N = 2; or moderate, N = 2), and moderate to severe fatigue was experienced by a subset of high dose group participants (moderate, N = 2; or severe, N = 1) at Day 5 and Day 6 .

Secondary (Immunogenicity and Efficacy) results

Name of Sponsor/Company (and Scientific and Public Contact Points, if applicable):
Alvea, LLC. and Telis Bioscience Inc., 19 Blackstone St, Cambridge, MA, 02139. USA

Name of finished product:
Alveavax-v1.2

Name of active ingredient:
BA.2 Omicron SARS-CoV-2 spike protein encoded in plasmid DNA

- The main immunogenicity variable was the fold change in GMT from baseline of serum anti-SARS-CoV-2 BA.2 antibody in the PP population.
- As the study was halted after 6 months and planned laboratory analyses were truncated, only the Day 28 data were sufficient for most of the treatment groups.
- The increase in GMT of serum anti-SARS-CoV-2 BA.2 antibody at Day 28 was lower than the expected 2-to-4-fold increase, and was approximately 1 for all the study groups, although 3 participants did not have a value recorded at baseline.
- The response in the control group, vaccinated with Janssen Ad26.COV2.S, was also below expected, with an increase of 164.6 in the GMT of serum anti-SARS-CoV-2 BA.2 antibody, and a fold increase of 1.31.
- The main efficacy variable was a repeat of the immunogenicity analysis, but in the mITT population, with two additional participants in the low dose group.
- As for the immunogenicity findings, but among the mITT population, there was little difference from baseline GMT for the anti-SARS-CoV-2 BA.2 antibody, and also for the anti-nucleocapsid protein antibody.
- Likewise, the control group failed to demonstrate a marked increase in GMT among the mITT population.

Exploratory analysis results

These results were not collected due to the lack of immunogenic or efficacy response at Day 28, according to the titers of anti-SARS-CoV-2 BA.2 antibodies in any of the study cohorts, or in the neutralizing antibodies in a subset of samples. It was decided to stop all further exploratory analysis.

Conclusions

The safety data showed that the Alveavax-1.2 vaccine was generally safe and well tolerated, with no SAEs causally related to the vaccine. The study was planned to end after 12 months but, due to suboptimal results at the interim analysis, the study was stopped after 6 months, and the immunogenicity and efficacy results were assessed at Day 28. The immunology data before Day 28 was incomplete due to the termination of further laboratory testing at other timepoints in order to reduce costs. Other measures of cellular immunogenicity were all omitted; only the GMT of anti-SARS-CoV-2 BA.2 antibody and anti-nucleocapsid protein antibody data were sufficient to form any analysis and conclusions. Participants continued to be monitored for safety for up to 6 months after completion of all study vaccinations.

Date and version of this report:
14-June-2023, Final

2 TABLE OF CONTENTS FOR THE INDIVIDUAL CLINICAL STUDY REPORT

Section **Page**

1 SYNOPSIS 2
2 TABLE OF CONTENTS FOR THE INDIVIDUAL CLINICAL STUDY REPORT 9
3 LIST OF ABBREVIATIONS AND DEFINITION OF TERMS 13
4 ETHICS 15
4.1 Independent Ethics Committee (IEC) or Institutional Review Board (IRB) 15
4.2 Ethical Conduct of the Study 15
4.3 Participant Information and Consent 15
5 INVESTIGATORS AND STUDY ADMINISTRATIVE STRUCTURE 16
6 INTRODUCTION 18
6.1 Rationale and Aims 18
6.2 Background 18
6.2.1 COVID-19 Omicron 18
6.2.2 Protection from Omicron 19
6.2.3 DNA vaccines 20
7 STUDY OBJECTIVES AND ENDPOINTS 21
7.1 Objectives 21
7.2 Endpoints 21
8 INVESTIGATIONAL PLAN 24
8.1 Overall Study Design and Plan 24
8.2 Discussion of Study Design, including the Choice of Control Groups 27
8.2.1 Risk/Benefit Assessment 28
8.3 Selection of Study Population 29
8.3.1 Inclusion Criteria 30
8.3.2 Exclusion Criteria 31
8.3.3 Removal of Participants from Therapy or Assessment 32
8.3.4 Stopping or Suspending the Study 33
8.4 Treatment 35
8.4.1 Treatments Administered 35
8.4.2 Identity of Investigational Product(s) 35
8.4.3 Avoidance of Bias 36
8.4.4 Selection of Dose(s) and Timing of Each Dose for Each Participant 36
8.4.5 Treatment Compliance 37
8.4.6 Prior and Concomitant Therapy 37
8.5 Safety, Immunogenicity and Efficacy Variables 39
8.5.1 Safety, Immunogenicity and Efficacy Assessments and Schedule of Assessments 39
8.6 Data Quality Assurance 44
8.7 Statistical Analysis Methods Planned in the Protocol and Determination of Sample Size 45
8.7.1 Statistical Plans 45
8.7.2 Determination of Sample Size 48
8.8 Changes in the Conduct of the Study or Planned Analyses 48
8.8.1 Changes in the Conduct of the Study 48
8.8.2 Changes in the Planned Analysis 58
9 STUDY PARTICIPANTS 59
9.1 Disposition of Participants 59
9.1.1 Study populations 61
9.1.2 Number of Participants at Each Visit 62
9.2 Protocol Deviations 62
9.3 Demographic and Other Baseline Characteristics 63
9.3.1 Demographics and Baseline Disease Characteristics 63

9.3.2 Medical History and Concurrent Illness 64
9.3.3 Prior and Concomitant Treatments 65
9.4 Measurements of Treatment Compliance 65
9.5 Extent of Exposure 65
10 SAFETY EVALUATION 66
10.1 ADVERSE EVENTS 66
10.1.1 Brief Summary of Adverse Events 66
10.1.2 Related Adverse Events 67
10.1.3 Categorization of All Adverse Events 67
10.2 ANALYSIS OF DEATHS, OTHER SERIOUS ADVERSE EVENTS, AND OTHER CLINICALLY MEANINGFUL ADVERSE EVENTS 67
10.2.1 Deaths, Other Serious Adverse Events, Discontinuation due to Adverse Events and Other Adverse Events of Special Interest 67
10.3 CLINICAL LABORATORY EVALUATION 67
10.3.1 Individual Laboratory Measurements by Participant and Abnormal Laboratory Values 67
10.3.2 Evaluation of Laboratory Values 67
10.4 Vital Signs, Physical Examinations, and Other Observations Related to Safety 68
10.4.1 Vital Signs 68
10.4.2 Abnormal Physical Examination Findings 68
10.4.3 Other Observations Related to Safety 68
10.5 Safety Results Summary 69
11 IMMUNOGENICITY AND EFFICACY EVALUATIONS 70
11.1 IMMUNOGENICITY AND EFFICACY RESULTS 70
11.1.1 Immunogenicity Endpoints 70
11.1.2 Efficacy Endpoints 71
11.2 Results of Statistical Issues Encountered During the Analysis 71
11.2.1 Adjustments for Covariates 71
11.2.2 Handling of Withdrawals, Discontinuations or Missing Data 71
11.2.3 Interim Analyses and Data Monitoring 71
11.2.4 Multicenter Studies 71
11.2.5 Multiple Comparisons/Multiplicity 71
11.2.6 Use of an “Efficacy Subset” of Participants 72
11.2.7 Examination of Subgroups 72
11.2.8 Tabulation of Individual Response Data 72
11.3 Immunogenicity and Efficacy Results Summary 72
12 DISCUSSION AND OVERALL CONCLUSIONS 73
12.1 Discussion 73
12.2 Conclusions 74
13 TABLES 75
13.1 DEMOGRAPHIC DATA 75
13.2 SAFETY DATA 93
13.2.1 Displays of Adverse Events 93
13.2.2 Listings of Deaths, Other Serious and Clinically Meaningful Adverse Events 107
13.2.3 Data Listings (Each Participant) for Abnormal Clinically Meaningful Laboratory Values, Vital Signs, Physical Examinations and Other Observations Related to Safety 108
13.3 IMMUNOGENICITY AND EFFICACY DATA 157
13.4 EXPLORATORY DATA 164
14 REFERENCE LIST 165
15 APPENDICES 169

LIST OF IN-TEXT TABLES

Table 5.1: Investigator and Study Administrative Structure (Protocol Number: Alvea-VAX-P00001) 16
Table 7.1: Exploratory Analysis Endpoints (Protocol Number: Alvea-VAX-P00001) 22
Table 8.1: Dosing Schema (Protocol Number: Alvea-VAX-P00001) 24
Table 8.2: Evaluation of Risk (Protocol Number: Alvea-VAX-P00001) 28
Table 8.3: Study intervention (s) administered (Protocol Number: Alvea-VAX-P00001) 35
Table 8.4: Schedule of Assessments (Protocol Number Alvea-VAX-P00001) 39
Table 8.5: Protocol amendments (Protocol Number: Alvea-VAX-P00001) 49
Table 9.1: Number of Participants Included in the Efficacy Analysis (Protocol Number: Alvea-VAX-P00001) 61
Table 9.2: Number (%) of Participants Assessed at Each Visit – mITT Population (Protocol Number: Alvea-VAX-P00001) 62
Table 9.3: Reasons for Premature Study Termination – mITT Population (Protocol Number: Alvea-VAX-P00001) 62
Table 9.4: Summary of Major Protocol Deviations – mITT Population (Protocol Number: Alvea-VAX-P00001) 63
Table 9.5: Participant Demography and Baseline Characteristics – mITT Population (Protocol Number: Alvea-VAX-P00001) 64
Table 10.1: Overview of TEAEs – Safety Population (Protocol Number: Alvea-VAX-P00001) 66
Table 11.1: Change from Baseline of ELISA BA.2 Antibody Titer (EC_{50}) – Per Protocol Population 70

LIST OF IN-TEXT FIGURES

Figure 8.1: Study Design Schema (Protocol Number: Alvea-VAX-P00001)....................26
Figure 9.1: Disposition of Participants (Protocol Number: Alvea-VAX-P00001) 60

3 LIST OF ABBREVIATIONS AND DEFINITION OF TERMS

AE	adverse event
AESI	adverse event of special interest
ALT	alanine transaminase
AST	aspartate transaminase
BMI	body mass index
BPM	beats per minute
BUN	blood urea nitrogen
cm	centimeters
COVID-19	Coronavirus Disease 2019
CRF	case report form
DNA	Deoxyribonucleic Acid
EC_{50}	half-maximal effective response
eCRF	electronic case report form
ELISA	Enzyme-Linked Immunosorbent Assay
EUA	Emergency Use Authorization
Fc	constant fragment
FDA	U.S. Food and Drug Administration
GCP	Good Clinical Practice
GMFR	geometric mean fold rise
GLP	Good Laboratory Practice
GMT	geometric mean titer
HIV	Human Immunodeficiency Virus
IC_{50}	half-maximal inhibitory concentration
ICF	informed consent form
ICH	International Conference for Harmonisation
ID	intradermal
IEC	independent ethics committee
IgG	immunoglobulin G
IM	intramuscular
IND	investigational new drug
IRB	Institutional Review Board
kg	kilogram
MedDRA	Medical Dictionary for Regulatory Activities
mITT	modified intent-to-treat
mRNA	messenger ribonucleic acid
NP	nasopharyngeal

PCR	Polymerase Chain Reaction
PK	Pharmacokinetics
PP	Per Protocol
QA	quality assurance
RBC	red blood cell
RBD	receptor-binding domain
RLU	relative light units
RNA	ribonucleic acid
SAE	serious adverse event
SAHPRA	South African Health Products Regulatory Authority
SAP	statistical analysis plan
SAS	Statistical Analysis System
SC	subcutaneous
SD	standard deviation
TEAE	treatment emergent adverse event
VOC	variant of concern
WBC	white blood cell
WHO	World Health Organization

4 ETHICS

4.1 INDEPENDENT ETHICS COMMITTEE (IEC) OR INSTITUTIONAL REVIEW BOARD (IRB)

In accordance with local requirements, this study was submitted to the regulatory authorities for approval/notification. The Protocol, corresponding case report form (CRF), and informed consent form (ICF) were reviewed by the IEC prior to initiating the study. The first participant was enrolled only after the IEC approved the Protocol and CRF for the study. IEC notifications as per GCP guidelines issued by the WHO were followed during the conduct of the study. Details of the IEC/IRB consulted are provided in **Appendix 15.1.3**.

4.2 ETHICAL CONDUCT OF THE STUDY

This study was conducted in full compliance with the protocol of the International Council for Harmonisation (ICH) Good Clinical Practice (GCP) guidelines ethical principles, which have their origin in the Declaration of Helsinki, 2008 version, US Investigational New Drug (IND) regulations (21 Code of Federal Regulations [CFR] 56), or all local regulations (as applicable).

The Investigator ensured that the study was conducted in accordance with the provisions, as stated in the ICH GCP guidelines, and complied with prevailing local laws and regulations. Furthermore, the participation of subjects in this study was reported to the appropriate local data protection agencies, in accordance with GCP. The principal Investigator ensured that appropriate training relevant to the study was given to the medical, nursing, and other staff involved. Any information relevant to the performance of this study was forwarded to the co-investigators and other staff involved.

4.3 PARTICIPANT INFORMATION AND CONSENT

Before the study began, the Investigator and/or designated study site personnel explained the purpose, procedures to be followed, potential hazards, available alternative therapeutic modalities, and rights of the participants (in English or in a language understandable to the participant). Prior to enrollment of the participant in the study, the participants were required to understand and sign the ICF (in English or in a language understandable to the participants), and a signature of an impartial witness (if applicable) and the study personnel obtaining consent was required. Each person's signature, as well as the date, was included. A sample ICF and CRF (unique pages only) are included in **Appendix 15.1.2**.

Written informed consent was obtained from each participant prior to enrollment in the study and before any protocol-directed procedures were performed. A unique participant identification number (participant number) was assigned to each participant at the time; this participant number was used throughout the study.

5 INVESTIGATORS AND STUDY ADMINISTRATIVE STRUCTURE

Table 5.1: Investigator and Study Administrative Structure (Protocol Number: Alvea-VAX-P00001)

**Sponsor**	Alvea, LLC. / Telis Bioscience Inc.<br>19 Blackstone St, Cambridge, MA, 02139. USA
**Contract research organization**	Micron Research Limited<br>109B, Lancaster Way Business Park, Ely, Cambridgeshire. CB6 3NX. U.K.
**Medical monitor**	Sriharsha Munnamgi and Drishti Pahuja (Bioclinica) from study initiation to 30 September 2022.<br><br>Tobias Odendahl (Alvea, LLC.) and Madeleine Lourens (Micron Research Limited) from 01 October 2022 to study close.
**Principal/Coordinating investigator**	Dr Veronique de Jager, South Africa (refer to **Appendix 15.1.4** for details)
**Other investigators**	Refer to **Appendix 15.1.4**
**Pharmacovigilance:**	Alvea, LLC.
**Central laboratory**	Cytespace<br>125 Amkor Road, Lyttelton Manor, Centurion, Gauteng, 0157, South Africa<br><br>Bioanalytical Research Corporation (BARC) – PBMC processing, 11 Napier Road, Richmond, Johannesburg, Gauteng, South Africa<br><br>The Moore laboratory at the National Institute for Communicable Diseases, HIV & SARS-CoV-2 Virology Section, Centre for HIV & STI's, 1 Modderfontein Road, Sandringham, Johannesburg, 2131, South Africa<br><br>(refer to **Appendix 15.1.4**/**Appendix 15.1.10** for details)
**Microbiology laboratory**	N/A
**Central ECG monitoring**	N/A
**Data monitoring committee**	Independent Medical Monitors<br><br>Dr Roland van Rensburg<br>Independent Medical Monitor (South Africa)

	E-mail: rolandmed@gmail.com<br>Tel: +27 83 944 1095<br><br>Dr. med. Jannik Stemler<br>Assistenzarzt | Wissenschaftlicher Mitarbeiter<br>University Hospital Cologne, Internal Medicine<br>E-mail: jannik.stemler@uk-koeln.de
**Randomization and distribution of study supplies**	Randomization: Clario (Bioclinica IRT)<br><br>Distribution of study supplies: LogicTrials
**Analytical laboratory**	The Moore laboratory at the National Institute for Communicable Diseases, HIV & SARS-CoV-2 Virology Section, Centre for HIV & STI's, 1 Modderfontein Road, Sandringham, Johannesburg, 2131, South Africa
**Pharmacokinetics**	None
**Statistics**	Micron Research Limited

Details of the affiliation, role in the study and qualifications of each investigator and key study personnel are included in **Appendix 15.1.4**.

6 INTRODUCTION

6.1 RATIONALE AND AIMS

This is a first-in-human, Phase 1, dose-finding trial to describe the safety, tolerability, and immunogenicity of Alveavax-v1.2 in primary vaccinated individuals. This investigative product is a Severe Acute Respiratory Syndrome Coronavirus 2 (SARS-CoV-2) Booster Vaccine candidate optimized for Omicron/BA.2. There are currently no licensed, variant-optimized booster vaccines to prevent infection with SARS-CoV-2 Omicron/BA.2. Approved or authorized booster vaccines are expensive, require a stringent cold chain, and have large-scale manufacturing issues, resulting in very limited availability in low- and middle-income countries. Given the rapid global spread of the Omicron variant, the rapid development of an easily distributable and affordable booster vaccine is of great importance.

The overall safety, tolerability and immunogenicity, including humoral and cell-mediated immunity, of Alveavax-v1.2 was assessed against SARS-CoV-2 variants. In a first-in-human dose-finding study design, the immune responses of three dose intradermal (ID) levels in vaccinated individuals were investigated, which could be used to inform a potential Phase 2 immunobridging trial. In addition, the feasibility and ease of ID vaccine administration was assessed, as well as the safety and immune response of the subcutaneous (SC) administration of ID injections. As a comparator, the approved Janssen Ad26.COV2.S COVID-19 vaccine was administered, which has been proven to be effective against SARS-CoV-2 Omicron infections, and could be sourced for the purposes of this trial.

6.2 BACKGROUND

6.2.1 COVID-19 Omicron

SARS-CoV-2, a novel coronavirus, was first reported in December 2019. The disease caused by SARS-CoV-2 was officially named by the World Health Organization (WHO) as coronavirus disease 2019 (COVID-19). The virus is highly transmissible between humans and has spread rapidly, causing the ongoing COVID-19 pandemic **[1,2]**. As of 31st May 2023, more than 765 million cases have been confirmed and almost 7 million lives claimed worldwide **[3]**.

The SARS-CoV-2 Omicron variant (B.1.1.529 lineage) was reported by the WHO in November 2021 as a novel variant of concern (VOC) with a number of mutations and immune evasive potential **[4]**. This variant harbors up to 59 mutations throughout its genome, over 30 of which are in the spike protein: the mediator of host cell entry and main target of neutralizing antibodies **[4]**. The Omicron variant is highly transmissible and has quickly become dominant in many parts of the world, increasing infections and straining healthcare systems. The current global epidemiology of SARS-CoV-2 is characterized by the dominance of the Omicron variant on a global scale **[5]**.

The Omicron variant has a significant replication advantage, a higher secondary attack rate, and evades humoral immunity induced by either infection or currently existing vaccination to a greater extent than previous variants **[6]**. Although the individual risk for severe disease with Omicron is lower than with other variants, the increased number of infections still translates to high absolute numbers of hospitalizations and deaths due to COVID-19 **[6]**. Omicron BA.1 (Nextstrain clade 21K) became the dominant variant in January 2022 in

countries for which there was strain typing capacity and was followed by the Omicron BA.2 variant (Nextstrain clade 21L) **[7]**.

BA.2's growth advantage is caused by higher transmission than BA.1, and potentially increased immune escape **[1]**. BA.2 has a similar number of mutations to BA.1 in the spike protein, spike receptor-binding domain (RBD), and overall **[8]**, although it only shares 32/60 of its mutations with the BA.1 strain **[4]**. This means that BA.2 has a greater divergence from its sister lineage, BA.1, than most other designated variants from the wild-type virus. Early data indicate that BA.2 has similar severity to BA.1 **[9]**.

6.2.2 Protection from Omicron

Vaccines are generally considered the most promising approach to mitigate the pandemic **[10]**. In August 2021, the U.S. Food and Drug Administration (FDA) approved a messenger ribonucleic acid (mRNA) vaccine (Pfizer-BioNTech/Comirnaty) as a two-dose series for prevention of symptomatic COVID-19 in persons aged ≥16 years and an Emergency Use Authorization (EUA) in those aged 12–15 years. A second mRNA vaccine (Moderna), as well as a recombinant, replication-incompetent adenovirus serotype 26 (Ad26) vector vaccine (Janssen vaccine [Johnson & Johnson] **[11]**), is authorized under an EUA for use in persons aged ≥18 years. Various regulatory agencies worldwide have approved these and other SARS-CoV-2 vaccines. All approved or authorized COVID-19 vaccines demonstrated efficacy (range 51% to 95%) against symptomatic, laboratory-confirmed COVID-19 in adults ≥18 years old **[12]**. Available evidence suggests that the currently approved or authorized COVID-19 vaccines are highly effective against severe disease, hospitalization, and death for a variety of strains, including Alpha (B.1.1.7), Beta (B.1.351), Gamma (P.1), and Delta (B.1.617.2) **[12]**.

Studies of SARS-CoV-2 variants have demonstrated that mutations within the RBD mediate escape from vaccine-induced neutralizing antibodies **[13,14,15]**. While primary vaccination series with vaccines against the ancestral strain have shown severely reduced efficacy against Omicron, recent studies for the mRNA and Janssen boosters have demonstrated efficacy against hospitalization **[12,16]**.

However, since the EUA of the first COVID-19 vaccine, only 30.1% of people in low-income countries have had at least one vaccine dose (as of 5th June 2023) **[17]**. Besides cost and dose availability, worldwide distribution of mRNA vaccines is limited by the requirement for cold chain storage and shipment **[18]**. According to the current literature, no emergency-use listed COVID-19 vaccine can be transported at room temperature.

There is a pressing need for vaccines that protect against the BA.2 Omicron strain and can be quickly and widely distributed. While the epidemiology or variants of SARS-CoV-2 cannot be comfortably predicted, the higher reproductive number of Omicron BA.2 coupled with breakthrough infections in those vaccinated may lead to future waves. New variants, should they arise, may be antigenically like the higher fitness Omicron lineage. These observations suggest that Omicron-optimized vaccination may be valuable. A cheap, easy to manufacture, and shelf-stable BA.2 vaccine could reduce the burden of the ongoing wave of Omicron SARS-CoV-2 and protect those in low- and middle-income countries.

6.2.3 DNA vaccines

Deoxyribonucleic acid (DNA) vaccines were first developed in the early 1990s and have shown safety and immunogenicity in both preclinical and clinical trials **[19,20]**. DNA vaccines are being developed and tested for a diverse set of infectious diseases like human immunodeficiency virus (HIV), malaria, and tuberculosis, and they have demonstrated promising potential in cancer immunotherapy **[21]**. While the immunogens themselves are not infectious, they can induce both cellular and humoral immune responses **[22,23]**.

DNA vaccines have been used in vaccine development for SARS-CoV-2-related viruses, such as Middle East Respiratory Syndrome virus and SARS-CoV-1 **[24]**. The DNA vaccines were found to be well tolerated in humans and induced an antibody response in >80% of the participants. Using a similar approach, a SARS-CoV-2 vaccine candidate (ZyCoV-D) has been developed by Zydus Cadila **[25]**. The vaccine was recently approved for emergency use in India, based on a 28,000-person trial **[25]**.

As a platform technology, DNA vaccines have great potential to address priority pathogens during public health emergencies **[26,27]**. Like mRNA vaccines, DNA vaccines can be designed quickly. The gene insert of a DNA vaccine can be rapidly adjusted in response to emerging global health threats, while the manufacturing and control of the new product may remain the same **[23]**. The greatest benefit of DNA vaccines, especially in resource limited settings, is that these are more stable than mRNA vaccines and can even be stored at ambient temperatures **[23]**.

To address vaccine delivery challenges and ensure equitable and rapid access to much needed COVID-19 booster vaccines, Alvea, LLC. Has developed a plasmid DNA booster vaccine, Alveavax-v1.2. The vaccine comprises double-stranded plasmid DNA carrying the gene for the SARS-Cov-2 spike protein containing Omicron/BA.2-specific mutations, as well as two proline mutations to stabilize the prefusion conformation of the protein in a pVAX1 backbone.

7 STUDY OBJECTIVES AND ENDPOINTS

7.1 OBJECTIVES

As this was a first-in-human, Phase 1, dose-finding trial, the primary objective was:

- To evaluate the safety and tolerability of Alveavax-v1.2 in healthy participants, compared with a control booster vaccine (the Janssen Ad26.COV2.S COVID-19 vaccine).

Secondary objectives were:

- To evaluate the immunogenicity as humoral immune response against SARS-CoV-2 BA.2/Omicron after a booster dose of Alveavax-v1.2
- To evaluate the clinical efficacy against SARS-CoV-2 after a booster dose of Alveavax-v1.2
- To evaluate the success rate of ID injections

Exploratory objectives were:

- To evaluate the cell-mediated immune response against SARS-CoV-2 BA.2/Omicron after a booster dose of Alveavax-v1.2
- To evaluate Fc effector functions against SARS-CoV-2 after a booster dose of Alveavax-v1.2
- To evaluate the humoral immune response against additional SARS-CoV-2 variants and sub-lineages after a booster dose of Alveavax-v1.2
- To correlate clinical efficacy with neutralizing antibody response
- To correlate clinical efficacy and immunogenicity with anti-nucleocapsid protein antibodies

7.2 ENDPOINTS

The endpoints for the primary objective of evaluation of safety and tolerability were the following:

1. Number of participants with solicited local and systemic adverse events (AEs) within 7 days after dose administration
2. Number of participants with unsolicited AEs within 28 days of vaccination
3. Number of participants with any serious adverse events (SAEs), adverse events of special interest (AESIs), and AEs leading to withdrawal during the entire period of study

The endpoints for the secondary objectives were as follows:

- The humoral immune response of booster vaccinations against SARS-CoV-2 BA.2/Omicron measured at Baseline (pre-vaccination; except for Point 1 below) and on Day 28, using the following:
    1. Change in geometric mean titer (GMT) of anti-spike protein (S) immunoglobulin G (IgG) antibody
    2. GMT of anti-spike protein (S) IgG antibody
    3. Geometric mean fold rise (GMFR) of anti-spike protein (S) IgG antibody
- Clinical efficacy measured on Day 7, Day 14, Day 28, Day 84, and Day 168, using the WHO clinical progression scale for COVID-19
- The success rate of ID injections as measured by the absolute number and fraction of ID injections that generated a clearly demarcated bleb, of ≥ 1 mm and ≥ 7 mm in diameter, clearly visible for at least 20 seconds, for 0.5 mg and 2 mg Alveavax-v1.2, respectively

Exploratory endpoints were only analyzed based on findings from secondary endpoints and Sponsor's determination, accordingly, and presented in **Table 7.1**.

Table 7.1: Exploratory Analysis Endpoints (Protocol Number: Alvea-VAX-P00001)

Exploratory analysis	Measurement
Cellular immune response of booster vaccinations against SARS-CoV-2 ancestral and BA.2/Omicron<br>– At Baseline (pre-vaccination) and Day 28	1. Spike-specific CD4+ T-cell response<br>2. Change in spike-specific CD4+ T-cell response<br>3. Spike-specific CD8+ T-cell response<br>4. Change in spike-specific CD8+ T-cell response
Fc effector functions against Omicron/BA.2, ancestral variants (such as Alpha, Beta, Delta), and additional Omicron subvariants (such as BA.1)<br>– At Baseline (pre-vaccination; except for Point 1), Day 7, Day 14, Day 28, Day 84, and Day 168	1. Change in relative light units (RLU), and<br>2. RLU of antibody dependent cellular cytotoxicity, antibody dependent cellular phagocytosis, antibody dependent cellular trogocytosis, complement deposition, Fc dimer receptor binding
Humoral immune response of booster vaccinations against ancestral, existing and yet to be defined variants (such as Alpha, Beta, Delta), and existing and yet to be defined sub-lineage (such as Omicron BA.1) SARS-CoV-2 strains<br>– At Baseline (pre-vaccination; except for Point 1), Day 7, Day 14, Day 28, Day 84, and Day 168	1. Change in GMT<br>2. GMT<br>3. GMFR of serum anti-SARS-CoV-2 neutralizing antibodies, serum anti-S IgG antibodies, serum anti-S-RBD IgG antibodies
Correlation of clinical efficacy with neutralizing antibody response<br>– At Day 7, Day 14, Day 28, Day 84, and Day 168	For each neutralizing antibody type:<br>1. Percentage of participants with neutralization IC_{50} >64 IU/mL against the respective strain<br>2. Percentage of participants with neutralization IC_{50} >128 IU/mL against the respective strain

**Exploratory analysis**	**Measurement**
Correlation of clinical efficacy and immunogenicity with anti-nucleocapsid protein antibodies<br>– At Baseline, Day 7, Day 14, Day 28, Day 84, and Day 168	1. Serologic change in GMT and GMFR between baseline and other blood samples for anti-nucleocapsid protein (N) IgG antibodies

8 INVESTIGATIONAL PLAN

8.1 OVERALL STUDY DESIGN AND PLAN

This was a first-in-human, open-label, active-controlled, randomized dose-finding study to evaluate safety, tolerability, and immunogenicity of ID and SC application of the plasmid DNA SARS-CoV-2 Omicron BA.2 vaccine Alveavax-v1.2 in primary Ad26.COV2.S vaccinated healthy adult individuals. The protocol and protocol amendments are provided in **Appendix 15.1.1**, and protocol amendments are summarized in **Table 8.5**. A sample CRF is in **Appendix** 15.1.2.

After signing the informed consent form, primary Ad26.COV2.S vaccinated participants were randomized into one of five treatment arms to receive Alveavax-v1.2 or a Ad26.COV2.S control booster vaccine. The original protocol included an additional treatment group, 2A, consisting of twenty participants, who were unvaccinated and had been infected by COVID-19. This group was removed on request of the Clinical Trials Committee of the South African Health Products Regulatory Authority (SAHPRA), following review. Participants were enrolled at 7 (+/- 3) sites in South Africa within 28 days after the initial screening to ensure they met all inclusion criteria and none of the exclusion criteria.

Each participant was administered a booster vaccine on Day 1 of the study and was monitored afterwards as described below and in **Section 8.4.1**. Participants were given a diary card, thermometer, and a measuring template used to estimate the diameter of local reactions such as erythema or swelling. For up to 7 days (the vaccine administration day and 6 days later), participants monitored temperature daily, and recorded any local pain and the size of swelling or erythema at the injection site. Any other adverse events were also recorded along with severity measured with a scale provided to them. Further details are given in Section 8.1.6 (Diary and Measurement Devices) of the protocol (**Appendix 15.1.1**). AEs (as defined in **Section 10.1**) and concomitant medications (as defined in **Section 8.4.6**) were collected throughout the study. A total of 130 male and female participants aged between 18 and 65 years, inclusive, who satisfied the inclusion and exclusion criteria were enrolled in five groups, and with vaccine administered according to **Table 8.1**.

A multi-arm study design was used to allow for the dose escalation, comparison with the control group, and testing of a different route of administration.

Table 8.1: Dosing Schema (Protocol Number: Alvea-VAX-P00001)

Group	No. of participants*	Dose	Delivery method	Route	Dose administration
1.a: Low dose	20	0.5 mg Alveavax-v1.2	Needle	ID	Single injection
1.b: Standard dose	40	2 mg Alveavax-v1.2	Needle	ID	Single injection
1.c: High dose	20	8 mg Alveavax-v1.2	Needle	ID	Four injections (4x 2 mg)†
1.d: SC injection	10	8 mg Alveavax-v1.2	Needle	SC	Single injection
1.e: Control	40	Janssen Ad26.COV2.S	Needle	IM	Single injection

Sample size determination in **Section 8.7.2**<br>†Four ID injections shall be administered immediately one after the other, preferably at the same anatomic site (e.g., upper arm) with a few centimeters distance between each injection.

Dependent upon safety and/or immunogenicity data generated during this study, it was possible that groups may be started at the next highest dose, groups may not be started, groups may be terminated early, and/or groups may be added with dose levels below the lowest stated dose or intermediate between the lowest and highest stated doses. The details of any such events that occurred during the study are described in **Section 9**.

A schema of the study design is provided in **Figure 8.1**. The enrollment started with the randomization of the first 10 participants into the low dose (1.a) arm and the control (1.e) arm (five participants each); subsequently, at least 24 hours apart, five participants were randomly allocated to the vaccinated standard dose (1.b) arm, followed by a review of 24-hour safety data by the independent medical monitor. Then the remaining participants assigned to the low and standard dose cohorts, in addition to those assigned to the control (1.e) arm, were recruited. In parallel, the first five participants of the high dose (1.c) arm were enrolled. In parallel, the first five participants of the high dose (1.c) arm were enrolled. Further recruitment and the SC injection arm (1.d) started as soon as the independent medical monitor had reviewed 24h safety data of the high dose arm.

For each dose level the following applied:

- Additional safety assessments (see **Section 8.5.1**)
- Controlled enrollment (for new dose levels low / standard and high dose):
    - No more than five participants to be vaccinated on the first day
    - The first five participants to be observed for at least 4 hours after vaccination for any acute reactions
    - Vaccination of the remaining participants (participant 6 and above) to commence no sooner than 24 hours after the fifth participant received his or her vaccination
- Application of stopping rules (see **Section 8.3.4**)
- Escalation between dose levels to be allowed only after independent medical monitor review of at least 24 hours post-dose safety data in this study

Full details of the study design are given in the protocol included in **Appendix 15.1.1**. A sample CRF is in **Appendix 15.1.2**.

Figure 8.1: Study Design Schema (Protocol Number: Alvea-VAX-P00001)

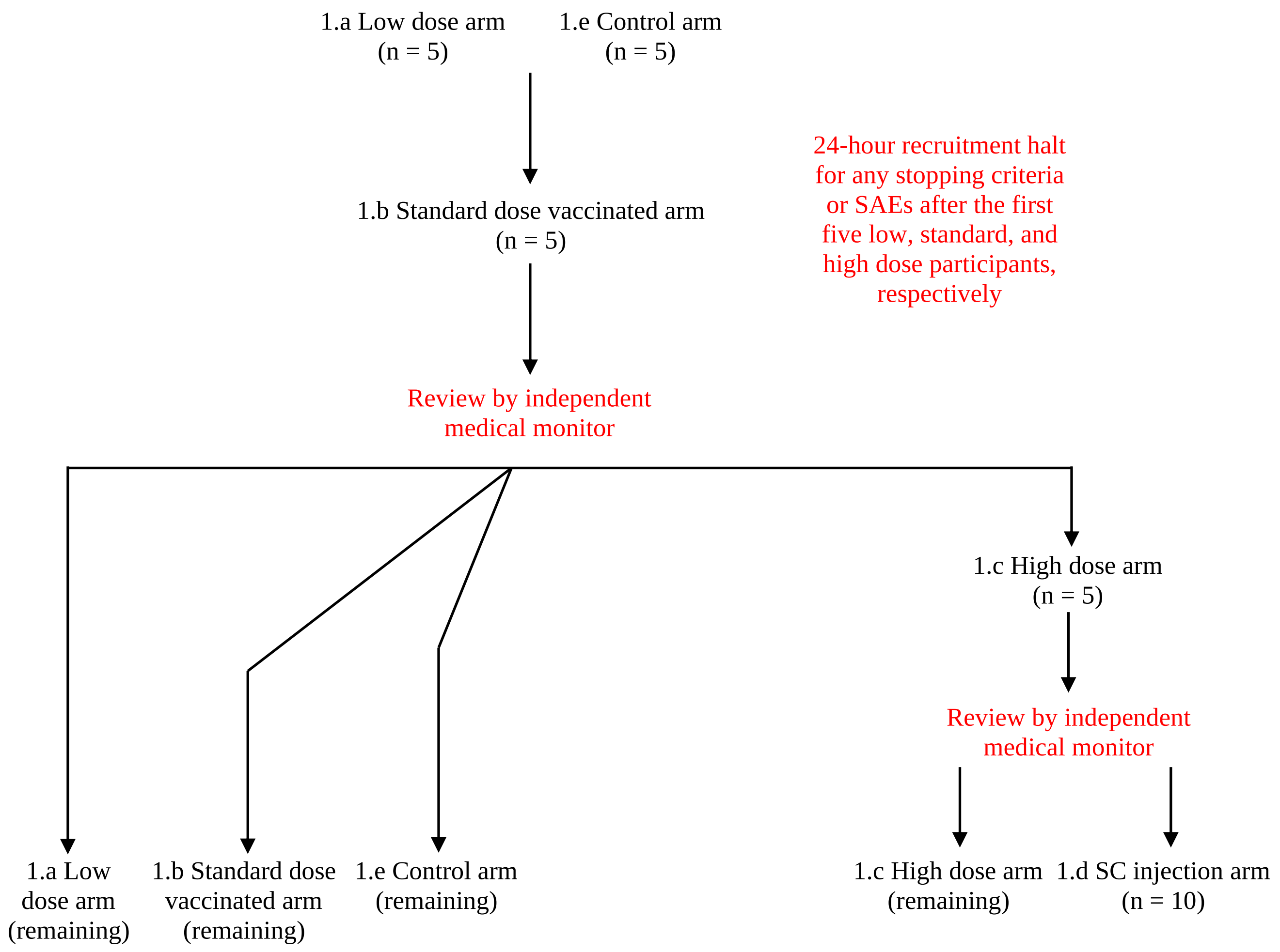

8.2 DISCUSSION OF STUDY DESIGN, INCLUDING THE CHOICE OF CONTROL GROUPS

The current study was the first time Alveavax-v1.2 was administered to humans. Safety and tolerability of chosen doses of Alveavax-v1.2 were supported by preclinical studies.

Previous phase 1, 2 and 3 clinical trials have shown DNA vaccines for SARS-CoV-2 to be safe and well tolerated in humans in a variety of doses and routes of administration **[2,28–31]**.

The selected standard dose of 2 mg of Alveavax-v1.2 was based on trial results of the first emergency authorized DNA vaccine against COVID-19, ZyCoV-D. ZyCoV-D is the closest extensively studied product to Alveavax-v1.2; utilizing the same plasmid backbone, pVAX1, and a similar antigen, SARS-CoV-2 spike protein, and also administered intradermally **[31]**.

Preclinical studies with ZyCoV-D showed that a 2 mg dose demonstrated protective efficacy **[32]**. The Phase 1 study showed that a dose of 2 mg, injected either by needle or with Pharmajet Tropis needle-free device, resulted in significantly higher immunogenicity than a dose of 1 mg in unvaccinated and SARS-CoV-2 naïve healthy individuals **[31]**. No deaths or serious adverse reactions were reported in this study of 48 enrolled participants **[31]**. Subsequently, ZyCoV-D ID primary 3 dose regimen at 2 mg per dose received emergency use authorization in India on the basis of interim Phase 3 study data **[33]**. Supporting this choice, ID administration of a 2 mg dose of DNA (followed by electroporation) was also safe and well tolerated in a Phase 1 trial for a different vaccine against SARS-CoV-2 **[34]**, as well as in previous human Phase 1 trials for Ebola **[35]** and Zika **[36]** vaccines.

As the above studies were conducted in SARS-CoV-2 naïve individuals, the current study additionally tested a 4x lower dose of 0.5 mg to assess whether a lower dose may cause a sufficient immune response in pre-immunized subjects.

DNA vaccines have been administered at higher doses with acceptable safety and tolerability. Human studies for the GX-19 pDNA COVID-19 vaccine showed a 3 mg dose to be safe and well tolerated **[28]**. AnGes DNA vaccine studies against COVID-19 suggest higher doses might be required to elicit a sufficient antibody response **[29]** and subsequently conducted a clinical trial testing single doses of up to 8 mg and total doses up to 16 mg, with no results reported at the time of writing **[30]**. The current study selection for the maximum dose was therefore 8 mg. As ID injections can erroneously be administered subcutaneously, both safety and immunogenicity data for ID and SC injections with 8 mg were collected.

Based on available nonclinical data of Alveavax-v1.2, data from human studies with related DNA vaccines, and taking into account the measures taken to minimize risk to participants participating in this study, the potential risks identified in association with the Alveavax-v1.2 SARS-CoV-2 vaccine are justified by the anticipated benefits that may be afforded to healthy participants (for further evaluation of risk and benefit, refer to **Table 8.2**).

No safety or immunogenicity information in humans is currently available for the Omicron-optimized Alveavax-v1.2 vaccine. Non-clinical safety of Alveavax-v1.2 was confirmed in Good Laboratory Practice (GLP) studies in mice. Clinical studies with other approved/experimental vaccines have not identified any undesirable side effects with the use of DNA vaccines developed for SARS-CoV. Common events associated with vaccines such as injection site pain, tenderness or pruritus and general side effects such as pyrexia, arthralgia, and diarrhea following vaccination were each seen in <10% of participants **[31]**.

Human reproductive safety data are not available for Alveavax-v1.2, but there was no suspicion of human teratogenicity based on the intended mechanism of action of the compound. Nevertheless, the use of a highly effective method of contraception was required (refer to Appendix 1 [Contraception] of the protocol in **Appendix 15.1.1**) and pregnant and breastfeeding women were excluded from the trial.

8.2.1 Risk/Benefit Assessment

The Omicron variant demonstrates immune escape and increased transmissibility in previously vaccinated or infected individuals. There are no licensed human SARS-CoV-2 vaccines available optimized for the Omicron variant currently.

As described above, only non-clinical safety of Alveavax-v1.2 has been confirmed in GLP studies in mice, and other clinical studies of DNA vaccines developed for SARS-CoV saw typically common side effects (e.g., one of pyrexia, arthralgia, or diarrhea, individually) in <10% of participants following vaccination **[31]**.

All mild to severe AEs were monitored during this study. Measures were in place to monitor participants. Safety was ensured through the review and appraisal of clinical data, laboratory profile and risk management documentation. AEs after vaccination were expected to be mild and manageable using routine symptom-driven standard of care as determined by the investigators.

8.2.1.1 Risk Assessment

Table 8.2: Evaluation of Risk (Protocol Number: Alvea-VAX-P00001)

Potential Risk of Clinical Significance	Summary of Data/Rationale for Risk	Mitigation Strategy
Potential for local reactions (injection site redness, injection site swelling, and injection site pain) and systemic events (fever, fatigue, headache, chills, vomiting, diarrhea, muscle pain, and joint pain) following vaccination.	These are common adverse reactions seen with other vaccines, as noted in the FDA Center for Biologics Evaluation and Research (CBER) guidelines on toxicity grading scales for healthy adult volunteers enrolled in preventive vaccine clinical trials.	The study design includes the use of controlled vaccination to closely monitor and ensure participant safety. A reactogenicity diary will be used to monitor local reactions and systemic events. Stopping rules are in place. The first 5 participants treated who received a low, standard, and high dose will be observed for 4 hours after vaccination and all other participants will be observed for 30 minutes after vaccination to assess any immediate AEs.
Unknown AEs and laboratory abnormalities with a novel vaccine.	Studying vaccine candidates in humans can uncover previously unknown or unexpected AEs. The safety of plasmid DNA delivery has been extensively validated and the WHO recognizes that "To	The study design includes the use of controlled vaccination to closely monitor and ensure participant safety. An independent medical Monitor (IMM) will also review safety data. Stopping rules

	date, published data from clinical trials indicate that DNA vaccines are safe and have acceptable reactogenicity profiles." **[22,23]**	are in place. The first 5 participants treated who received a low, standard, and high dose will be observed for 4 hours after vaccination and all other participants will be observed for 30 minutes after vaccination to assess any immediate AEs.
Participants will be required to attend healthcare facilities during the global SARS-CoV-2 pandemic.	Without appropriate social distancing and PPE, there is a potential for increased exposure to SARS-CoV-2.	The Sponsor will work with sites to ensure an appropriate COVID-19 prevention strategy.
Venipuncture, ID, SC, and IM injections will be performed during the study.	There is the risk of bleeding, bruising, hematoma formation, and infection at the injection site, and pain associated with the injection(s).	Only appropriately qualified personnel will obtain the blood draw, and perform ID, SC, and IM injection.

8.2.1.2 Benefit Assessment

Benefits to individual participants may include:

- Receipt of a potentially efficacious Omicron-optimized COVID-19 booster vaccine during a global pandemic and Omicron wave
- Access to extensive diagnostic testing and medical check-ups
- Early access to vaccine if efficacious and approved
- Contributing to research to help others during a global pandemic

8.2.1.3 Overall Benefit/Risk Conclusion

Based on available nonclinical data of Alveavax-v1.2, data from human studies with related DNA vaccines, and considering the measures taken to minimize risk to participants participating in this study, the potential risks identified in association with the Alveavax-v1.2 SARS-CoV-2 vaccine were justified by the anticipated benefits that may be afforded to healthy participants.

8.3 SELECTION OF STUDY POPULATION

Healthy adult individuals, previously having received a primary Ad26.COV2.S vaccination series against SARS-CoV-2, satisfying all the eligibility criteria were eligible to participate in the study. Screening for eligible participants was performed within 28 days of vaccination in the study.

The inclusion and exclusion criteria for enrolling participants in this study are described below. If there was a question about the inclusion or exclusion criteria, the Investigator was to consult with the appropriate Sponsor representative and resolve any issues before enrolling a participant in the study. Waivers were not allowed, unless otherwise specified in the inclusion / exclusion criteria.

Potential participants must have been willing and able to adhere to the following lifestyle restrictions during the course of the study to be eligible for participation:

1. Refer to protocol section 6.7 (Concomitant and Prohibited Therapies) for details regarding prohibited and restricted therapy during the study.
2. Agree to follow all requirements that must be met during the study as noted in the inclusion and exclusion criteria.

Screening for eligible participants was performed within 28 days of vaccination in the study. A participant was considered to be a screen failure if the participant signed the informed consent form but was ineligible at the screening visit or withdrew before receiving trial medication. The Investigators were to account for all participants who signed an informed consent form for the study.

All potential participants who were screened for enrollment in this study, including screening failures, were listed on the participant screening list. The primary reason for screen failure was recorded in the electronic case report form (eCRF). Participant identification numbers assigned to participants who failed screening were not reused.

The Investigators were responsible for all participants who signed an informed consent form for the study. If a participant was found not to be eligible to participate in the study at the screening visit, appropriate eCRFs were completed by the Investigators. The eCRF included the primary reason for the screen failure. Identification numbers assigned to ineligible participants were not reused.

8.3.1 Inclusion Criteria

Participants were required to satisfy all of the following criteria to be enrolled in the study:

1. Healthy adult male and female volunteers between 18 and 65 years of age, inclusive.
2. Participants who received a primary Janssen Ad26.COV2.S COVID-19 vaccine ≥ 60 days prior to receiving the study vaccine (Day 1) in this study.
3. Body mass index (BMI) within the range 18–32 kg/m^2 (both inclusive).
4. Participants who, judged by the Investigator, are in stable health as determined by their pre-study medical history, physical examination, and clinical laboratory tests.
5. Female participants must be either of non-childbearing potential, i.e., surgically sterilized (defined as having undergone hysterectomy and/or bilateral oophorectomy and/or bilateral salpingectomy; tubal ligation alone not considered sufficient) or one year postmenopausal; or, if of childbearing potential, they must be abstinent or have used adequate contraceptive precautions (refer to Appendix 1 [Contraception] of the protocol in **Appendix 15.1.1**) for 30 days prior to receiving the study vaccination and 84 days post-vaccine.
6. Sexually active male participants who are considered sexually fertile must agree to use a barrier method of contraception during sexual activity with a female of childbearing potential from the time of vaccination until at least 84 days after the vaccination.
7. Participants must provide written informed consent, or their legal representative must understand and give written consent to the procedure.

8. Participants must be willing and able to comply with all the required study visits and follow-up required by this protocol, and be able to complete the diary card after vaccination or have a caregiver available to assist with these matters.

8.3.2 Exclusion Criteria

Any potential participant who met any of the following criteria were excluded from participating in the study:

1. Received any other SARS-CoV-2 vaccination than a single Janssen Ad26.COV2.S COVID-19 vaccine or plans to receive any additional SARS-CoV-2 vaccination within 90 days after the study vaccine (Day 1).
2. Recovered from SARS-CoV-2 infection determined by history of a positive SARS-CoV-2 test (e.g., PCR, rapid antigen test, etc.) or suspicion of a SARS-CoV-2 infection based on the (verbal) medical history within less than 60 days from the day of vaccination (Day 1) in this study.
3. History of close contact (face-to-face contact within 1 meter or contact in a closed space for more than 15 minutes) without wearing a face-mask with a confirmed active SARS-CoV-2-positive patient within 5 days prior to Day 1.
4. Have received any live-virus vaccine within 4 weeks or inactivated vaccine, including influenza vaccine, within 2 weeks (both licensed and investigational vaccines) prior to the study vaccine (Day 1).
5. Previous participation in any clinical trial of a SARS-CoV-2 vaccine candidate.
6. Have any febrile illness (temperature ≥ 38°C/100.4°F) or any active acute illness or infection (including a positive SARS-CoV-2 PCR test) within 7 days prior to administration of vaccination (Day 1) in this study. Participants may be re-evaluated once all symptoms have resolved.
7. History of severe adverse reaction associated with a vaccine and/or severe allergic reaction (e.g., anaphylaxis) or contraindications to any component of the study intervention(s).
8. History of, or positive screening test for HIV I or II.
9. Any clinically significant finding during screening or check-in that, in the Investigator’s judgment, results in an increased safety risk.
10. History of cerebral venous sinus thrombosis, antiphospholipid syndrome, or a history of heparin-induced thrombocytopenia and thrombosis (HITT or HIT type 2).
11. Any confirmed or suspected immunosuppressive or immunodeficient state; asplenia; recurrent severe infections and use of immunosuppressant medication within the past 3 months, except topical and inhaled steroids, or short-term oral steroids (course lasting ≤ 14 days or ≤ 20 mg/day).
12. History of receiving blood transfusion, blood products, immunoglobulin, or immune stimulants within 3 months prior to Day 1.

13. Is currently participating in any other study or has received any investigational drug in the last 6 weeks or 5× the half-life of the drug (whichever is longer) prior to screening.
14. For female participants of childbearing potential who are pregnant (positive pregnancy test at the screening or check-in), currently breastfeeding, or attempting to conceive.
15. Any addiction that may interfere with the participant’s ability to comply with trial procedures.
16. Inability to be venipunctured or tolerate venous, IM, SC, or ID puncture.
17. Have a rash, dermatological condition, tattoo, or any other abnormality at the injection site that may interfere with injection site reaction rating. Investigator discretion was permitted with this exclusion criterion.
18. Use of prophylactic medications (e.g., antihistamines [H1 receptor antagonists], nonsteroidal anti-inflammatory drugs [NSAIDs], systemic glucocorticoids, non-opioid, and opioid analgesics) within 24 hours prior to the vaccination to prevent or pre-empt symptoms due to vaccination.
19. Any condition or abnormal baseline findings or any other unspecified reason, which in the Investigator’s judgment might increase the risk to the participant or decrease the chance of obtaining satisfactory data needed to achieve the objective of the study.
20. Participants identified as an Investigator or employee of the Investigator or clinical site with direct involvement in the proposed study, or identified as an immediate family member (i.e., parent, spouse, natural or adopted child) of the Investigator, or employee with direct involvement in the proposed study, or any employees of the Sponsor company.

8.3.3 Removal of Participants from Therapy or Assessment

Participants were able to voluntarily withdraw from the study for any reason at any time if they withdrew consent to participate. They were not required to state their reasons for withdrawing their consent. A participant may have been discontinued from the study if there was a serious or intolerable AE, or if it would not be in the participant’s best interest to continue further in the study according to the Investigator.

The Investigator may have withdrawn a participant from the study if they met any of the following withdrawal criteria:

- The participant wished to withdraw their consent for participation
- Severe (≥ Grade 3) AE or laboratory abnormality related to vaccines
- The participant suffered from significant intercurrent illness or underwent major surgical intervention during the study
- Lost to follow-up
- Administration of any vaccine or prohibited therapies within 28 days of administration of the study vaccine

- Participant in non-compliance to protocol (including violation of enrollment criteria)
- When it was not in the participant's best interest to continue, in the Investigator's opinion

The date and the reason the participant was withdrawn from the study was documented in the eCRF. Participants who were withdrawn because of AEs were clearly distinguished from participants who were withdrawn for other reasons. Withdrawn participants who had been administered the study vaccine were considered for safety analysis.

If randomized participant were withdrawn from vaccination before the study vaccine was administered, additional participants may have been recruited to replace these participants at the discretion of the Sponsor. Any replacement participant was assigned to the same group as the original (discontinued) participant. If randomized participant were withdrawn after the study vaccine was administered, they were not replaced.

A participant was considered lost to follow-up if he or she repeatedly failed to return for scheduled visits and was unable to be contacted by the study site. A participant could not be deemed lost to follow-up until all reasonable efforts made by the study-site personnel to contact the participant were deemed futile. The following actions were taken if a participant failed to return to the study site for a required study visit:

Every attempt was made to contact study participants who were lost to follow-up. Sites attempted to collect and save a secondary contact number and email for each participant. At least four attempts at contact were made and recorded in the source documents on three distinct days by phone, text message and email [if available]. As a last resort, any participant with whom clinic staff no longer had contact with was notified of a request to be contacted either by registered letter or, failing that, by such other means as the site determined as the most reliable method. All attempts at contact were documented in the participant's source documents.

If a study site closed, e.g., for operational, financial, or other reasons, and the Investigator could not reach the participant to inform them, their contact information was transferred to another study site.

8.3.4 Stopping or Suspending the Study

The Sponsor, the Investigator (following consultation with the Sponsor), SAHPRA, or IEC/IRB had the right to discontinue this study at any time after appropriate consultation among the involved parties. The Sponsor had the right to suspend or discontinue their investigational product development at any time.

Conditions that may warrant termination of the study included but were not limited to the following:

- The discovery of unexpected and relevant conditions or events that suggested a possible hazard or unacceptable risk to the participants enrolled in the clinical study.
- Early definitive results/data proving no effect

If the study was terminated and/or the site closed for any reason previously listed or for other factors, copies of study and study product documentation were returned to the Sponsor. The study site kept an archive of site-specific documents.

Throughout the study period, if any SAE or serious complaint occurred, Investigators were obligated to review the participant's data immediately and make a medical judgment of whether the SAE or serious complaint was vaccine related or not. If any of the following situations occurred, the Principal Investigators informed the Sponsor / Contract Research Organization, and study recruitment was suspended until further notice.

The AE review was as per FDA guidance, Toxicity Grading Scale for Healthy Adult and Adolescent Volunteers Enrolled in Preventive Vaccine Clinical Trials (refer to Appendix 3 [Toxicity Grading Scale] of the protocol in **Appendix 15.1.1**).

In the event of such an occurrence, the Sponsor reserved the right to halt and review this study, and discuss it with the Investigators (including the reasons for taking such action).

- Vaccination was placed on hold if (refer to Section 8.5.4 [Evaluating Adverse Events] of the protocol in **Appendix 15.1.1**)
    - Two or more participants reported the same or similar severe (Grade 4) AE after vaccination **possibly related** to the study investigational product by the Investigator, or
    - Any participant vaccinated developed a severe (Grade 4) AE event after vaccination **probably or definitely related** to the study investigational product by the Investigator.
- Opinions of IEC and IMM were collected before enrolling further participants.

8.4 TREATMENT

8.4.1 Treatments Administered

The study evaluated a single-dose schedule of multiple dose levels and application variations of an investigational plasmid DNA booster vaccine candidate (Alveavax-v1.2) compared with a COVID-19 booster vaccine as control for active immunization optimized for COVID-19 Omicron.

The investigational plasmid DNA booster vaccine candidate and the control vaccine were the potential study interventions that were administered to a study participant (see **Table 8.3**):

- Alveavax-v1.2: 0.5 mg, 2 mg, or 8 mg
- Janssen Ad26.COV2.S COVID-19 vaccine: 0.5 mL

Table 8.3: Study intervention (s) administered (Protocol Number: Alvea-VAX-P00001)

Intervention Name	Alveavax-v1.2	Janssen Ad26.COV2.S
Type	Vaccine	Vaccine
Vaccine Type	Plasmid DNA	Adenovirus vector
Unit Dose Strength	5 mg / 1 mL	8.92 log_{10} infectious units / 0.5 mL
Dose Level	0.5 mg, 2 mg, or 8 mg	8.92 log_{10} infectious units
Route(s) of Administration	ID and SC injection	IM injection
Use	Experimental	Control
Investigational or Non-Investigational Medicinal Product	Investigational Medicinal Product	Non-Investigational Medicinal Product
Sourcing	Provided centrally by the Sponsor	Provided centrally by the Sponsor
Packaging and Labeling	Study intervention provided in a glass vial as open-label supply. Each vial labeled as required per country requirement	2.5 mL suspension in a multi-dose vial (type I glass) with a rubber stopper, aluminum crimp and blue plastic cap.

8.4.2 Identity of Investigational Product(s)

Alveavax-v1.2 (Alvea, LLC.) is a preservative-free, sterile formulation of plasmid DNA in an isotonic phosphate-buffered saline. The plasmid DNA substance is the only active substance in this product. The product is a concentrate for injection and filled at 5 ± 0.5 mg/mL. Isotonic phosphate-buffered saline solution was sourced as an approved medicinal product.

The investigational product was provided as a solution for injection. No further dilution was required. Each vial contained two standard doses. Detailed instructions for storage and handling were provided in the respective trial-specific Pharmacy Manuals.

The composition of the investigational product and the functions of the respective components are given in Appendix 4 (Composition of investigational product) of the protocol (**Appendix 15.1.1**). More detailed information about the study drug can be found in the Alveavax-v1.2 Investigator's Brochure.

Dose levels were 0.5 mg, 2 mg, or 8 mg.

Routes of administration were ID or SC injections given to the outer surface of the upper arm (or front of the thigh, or lower back).

The active control vaccine for the study, Janssen Ad26.COV2.S (Johnson & Johnson), is a COVID-19 booster vaccine authorized for emergency use / approved. It is available as a single booster dose for individuals who have completed the primary series of vaccinations. This booster vaccine has been demonstrated to be effective against Omicron and was available for use in this study.

A single booster dose of the control vaccine was administered via IM injection in dosages as per the prescribing information.

Investigational Product batch numbers can be found in **Appendix 15.1.6**.

8.4.3 Avoidance of Bias

8.4.3.1 Method of assigning participants to treatment groups

Following the enrollment and monitoring of the first five participants in each of the 1.a low dose arm, 1.e control arm and 1.b standard dose arm, the remaining participants were randomly assigned into one of these arms.

Following the enrollment and monitoring of the first five participants in the 1.c high dose arm, the remaining participants in the high dose and SC injection arm were randomly assigned into one of these arms.

Randomization was performed centrally by Clario (Bioclinica IRT), who generated the sequence and provided the mechanism for treatment allocation. All details of the randomization process can be found in the "Kit and Randomization Specification (KARS)" document [Alvea-VAX-P0001_Kit Rand Spec_(KARS)_v2.0.pdf] in **Appendix 15.1.7** for further details.

8.4.3.2 Blinding and unblinding

This was a randomized, open-label study, as described in the protocol (in **Appendix 15.1.1**).

8.4.4 Selection of Dose(s) and Timing of Each Dose for Each Participant

The participants received a total of up to four injections, either one to four ID, one SC or one IM via needle as per **Table 8.1** at ambient temperature.

The ID and SC injections were given to the outer surface of the upper arm (or front of the thigh, or lower back). The IM injections were handled and administered as per the protocol for the control booster vaccine.

Administration of study interventions were performed by an appropriately qualified, GCP-trained, and vaccine-experienced member of the study staff (e.g., physician, nurse, physician's assistant, nurse practitioner, pharmacist, or medical assistant) as allowed by local, state, and institutional guidance.

See **Section 8.1** (Overall Study Design and Plan) and **Table 8.1** (Dosing Schema).

8.4.5 Treatment Compliance

Study vaccines were administered by unblinded qualified study site personnel at the study site according to the Pharmacy Manual. Appropriate personnel were adequately trained to ensure proper ID administration of the product, prior to injection into participants.

Every injection was checked by the study person who administered the vaccine immediately after administration to confirm that the syringe was completely empty. Additionally, for ID injections, to confirm that a clearly demarcated bleb ≥ 1 mm and ≥ 7 mm in diameter was generated and clearly visible for at least 20 seconds, for 0.5 mg and 2 mg Alveavax-v1.2, respectively. The anatomical site of the bleb, the bleb size and if the bleb remained for at least 20 seconds was documented in the eCRF (including date and time of injection and location used for IM, SC, or ID injection).

8.4.6 Prior and Concomitant Therapy

Concomitant medication given to, or taken by, the participant during the study was clearly documented on the eCRF, as follows:

- From 1 year to 30 days before the vaccination all immunoglobulin, immune stimulants, and vaccines received.
- From 30 days before the vaccination (Day 1) to study Day 28 (Visit 5) – all prescription, non-prescription, or over-the-counter medications (such as aspirin or antacids), vitamins, mineral supplements, and herbal remedies; all medication used to treat conditions reported as medical history; and medication used to treat AEs (solicited AEs, unsolicited AEs, AESI, SAE, or AE leading to withdrawal).
- From Day 29 to the end of the study, inclusive: any concomitant medication(s) administered to treat AEs: on-going medications for AE that occurred before Day 28; and medication for AESI, SAE, or AE leading to withdrawal.

Information about the medication's generic name / trade name (or names, if combination medication), indication, total daily dose, route of administration, and start and end dates of treatment were entered in the eCRF.

A note was made of whether the medication being taken was prophylactic. A prophylactic medication was defined as a medication administered in the absence of any symptom and in anticipation of a reaction to treatment. Concomitant medication administered for the treatment of an AE or SAE was recorded on the Concomitant Medication Log of the CRF and SAE Report.

Medications considered necessary for the participants, and which were not known to interfere with the study vaccine, were allowed at the discretion of the Investigator, and appropriate records of the same were kept in the CRF. Furthermore, the concomitant use of medications for the treatment of other concomitant diseases not known to interact with the study vaccine were permitted as deemed necessary by the Investigator. A list of all the concomitant medications consumed by the participant were recorded in the CRF.

Use of the following medications and therapies within the specified time was not permitted:

1. SARS-CoV-2 vaccination 90 days after Day 1.
2. Any live-virus vaccine (both licensed and investigational) within 4 weeks prior to and 4 weeks after Day 1.
3. Any inactivated vaccine (both licensed and investigational), including influenza vaccine, within 2 weeks prior to and 2 weeks after Day 1.
4. Any blood products, including immunoglobulin, or immune stimulants within 3 months prior to Day 1.
5. Immunosuppressant or other immune modifying drugs within 3 months prior to Day 1.
6. Use of prophylactic medications (e.g., antihistamines [H1 receptor antagonists], antipyretics, nonsteroidal anti-inflammatory drugs [NSAIDs], systemic and topical glucocorticoids, non-opioid, and opioid analgesics) within 24 hours prior to the vaccination to prevent or preempt symptoms due to vaccination.

Depending on the time of the occurrence, any participant who received a prohibited concomitant medication was not included in the immunogenicity analyses.

The following medications could lead to drug interactions with Alveavax-v1.2 vaccine and influence immunogenicity results:

1. Immunoglobulins, blood, or blood products:
    a. These can affect study evaluations related to immunogenicity data for the vaccine.
2. Immunostimulant agents:
    a. These could change the immunological response of the vaccine.
3. Immunosuppressive agents:
    a. These may result in the participant not developing optimum immunologic responses to the vaccine.
4. Antiviral medications:
    a. These could change the immunological response of the vaccine.
5. Anti-coagulants:
    a. These could put the participant at increased risk of formation of injection site hematoma.

8.5 SAFETY, IMMUNOGENICITY AND EFFICACY VARIABLES

8.5.1 Safety, Immunogenicity and Efficacy Assessments and Schedule of Assessments

A schema of the study design is presented in **Figure 8.1** and the schedule of assessments is shown in **Table 8.4**. Information on the blood collection at study visit is listed in **Table 8.4**.

Table 8.4: Schedule of Assessments (Protocol Number Alvea-VAX-P00001)

**No.**	**Assessment**	**Screening**	**Enrollment and vaccine administration**		**Follow-up Period**			
		**Day -28 to Day -1**	**Check-In[G]**	**Dosing (Day 1)**	**Day 3 (call)[A]**	**Day 7 ± 2 d**	**Day 14 ±2 d, Day 28 ±2 d, Day 84 ±7 d**	**Day 168 ±14 d (End-of-Study)**
	**VISIT #**	**V-1**	**V1**		**V2**	**V3**	**V4, 5, 6**	**V7**
1.	Informed consent process	X						
2.	Inclusion / Exclusion criteria	X	X					
3.	Demographics (age, sex, height, and weight)	X						X (weight)
4.	Medical history	X	X[F]					X
5.	Provision of emergency contact card, thermometer, and measuring device/ruler		X					
6.	Randomization			X				
Safety Assessments								
7.	Physical examination[E]	X	X			X		X
8.	Vital signs	X	X	X[D]		X		X
9.	AE assessment	X	X	X	X	X	X	X
10.	COVID-19 assessment					X	X	X
Laboratory Assessments								
11.	Hematology	X	X[C]			X		
12.	Chemistry	X	X[C]			X		
13.	Urine pregnancy test		X[B]				X	
14.	Serology	X						

No.	Assessment	Screening	Enrollment and vaccine administration		Follow-up Period			
		Day -28 to Day -1	Check-In[G]	Dosing (Day 1)	Day 3 (call)[A]	Day 7 ± 2 d	Day 14 ±2 d, Day 28 ±2 d, Day 84 ±7 d	Day 168 ±14 d (End-of-Study)
	VISIT #	V-1	V1		V2	V3	V4, 5, 6	V7
15.	Blood sample for immunology		X			X	X	X
16.	Nasopharyngeal swab sample for PCR for SARS-CoV-2		X			X	X[H]	
Other Procedures								
17.	Vaccination			X				
18.	Participant diary card issue and / or review			X		X		
19.	Inquire pregnancy[I], COVID-19 infections and vaccinations						X	X

A. Telephonic safety follow-up performed on Day 3.

B. Urine pregnancy test performed for female participants of childbearing potential on screening, pre-dose, on Day 28, and on Day 84.

C. Clinical lab investigations performed if more than 7 days have passed since the screening.

D. Vitals (including blood pressure, pulse rate, and temperature) performed at pre-dose and 1, 2, 3, and 4 hours (±30 minutes) post-dose for the first 5 participants of each dose level; for all other participants at pre-dose and 30 minutes post-dose.

E. A history-directed physical examination done at baseline, followed by an abbreviated, symptom-directed physical examination at subsequent time points.

F. Medical history was updated from the screening visit.

G. Check-in was within 48 hours prior to dosing.

H. Nasopharyngeal (NP) swab sample on Day 14 and 28 only.

I. Confirmed with female participants of childbearing potential and male participants who had a female partner if they/their partner had become pregnant since Day 1 of the study.

8.5.1.1 Primary (Safety) assessments

The investigator evaluated safety by AEs monitoring, vital signs, physical examination, clinical laboratory tests and concomitant medications.

Participants were assessed for the occurrence of AEs throughout the study but with special emphasis on the first seven days following vaccination. AEs specifically related to vaccination will be documented by the Investigator. All other AEs reported by the

participants were also recorded. All AEs encountered during the study were reported in the eCRF. If possible, a diagnosis was to be documented rather than signs and symptoms.

Safety assessments included the following:

- Solicited local and systemic AEs within 7 days after dose administration
- Unsolicited AEs within 28 days of vaccination
- Any SAEs, AESIs, and AEs leading to withdrawal during the entire period of study

An AE was defined as any undesired medical occurrence in a patient or clinical investigation patient receiving a pharmaceutical product, which does not necessarily have a causal relationship with this treatment. An AE can therefore be any unfavorable sign and unintended sign (including an abnormal laboratory finding), symptom, or disease temporarily associated with the use of a vaccine, whether related to the vaccine or not. This definition includes intercurrent illnesses or injuries and exacerbation of pre-existing conditions.

Planned hospital admissions and/or surgical operations pre-scheduled prior to the participation in this study for a pre-existing illness or disease from before the vaccine performed during the participation of this study are not to be considered as AEs.

During the first seven days (day of vaccination and next 6 days) following vaccination, the participants were to record in their diary cards the occurrence and severity of the following signs and symptoms:

Local (at injection site)

- Pain
- Erythema (redness; surface diameter in mm)
- Swelling (surface diameter in mm)
- Induration (surface diameter in mm)
- Ecchymosis (bruising; surface diameter in mm)
- Tenderness
- Itching

Systemic

- Fever (≥ 38.0°C or ≥100.40°F; tympanic or oral temperature)
- Headache
- Chills/shivering
- Fatigue
- Nausea
- Vomiting

- Diarrhea
- Malaise
- Myalgia (muscle pain)
- Arthralgia (joint pain)

8.5.1.1.1 Safety – clinical laboratory evaluation

The safety laboratory testing was performed at the local laboratory following the laboratory guidelines. Copies of laboratory accreditation certificates and reference ranges were provided to the Sponsor prior to the analysis of the first participant sample. Blood samples for the laboratory tests for hematology and biochemistry were collected as per **Table 8.4** (Schedule of Assessments). The volume of blood to be collected at each visit was described in Appendix 2 (Blood collection) of the protocol (**Appendix 15.1.1**). Lab results were directly imported via electronic data transfer into the main database. The following laboratory evaluations were to be performed during the study, according to the local laboratory instructions:

- Hematology: hemoglobin, red blood cell (RBC), white blood cell (WBC) with differential count and platelet count
- Biochemistry: total bilirubin, creatinine, blood urea nitrogen (BUN), alanine transaminase (ALT), aspartate aminotransferase (AST)
- Serology: Blood samples were taken at the screening visit only for determination of active infections for hepatitis B virus, hepatitis C virus, and HIV antibody testing
- Urine pregnancy test (women of childbearing potential only): beta-human chorionic gonadotropin

Pregnancy tests may have been urine or serum tests and must have had a sensitivity of at least 25 mIU/mL. Pregnancy tests were performed in women of childbearing potential at the times listed in the Schedule of Assessments (**Table 8.4**). A negative pregnancy test result was required prior to the participant's receiving the study intervention. Pregnancy tests may have also been repeated, if requested by IECs/IRBs or if required by local regulations. In the case of a positive confirmed pregnancy before the study vaccination, the participant was to be promptly withdrawn from the study.

Abnormal laboratory values or changes were not reported as AEs if they were not clinically significant, and they were only recorded as AEs if a therapeutic action was needed or judged by the Investigators.

8.5.1.1.2 Safety – vital sign measurements

Determination of vital signs was conducted by the Investigator or a designated medically-trained clinician. Vital signs (including systolic/diastolic blood pressure in sitting position preceded by at least 5 minutes of rest in a quiet setting without distractions [e.g., television,

cell phones], pulse rate, and body temperature [tympanic measurement preferred, or in accordance with the local standard of care]) were performed according to **Table 8.4** (Schedule of Assessments). Participants with fever defined as tympanic temperature ≥ 38.0°C (100.4°F) should not have received a study vaccine.

Body weight was recorded in kilograms (kg) to 1 decimal place (lightly clothed without coat and footwear), and body height (without footwear) was measured in centimeters (cm) without decimal places at the screening visit.

8.5.1.1.3 Safety – physical examination

A general physical examination was conducted by the Investigator or a designated medically-trained clinician. A history-directed general physical examination was done at screening and prior to administration of vaccination at Visit 1. An abbreviated, symptom-directed examination based on any clinically relevant issues or symptoms, and medical history was to be done at all other subsequent time points. Any abnormality or change in severity found was judged as clinically significant or non-significant by the Investigator and documented in the eCRF.

8.5.1.2 Secondary (Immunogenicity and Efficacy) assessments

Immunogenicity assessments included the following:

- BA.2 anti-spike protein (S) IgG antibody titer

Efficacy assessments included the following:

- WHO clinical progression scale for COVID-19
- Measurement of clearly demarcated bleb, measuring ≥ 1 mm and ≥ 7 mm in diameter, clearly visible for at least 20 seconds, for 0.5 mg and 2 mg Alveavax-v1.2 respectively
- Baseline and other blood anti-nucleocapsid protein (N) IgG antibodies titers

8.5.1.3 Exploratory analysis measurements

Planned exploratory analysis assessments included the following:

Cellular immune response

- serum anti-SARS-CoV-2 BA.2 neutralizing antibody titer
- Spike-specific CD4+ T-cell response
- Spike-specific CD8+ T-cell response
- RLU of antibody dependent cellular cytotoxicity, antibody dependent cellular phagocytosis, antibody dependent cellular trogocytosis, complement deposition, Fc dimer receptor binding
- serum anti-SARS-CoV-2 neutralizing antibodies
- serum anti-S IgG antibodies
- serum anti-S-RBD IgG antibodies
- BA.2 spike-specific CD4+ T-cell response
- BA.2 spike-specific CD8+ T-cell response

- Activity against Omicron/BA.2, ancestral variants (such as Alpha, Beta, Delta), and additional Omicron Subvariants (such as BA.1), measured as RLU of antibody dependent cellular cytotoxicity, antibody dependent cellular phagocytosis, antibody dependent cellular trogocytosis, complement deposition, and Fc dimer receptor binding
- Humoral immune response of booster vaccinations against ancestral, existing, and yet to be defined variants (such as Alpha, Beta, Delta), and existing and yet to be defined sub-lineage (such as Omicron BA.1) SARS-CoV-2 strains, measured as antibody titers of serum anti-SARS-CoV-2 neutralizing antibodies, serum anti-S IgG, and serum anti-S-RBD IgG

8.6 DATA QUALITY ASSURANCE

The CRO and each site were responsible for ensuring that all processes used to promote outcome data quality, during and after data collection, were followed. All study procedures and all processes used to enhance outcome data quality were performed according to the protocol (**Appendix 15.1.1**) and the CRO responsibilities were conducted according to methodologies described in the project-specific procedures manuals.

During the study, the unblinded monitor visited the site regularly to check the completeness of participant records, the accuracy of entries in the CRFs, the adherence to the protocol and to GCP, and the progress of enrollment. The unblinded monitor ensured that the study medication was being stored, dispensed, and accounted for according to specifications. Key study site personnel had to be available to assist the unblinded monitors during these visits.

Subsequently, information entered directly into the study eCRF database was systematically checked by data management personnel, using system generated error messages and system generated line listings. All entered information were updated by site personnel under the instruction of the CRO data management personnel and medical monitors. The CRO data management personnel and medical monitors entered data queries directly into the electronic data capture system. All responses and resolutions were captured in the eCRF. Periodic quality control checks were performed on all raised issues and responses to ensure they were closed appropriately.

All participant forms were locked once the Investigator signatures had been obtained. Participant casebooks reports were created for each site and issued to site. The database was locked to all study personnel (except for administrator and read-only roles) upon receipt of the last confirmation of data receipt from the sites.

All personnel had the same eCRF completion guidelines to work from. Role-specific training was completed before sites could process data in the production eCRF.

This clinical study report has been subjected to a quality control review.

8.7 STATISTICAL ANALYSIS METHODS PLANNED IN THE PROTOCOL AND DETERMINATION OF SAMPLE SIZE

8.7.1 Statistical Plans

The statistical analysis plan (SAP) included details of the immunogenicity and safety analysis. The final SAP is provided in **Appendix 15.1.9** on Documentation of statistical methods. The SAP was finalized prior to the first analysis of study data. Statistical analysis was performed using SAS® software (version 9.4 or higher; SAS Institute Inc., USA).

A detailed data management plan was available for the study.

Since the study was a Phase 1 study primarily assessing safety, all data were analyzed descriptively without a formal statistical hypothesis.

8.7.1.1 General approaches

The processes used to promote outcome data quality during and after data collection can be found in **Section 8.6** above, and in the study protocol (**Appendix 15.1.1**) and SAP (provided in **Appendix 15.1.9**).

All tables, listings and graphs were produced by Micron Research Limited (Ely, UK) using SAS® Version 9.4 or later (SAS Institute, Cary, NC 27513). Specifications for tables, graphs, and data listing formats for this study can be found in **Appendix 15.1.9**. All data collected on the eCRF were listed by participant; all randomized participants were included in the listings (found in **Appendix 15.2**). All tables, listings and graphs were subjected to a full quality control check, following their generation. Only the reason for discontinuation would be listed for participants classed as screening failures (i.e., a participant who had signed informed consent and had failed any inclusion or exclusion criteria or withdrew before any treatment had started).

Further to the SAP (**Appendix 15.1.9**), all summaries were presented by treatment group using the Alveavax-v1.2 dose ("Low Dose", "Standard", "High Dose", or "SC") as the column heading, plus a total column of the Alveavax-v1.2 dose groups combined (named "Combined", and the control group.

Categorical variables were summarized as frequencies and percentages. Unless otherwise stated, the denominator for percentages was the number of participants in each treatment group in the analysis population. Percentages were presented to one decimal place. A "Missing" would only be presented on the categorical summaries if the Investigator actually recorded "Missing" as an outcome.

Continuous variables were summarized using descriptive statistics (number of participants with an observation [n], mean, standard deviation [SD], median, and range). Unless otherwise specified, all continuous variables were given to one decimal place. No hypothesis tests were planned but confidence intervals, if reported, would be at the 95% level. In general, where partial dates were recorded on the eCRF (missing day or missing day and month) and where these cannot be resolved by queries, dates would be estimated for the purpose of calculating durations. Where a start/onset date was partial, the first day of the month would be assumed if the day was missing, and the first month of the study would be assumed if the month was missing. Where a stop/end date was partial, the last day of the month would be assumed if the

day was missing, and the final month of the study would be assumed if the month was missing. Partial concomitant medication and AE dates were dealt with on a case-by-case basis and discussed with the Sponsor where applicable.

The number of participants screened, the number of participants enrolled, including the number of participants enrolled but not treated and the number of participants enrolled and treated, were summarized by region and overall. Treated participants were defined as those that received at least one dose of study medication. The number and percentage of participants treated, the number and percentage of participants in each analysis population (defined in **Section 9.1.1** below), the number and percentage of participants completing the study and the number and percentage of participants withdrawing from the study, including the reasons for withdrawal, were summarized for all enrolled participants.

Descriptive summaries of participants' demographic and baseline characteristics were to be presented for the modified intent to treat (mITT) population by treatment group, region and overall. Demographics would include age (years), sex, race, height (m), weight (kg) and BMI (kg/m^2). Age was calculated from the date of birth (or year of birth) relative to the screening visit date if date of birth (or year of birth) is recorded.

8.7.1.2 Primary (Safety) endpoint methods

As this drug has never been tested in humans, the primary outcome was the measurement of the safety endpoints. All participants were instructed on how to measure any solicited local AEs, as described in Section 8.1.6 (Diary and Measurement Devices) of the protocol (**Appendix 15.1.1**).

All summary tables of safety data were to be presented for the Safety population. AEs were coded using MedDRA version 25.1. Coding included the system organ class and preferred term. Verbatim descriptions and coded terms were listed for all AEs.

The overall number of participants and incidence proportion (number of participants experiencing any AEs by the total number of participants) of any solicited local and systemic AEs within seven days of dose administration was presented. The incidence of each solicited AE (at MedDRA system organ class and preferred term level) was also shown. The overall number and incidence proportion of unsolicited AEs for up to 6 months after vaccination was analyzed in a similar fashion. For each participant, multiple occurrences of the same event were counted only once within a system organ class and preferred term i.e., as patient events. The denominator for percentages was the number of participants in the respective treatment groups.

SAEs, AESIs, and AEs leading to participant discontinuation were presented in the form of a listing, with number and proportion for each one. In addition, the overall incidence proportion of these categories of AE was to be shown.

For participant level tabulation a maximum intensity and highest relationship to investigational drug was to be presented in separate tables.

8.7.1.3 Secondary (Immunogenicity and Efficacy) endpoint methods

The secondary outcome was the measurement of the following immunogenicity parameters at baseline, summarized by study group:

- Geometric mean titer (GMT) of serum anti-SARS-CoV-2 BA.2 antibody
- GMT of BA.2 anti-spike protein (S) antibody
- BA.2 Spike specific CD4+ T-cell count
- BA.2 Spike specific CD8+ T-cell count
- GMT of anti-nucleocapsid protein (N) antibody
- Percentage of participants with neutralization IC_{50} ≥100 IU/mL
- Percentage of participants with neutralization IC_{50} > 200 IU/mL
- Percentage of participants with neutralization IC_{50} >400 IU/mL
- Percentage of participants with neutralization IC_{50} >800 IU/mL
- Baseline assessments of antibody dependent cellular cytotoxicity, phagocytosis and trogocytosis
- Baseline measures of complement fixation
- Baseline measures of Fc receptor binding

In addition, description of GMT, change in GMT, and GMFR of serum anti-spike protein (S) IgG antibody and the change in GMT and GFMR of anti-N protein (N) IgG antibody at Day 28.

The number and percentage of participants being found to be SARS-CoV-2 positive by external testing (score 1-10), number and percentage of participants with ambulatory mild disease (score 1-3), number and percentage of participants hospitalized with moderate disease (score 4-5), number and percentage of participants hospitalized with severe disease (score 6-9), number and percentage of participants who died (score 10) while being SARS-CoV-2 positive with the exact 95% CI was also to be presented separately for each day: Day 7, Day 14, Day 28, Day 84, Day 168.

8.7.1.4 Other efficacy endpoint methods

Characterization of humoral antibody immune response of booster vaccinations against ancestral and variant (Alpha, Beta, Delta, BA.1, and any newly discovered VOC) SARS-CoV-2 Strains measured using GMT of anti-SARS-CoV-2 neutralizing antibody at each timepoint (Pre-vaccination, Day 7, Day 14, Day 28, Day 84, and Day 168) were made using the approach described for the primary (safety) endpoint analyses.

Characterization of the cellular immune response following booster vaccinations against SARS-CoV-2 BA.2/Omicron measured using cellular immune responses CD4+ and CD8+ T-cell response.

The RLU and change in RLU of serum antibody-dependent cell-mediated cytotoxicity (ADCC) against existing and yet to be defined variants and sub-lineages at the defined time points presented unadjusted in tabular form.

It is to be noted that the exploratory analyses were planned but not measured due to a lack of relevant humoral immune response found in binding antibodies against SARS-CoV-2 BA.2 in any of the study cohorts at study Day 28.

8.7.1.5 Method(s) for statistical issues encountered during the analysis

There were no statistical issues encountered during the analysis.

8.7.2 Determination of Sample Size

Due to a number of exploratory analyses built into the study design, heuristic approach was used to select group sample sizes sufficient to assess safety across the five dosing groups and explore immunogenicity of the vaccine under study compared with the active control vaccine.

Groups of 40 participants each were enrolled in the standard dose and control arms of vaccinated individuals to assess safety and variability in immune response relative to baseline immunogenicity. Forty is the median group size in a recent systematic analysis of clinical trial sample sizes in viral diseases **[37]**.

The safety and immune response of the standard dose in vaccinated individuals were compared with groups of 20 participants each in the low dose and high dose arm in vaccinated individuals.

Ten participants were enrolled to assess safety and confirm seroconversion for SC injections.

The sample sizes projected for each dose group in the protocol (Section 9.2 of the protocol in **Appendix 15.1.1**) and SAP (see **Appendix 15.1.9**) were exactly met. The sample sizes were based on previous experience, not a statistical power calculation.

8.8 CHANGES IN THE CONDUCT OF THE STUDY OR PLANNED ANALYSES

8.8.1 Changes in the Conduct of the Study

Extensive conversations with Sponsor were a prerequisite for any amendments to the protocol. These amendments were recorded, dated, and signed by all signatories (or their successors) of the premier protocol design. Prior written approval of the Ethics Committee and SAHPRA was another prerequisite for changes to or deviations from the protocol. Changes made to prevent immediate hazards to participants or minor logistical changes did not need written approval; however, the Ethics Committee and SAHPRA had to be notified of them within 30 days.

A first protocol amendment was made between versions 2.0 and 3.0 of the protocol: the removal of treatment group 2A. This was participants who were unvaccinated but had been infected by COVID-19. This group was removed following review by, and on the request of the Clinical Trials Committee of the regulatory authority, SAHPRA. This resulted in the reduction of participant numbers from 150 to 130.

The second protocol amendment, made between versions 3.0 and 4.0, was a change in the safety email address due to the change of pharmacovigilance services provider from Bioclinica to Alvea, LLC. in-house pharmacovigilance. This required updated contact details in the protocol.

Protocol amendments were also made between versions 4.0 and 5.0. These were made due to the lack of relevant humoral immune response data at Day 28 in anti-SARS-CoV-2 BA.2 antibodies in any of the study cohorts. The neutralizing antibody responses in a subset of samples confirmed this observation. The Sponsor did not expect to find anything different by analyzing samples at different time points or with different testing methods. Therefore, the Sponsor recommended stopping any further tests (i.e., neutralizing antibody assays, cellular immunogenicity assessments, binding antibody tests) at other timepoints.

Protocol amendments are included in **Appendix 15.1.1** and described below in **Table 8.5**.

Table 8.5: Protocol amendments (Protocol Number: Alvea-VAX-P00001)

Date	Protocol version	Section	Reason	Old text	New text
13-May-22	v2.0 18March2022	Study title	Drop unvaccinated arm (2.a)	A Phase 1 open-label, active-controlled, randomized dose-finding study to evaluate safety, tolerability, and immunogenicity of intradermal and subcutaneous application of the plasmid DNA SARS-CoV-2 Omicron BA.2 vaccine Alveavax-v1.2 in primary Ad26.COV2.S vaccinated or SARS-CoV-2 recovered unvaccinated healthy individuals.	A Phase 1 open-label, active-controlled, randomized dose-finding study to evaluate safety, tolerability, and immunogenicity of intradermal and subcutaneous application of the plasmid DNA SARS-CoV-2 Omicron BA.2 vaccine Alveavax-v1.2 in primary Ad26.COV2.S vaccinated healthy individuals.
13-May-22	v2.0 18March2022	Synopsis: Target Sample	Drop unvaccinated arm (2.a)	150	130
13-May-22	v2.0 18March2022	Synopsis: Characteristics and Number of Participants	Drop unvaccinated arm (2.a)	150 healthy individuals (between 18 and 65 years of age, both inclusive), previously having received a primary Ad26.COV2.S vaccination series against SARS-CoV-2 or being unvaccinated and recovered from SARS-CoV-2, satisfying all the eligibility criteria will be eligible to participate in the study. Screening for eligible participants will be performed within 28 days of vaccination in the study.	130 healthy individuals (between 18 and 65 years of age, both inclusive), previously having received a primary Ad26.COV2.S vaccination series against SARS-CoV-2, satisfying all the eligibility criteria will be eligible to participate in the study. Screening for eligible participants will be performed within 28 days of vaccination in the study.
13-May-22	v2.0 18March2022	1.2 Schema	Drop unvaccinated arm (2.a)	Note: The enrollment will start with the first ten participants randomized into the low dose (1.a) arm, the control arm (1.e), subsequently at least 24 hours apart five participants to the vaccinated standard dose (1.b) arm, followed by a review of 24h safety data by the independent medical monitor. Then, the remaining participants assigned to the low and standard dose cohorts, in addition to those assigned to the control arm (1.e) and unvaccinated standard dose arm (2.a) will be recruited. In parallel, the first five participants of the high dose (1.c) arm will be enrolled. Further recruitment and the SC injection arm (1.d) start as soon as the independent medical monitor has reviewed 24h safety data of the high dose arm.	*Removed arm 2.a from image.*<br>Note: The enrollment will start with the first ten participants randomized into the low dose (1.a) arm, the control arm (1.e), subsequently at least 24 hours apart five participants to the vaccinated standard dose (1.b) arm, followed by a review of 24h safety data by the independent medical monitor. Then, the remaining participants assigned to the low and standard dose cohorts, in addition to those assigned to the control arm (1.e) will be recruited. In parallel, the first five participants of the high dose (1.c) arm will be enrolled. Further recruitment and the SC injection arm (1.d) start as soon as the independent medical monitor has reviewed 24h safety data of the high dose arm.

Date	Protocol version	Section	Reason	Old text	New text
13-May-22	v2.0 18March2022	2.1 Study Rationale	Drop unvaccinated arm (2.a)	The objective of this first in human Phase 1 dose-finding trial is to describe the safety, tolerability, and immunogenicity of Alveavax-v1.2 in primary vaccinated as well as recovered individuals.<br>[...]<br>In a first in human dose-finding study design we will investigate the immune responses of three dose intradermal (ID) levels in vaccinated individuals, and one ID dose in recovered individuals.	The objective of this first in human Phase 1 dose-finding trial is to describe the safety, tolerability, and immunogenicity of Alveavax-v1.2 in primary vaccinated individuals.<br>[...]<br>In a first in human dose-finding study design we will investigate the immune responses of three dose intradermal (ID) levels in vaccinated individuals.
13-May-22	v2.0 18March2022	4.1 Overall Design	Drop unvaccinated arm (2.a)	This is a first in human, open-label, active-controlled, randomized dose-finding study to evaluate safety, tolerability, and immunogenicity of ID and SC application of the plasmid DNA SARS-CoV-2 Omicron BA.2 vaccine Alveavax-v1.2 in primary Ad26.COV2.S vaccinated or SARS-CoV-2 recovered unvaccinated healthy individuals.<br>Primary Ad26.COV2.S vaccinated participants will be randomized into one of 5 treatment arms to receive Alveavax-v1.2 or a Ad26.COV2.S control booster vaccine. Unvaccinated participants will receive a predetermined dose of Alveavax-v1.2.	This is a first in human, open-label, active-controlled, randomized dose-finding study to evaluate safety, tolerability, and immunogenicity of ID and SC application of the plasmid DNA SARS-CoV-2 Omicron BA.2 vaccine Alveavax-v1.2 in primary Ad26.COV2.S vaccinated healthy individuals.<br>Primary Ad26.COV2.S vaccinated participants will be randomized into one of 5 treatment arms to receive Alveavax-v1.2 or a Ad26.COV2.S control booster vaccine.
13-May-22	v2.0 18March2022	4.1 Overall Design	Drop unvaccinated arm (2.a)	A total of 150 male and female participants aged between 18 and 65 years who satisfy the inclusion and exclusion criteria are planned to be enrolled in six groups and with vaccine administered according to Table 3:<br>Individuals with a primary vaccination will get either:<br>Low dose: 0.5 mg Alveavax-v1.2 in one ID injection<br>Standard dose: 2 mg Alveavax-v1.2 in one ID injection<br>High dose: 8 mg Alveavax-v1.2 in four ID injections*<br>SC injection: 8 mg Alveavax-v1.2 in one SC injection<br>Control: Janssen Ad26.COV2.S in one IM injection<br>Unvaccinated individuals will get Standard dose: 2 mg Alveavax-v1.2 in one ID injection	A total of 130 male and female participants aged between 18 and 65 years who satisfy the inclusion and exclusion criteria are planned to be enrolled in five groups and with vaccine administered according to Table 3:<br>Individuals with a primary vaccination will get either:<br>Low dose: 0.5 mg Alveavax-v1.2 in one ID injection<br>Standard dose: 2 mg Alveavax-v1.2 in one ID injection<br>High dose: 8 mg Alveavax-v1.2 in four ID injections*<br>SC injection: 8 mg Alveavax-v1.2 in one SC injection<br>Control: Janssen Ad26.COV2.S in one IM injection
13-May-22	v2.0 18March2022	4.1 Overall Design	Drop unvaccinated arm (2.a)	The enrollment will start with the first five participants in the low dose (1.a) arm and the vaccinated standard dose (1.b) arm, followed by a review of 24h safety data by the independent medical monitor. Then, the remaining participants from the low and standard dose arms, as well as the control arm (1.e) and unvaccinated standard dose arm (2.a) will be recruited. In parallel, the first five participants of the high dose (1.c) arm	The enrollment will start with the first five participants in the low dose (1.a) arm and the vaccinated standard dose (1.b) arm, followed by a review of 24h safety data by the independent medical monitor. Then, the remaining participants from the low and standard dose arms, as well as the control arm (1.e) will be recruited. In parallel, the first five

Date	Protocol version	Section	Reason	Old text	New text
				are enrolled. Further recruitment and the SC injection arm (1.d) start as soon as the independent medical monitor has reviewed 24h safety data of the high dose arm (see schema in 1.2).	participants of the high dose (1.c) arm are enrolled. Further recruitment and the SC injection arm (1.d) start as soon as the independent medical monitor has reviewed 24h safety data of the high dose arm (see schema in 1.2).
13-May-22	v2.0 18March2022	5 Study Population	Drop unvaccinated arm (2.a)	Healthy adult individuals, previously having received a primary Ad26.COV2.S vaccination series against SARS-CoV-2 or being unvaccinated and recovered from SARS-CoV-2, satisfying all the eligibility criteria will be eligible to participate in the study. Screening for eligible participants will be performed within 28 days of vaccination in the study.	Healthy adult individuals, previously having received a primary Ad26.COV2.S vaccination series against SARS-CoV-2, satisfying all the eligibility criteria will be eligible to participate in the study. Screening for eligible participants will be performed within 28 days of vaccination in the study.
13-May-22	v2.0 18March2022	5.1 Number of Participants Planned	Drop unvaccinated arm (2.a)	A total of 150 healthy participants will be enrolled in six groups.	A total of 130 healthy participants will be enrolled in five groups.
13-May-22	v2.0 18March2022	5.2 Inclusion Criteria	Drop unvaccinated arm (2.a)	Participants who received a primary Janssen Ad26.COV2.S vaccine ≥ 60 days prior to receiving the study vaccine and/or are recovered from SARS-CoV-2, defined as a history of a positive SARS-CoV-2 test (e.g. PCR, rapid antigen test, etc.) ≥ 60 days prior to receiving the study vaccination (Day 1) in this study.	Participants who received a primary Janssen Ad26.COV2.S vaccine ≥ 60 days prior to receiving the study vaccine (Day 1) in this study.
13-May-22	v2.0 18March2022	6.2 Dosage and Treatment Schedule	Drop unvaccinated arm (2.a)		Table 3: Dosing Schema Remove Group 2
13-May-22	v2.0 18March2022	6.5.1 Procedures for Randomization and Stratification	Drop unvaccinated arm (2.a)	Group 2 will not be randomized as it contains only a single intervention.	*[removed]*
13-May-22	v2.0 18March2022	9.2 Sample size determination	Drop unvaccinated arm (2.a)	The safety and immune response of the standard dose in vaccinated individuals will be compared with groups of 20 participants each in the low dose and high dose arm in vaccinated individuals as well as the standard dose in unvaccinated, recovered individuals.	The safety and immune response of the standard dose in vaccinated individuals will be compared with groups of 20 participants each in the low dose and high dose arm in vaccinated individuals.
13-May-22	v2.0 18March2022	12. Signature Page	Drop unvaccinated arm (2.a)		[removed date]
13-May-22	v2.0 18March2022	Appendix 5: Protocol amendments	Drop unvaccinated arm (2.a)		[Added appendix]
13-May-22	v2.0 18March2022	Previous Version	Drop unvaccinated arm (2.a)	[none]	2.0
13-May-22	v2.0 18March2022	Protocol Version	Drop unvaccinated arm (2.a)	2.0	3.0
25-Aug-22	v3.0 16May2022	1.3 SCHEDULE OF ACTIVITIES	Updated schedule of activities	Urine pregnancy test done at check-in	Urine pregnancy test will be performed for female participants of childbearing potential on screening, pre-dose, on Day 28, and on Day 84

Date	Protocol version	Section	Reason	Old text	New text
		(SOA, TABLE 1)			
25-Aug-22	v3.0 16May2022	8.5.4 Evaluating Adverse Events	Change safety email address	safety.alvea@bioclinica.com	safety.alvea-vax-p00001@alveavax.com
25-Aug-22	v3.0 16May2022	Protocol Version	Change safety email address	3.0	4.0
16-Nov-22	v4.0 13-Sep-2022	Protocol Version	End study after 6 months	2022-09-13 7pm CEST	2022-11-16 9am CEST
16-Nov-22	v4.0 13-Sep-2022	Protocol Version	End study after 6 months	4.0	5.0
16-Nov-22	v4.0 13-Sep-2022	Protocol Version	End study after 6 months	3.0	4.0
16-Nov-22	v4.0 13-Sep-2022	Protocol Version	End study after 6 months	April 2022 (planned)	April 2022
16-Nov-22	v4.0 13-Sep-2022	Protocol Version	End study after 6 months	Recruiting	Enrollment completed
16-Nov-22	v4.0 13-Sep-2022	Protocol Version	End study after 6 months	4.0	5.0
16-Nov-22	v4.0 13-Sep-2022	1.1 Synopsis	End study after 6 months	16 months	10 months
16-Nov-22	v4.0 13-Sep-2022	1.1 Synopsis	End study after 6 months	Efficacy assessments will include the following:<br>Clinical efficacy measured using the WHO clinical progression scale for COVID-19<br>Percentage of participants with BA.2 neutralization IC_{50} >100 IU/mL<br>Percentage of participants with BA.2 neutralization IC_{50} >200 IU/mL<br>Percentage of participants with BA.2 neutralization IC_{50} >400 IU/mL<br>Percentage of participants with BA.2 neutralization IC_{50} >800 IU/mL<br>Absolute number and fraction of ID injections which generated a ≥ 1 mm and ≥ 7 mm in diameter clearly demarcated bleb, clearly visible for at least 20 seconds, for 0.5 mg and 2 mg Alveavax-v1.2 respectively<br>Percentage of participants with neutralization IC_{50} >64 IU/mL against the respective strain<br>Percentage of participants with neutralization IC_{50} >128 IU/mL against the respective strain<br>Serologic change in GMT and GMFR between baseline and other blood samples for anti-nucleocapsid protein (N) IgG antibodies	Efficacy assessments will include the following:<br>Clinical efficacy measured using the WHO clinical progression scale for COVID-19<br>Absolute number and fraction of ID injections which generated a ≥ 1 mm and ≥ 7 mm in diameter clearly demarcated bleb, clearly visible for at least 20 seconds, for 0.5 mg and 2 mg Alveavax-v1.2 respectively<br>Percentage of participants with neutralization IC_{50} >64 IU/mL against the respective strain<br>Percentage of participants with neutralization IC_{50} >128 IU/mL against the respective strain<br>Serologic change in GMT and GMFR between baseline and other blood samples for anti-nucleocapsid protein (N) IgG antibodies
16-Nov-22	v4.0 13-Sep-2022	1.1 Synopsis	End study after 6 months	Description of GMT, change in GMT, and GMFR of serum anti-SARS-CoV-2 neutralizing antibody titers and the change in GMT and GFR of anti-spike protein (S) IgG	Description of GMT, change in GMT, and GMFR of serum anti-spike protein (S) IgG antibody titers and the change in GMT

Date	Protocol version	Section	Reason	Old text	New text
				antibody and anti-N protein (N) IgG antibody at Day 7, Day 14, Day 28, Day 84, and Day 168 and Day 365.	and GFR of anti-N protein (N) IgG antibody at Day 28.
16-Nov-22	v4.0 13-Sep-2022	1.1 Synopsis	End study after 6 months	The number and percentage of participants being found to be SARS-CoV-2 positive by external testing (score 1-10), number and percentage of participants with ambulatory mild disease (score 1-3), number and percentage of participants hospitalized with moderate disease (score 4-5), number and percentage of participants hospitalized with severe disease (score 6-9), number and percentage of participants who died (score 10) while being SARS-CoV-2 positive with the exact 95% CI will also be presented separately for each day: Day 7, Day 14, Day 28, Day 84, Day 168, Day 365.	The number and percentage of participants being found to be SARS-CoV-2 positive by external testing (score 1-10), number and percentage of participants with ambulatory mild disease (score 1-3), number and percentage of participants hospitalized with moderate disease (score 4-5), number and percentage of participants hospitalized with severe disease (score 6-9), number and percentage of participants who died (score 10) while being SARS-CoV-2 positive with the exact 95% CI will also be presented separately for each day: Day 7, Day 14, Day 28, Day 84, Day 168.
16-Nov-22	v4.0 13-Sep-2022	1.1 Synopsis	End study after 6 months	Characterization of humoral antibody immune response of booster vaccinations against Ancestral and Variant (Alpha, Beta, Delta, BA.1, and any newly discovered VOC) SARS-CoV-2 Strains measured using GMT of anti-SARS-CoV-2 neutralizing antibody at each timepoint (Pre-vaccination, Day 7, Day 14, Day 28, Day 84, and Day 168 and Day 365) will be made using the approach described for the primary endpoint analyses.	Characterization of humoral antibody immune response of booster vaccinations against Ancestral and Variant (Alpha, Beta, Delta, BA.1, and any newly discovered VOC) SARS-CoV-2 Strains measured using GMT of anti-SARS-CoV-2 neutralizing antibody at each timepoint (Pre-vaccination, Day 7, Day 14, Day 28, Day 84, and Day 168) will be made using the approach described for the primary endpoint analyses.
16-Nov-22	v4.0 13-Sep-2022	1.3 Schedule of Activities (assessment)	End study after 6 months	D14 ±2d,<br>D28 ±2d,<br>D84 ±7d,<br>D168 ±14d	D14 ±2d,<br>D28 ±2d,<br>D84 ±7d
16-Nov-22	v4.0 13-Sep-2022	1.3 Schedule of Activities (assessment)	End study after 6 months	D365 ±14d<br>(EOS)	D168 ±14d<br>(EOS)
16-Nov-22	v4.0 13-Sep-2022	1.3 Schedule of Activities (assessment)	End study after 6 months	V4, 5, 6, 7<br>V8	V4, 5, 6<br>V7
16-Nov-22	v4.0 13-Sep-2022	3 Objectives and Endpoints	End study after 6 months	The objective of the study is to assess the tolerability, safety, and immunogenicity of different doses and routes of administration of the Alveavax-v1.2 vaccine in healthy individuals.	The objective of the study is to assess the tolerability, safety, and immunogenicity of different doses and routes of administration of the Alveavax-v1.2 vaccine in healthy individuals.<br>Exploratory endpoints will or will not be tested based on findings from secondary endpoints and sponsor's determination accordingly.
16-Nov-22	v4.0 13-Sep-2022	3 Objectives and Endpoints	End study after 6 months	Day 1, 7, 28, after 3, and 6, and 12 months	Day 1, 7, 28, after 3, and 6 months

Date	Protocol version	Section	Reason	Old text	New text
16-Nov-22	v4.0<br>13-Sep-2022	3 Objectives and Endpoints (Humoral immune response)	End study after 6 months	**Humoral immune response**<br>Characterization of humoral immune response of booster vaccinations against SARS-CoV-2 BA.2/Omicron measured using the following:<br>1. Change in geometric mean titer (GMT) of serum anti-SARS-CoV-2 BA.2 neutralizing antibody titers<br>2.GMT of serum anti-SARS-CoV-2 BA.2 neutralizing antibody titers<br>3.Geometric mean fold rise (GMFR) of serum anti-SARS-CoV-2 BA.2 neutralizing antibody<br>4. Four-fold increase rate of anti-SARS-CoV-2 BA.2 neutralizing antibody<br>5. Change in GMT of anti-spike protein (S) immunoglobulin G (IgG) antibody<br>6. GMT of anti-spike protein (S) IgG antibody<br>7.GMFR of anti-spike protein (S) IgG<br>8. Percentage of participants with neutralization IC_{50} >100 IU/mL<br>9. Percentage of participants with neutralization IC_{50} >200 IU/mL<br>10. Percentage of participants with neutralization IC_{50} >400 IU/mL<br>11. Percentage of participants with neutralization IC_{50} >800 IU/mL	**Humoral immune response**<br>Characterization of humoral immune response of booster vaccinations against SARS-CoV-2 BA.2/Omicron measured using the following:<br>1. Change in GMT of anti-spike protein (S) immunoglobulin G (IgG) antibody<br>2. GMT of anti-spike protein (S) IgG antibody<br>3. GMFR of anti-spike protein (S) IgG
16-Nov-22	v4.0<br>13-Sep-2022	3 Objectives and Endpoints (Humoral immune response)	End study after 6 months	**Humoral immune response**<br>Baseline (pre-vaccination; except for Point 1), Day 7,<br>Day 14,<br>Day 28,<br>Day 84,<br>Day 168,<br>Day 365	Baseline (pre-vaccination; except for Point 1),<br>Day 28
16-Nov-22	v4.0<br>13-Sep-2022	3 Objectives and Endpoints (Clinical efficacy)	End study after 6 months	Day 7,<br>Day 14,<br>Day 28,<br>Day 84,<br>Day 168,<br>Day 365	Day 7,<br>Day 14,<br>Day 28,<br>Day 84,<br>Day 168
16-Nov-22	v4.0<br>13-Sep-2022	3 Objectives and Endpoints (Cellular immune response)	End study after 6 months	Baseline (pre-vaccination),<br>Day 28,<br>Day 168	Baseline (pre-vaccination),<br>Day 28
16-Nov-22	v4.0<br>13-Sep-2022	3 Objectives and Endpoints (Cellular immune response)	End study after 6 months	Baseline (pre-vaccination; except for Point 1),<br>Day 7,<br>Day 14,<br>Day 28,<br>Day 84,<br>Day 168,<br>Day 365	Baseline (pre-vaccination; except for Point 1),<br>Day 7,<br>Day 14,<br>Day 28,<br>Day 84,<br>Day 168
16-Nov-22	v4.0<br>13-Sep-2022	3 Objectives and Endpoints (Cellular	End study after 6 months	Baseline (pre-vaccination; except for Point 1),<br>Day 7,	Baseline (pre-vaccination; except for Point 1),<br>Day 7,

Date	Protocol version	Section	Reason	Old text	New text
		immune response)		Day 14,<br>Day 28,<br>Day 84,<br>Day 168,<br>Day 365	Day 14,<br>Day 28,<br>Day 84,<br>Day 168
16-Nov-22	v4.0<br>13-Sep-2022	3 Objectives and Endpoints (Cellular immune response)	End study after 6 months	Day 7,<br>Day 14,<br>Day 28,<br>Day 84,<br>Day 168,<br>Day 365	Day 7,<br>Day 14,<br>Day 28,<br>Day 84,<br>Day 168
16-Nov-22	v4.0<br>13-Sep-2022	3 Objectives and Endpoints (Cellular immune response)	End study after 6 months	Baseline, Day 7,<br>Day 14,<br>Day 28,<br>Day 84,<br>Day 168,<br>Day 365	Baseline, Day 7,<br>Day 14,<br>Day 28,<br>Day 84,<br>Day 168
16-Nov-22	v4.0<br>13-Sep-2022	4.2 Scientific Rationale for Study Design	End study after 6 months	The Alveavax-v1.2 vaccine is being developed to prevent severe cases of COVID-19, the disease resulting from SARS-CoV-2 infection. The study is designed to primarily evaluate the tolerability and safety of Alveavax-v1.2 for up to 12 months after the booster dose with Alveavax-v1.2. Additionally the study will determine immunogenicity endpoints and descriptive clinical efficacy against SARS-CoV-2 infection as secondary objectives.	The Alveavax-v1.2 vaccine is being developed to prevent severe cases of COVID-19, the disease resulting from SARS-CoV-2 infection. The study is designed to primarily evaluate the tolerability and safety of Alveavax-v1.2 for up to 6 months after the booster dose with Alveavax-v1.2. Additionally the study will determine immunogenicity endpoints and descriptive clinical efficacy against SARS-CoV-2 infection as secondary objectives.
16-Nov-22	v4.0<br>13-Sep-2022	8.2 Visit Schedules	End study after 6 months	**Day 14 (± 2 days), Day 28 (± 2 days), and Day 84 (± 7 days), and Day 168 (± 14 days)**<br>- Recording of adverse events<br>- Blood sample collection for immunogenicity and cellular response analysis<br>- Confirm with female participants of childbearing potential and male participants who have a female partner if they/their partner became pregnant since Day 1<br>- Recording of COVID-19 infections and vaccinations since Day 1<br>- NP swab sample for COVID-19 PCR on Day 14 and Day 28	**Day 14 (± 2 days), Day 28 (± 2 days), and Day 84 (± 7 days)**<br>- Recording of adverse events<br>- Blood sample collection for immunogenicity and cellular response analysis<br>- Confirm with female participants of childbearing potential and male participants who have a female partner if they/their partner became pregnant since Day 1<br>- Recording of COVID-19 infections and vaccinations since Day 1<br>- NP swab sample for COVID-19 PCR on Day 14 and Day 28
16-Nov-22	v4.0<br>13-Sep-2022	4.2 Visit Schedules	End study after 6 months	**Day 365 (± 14 days) (End-of-Study)**<br>- Abbreviated, symptom-directed physical examination<br>- Vital signs and weight<br>- Recording of adverse events<br>- Blood sample collection for immunogenicity and cellular response analysis<br>- Recording of COVID-19 infections and vaccinations since Day 1	Day 168 (± 14 days) (End-of-Study)<br>- Abbreviated, symptom-directed physical examination<br>- Vital signs and weight<br>- Recording of adverse events<br>- Blood sample collection for immunogenicity response analysis<br>- Recording of COVID-19 infections and vaccinations since Day 1

Date	Protocol version	Section	Reason	Old text	New text
				- Confirm with female participants of childbearing potential and male participants who have a female partner if they/their partner became pregnant since Day 1 of the study.	- Confirm with female participants of childbearing potential and male participants who have a female partner if they/their partner became pregnant since Day 1 of the study.
16-Nov-22	v4.0 13-Sep-2022	8.5 Adverse Events	End study after 6 months	**Treatment-Emergent Adverse Event (TEAE)** A treatment-emergent adverse event (TEAE) is defined as any event at or after the time of exposure to study vaccine or any event already present that worsens in either intensity or frequency following exposure to the study vaccine, until the end of the participant's participation in the study (i.e., Visit 8, Day 365 in Table 1, participant withdrawal, or participant lost to follow-up).	**Treatment-Emergent Adverse Event (TEAE)** A treatment-emergent adverse event (TEAE) is defined as any event at or after the time of exposure to study vaccine or any event already present that worsens in either intensity or frequency following exposure to the study vaccine, until the end of the participant's participation in the study (i.e., Visit 7, Day 168 in Table 1, participant withdrawal, or participant lost to follow-up).
16-Nov-22	v4.0 13-Sep-2022	8.5.2 Recording and Handling of Adverse Events	End study after 6 months	SAEs, AESIs, and AEs leading to study drug withdrawal should be collected starting from screening until the end of study (Day 365, Visit 8 in Table 1) and reported at each visit during the study.	SAEs, AESIs, and AEs leading to study drug withdrawal should be collected starting from screening until the end of study (Day 168, Visit 7 in Table 1) and reported at each visit during the study.
16-Nov-22	v4.0 13-Sep-2022	8.5 Recording and Handling of Adverse Events (Table 6)	End study after 6 months	**Duration of Collection*** 7 days (Day 1 and 6 subsequent days) after the vaccination<br>Screening and 28 days (Visit 5) after the vaccination during the whole study<br>(Screening until Day 365) during the whole study<br>(Screening until Day 365) during the whole study<br>(Screening until Day 365)<br>After the vaccination (Day 1 until Day 365)	**Duration of Collection*** 7 days (Day 1 and 6 subsequent days) after the vaccination<br>Screening and 28 days (Visit 5) after the vaccination during the whole study<br>(Screening until Day 168) during the whole study<br>(Screening until Day 168) during the whole study<br>(Screening until Day 168)<br>After the vaccination (Day 1 until Day 168)
16-Nov-22	v4.0 13-Sep-2022	8.5.5 Reporting Serious Adverse Events	End study after 6 months	**Discontinuation of the Study due to Adverse Events** The reason for a participant being discontinued from the study will be recorded in the CRF. A discontinuation occurs when an enrolled participant ceases participation in the study, regardless of the circumstances, prior to the completion of the study. A discontinuation must be reported immediately to the Sponsor if it is due to a SAE. The final evaluation as required by the protocol (see Table 1 Visit 8, Day 365) will be performed at the time of discontinuation if medically acceptable or as soon as possible after that. The Investigator will record the reason for study discontinuation and, if possible, provide or arrange for appropriate follow-up and document the cause of the participant's condition.	**Discontinuation of the Study due to Adverse Events** The reason for a participant being discontinued from the study will be recorded in the CRF. A discontinuation occurs when an enrolled participant ceases participation in the study, regardless of the circumstances, prior to the completion of the study. A discontinuation must be reported immediately to the Sponsor if it is due to a SAE. The final evaluation as required by the protocol (see Table 1 Visit 7, Day 168) will be performed at the time of discontinuation if medically acceptable or as soon as possible after that. The Investigator will record the reason for study discontinuation and, if possible, provide or arrange for

Date	Protocol version	Section	Reason	Old text	New text
					appropriate follow-up and document the cause of the participant's condition.
16-Nov-22	v4.0 13-Sep-2022	9.4.3. Secondary Endpoints Analysis	End study after 6 months	Description of GMT, change in GMT, and GMFR of serum anti-SARS-CoV-2 neutralizing antibody titers and the change in GMT and GFR of anti-spike protein (S) IgG antibody and anti-N protein (N) IgG antibody at Day 7, Day 14, Day 28, Day 84, and Day 168 and Day 365.<br><br>Table showing the number and percentage of participants with neutralization IC_{50} >100 IU/ml, IC_{50} >200 IU/mL, IC_{50} >400 IU/mL, and IC_{50} >800 IU/mL against the Omicron variant with corresponding 95% CI.	Description of GMT, change in GMT, and GMFR of anti-spike protein (S) IgG antibody and the change in GMT and GFR of anti-N protein (N) IgG antibody at Day 28.
16-Nov-22	v4.0 13-Sep-2022	9.4.3. Secondary Endpoints Analysis	End study after 6 months	The number and percentage of participants being found to be SARS-CoV-2 positive by external testing (score 1-10), number and percentage of participants with ambulatory mild disease (score 1-3), number and percentage of participants hospitalized with moderate disease (score 4-5), number and percentage of participants hospitalized with severe disease (score 6-9), number and percentage of participants who died (score 10) while being SARS-CoV-2 positive with the exact 95% CI will also be presented separately for each day: Day 7, Day 14, Day 28, Day 84, Day 168, and Day 365.	The number and percentage of participants being found to be SARS-CoV-2 positive by external testing (score 1-10), number and percentage of participants with ambulatory mild disease (score 1-3), number and percentage of participants hospitalized with moderate disease (score 4-5), number and percentage of participants hospitalized with severe disease (score 6-9), number and percentage of participants who died (score 10) while being SARS-CoV-2 positive with the exact 95% CI will also be presented separately for each day: Day 7, Day 14, Day 28, Day 84, and Day 168.
16-Nov-22	v4.0 13-Sep-2022	9.4.6. Exploratory Analysis	End study after 6 months	Characterization of humoral antibody immune response of booster vaccinations against Ancestral and Variant (Alpha, Beta, Delta, BA.1, and any newly discovered VOC) SARS-CoV-2 Strains measured using GMT of anti-SARS-CoV-2 neutralizing antibody at each timepoint (Pre-vaccination, Day 7, Day 14, Day 28, Day 84, Day 168 and Day 365) will be made using the approach described for the primary endpoint analyses.	Characterization of humoral antibody immune response of booster vaccinations against Ancestral and Variant (Alpha, Beta, Delta, BA.1, and any newly discovered VOC) SARS-CoV-2 Strains measured using GMT of anti-SARS-CoV-2 neutralizing antibody at each timepoint (Pre-vaccination, Day 7, Day 14, Day 28, Day 84, and Day 168) will be made using the approach described for the primary endpoint analyses.
16-Nov-22	v4.0 13-Sep-2022	Appendix 2: Blood collection	End study after 6 months	At each on-site visit, blood will be collected as per Schedule of Activities (Table 1). Blood for serology tests, hematology and chemistry, and for the measurement of humoral and cell-mediated immune responses, will be collected as follows (Table 7):<br>The vaccination time and sampling times should be recorded accurately in the appropriate section of the eCRF.	At each on-site visit, blood will be collected as per Schedule of Activities (Table 1). Blood for serology tests, hematology and chemistry, and for the measurement of humoral and cell-mediated immune responses, will be collected as follows (Table 7):<br>The vaccination time and sampling times should be recorded accurately in the appropriate section of the eCRF.

Date	Protocol version	Section	Reason	Old text	New text
				The total blood volume to be taken from each participant over the study period is about 150 mL, and will be as follows:	The total blood volume to be taken from each participant over the study period is about 120 mL, and will be as follows:
16-Nov-22	v4.0 13-Sep-2022	Appendix 2: Blood collection (Table 7 Blood collection at Study Visit)	End study after 6 months	Visit 7<br>Follow-up: Day 168<br>Humoral immunology<br>Cellular immunology<br>5 mL<br>30 mL	*[removed row]*
16-Nov-22	v4.0 13-Sep-2022	Appendix 2: Blood collection (Table 7 Blood collection at Study Visit)	End study after 6 months	Visit 8<br>End of study: Day 365<br>Humoral immunology<br>5 mL	Visit 7<br>End of study: Day 168<br>Humoral immunology<br>5 mL

8.8.2 Changes in the Planned Analysis

The SAP was finalized on 20th October 2022, prior to database lock.

For the safety analyses, the following adverse event tables were produced:

- All AEs throughout the study
- All SAEs throughout the study
- All AEs regarded as possibly or probably related to the vaccination
- All AEs leading to withdrawal from the study
- All AEs where the outcome was fatal

For the main immunogenicity analysis, due to the lack of a relevant humoral immune response at Day 28 in any of the Alveavax-v1.2 dose groups, the study was stopped by the Sponsor after 6 months, and the main efficacy result was at Day 28. However, some immunogenicity data before Day 28 was incomplete because the Sponsor recommended stopping laboratory analysis of samples to reduce costs. Participants continued to be monitored for safety for 6 months after completion of all study vaccinations.

Departures from the protocol were entirely occasioned by the Sponsor's decision to terminate the study after 6 months and to reduce the analysis of the immunology data. The protocol was amended to take account of the changes, but the SAP was not.

9 STUDY PARTICIPANTS

These were healthy adult individuals, previously having received a primary Ad26.COV2.S vaccination series against SARS-CoV-2, satisfying all the eligibility criteria. All patient listings can be found in **Appendix 15.2**.

9.1 DISPOSITION OF PARTICIPANTS

130 participants were enrolled in the study and vaccinated with either the Alveavax-v1.2 vaccine, or the Janssen Ad26.COV2.S vaccine in the case of the control group. The reasons for discontinuation are summarized in **Figure 9.1** and **Table 9.3**, are and listed by center and treatment group in **Appendix 15.2.1, Listing 1**. The study had only a control group and there was no negative (unvaccinated) control.

The study groups and number of participants were as follows:

- Group 1.a: Low dose group, 0.5 mg Alveavax-v1.2 in one ID injection (20 participants)
- Group 1.b: Standard dose group, 2 mg Alveavax-v1.2 in one ID injection (40 participants)
- Group 1.c: High dose group, 8 mg Alveavax-v1.2 as four ID injections of 2 mg (20 participants)
- Group 1.d: SC injection group, 8 mg Alveavax-v1.2 as a single SC injection (10 participants)
- Group 1.e: Control booster group, Janssen Ad26.COV2.S, 8.92 $\log_{10}$ infectious units/0.5 mL, as a single IM injection (40 participants)

In the statistical tables, the four Alveavax-v1.2 dose groups are shown separately, with a fifth column consisting of all four dose levels of Alveavax-v1.2 combined (N = 90). The control group is presented separately.

Figure 9.1: Disposition of Participants (Protocol Number: Alvea-VAX-P00001)

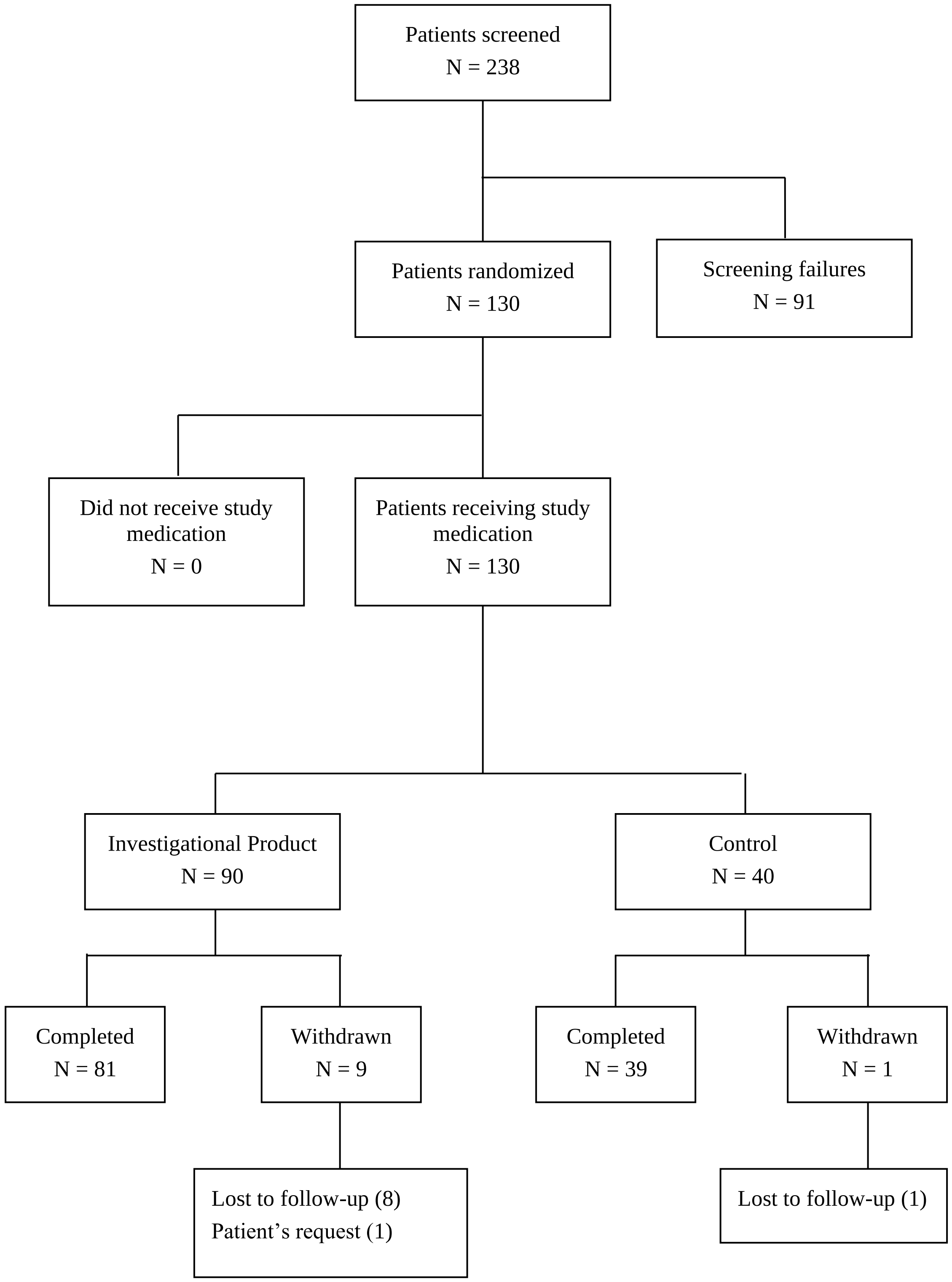

Data source: Appendix 15.2.1, Listing 1.

9.1.1 Study populations

The date of first enrollment was 30 June 2022; the date that the last participant completed was 28 February 2023.

Safety Population

The safety population was the set of all enrolled participants who were administered with a dose of the investigational product. Participants were grouped as treated.

Modified Intent to Treat Population (mITT)

All enrolled participants who were administered with the vaccine and experienced at least one post-baseline immunogenicity readout comprised the mITT population. Missing or non-evaluable measurements were not replaced. Participants were grouped as treated.

Per Protocol (PP) Population

The PP analysis population included all enrolled participants who met all the inclusion/exclusion criteria and who did not have any major protocol deviations. Participants were grouped as treated.

The modified intent-to-treat (mITT) and Safety populations were identical. The definition of mITT in the protocol and SAP, that participants had to be vaccinated and have at least one post-baseline immunogenicity test, was not feasible due to the cancellation of planned immunogenicity tests. All participants had data up to Day 7 and were therefore regarded as mITT.

The PP population was used only for the immunogenicity analysis **(Table 11.1)**. Participants were excluded from the PP population if they failed to complete the study, did not receive the full dose on vaccination, or did not have a Day 28 immunogenicity result **(Table 9.4)**. This last point represents a change from the SAP but is included since the study was effectively ended at Day 28, except for safety data.

The analysis populations are shown in **Table 9.1**.

Table 9.1: Number of Participants Included in the Efficacy Analysis (Protocol Number: Alvea-VAX-P00001)

Study population	Low Dose N = 20	Standard N = 40	High Dose N = 20	SC Injection N = 10	Combined N = 90	Control N = 40
**mITT**	18 (90.0%)	37 (92.5%)	17 (85.0%)	9 (90.0%)	81 (90.0%)	39 (97.5%)
**Safety**	18 (90.0%)	37 (92.5%)	17 (85.0%)	9 (90.0%)	81 (90.0%)	39 (97.5%)
**PP**	18 (90.0%)	36 (90.0%)	15 (75.0%)	9 (90.0%)	78 (86.7%)	38 (95.0%)

Data source: Section 13.1, Table 1.

9.1.2 Number of Participants at Each Visit

All 130 patients attended Visits 1 and 2 on Study Days 1 and 7, respectively. More than 98% (128/130) of patients attended Visit 3 (Day 14) and almost 97% (126/130) of patients attended Visit 4 (Day 28) (**Table 9.2**).

Table 9.2: Number (%) of Participants Assessed at Each Visit – mITT Population (Protocol Number: Alvea-VAX-P00001)

Visit no.	Day	Low Dose N = 20	Standard N = 40	High Dose N = 20	SC Injection N = 10	Combined N = 90	Control N = 40
**BL/1.**	**1**	20 (100%)	40 (100%)	20 (100%)	10 (100%)	90 (100%)	40 (100%)
**3.**	**7**	20 (100%)	40 (100%)	20 (100%)	10 (100%)	90 (100%)	40 (100%)
**4.**	**14**	20 (100%)	40 (100%)	19 (95.0%)	10 (100%)	89 (98.9%)	39 (97.5%)
**5.**	**28**	20 (100%)	40 (100%)	18 (90.0%)	9 (90.0%)	87 (96.7%)	39 (97.5%)

BL, baseline.

Data source: **Section 13.1, Table 2.1** (Baseline) and **Section 13.3, Table 3.5** (Day 7, Day 14, and Day 28).

A total of 10 participants of the 130 enrolled did not complete the study. The reasons for withdrawal are summarized in **Table 9.3**.

Table 9.3: Reasons for Premature Study Termination – mITT Population (Protocol Number: Alvea-VAX-P00001)

Reason	Low Dose N = 20	Standard N = 40	High Dose N = 20	SC Injection N = 10	Combined N = 90	Control N = 40
**Withdrawal by participant**	0 (0.0%)	1 (2.5%)	0 (0.0%)	0 (0.0%)	1 (1.1%)	0 (0.0%)
**Lost to follow-up***	2 (10.0%)	2 (5.0%)	3 (15.0%)	1 (10.0%)	8 (8.9%)	1 (2.5%)

Data source: Appendix 15.2.1, Listing 1.

*Includes two participants incorrectly recorded by the Investigator as being withdrawn due to a physician decision but were confirmed by the site as being lost to follow-up.

All participants who discontinued from the study are listed by center and treatment group in **Appendix 15.2.1, Listing 1**.

9.2 PROTOCOL DEVIATIONS

Any actions not compliant with the protocol guidelines were considered a deviation. These actions could have been the deviation of the participant, Investigator, or other clinical trial staff. Remedial actions may have been created and enacted by the site as they saw fit for any deviations.

Protocol deviations were outlined and documented. This document was kept in the participant's source document and in the site file. Local IECs were notified of protocol deviations, as recommended by respective IECs. It was the duty of the Investigator and all employees to understand and act upon the recommended notification process to IECs for deviations.

Any deviations from the planned vaccination program were recorded and summarized as frequencies and percentages. In the event that a participant received the wrong vaccination, they would be included in the treatment group of the vaccination that they actually received.

All participants with protocol deviations are summarized in **Table 9.4** and listed in **Appendix 15.2.2, Listing 1** and **Appendix 15.2.5, Listing 8**. A total of 7 participants were excluded for protocol deviations: six participants were from the Alveavax-1.2 dose groups and one participant received the control. Of these 7 participants with protocol deviations, four did not receive the full dose on vaccination and three did not have a Day 28 immunogenicity result. As mentioned above, this last point represents a change from the SAP but is included since the study was effectively ended at Day 28 except for safety data.

Table 9.4: Summary of Major Protocol Deviations – mITT Population (Protocol Number: Alvea-VAX-P00001)

Protocol violation	Low Dose N = 20	Standard N = 40	High Dose N = 20	SC Injection N = 10	Combined N = 90	Control N = 40
**Any major protocol violation***	0 (0.0%)	1 (2.5%)	4 (20.0%)	1 (1.0%)	6 (6.7)	1 (2.5%)
Full dose not administered†	0 (0.0%)	1 (2.5%)	2 (10.0%)	1 (10.0%)	4 (4.4%)	0 (0.0%)
Day 28 clinical evaluation not performed	0 (0.0%)	0 (0.0%)	2 (10.0%)	0 (0.0%)	2 (2.2%)	1 (2.5%)

*These participants were all excluded from the PP population. Note: A participant may have had more than one violation.

†The volume of vaccination administered was not recorded and these participants likely received a partial dose.

Data source: **Appendix 15.2.2, Listing 1**.

9.3 DEMOGRAPHIC AND OTHER BASELINE CHARACTERISTICS

Details of participant demographic and baseline data are shown in **Table 9.5** and **Section 13.1, Table 2.1**, and are listed by center and treatment group in **Appendix 15.2.4, Listing 2**.

9.3.1 Demographics and Baseline Disease Characteristics

Among the mITT population, the treatment groups were similar with respect to participant sex, age, race, height, body weight, and BMI (**Table 9.5**).

Table 9.5: Participant Demography and Baseline Characteristics – mITT Population (Protocol Number: Alvea-VAX-P00001)

Participant data	Low Dose N = 20	Standard N = 40	High Dose N = 20	SC Injection N = 10	Combined N = 90	Control N = 40
**Gender n (%)**						
Male	11 (55.0%)	26 (65.0%)	10 (50.0%)	4 (40.0%)	51 (56.7%)	25 (62.5%)
Female	9 (45.0%)	14 (35.0%)	10 (50.0%)	6 (60.0%)	39 (43.3%)	15 (37.5%)
**Age (y)**						
Mean (SD)	35.5 (13.30)	32.2 (11.00)	31.0 (9.19)	34.2 (11.83)	32.9 (11.21)	33.3 (11.82)
Median	34.5	29.5	31.0	36.5	31.0	29.5
Range	19.0 – 61.0	18.0 – 58.0	19.0 – 57.0	21.0 – 57.0	18.0 – 61.0	19.0 – 60.0
**Race n (%)**						
Black African	16 (80.0%)	39 (97.5%)	18 (90.0%)	8 (80.0%)	81 (90.0%)	37 (92.5%)
Mixed race-Colored	1 (5.0%)	0 (0.0)	0 (0.0)	0 (0.0)	1 (1.1%)	0 (0.0)
Southern African-Colored	3 (15.0%)	1 (2.5%)	2 (10.0%)	2 (20.0%)	8 (8.9%)	3 (7.5%)
**Height (cm)**						
Mean (SD)	165.3 (9.36)	166.3 (7.84)	165.1 (8.21)	165.4 (8.65)	165.7 (8.24)	165.7 (9.31)
Median	167.5	166.5	161.9	164.2	165.9	167.5
Range	145.5 – 176.2	147.0 – 182.4	146.4 – 182.0	155.0 – 178.4	145.5 – 182.4	145.0 – 181.0
**Weight (kg)**						
Mean (SD)	68.2 (11.67)	65.1 (10.69)	65.6 (11.13)	60.8 (11.14)	65.4 (11.06)	64.7 (12.14)
Median	68.1	67.3	62.4	57.2	65.9	61.6
Range	48.9 – 86.0	43.1 – 87.0	46.3 – 85.8	45.3 – 79.9	43.1 – 87.0	42.4 – 90.0
**BMI (kg/m^2)**						
Mean (SD)	25.0 (4.19)	23.6 (4.07)	24.1 (4.03)	22.5 (5.30)	23.9 (4.23)	23.6 (3.97)
Median	24.9	23.0	22.6	20.9	23.2	22.6
Range	19.1 – 32.0	17.7 – 31.6	19.8 – 32.9	17.5 – 31.6	17.5 – 32.9	18.1 – 31.5

Data source: Appendix 15.2.4, Listing 2.

9.3.2 Medical History and Concurrent Illness

Details of medical history are shown in Section 13.1, Table 2.7 and **Appendix 15.2.4, Listing 7**. The majority of participants reported no medical history. The low and high dose groups both reported medical history in 35% of participants, whereas the other groups reported medical history in less than 35%. Among all Alveavax-1.2 dose groups combined (N = 90), vascular disorders (hypertension, 6 [6.7%]), respiratory/thoracic disorders (7 [7.8%]),

and musculoskeletal disorders (5 [5.6%]) were the most frequently reported. Most other conditions were only present in one or two participants per group.

9.3.3 Prior and Concomitant Treatments

Details of prior and concomitant medication are shown in **Section 13.1, Table 2.3** and **Appendix 15.2.4, Listing 3**. Concomitant medication included a range of both prescription and over-the-counter medication. The most common concomitant medication was analgesics; taken by 32 (35.6%) participants among all Alveavax-1.2 treatment groups combined (N = 90) and by 11 (27.5%) among the control group (N = 40).

9.4 MEASUREMENTS OF TREATMENT COMPLIANCE

Details of individual participants' compliance with the study medication are shown in **Appendix 15.2.5, Listing 8**. A total of 4 of the 90 (4.4%) participants in the Alveavax-1.2 dose groups combined did not receive their full dose of either ID or SC Alveavax-1.2 (**Table 9.4**). Two patients were in the high dose Alveavax-1.2 group, with one participant each in the standard or SC injection groups. All participants in the control group received their single IM injection of Janssen Ad26.COV2.S.

9.5 EXTENT OF EXPOSURE

The volume of vaccination administered was not recorded and only whether participants received the full dose. All 20 were administered in the low dose Alveavax-1.2 group, and all were administered in the standard dose group except for one participant (103/025), who did not receive their full single ID injection of 2 mg Alveavax-1.2.

In the Alveavax-1.2 high dose group, one participant (101/022) did not fully receive their first or fourth ID 2 mg injection, and another participant (101/036) in the high dose group did not fully receive the third of the four 2 mg Alveavax-1.2 ID injections. One participant (103/017) from the SC injection group did not receive their single SC injection of 8 mg Alveavax-1.2 and discontinued from the study.

The participants in the standard and high dose groups were excluded from the PP population but included in the mITT or Safety populations as they all had been administered a dose of the investigational product (Safety population), and in addition, experienced at least one post-baseline immunogenicity readout (mITT population). The participant in the SC injection group was excluded from both the mITT and the PP population.

10 SAFETY EVALUATION

The safety analyses included collection of adverse events throughout the study, a laboratory screen, vital signs, and tables of symptom severity resulting from vaccination.

10.1 ADVERSE EVENTS

TEAEs throughout the study are summarized in Table 10.1 and Section 13.2.1, Table 4, and all AEs are listed by participant in **Appendix 15.2.7, Listings 12–14**. Section 13.2.1, Table 4.2, which summarizes related AEs, was not in the SAP but was included because there were a large number of events classed as probably or possibly related to the vaccination.

10.1.1 Brief Summary of Adverse Events

All AEs among the Safety population are listed by participant in **Appendix 15.2.7, Listing 12**. All AEs are listed by participant and characterized by severity in **Appendix 15.2.7, Listing 13**. If a participant reported the same AE more than once, it counted as a single participant AE.

Table 10.1: Overview of TEAEs – Safety Population (Protocol Number: Alvea-VAX-P00001)

	Low Dose N = 20	Standard N = 40	High Dose N = 20	SC Injection N = 10	Combined N = 90	Control N = 40
**Number (%) of Participants Reporting any TEAE***	**n=8 (40.0%) [30]**	**n=21 (52.5%) [97]**	**n=16 (80.0%) [94]**	**n=7 (70.0%) [33]**	**n=52 (57.8%) [254]**	**n=29 (72.5%) [82]**
**Any Serious TEAE**	0	0	1 (5.0%)	0	1 (1.1%)	0
**Any Treatment Related TEAE**	6 (30.0%)	16 (40.0%)	14 (70.0%)	7 (70.0%)	43 (47.8%)	19 (47.5%)
**Any Treatment Related Serious TEAE**	0	0	0	0	0	0
**TEAEs Resulting in Withdrawal**	0	0	0	0	0	0
**TEAEs Resulting in Death**	0	0	0	0	0	0

*The number in square brackets [x] is the number of events recorded. If a participant reported the same event more than once it counts as a single participant event

Data source: Section 13.2.1, Table 4; Appendix 15.2.7, Listing 12 and Appendix 15.2.7, Listing 13.

10.1.2 Related Adverse Events

AEs that were considered by the Investigator to be possibly or probably related to the study medication are shown by system organ class and preferred term frequency in **Section 13.2.1, Table 4.2**.

10.1.3 Categorization of All Adverse Events

All TEAEs are categorized by system organ class and preferred term in **Section 13.2.1, Table 4.1** and **Section 13.2.1, Table 4.2** and listed by participant in **Appendix 15.2.7, Listing 13**.

10.2 ANALYSIS OF DEATHS, OTHER SERIOUS ADVERSE EVENTS, AND OTHER CLINICALLY MEANINGFUL ADVERSE EVENTS

10.2.1 Deaths, Other Serious Adverse Events, Discontinuation due to Adverse Events and Other Adverse Events of Special Interest

One participant (103/032) recorded three serious adverse events **(Section 13.2.1, Table 4.3)**. This participant had a severe lower respiratory infection which resulted in hospitalization (refer to SAE form [Alvea-VAX-P0001_103-032_SAE 01_Updated follow up report.pdf] in **Appendix 15.3)**. The event started on 10 October 2022 and resolved by 17 October, with treatment including amoxicillin-clavulanate. The event was not related to treatment with the study drug. The same participant also had fecaloma, which resolved, and pregnancy, recorded as a serious adverse event and ongoing at the end of the study.

There were no fatal adverse events and no AESIs during the study. All AEs by maximum severity are listed in **Section 13.2.1, Table 4.3** and all SAEs are listed by participant in **Appendix 15.2.7, Listing 14**.

10.3 CLINICAL LABORATORY EVALUATION

10.3.1 Individual Laboratory Measurements by Participant and Abnormal Laboratory Values

The safety analyses included a laboratory screen, vital signs, and tables of symptom severity resulting from vaccination.

Individual laboratory measurements by participant are provided in **Appendix 15.2.8, Listing 17**.

10.3.2 Evaluation of Laboratory Values

10.3.2.1 Laboratory values over time

10.3.2.1.1 Hematology

The hematology data were similar between baseline and Day 7 for each treatment group (**Section 13.2.3, Table 8**). **Section 13.2.3, Table 8.1** indicates that some patients had a

substantial reduction in platelet counts between baseline and Day 7, but **Section 13.2.3, Table 8.2** shows that the majority of participants were within the normal range.

10.3.2.1.2 Clinical chemistry

The clinical chemistry findings were similar between baseline and Day 7 for each treatment group (**Section 13.2.3, Table 7**). A small number of participants across the study groups had raised liver function tests above normal (ALT, N = 7; or AST, N = 10) at baseline (**Section 13.2.3, Table 7.2**) but at Day 7, there was no indication that there was any further increase following treatment.

10.3.2.2 Individual participant changes in laboratory values

See **Appendix 15.2.8, Listing 17**.

10.3.2.3 Individual clinically meaningful laboratory abnormalities

See **Appendix 15.2.8, Listing 17**.

10.4 VITAL SIGNS, PHYSICAL EXAMINATIONS, AND OTHER OBSERVATIONS RELATED TO SAFETY

10.4.1 Vital Signs

Vital signs are listed by participant in **Appendix 15.2.4, Listing 6**. Vital signs were recorded at baseline, at 30 minutes post-dose and at Day 7. There was no indication of any marked changes following vaccination.

10.4.2 Abnormal Physical Examination Findings

Abnormal Physical Examination Findings are listed by participant in **Appendix 15.2.4, Listing 5**.

10.4.3 Other Observations Related to Safety

Section 13.2.3, Tables 4.4–4.19 summarize data from the participants' diary cards from the day of vaccination to Day 6 post-vaccination. These recorded the severity of signs and symptoms possibly associated with the vaccination. Most recorded only mild to moderate signs and symptoms which readily resolved. Pain (**Section 13.2.3, Table 4.4**) was mild to moderate and lasted up to Day 6 in a small number of participants (mild, N = 2; or moderate, N = 2). Moderate to severe fatigue (**Section 13.2.3, Table 4.13**) was experienced by a subset of participants in the high dose group (moderate = 2; or severe, N = 1) at Day 5 and Day 6.

The measurement of body temperature (**Section 13.2.3, Table 4.19**) by participants appeared unreliable as recorded temperatures of 35°C and below would seem unlikely.

10.5 SAFETY RESULTS SUMMARY

- A total of three SAEs were recorded during the study in a single participant (a severe lower respiratory infection requiring hospitalization, a fecaloma which resolved, and a pregnancy which was ongoing at the end of the study). None started within 7 days of receiving the study vaccination.
- There were no AESIs or deaths during the study.
- The hematology data were similar between baseline and Study Day 7, with the majority of participants within the normal range at Day 7.
- A small number of participants had raised liver function tests above normal (ALT, N = 7; or AST, N = 10) at baseline but there was no indication of any further increase following treatment.
- There were no significant changes in any vital signs between baseline and Day 7, following vaccination.
- From Day 1 (vaccination) to Day 6 post-vaccination, most participants recorded only mild to moderate signs and symptoms which readily resolved.
- Mild to moderate pain lasted up to Day 6 in a small number of participants (mild, N = 2; or moderate, N = 2), and moderate to severe fatigue was experienced by a subset of participants in the high dose group (moderate, N = 2; or severe, N = 1) at Day 5 and Day 6.
- Participants continued to be monitored for safety for up to 6 months after completion of all study vaccinations.

11 IMMUNOGENICITY AND EFFICACY EVALUATIONS

11.1 IMMUNOGENICITY AND EFFICACY RESULTS

The final immunogenicity and efficacy analyses were amended from what was planned in version 5.0 of the protocol. The measures of cellular immunogenicity were all omitted, except for the GMT of serum/plasma anti-SARS-CoV-2 BA.2 antibody and anti-nucleocapsid antibody, with sufficient data to form any analysis and conclusions. Also, the main immunogenicity and efficacy endpoints were expressed as the half-maximal response (EC_{50}) instead of the half-maximal inhibition (IC_{50}), as described in version 5.0 of the protocol and final SAP. The immunogenicity and efficacy findings that were completed are presented in **Table 11.1**; **Section 13.3, Tables 3.0.1–3.5** and **Appendix 15.2.6, Listings 9–11**.

11.1.1 Immunogenicity Endpoints

In the protocol and final SAP, the main immunogenicity endpoint was the change in GMT from baseline to all subsequent post-dose assessments of anti-SARS-CoV-2 BA.2 antibody. As noted above, the study was halted after 6 months, and planned measurements were not carried out.

The fold changes in GMT of anti-SARS-CoV-2 BA.2 antibody are shown for the PP population in **Table 11.1**. Only the Day 28 data was complete for most of the treatment groups. The increase in GMT from baseline data to Day 28 was approximately 1 for all the study groups. In addition, data for the control group showed an increase of 164.6 in the GMT of anti-SARS-CoV-2 BA.2 antibody and a fold increase of 1.31.

Table 11.1: Change from Baseline of ELISA BA.2 Antibody Titer (EC_{50}) – Per Protocol Population

Parameters	Low Dose N = 20	Standard N = 40	High Dose N = 20	SC Injection N = 10	Combined N = 90	Control N = 40
**n**	11	20	0	0	31	21
**Change in GMT at Day 7***	-5.15	-64.52			-43.90	138.04
**N**	12	21	0	0	33	20
**Change in GMT at Day 14***	48.13	-43.06			-11.69	326.91
**N**	18	35	14	8	75	37
**Change in GMT at Day 28***	-19.02	-36.62	-22.01	46.52	-23.47	164.60
**N**	18	35	14	8	75	37
**Fold Change in GMT at Day 28**	0.97	0.93	0.96	1.06	0.96	1.31

Only participants with both a baseline value and a value at the respective visits are included in the calculation of change from baseline.

Data source: Section 13.3, Table 3.0.1.

The change from baseline can only be calculated for participants who had both a baseline and Day 28 titer. **Section 13.3, Table 2.2.1** shows that not all participants had a baseline value.

11.1.2 Efficacy Endpoints

The efficacy analyses are shown in **Section 13.3, Tables 3.0.1–3.5**. **Section 13.3, Table 3.1.1** is a repeat of the immunogenicity analysis, presented in **Table 11.1**, but with the mITT population. The addition of two participants to the low dose group increased the change seen but, overall, there was still no marked difference in the fold change of GMT between baseline and Day 28.

Section 13.3, Table 3.2.1 shows the change in GMT of the anti-nucleocapsid protein antibody, for which there were sufficient data to present. As for main immunogenicity analysis, the efficacy analysis there was a minimal increase in GMT, compared with baseline, for the Alveavax-1.2 and control groups.

11.2 RESULTS OF STATISTICAL ISSUES ENCOUNTERED DURING THE ANALYSIS

11.2.1 Adjustments for Covariates

Not relevant to this study.

11.2.2 Handling of Withdrawals, Discontinuations or Missing Data

No imputation of missing data was performed for this study. Refer to **Section 8.7.1.1** or the SAP (provided in **Appendix 15.1.9**) for further details on the handling of missing data.

11.2.3 Interim Analyses and Data Monitoring

Interim review of safety findings occurred during the study. Interim summary of immune response may have occurred periodically through the study for Phase 2 planning. No modification to this study occurred based on these analyses.

11.2.4 Multicenter Studies

Tests of homogeneity across centers were not relevant to this study.

11.2.5 Multiple Comparisons/Multiplicity

No corrections for multiple testing were planned as the analyses were used only to inform Phase 2 study design.

11.2.6 Use of an "Efficacy Subset" of Participants

The PP population was used for the main immunogenicity analysis. Protocol violations were recorded during the study and were used to determine whether participants should be included in the PP population.

11.2.7 Examination of Subgroups

The recording of all categories of AEs were assessed in the Safety population. The main immunogenicity endpoints were analyzed in the PP population, with other endpoints of immunogenicity and efficacy analyzed in the mITT population.

11.2.8 Tabulation of Individual Response Data

Individual response data are shown in **Appendix 15.2.6**.

11.3 IMMUNOGENICITY AND EFFICACY RESULTS SUMMARY

- The increase in GMT of anti-SARS-CoV-2 BA.2 antibody from baseline to Day 28 in the PP population was approximately 1-fold in each Alveavax-1.2 treatment group.
- Similarly, a minimal increase, of approximately 1-fold, in the GMT of anti-SARS-CoV-2 BA.2 antibody and anti-nucleocapsid antibody was observed in the mITT population between baseline and Day 28.
- A limited response was also observed in the control group, with the Janssen Ad26.COV2 vaccine (1.31-fold in both PP and mITT populations).
- Immunology data before Day 28 were not complete because the laboratory analysis of samples was terminated at the later timepoints in order to reduce costs.

12 DISCUSSION AND OVERALL CONCLUSIONS

12.1 DISCUSSION

This first-in-human, randomized, dose-finding study (South African National Clinical Trials Registry Identifier: DOH-27-062022-5157 / ClinicalTrials.gov Identifier: NCT05844202) found that the Alveavax-1.2 vaccine was generally safe and well tolerated among all treatment groups, with no SAEs relating to treatment. ZyCoV-D, a technologically comparable plasmid DNA vaccine, did not show any serious adverse events causally related to the vaccine in their phase III trial **[35]**.

The strengths of this study included the controlled vaccination enrolment to closely monitor and ensure participant safety, the multicenter and multi-arm study design, and the large population for a Phase 1 study. The benefits to the participants included receipt of a potentially efficacious Omicron-optimized COVID-19 booster vaccine, access to extensive diagnostic testing and medical check-ups, and contribution to research of COVID-19 during a global pandemic.

Protocol amendments were made between versions 4.0 and 5.0 due to the lack of a relevant humoral immune response data at Day 28 in anti-SARS-CoV-2 BA.2 antibodies in any of the study cohorts; the main immunogenicity endpoint. The neutralizing antibody responses in a subset of samples confirmed this observation. The Sponsor did not expect to find anything different by analyzing samples on different time points or with different testing methods. Therefore, the Sponsor recommended stopping any further tests (i.e., neutralizing antibody assays, cellular immunogenicity assessments, binding antibody tests) at other timepoints.

In addition, the Sponsor recommended ending the study for participants after 6 months instead of after 12 months follow-up. According to the FDA and WHO guidance, serious and other medically attended AEs in all study participants shall be observed for at least 6 months after completion of all study vaccinations **[36,37]**. Given the lack of immune response, a follow-up for assessing the immune response after 12 months would not add scientific value.

As for the Alveavax-1.2 treatment groups, the control comparator group did not show a marked increase in GMT from baseline data to Day 28. Since the control, the Janssen Ad26.COV2 vaccine, was a known effective vaccine, it is possible that there were limitations in the design and conduct of the study, whereby the baseline antibody titers may have been too high to show an increase after treatment. Further research on the expected titration levels of a booster vaccine could be warranted; however, the Sponsor decided to end the study after 6 months instead of 12. Further explanations for the lack of an observed immunogenic response at Day 28 could be that either the study vaccines were not stored correctly and lost potency, or that participant samples were not taken or stored correctly, giving false results.

The current immunogenicity and efficacy analysis of the comparison of GMT values meant that there were no other statistical analyses conducted, such as measurement of SD, median, or range, as can be used to summarize continuous variables. Calculations of change from baseline are often done by individual participant, and thereafter determining the mean, SD, median and range. This was not possible in this case, with the log data used to calculate GMT. Therefore, there is only a single statistic of the change in GM from baseline. Further study limitations were that the study population was only from South Africa, and that they were a healthy population only that, in particular, excluded HIV patients.

12.2 CONCLUSIONS

The Alveavax-1.2 vaccine was generally safe and well tolerated in all treatment groups; however, the lack of notable increase in the GMT of anti-SARS-CoV-2 BA.2 and anti-nucleocapsid antibodies led to the Sponsor terminating the study after 6 months instead of 12. The immunogenicity and efficacy analyses were effectively ended at Study Day 28. In conclusion, the Sponsor did not see a safety risk in ending the study earlier nor a scientific value in any additional testing. Participants continued to be monitored for safety for up to 6 months after completion of all study vaccinations.

13 TABLES

13.1 DEMOGRAPHIC DATA

Table 1: Participant Disposition

	Low Dose N = 20	Standard N = 40	High Dose N = 20	SC Injection N= 10	Combined N = 90	Control N = 40
**Study Completed (mITT Population)**						
**n**	18	37	17	9	81	39
**Completed**	18 (90.0%)	37 (92.5%)	17 (85.0%)	9 (90.0%)	81 (90.0%)	39 (97.5%)
**Reason for Non-Completion**						
**n**	2	3	3	1	9	1
**Lost to follow-up**	2 (10.0%)	2 (5.0%)	3 (15.0%)	1 (10.0%)	8 (8.9%)	1 (2.5%)
**Withdrawal by participant**	0	1 (2.5%)	0	0	1 (1.1%)	0
**Per Protocol Population**						
**n**	18	36	15	9	78	38
**Completed**	18 (90.0%)	36 (90.0%)	15 (75.0%)	9 (90.0%)	78 (86.7%)	38 (95.0%)

Program V_eos Date: 26MAY2023 Unique Number: 8852

This table only includes participants who were randomized. Screening failures are included in the appropriate Listing only.

The mITT and Safety populations are identical

Participants who did not have the full dose at Vaccination or who had no Day 28 results are excluded from the Per Protocol population

Two participants were recorded by the Investigator as being withdrawn by the Physicians decision. Examination of the details suggests that they should have been regarded as Lost to Follow Up

Table 2.1: Patient Demography and Baseline Characteristics – mITT Population

	Low Dose N= 20	Standard N= 40	High Dose N= 20	SC Injection N= 10	Combined N= 90	Control N= 40
**Participant Sex**						
**n**	20	40	20	10	90	40
**Male**	11 (55.0%)	26 (65.0%)	10 (50.0%)	4 (40.0%)	51 (56.7%)	25 (62.5%)
**Female**	9 (45.0%)	14 (35.0%)	10 (50.0%)	6 (60.0%)	39 (43.3%)	15 (37.5%)
**Participant Age (y)**						
**n**	20	40	20	10	90	40
**Mean (SD)**	35.5 (13.30)	32.2 (11.00)	31.0 (9.19)	34.2 (11.83)	32.9 (11.21)	33.3 (11.82)
**Median**	34.5	29.5	31.0	36.5	31.0	29.5
**Min-Max**	19.0 to 61.0	18.0 to 58.0	19.0 to 57.0	21.0 to 57.0	18.0 to 61.0	19.0 to 60.0
**Age Groups**						
**n**	20	40	20	10	90	40
**18 to 24**	6 (30.0%)	12 (30.0%)	7 (35.0%)	3 (30.0%)	28 (31.1%)	12 (30.0%)
**25 to 49**	10 (50.0%)	24 (60.0%)	12 (60.0%)	6 (60.0%)	52 (57.8%)	23 (57.5%)
**50 to 64**	4 (20.0%)	4 (10.0%)	1 (5.0%)	1 (10.0%)	10 (11.1%)	5 (12.5%)
**Race**						
**n**	20	40	20	10	90	40
**Black African**	16 (80.0%)	39 (97.5%)	18 (90.0%)	8 (80.0%)	81 (90.0%)	37 (92.5%)
**Mixed race-Colored**	1 (5.0%)	0	0	0	1 (1.1%)	0
**Southern African Colored**	3 (15.0%)	1 (2.5%)	2 (10.0%)	2 (20.0%)	8 (8.9%)	3 (7.5%)
**Participant Height (cm)**						
**n**	20	40	20	10	90	40
**Mean (SD)**	165.3 (9.36)	166.3 (7.84)	165.1 (8.21)	165.4 (8.65)	165.7 (8.24)	165.7 (9.31)
**Median**	167.5	166.5	161.9	164.2	165.9	167.5
**Min-Max**	145.5 to 176.2	147.0 to 182.4	146.4 to 182.0	155.0 to 178.4	145.5 to 182.4	145.0 to 181.0
**Participant Weight (kg)**						
**n**	20	40	20	10	90	40
**Mean (SD)**	68.2 (11.67)	65.1 (10.69)	65.6 (11.13)	60.8 (11.14)	65.4 (11.06)	64.7 (12.14)
**Median**	68.1	67.3	62.4	57.2	65.9	61.6
**Min-Max**	48.9 to 86.0	43.1 to 87.0	46.3 to 85.8	45.3 to 79.9	43.1 to 87.0	42.4 to 90.0

	Low Dose N= 20	Standard N= 40	High Dose N= 20	SC Injection N= 10	Combined N= 90	Control N= 40
**Participant BMI (kg/m^2)**						
**n**	20	40	20	10	90	40
**Mean (SD)**	25.0 (4.19)	23.6 (4.07)	24.1 (4.03)	22.5 (5.30)	23.9 (4.23)	23.6 (3.97)
**Median**	24.9	23.0	22.6	20.9	23.2	22.6
**Min-Max**	19.1 to 32.0	17.7 to 31.6	19.8 to 32.9	17.5 to 31.6	17.5 to 32.9	18.1 to 31.5

Program V_demog Date: 13APR2023 Unique Number: 8762

Age is calculated from the YOB and date of informed consent

Table 2.3: Concomitant Medication – mITT Population

WHO Drug Global March 2020	Low Dose N= 20	Standard N= 40	High Dose N= 20	SC Injection N= 10	Combined N= 90	Control N= 40
**Number (%) of Participants reporting any concomitant medication**	**n=10 (50.0%) [35]**	**n=20 (50.0%) [58]**	**n=13 (65.0%) [68]**	**n=7 (70.0%) [17]**	**n=50 (55.6%) [178]**	**n=24 (60.0%) [55]**
**AGENTS ACTING ON THE RENIN-ANGIOTENSIN SYSTEM**	0	1 (2.5%)	0	0	1 (1.1%)	1 (2.5%)
**Enalapril**	0	1 (2.5%)	0	0	1 (1.1%)	1 (2.5%)
**ANALGESICS**	7 (35.0%)	13 (32.5%)	7 (35.0%)	4 (40.0%)	31 (34.4%)	11 (27.5%)
**Benylin four flu**	0	1 (2.5%)	0	0	1 (1.1%)	0
**Calpol**	0	1 (2.5%)	0	0	1 (1.1%)	0
**Compral**	0	0	1 (5.0%)	0	1 (1.1%)	1 (2.5%)
**Corenza cold and flu**	0	0	1 (5.0%)	0	1 (1.1%)	0
**Disprin**	1 (5.0%)	1 (2.5%)	2 (10.0%)	0	4 (4.4%)	0
**Flustat**	0	0	0	0	0	2 (5.0%)
**Grandpa**	0	3 (7.5%)	1 (5.0%)	0	4 (4.4%)	1 (2.5%)
**Med Lemon**	0	2 (5.0%)	0	0	2 (2.2%)	0
**Mybulen**	0	0	1 (5.0%)	0	1 (1.1%)	0
**Painamol**	0	0	0	0	0	2 (5.0%)
**Panado**	2 (10.0%)	7 (17.5%)	3 (15.0%)	2 (20.0%)	14 (15.6%)	4 (10.0%)
**Paracetamol**	4 (20.0%)	5 (12.5%)	2 (10.0%)	1 (10.0%)	12 (13.3%)	3 (7.5%)
**Perfalgan**	0	0	1 (5.0%)	0	1 (1.1%)	0
**Sinucon**	0	1 (2.5%)	0	0	1 (1.1%)	0
**Sinuend**	1 (5.0%)	0	0	0	1 (1.1%)	0
**Sinutab Extra strength**	0	1 (2.5%)	0	1 (10.0%)	2 (2.2%)	0
**Tramadol**	1 (5.0%)	0	2 (10.0%)	1 (10.0%)	4 (4.4%)	0
**Vicks Medinite**	0	0	0	0	0	1 (2.5%)
**ANTHELMINTICS**	0	1 (2.5%)	0	0	1 (1.1%)	0
**Mebendazole**	0	1 (2.5%)	0	0	1 (1.1%)	0
**ANTI-PARKINSON DRUGS**	0	0	1 (5.0%)	0	1 (1.1%)	0
**Orphenadrine**	0	0	1 (5.0%)	0	1 (1.1%)	0
**ANTIANEMIC PREPARATIONS**	0	0	1 (5.0%)	0	1 (1.1%)	0
**Ferrous Sulphate**	0	0	1 (5.0%)	0	1 (1.1%)	0
**Folic Acid**	0	0	1 (5.0%)	0	1 (1.1%)	0

WHO Drug Global March 2020	Low Dose N= 20	Standard N= 40	High Dose N= 20	SC Injection N= 10	Combined N= 90	Control N= 40
**Number (%) of Participants reporting any concomitant medication**	**n=10 (50.0%) [35]**	**n=20 (50.0%) [58]**	**n=13 (65.0%) [68]**	**n=7 (70.0%) [17]**	**n=50 (55.6%) [178]**	**n=24 (60.0%) [55]**
**ANTIBACTERIALS FOR SYSTEMIC USE**	2 (10.0%)	4 (10.0%)	3 (15.0%)	2 (20.0%)	11 (12.2%)	5 (12.5%)
**Amoxicillin**	0	2 (5.0%)	2 (10.0%)	0	4 (4.4%)	0
**Amoxil**	1 (5.0%)	2 (5.0%)	1 (5.0%)	1 (10.0%)	5 (5.6%)	3 (7.5%)
**Augmentin**	0	0	1 (5.0%)	0	1 (1.1%)	0
**Azithromycin**	1 (5.0%)	0	1 (5.0%)	0	2 (2.2%)	2 (5.0%)
**Ceftriaxone**	1 (5.0%)	0	0	0	1 (1.1%)	0
**Ciproflaxacin**	0	0	0	1 (10.0%)	1 (1.1%)	0
**Co Amoxiclav**	0	0	2 (10.0%)	0	2 (2.2%)	0
**Metronidazole**	1 (5.0%)	1 (2.5%)	0	0	2 (2.2%)	0
**Zithromax**	0	0	0	0	0	1 (2.5%)
**ANTIDIARRHEALS-INTESTINAL ANTIINFLAMMATORY/ANTIINFECTIVE AGENTS**	1 (5.0%)	0	1 (5.0%)	0	2 (2.2%)	1 (2.5%)
**Adco-Loperamide**	0	0	1 (5.0%)	0	1 (1.1%)	0
**Loperamide**	1 (5.0%)	0	0	0	1 (1.1%)	1 (2.5%)
**ANTIHISTAMINES FOR SYSTEMIC USE**	2 (10.0%)	0	2 (10.0%)	1 (10.0%)	5 (5.6%)	3 (7.5%)
**Allergex**	2 (10.0%)	0	1 (5.0%)	1 (10.0%)	4 (4.4%)	1 (2.5%)
**Biocort**	0	0	1 (5.0%)	0	1 (1.1%)	0
**Chloropyramine**	0	0	0	0	0	1 (2.5%)
**Chlorpheniramine maleate**	0	0	0	0	0	1 (2.5%)
**ANTIINFLAMMATORY AND ANTIRHEUMATIC PRODUCTS**	1 (5.0%)	2 (5.0%)	2 (10.0%)	1 (10.0%)	6 (6.7%)	2 (5.0%)
**Brufen**	1 (5.0%)	2 (5.0%)	0	1 (10.0%)	4 (4.4%)	1 (2.5%)
**Ibuprofen**	0	0	2 (10.0%)	0	2 (2.2%)	0
**Nurofen**	0	0	0	0	0	1 (2.5%)
**ANTITHROMBOTIC AGENTS**	0	0	0	0	0	1 (2.5%)
**Aspirin**	0	0	0	0	0	1 (2.5%)
**ANTIVIRALS FOR SYSTEMIC USE**	0	0	1 (5.0%)	0	1 (1.1%)	0

WHO Drug Global March 2020	Low Dose N= 20	Standard N= 40	High Dose N= 20	SC Injection N= 10	Combined N= 90	Control N= 40
**Number (%) of Participants reporting any concomitant medication**	**n=10 (50.0%) [35]**	**n=20 (50.0%) [58]**	**n=13 (65.0%) [68]**	**n=7 (70.0%) [17]**	**n=50 (55.6%) [178]**	**n=24 (60.0%) [55]**
Tenemine	0	0	1 (5.0%)	0	1 (1.1%)	0
BLOOD SUBSTITUTES AND PERFUSION SOLUTIONS	0	0	1 (5.0%)	0	1 (1.1%)	0
Lactated ringer's	0	0	1 (5.0%)	0	1 (1.1%)	0
CALCIUM CHANNEL BLOCKERS	0	0	0	1 (10.0%)	1 (1.1%)	1 (2.5%)
Nifedipine	0	0	0	1 (10.0%)	1 (1.1%)	1 (2.5%)
CORTICOSTEROIDS-DERMATOLOGICAL PREPARATIONS	1 (5.0%)	0	1 (5.0%)	0	2 (2.2%)	0
Advantan	0	0	1 (5.0%)	0	1 (1.1%)	0
Persivate	1 (5.0%)	0	0	0	1 (1.1%)	0
COUGH AND COLD PREPARATIONS	0	2 (5.0%)	0	0	2 (2.2%)	1 (2.5%)
Clear cough syrup	0	0	0	0	0	1 (2.5%)
Vicks Acta Plus	0	1 (2.5%)	0	0	1 (1.1%)	0
Vicks Cough	0	1 (2.5%)	0	0	1 (1.1%)	0
DIURETICS	1 (5.0%)	2 (5.0%)	0	2 (20.0%)	5 (5.6%)	2 (5.0%)
Hydrochlorothiazide	1 (5.0%	1 (2.5%)	0	1 (10.0%)	3 (3.3%)	1 (2.5%)
Ridaq	0	1 (2.5%)	0	1 (10.0%)	2 (2.2%)	1 (2.5%)
DRUGS FOR ACID RELATED DISORDERS	0	0	4 (20.0%)	0	4 (4.4%)	1 (2.5%)
Gaviscon	0	0	0	0	0	1 (2.5%)
Gelacid	0	0	1 (5.0%)	0	1 (1.1%)	0
Lansoloc	0	0	1 (5.0%)	0	1 (1.1%)	0
Omeprazid	0	0	1 (5.0%)	0	1 (1.1%)	0
Omeprazole	0	0	2 (10.0%)	0	2 (2.2%)	0
Pantaloc	0	0	1 (5.0%)	0	1 (1.1%)	0
Roznal otc	0	0	0	0	0	1 (2.5%)
DRUGS FOR CONSTIPATION	0	0	1 (5.0%)	0	1 (1.1%)	0
Adco Fosenema	0	0	1 (5.0%)	0	1 (1.1%)	0
Lacson	0	0	1 (5.0%)	0	1 (1.1%)	0

WHO Drug Global March 2020	Low Dose N= 20	Standard N= 40	High Dose N= 20	SC Injection N= 10	Combined N= 90	Control N= 40
**Number (%) of Participants reporting any concomitant medication**	**n=10 (50.0%) [35]**	**n=20 (50.0%) [58]**	**n=13 (65.0%) [68]**	**n=7 (70.0%) [17]**	**n=50 (55.6%) [178]**	**n=24 (60.0%) [55]**
**DRUGS FOR FUNCTIONAL GASTROINTESTINAL DISORDERS**	1 (5.0%)	0	0	1 (10.0%)	2 (2.2%)	1 (2.5%)
**Adco contromet**	0	0	0	0	0	1 (2.5%)
**Buscopan**	1 (5.0%)	0	0	1 (10.0%)	2 (2.2%)	0
**DRUGS FOR OBSTRUCTIVE AIRWAY DISEASES**	1 (5.0%)	2 (5.0%)	0	0	3 (3.3%)	0
**Alcophyllex**	0	2 (5.0%)	0	0	2 (2.2%)	0
**Asthavent (NDI)**	1 (5.0%)	0	0	0	1 (1.1%)	0
**DRUGS USED IN DIABETES**	1 (5.0%)	0	0	0	1 (1.1%)	0
**Glimepiride**	1 (5.0%)	0	0	0	1 (1.1%)	0
**Metformin**	1 (5.0%)	0	0	0	1 (1.1%)	0
**EMOLLIENTS AND PROTECTIVES**	0	0	1 (5.0%)	0	1 (1.1%)	0
**Aqueous**	0	0	1 (5.0%)	0	1 (1.1%)	0
**Emulsifying Ointment BP**	0	0	1 (5.0%)	0	1 (1.1%)	0
**LIPID MODIFYING AGENTS**	1 (5.0%)	0	0	0	1 (1.1%)	0
**Simvastatin**	1 (5.0%)	0	0	0	1 (1.1%)	0
**MINERAL SUPPLEMENTS**	0	0	1 (5.0%)	0	1 (1.1%)	0
**Calcium**	0	0	1 (5.0%)	0	1 (1.1%)	0
**Normal saline**	0	0	1 (5.0%)	0	1 (1.1%)	0
**NASAL PREPARATIONS**	0	2 (5.0%)	0	1 (10.0%)	3 (3.3%)	0
**ILIADIN**	0	1 (2.5%)	0	0	1 (1.1%)	0
**Sinutab nasal spray**	0	1 (2.5%)	0	1 (10.0%)	2 (2.2%)	0
**OPHTHALMOLOGICALS**	0	0	0	0	0	1 (2.5%)
**refresh tears**	0	0	0	0	0	1 (2.5%)
**OTHER GYNECOLOGICALS**	1 (5.0%)	0	0	0	1 (1.1%)	0
**mirena**	1 (5.0%)	0	0	0	1 (1.1%)	0
**PSYCHOANALEPTICS**	0	1 (2.5%)	1 (5.0%)	0	2 (2.2%)	0
**Fluoxetine**	0	0	1 (5.0%)	0	1 (1.1%)	0
**Flutex**	0	1 (2.5%)	0	0	1 (1.1%)	0
**PSYCHOLEPTICS**	0	0	1 (5.0%)	0	1 (1.1%)	0
**Olanzapine**	0	0	1 (5.0%)	0	1 (1.1%)	0

WHO Drug Global March 2020	Low Dose N= 20	Standard N= 40	High Dose N= 20	SC Injection N= 10	Combined N= 90	Control N= 40
**Number (%) of Participants reporting any concomitant medication**	**n=10 (50.0%) [35]**	**n=20 (50.0%) [58]**	**n=13 (65.0%) [68]**	**n=7 (70.0%) [17]**	**n=50 (55.6%) [178]**	**n=24 (60.0%) [55]**
**SEX HORMONES AND MODULATORS OF THE GENITAL SYSTEM**	4 (20.0%)	10 (25.0%)	8 (40.0%)	3 (30.0%)	25 (27.8%)	9 (22.5%)
**Depo-Provera**	0	3 (7.5%)	1 (5.0%)	1 (10.0%)	5 (5.5%)	1 (2.5%)
**Implanon**	1 (5.0%)	2 (5.0%)	1 (5.0%)	0	4 (4.4%)	2 (5.0%)
**Medroxyprogesterone acetate**	1 (5.0%)	1 (2.5%)	0	0	2 (2.2%)	2 (5.0%)
**Microval**	0	1 (2.5%)	0	0	1 (1.1%)	0
**Nexplanon**	0	1 (2.5%)	0	0	1 (1.1%)	0
**Norethisterone Enanthate**	1 (5.0%)	0	0	0	1 (1.1%)	2 (5.0%)
**Nur-isterate**	1 (5.0%)	1 (2.5%)	3 (15.0%)	2 (20.0%)	7 (7.7%)	0
**Oralcon**	0	0	1 (5.0%)	0	1 (1.1%)	0
**Petogen**	0	0	2 (10.0%)	0	2 (2.2%)	3 (7.5%)
**Triphasil**	0	2 (5.0%)	1 (5.0%)	0	3 (3.3%)	0
**STOMATOLOGICAL PREPARATIONS**	0	0	1 (5.0%)	0	1 (1.1%)	0
**Flagyl**	0	0	1 (5.0%)	0	1 (1.1%)	0
**THROAT PREPARATIONS**	0	0	0	0	0	1 (2.5%)
**Betadine sore throat gargle**	0	0	0	0	0	1 (2.5%)
**TOPICAL PRODUCTS FOR JOINT AND MUSCULAR PAIN**	0	1 (2.5%)	1 (5.0%)	0	2 (2.2%)	1 (2.5%)
**Deep Heat**	0	1 (2.5%)	0	0	1 (1.1%)	0
**Diclofenac**	0	0	1 (5.0%)	0	1 (1.1%)	0
**Rub Rub**	0	0	0	0	0	1 (2.5%)
**VITAMINS**	1 (5.0%)	0	2 (10.0%)	0	3 (3.3%)	0
**Med-lemon**	0	0	1 (5.0%)	0	1 (1.1%)	1 (2.5%)
**Multivitamin**	0	0	1 (5.0%)	0	1 (1.1%)	0
**Zypol**	1 (5.0%)	0	1 (5.0%)	0	2 (2.2%)	0

Program V_conmed Date: 22MAY2023 Unique Number: 8851

Medication which was ongoing during the study or stopped less than five days before vaccination is included as Concomitant Medication

The number in square brackets [x] is the number of individual occurrences of a Concomitant Medication record

Table 2.4: COVID-19 Vaccination History – mITT Population

	Low Dose N= 20	Standard N= 40	High Dose N= 20	SC Injection N= 10	Combined N= 90	Control N= 40
**COVID-19 Vaccination**						
**n**	20	40	20	10	90	40
**Yes**	20 (100%)	40 (100%)	20 (100%)	10 (100%)	90 (100%)	40 (100%)
**Janssen Vaccine Administered**						
**n**	20	40	20	10	90	40
**Yes**	20 (100%)	40 (100%)	20 (100%)	10 (100%)	90 (100%)	40 (100%)
**Days Before Informed Consent**						
**n**	20	40	20	10	90	40
**Mean (SD)**	247.3 (85.4)	262.4 (110.1)	283.2 (113.7)	278.1 (83.1)	265.4 (102.4)	264.0 (77.2)
**Median**	245.0	251.0	305.0	294.0	266.5	270.5
**Min-Max**	70.0 to 393.0	64.0 to 685.0	70.0 to 491.0	93.0 to 380.0	64.0 to 685.0	85.0 to 508.0

Program V_vhist Date: 13APR2023 Unique Number: 8765

Table 2.5: Physical Examination at Baseline – mITT Population

	Low Dose N= 20	Standard N= 40	High Dose N= 20	SC Injection N= 10	Combined N= 90	Control N= 40
**Skin Abnormal**						
**n**	1	0	1	1	3	2
**Not Clinically Significant**	1 (5.0%)		1 (5.0%)	1 (10.0%)	3 (3.3%)	2 (5.0%)
**Chest Abnormal**						
**n**	0	0	0	0	0	0
**Respiratory System Abnormal**						
**n**	2	0	0	0	2	0
**Not Clinically Significant**	2 (10.0%)				2 (2.2%)	
**Genitourinary System Abnormal**						
**n**	0	0	0	0	0	0
**Neurologic System Abnormal**						
**n**	0	0	0	0	0	0
**Psychiatric System Abnormal**						
**n**	0	0	0	0	0	0
**Neck Abnormal**						
**n**	0	0	0	0	0	0
**Musculoskeletal System Abnormal**						
**n**	0	1	0	0	1	2
**Not Clinically Significant**		1 (2.5%)			1 (1.1%)	2 (5.0%)
**Lymphatic System Abnormal**						
**n**	0	1	0	0	1	0
**Not Clinically Significant**		1 (2.5%)			1 (1.1%)	
**Gastrointestinal (abdomen) Abnormal**						
**n**	1	2	1	1	5	3

	Low Dose N= 20	Standard N= 40	High Dose N= 20	SC Injection N= 10	Combined N= 90	Control N= 40
**Not Clinically Significant**	1 (5.0%)	2 (5.0%)	1 (5.0%)	1 (10.0%)	5 (5.6%)	3 (7.5%)
**Eyes Abnormal**						
**n**	0	0	0	0	0	2
**Not Clinically Significant**						2 (5.0%)
**Ears nose mouth and throat Abnormal**						
**n**	1	0	0	1	2	0
**Not Clinically Significant**	1 (5.0%)			1 (10.0%)	2 (2.2%)	
**Cardiovascular System Abnormal**						
**n**	1	0	0	0	1	0
**Not Clinically Significant**	1 (5.0%)				1 (1.1%)	
**Other Systems Abnormal**						
**n**	0	2	0	0	2	1
**Not Clinically Significant**		2 (5.0%)			2 (2.2%)	1 (2.5%)

Program V_phys Date: 13APR2023 Unique Number: 8766

Only abnormal findings are included in the table

Table 2.6.1: Vital Signs Pre-Dose on Vaccination Day – mITT Population

	Low Dose N= 20	Standard N= 40	High Dose N= 20	SC Injection N= 10	Combined N= 90	Control N= 40
**Systolic Blood Pressure (mmHg)**						
**n**	20	40	20	10	90	40
**Mean (SD)**	125.1 (8.61)	125.1 (8.95)	119.4 (8.67)	119.5 (10.99)	123.2 (9.30)	124.4 (11.82)
**Median**	126.0	127.0	120.5	120.5	124.0	124.0
**Min-Max**	102.0 to 137.0	105.0 to 144.0	106.0 to 135.0	105.0 to 139.0	102.0 to 144.0	105.0 to 148.0
**Diastolic Blood Pressure (mmHg)**						
**n**	20	40	20	10	90	40
**Mean (SD)**	78.3 (7.69)	79.9 (8.15)	77.6 (5.48)	79.6 (8.78)	79.0 (7.54)	79.9 (7.42)
**Median**	78.5	81.5	75.0	81.0	79.0	80.5
**Min-Max**	65.0 to 89.0	59.0 to 94.0	72.0 to 89.0	62.0 to 89.0	59.0 to 94.0	62.0 to 92.0
**Body Temperature (C)**						
**n**	20	40	20	10	90	40
**Mean (SD)**	36.3 (0.31)	36.4 (0.45)	36.2 (0.44)	36.5 (0.30)	36.3 (0.41)	36.3 (0.42)
**Median**	36.3	36.5	36.3	36.3	36.4	36.4
**Min-Max**	35.6 to 36.8	35.4 to 37.2	35.2 to 37.0	36.1 to 37.0	35.2 to 37.2	35.0 to 37.2
**Heart Rate (BPM)**						
**n**	20	40	20	10	90	40
**Mean (SD)**	75.2 (11.45)	71.5 (10.32)	74.9 (12.19)	82.0 (8.74)	74.2 (11.16)	75.8 (11.43)
**Median**	75.5	73.0	76.5	82.5	74.5	76.0
**Min-Max**	53.0 to 95.0	52.0 to 96.0	53.0 to 99.0	67.0 to 95.0	52.0 to 99.0	59.0 to 99.0

Program V_signs Date: 13APR2023 Unique Number: 8767

Table 2.6.2: Vital Signs 30 Minutes Post Dose on Vaccination Day – mITT Population

	Low Dose N= 20	Standard N= 40	High Dose N= 20	SC Injection N= 10	Combined N= 90	Control N= 40
**Systolic Blood Pressure (mmHg)**						
**n**	20	39	20	10	89	40
**Mean (SD)**	122.6 (9.40)	124.2 (10.18)	121.4 (8.49)	119.4 (13.38)	122.6 (10.02)	124.2 (14.52)
**Median**	121.5	123.0	119.5	115.0	121.0	124.0
**Min-Max**	107.0 to 141.0	109.0 to 163.0	103.0 to 135.0	104.0 to 143.0	103.0 to 163.0	101.0 to 156.0
**Diastolic Blood Pressure (mmHg)**						
**n**	20	39	20	10	89	40
**Mean (SD)**	78.7 (7.86)	81.4 (8.01)	79.3 (4.93)	79.1 (10.92)	80.0 (7.74)	79.2 (9.40)
**Median**	76.0	82.0	80.0	76.0	80.0	78.5
**Min-Max**	62.0 to 93.0	67.0 to 109.0	72.0 to 91.0	63.0 to 94.0	62.0 to 109.0	60.0 to 101.0
**Body Temperature (C)**						
**n**	20	39	20	10	89	40
**Mean (SD)**	36.4 (0.29)	36.4 (0.37)	36.4 (0.38)	36.6 (0.37)	36.4 (0.36)	36.4 (0.35)
**Median**	36.5	36.4	36.4	36.7	36.4	36.5
**Min-Max**	35.8 to 36.9	35.5 to 37.1	35.7 to 37.0	36.2 to 37.2	35.5 to 37.2	35.5 to 37.0
**Heart Rate (BPM)**						
**n**	20	39	20	10	89	40
**Mean (SD)**	76.8 (10.29)	71.3 (10.30)	69.6 (9.62)	79.1 (11.67)	73.0 (10.68)	74.4 (9.69)
**Median**	79.0	70.0	68.5	81.5	73.0	74.0
**Min-Max**	58.0 to 100.0	54.0 to 89.0	54.0 to 89.0	61.0 to 99.0	54.0 to 100.0	56.0 to 96.0

Program V_signs Date: 13APR2023 Unique Number: 8768

Table 2.7: Medical History Classified by System Organ Class and Preferred Term – Safety Population

	Low Dose N= 20	Standard N= 40	High Dose N= 20	SC Injection N= 10	Combined N= 90	Control N= 40
**Number (%) of Participants**	**n=7 (35.0%) [17]**	**n=7 (17.5%) [8]**	**n=7 (35.0%) [11]**	**n=3 (30.0%) [5]**	**n=24 (26.7%) [41]**	**n=7 (17.5%) [8]**
**Participants reporting a single history**	2 (10.0)	6 (15.0)	6 (30.0)	2 (20.0)	16 (17.8)	6 (15.0)
**Participants reporting more than one history**	5 (25.0)	1 (2.5)	1 (5.0)	1 (10.0)	8 (8.9)	1 (2.5)
**VASCULAR DISORDERS**	1 (5.0%)	2 (5.0%)	1 (5.0%)	2 (20.0%)	6 (6.7%)	2 (5.0%)
**Hypertension**	1 (5.0%)	2 (5.0%)	1 (5.0%)	2 (20.0%)	6 (6.7%)	2 (5.0%)
**RESPIRATORY-THORACIC AND MEDIASTINAL DISORDERS**	3 (15.0%)	1 (2.5%)	2 (10.0%)	1 (10.0%)	7 (7.8%)	0
**Allergic sinusitis**	1 (5.0%)	1 (2.5%)	0	1 (10.0%)	3 (3.3%)	0
**Asthma**	2 (10.0%)	0	0	0	2 (2.2%)	0
**Epistaxis**	0	0	1 (5.0%)	0	1 (1.1%)	0
**Rhinorrhea**	0	0	1 (5.0%)	0	1 (1.1%)	0
**SURGICAL AND MEDICAL PROCEDURES**	0	0	0	1 (10.0%)	1 (1.1%)	0
**Caesarean section**	0	0	0	1 (10.0%)	1 (1.1%)	0
**Sterilization**	0	0	0	1 (10.0%)	1 (1.1%)	0
**BLOOD AND LYMPHATIC SYSTEM DISORDERS**	0	1 (2.5%)	0	0	1 (1.1%)	0
**Neutropenia**	0	1 (2.5%)	0	0	1 (1.1%)	0
**EYE DISORDERS**	0	0	0	0	0	2 (5.0%)
**Conjunctivitis allergic**	0	0	0	0	0	1 (2.5%)
**Strabismus**	0	0	0	0	0	1 (2.5%)
**GASTROINTESTINAL DISORDERS**	1 (5.0%)	0	1 (5.0%)	0	2 (2.2%)	0
**Abdominal pain lower**	1 (5.0%)	0	0	0	1 (1.1%)	0
**Gastroesophageal reflux disease**	0	0	1 (5.0%)	0	1 (1.1%)	0
**HEPATOBILIARY DISORDERS**	0	0	1 (5.0%)	0	1 (1.1%)	0
**Hepatitis alcoholic**	0	0	1 (5.0%)	0	1 (1.1%)	0
**IMMUNE SYSTEM DISORDERS**	0	1 (2.5%)	1 (5.0%)	0	2 (2.2%)	0
**Food allergy**	0	1 (2.5%)	1 (5.0%)	0	2 (2.2%)	0

	Low Dose N= 20	Standard N= 40	High Dose N= 20	SC Injection N= 10	Combined N= 90	Control N= 40
**Number (%) of Participants**	**n=7 (35.0%) [17]**	**n=7 (17.5%) [8]**	**n=7 (35.0%) [11]**	**n=3 (30.0%) [5]**	**n=24 (26.7%) [41]**	**n=7 (17.5%) [8]**
**INFECTIONS AND INFESTATIONS**	1 (5.0%)	0	0	0	1 (1.1%)	1 (2.5%)
**Acarodermatitis**	0	0	0	0	0	1 (2.5%)
**Pneumonia**	1 (5.0%)	0	0	0	1 (1.1%)	0
**INJURY- POISONING AND PROCEDURAL COMPLICATIONS**	0	0	0	0	0	2 (5.0%)
**Contusion**	0	0	0	0	0	1 (2.5%)
**Joint injury**	0	0	0	0	0	1 (2.5%)
**INVESTIGATIONS**	0	1 (2.5%)	0	0	1 (1.1%)	0
**Blood pressure increased**	0	1 (2.5%)	0	0	1 (1.1%)	0
**METABOLISM AND NUTRITION DISORDERS**	1 (5.0%)	0	1 (5.0%)	0	2 (2.2%)	0
**Diabetes mellitus**	1 (5.0%)	0	0	0	1 (1.1%)	0
**Hypercholesterolemia**	1 (5.0%)	0	0	0	1 (1.1%)	0
**Obesity**	0	0	1 (5.0%)	0	1 (1.1%)	0
**MUSCULOSKELETAL AND CONNECTIVE TISSUE DISORDERS**	3 (15.0%)	1 (2.5%)	1 (5.0%)	0	5 (5.6%)	1 (2.5%)
**Arthralgia**	1 (5.0%)	0	0	0	1 (1.1%)	1 (2.5%)
**Arthritis**	2 (10.0%)	0	0	0	2 (2.2%)	0
**Myalgia**	2 (10.0%)	0	1 (5.0%)	0	3 (3.3%)	0
**Osteoarthritis**	0	0	1 (5.0%)	0	1 (1.1%)	0
**Synovial cyst**	0	1 (2.5%)	0	0	1 (1.1%)	0
**NERVOUS SYSTEM DISORDERS**	2 (10.0%)	0	0	0	2 (2.2%)	0
**Headache**	2 (10.0%)	0	0	0	2 (2.2%)	0
**PSYCHIATRIC DISORDERS**	0	0	2 (10.0%)	0	2 (2.2%)	0
**Depression**	0	0	1 (5.0%)	0	1 (1.1%)	0
**Schizophrenia**	0	0	1 (5.0%)	0	1 (1.1%)	0
**SKIN AND SUBCUTANEOUS TISSUE DISORDERS**	2 (10.0%)	0	0	0	2 (2.2%)	0
**Eczema**	1 (5.0%)	0	0	0	1 (1.1%)	0
**Rash**	1 (5.0%)	0	0	0	1 (1.1%)	0

SAS Program: V_mhist Date: 13APR2023 Unique Number: 8769

	Low Dose N= 20	Standard N= 40	High Dose N= 20	SC Injection N= 10	Combined N= 90	Control N= 40
**Number (%) of Participants**	**n=7 (35.0%) [17]**	**n=7 (17.5%) [8]**	**n=7 (35.0%) [11]**	**n=3 (30.0%) [5]**	**n=24 (26.7%) [41]**	**n=7 (17.5%) [8]**

The number in square brackets [x] is the number of individual Medical History reports

Where a participant reports two or more Medical Histories in the same SOC group both events are recorded but only one record is counted in the SOC group

Only Medical History which was ongoing at the start of the study is included

Table 2.8: Administration of Vaccines – mITT Population

	Low Dose N= 20	Standard N= 40	High Dose N= 20	SC Injection N= 10	Combined N= 90	Control N= 40
**Intradermal Injection**						
**n**	20	40	20	0	80	0
**0.5 mg Alveavax-v1.2**	20 (100%)	0	0		20 (22.2%)	
**2 mg Alveavax-v1.2**	0	40 (100%)	0		40 (44.4%)	
**8mg Alveavax-v1.2**	0	0	20 (100%)		20 (22.2%)	
**Intramuscular Injection**						
**n**	0	0	0	0	0	40
**Janssen Ad26.COV2.S**						40 (100%)
**Sub-cutaneous Injection**						
**n**	0	0	0	10	10	0
**8mg Alveavax-v1.2**				10 (100%)	10 (11.1%)	
**Number of Vaccinations**						
**n**	20	40	80	10	150	40
**1**	20 (100%)	40 (100%)	20 (25.0%)	10 (100%)	90 (60.0%)	40 (100%)
**2**	0	0	20 (25.0%)	0	20 (13.3%)	0
**3**	0	0	20 (25.0%)	0	20 (13.3%)	0
**4**	0	0	20 (25.0%)	0	20 (13.3%)	0
**Was the Full Dose Administered**						
**n**	20	40	80	10	150	40
**Yes**	20 (100%)	39 (97.5%)	77 (96.3%)	9 (90.0%)	145 (96.7%)	40 (100%)
**No**	0	1 (2.5%)	3 (3.8%)	1 (10.0%)	5 (3.3%)	0

Program V_vacc Date: 13APR2023 Unique Number: 8770

The denominator for the number of vaccinations and the number administered a full dose is the total number of vaccinations not the number of participants

Table 2.9: Size of the Bleb following Intradermal Vaccination – mITT Population

	Low Dose N= 20	Standard N= 40	High Dose Vacc 1 N= 20	High Dose Vacc 2 N= 20	High Dose Vacc 3 N= 20	High Dose Vacc 4 N= 20
**Size of Bleb (mm) when it Lasted 20 seconds or more**						
**n**	19	39	20	20	20	20
**Mean (SD)**	6.9 (2.44)	10.7 (2.66)	10.7 (2.30)	11.1 (2.10)	10.7 (2.25)	10.6 (1.70)
**Median**	8.0	10.0	12.0	12.0	11.0	10.0
**Min-Max**	3.0 to 10.0	7.0 to 16.0	6.0 to 14.0	7.0 to 14.0	7.0 to 14.0	7.0 to 14.0

Program V_bleb Date: 13APR2023 Unique Number: 8771

Note: only two participants had blebs lasting less than 20 seconds. They are not included in the table.

13.2 SAFETY DATA

13.2.1 Displays of Adverse Events

Table 4: Adverse Events Summary – Safety Population

	Low Dose N= 20	Standard N= 40	High Dose N= 20	SC Injection N= 10	Combined N= 90	Control N= 40
**Number (%) of Participants Reporting any TEAE**	**n=8 (40.0%) [30]**	**n=21 (52.5%) [97]**	**n=16 (80.0%) [94]**	**n=7 (70.0%) [33]**	**n=52 (57.8%) [254]**	**n=29 (72.5%) [82]**
**Type and Outcome of Adverse Events**						
**Subjects Reporting any Serious TEAE**	0	0	1 (5.0%)	0	1 (1.1%)	0
**Subjects Reporting a Treatment Related TEAE**	6 (30.0%)	16 (40.0%)	14 (70.0%)	7 (70.0%)	43 (47.8%)	19 (47.5%)
**Subjects Reporting a Treatment Related Serious TEAE**	0	0	0	0	0	0
**TEAEs Resulting in Withdrawal**	0	0	0	0	0	0
**TEAEs which were ultimately Fatal**	0	0	0	0	0	0

SAS Program: V_ae02 Date: 17APR2023 Unique Number: 8810

If a participant reports the same event more than once it counts as a single participant event

TEAE = Treatment Emergent Adverse Events

The number in square brackets [x] is the number of events recorded

Table 4.1: All Adverse Events Classified by System Organ Class and Preferred Term – Safety Population

By System Organ Class and Preferred Term	Low Dose N= 20	Standard N= 40	High Dose N= 20	SC Injection N= 10	Combined N= 90	Control N= 40
**Number (%) of Participants Reporting any TEAE**	**n=8 (40.0%) [30]**	**n=21 (52.5%) [97]**	**n=16 (80.0%) [94]**	**n=7 (70.0%) [33]**	**n=52 (57.8%) [254]**	**n=29 (72.5%) [82]**
**GENERAL DISORDERS AND ADMINISTRATION SITE CONDITIONS**	5 (25.0%) [13]	10 (25.0%) [44]	13 (65.0%) [57]	6 (60.0%) [14]	34 (37.8%) [128]	16 (40.0%) [40]
**Injection site reaction**	4 (20.0%) [10]	8 (20.0%) [31]	12 (60.0%) [48]	5 (50.0%) [9]	29 (32.2%) [98]	16 (40.0%) [35]
**Chills**	0	2 (5.0%) [3]	3 (15.0%) [3]	2 (20.0%) [2]	7 (7.8%) [8]	2 (5.0%) [2]
**Fatigue**	1 (5.0%) [1]	6 (15.0%) [8]	5 (25.0%) [5]	2 (20.0%) [2]	14 (15.6%) [16]	1 (2.5%) [1]
**Influenza like illness**	0	0	0	1 (10.0%) [1]	1 (1.1%) [1]	1 (2.5%) [1]
**Malaise**	1 (5.0%) [1]	2 (5.0%) [2]	0	0	3 (3.3%) [3]	0
**Pyrexia**	1 (5.0%) [1]	0	1 (5.0%) [1]	0	2 (2.2%) [2]	1 (2.5%) [1]
**GASTROINTESTINAL DISORDERS**	2 (10.0%) [4]	4 (10.0%) [5]	7 (35.0%) [11]	4 (40.0%) [6]	17 (18.9%) [26]	5 (12.5%) [6]
**Diarrhea**	1 (5.0%) [1]	1 (2.5%) [2]	3 (15.0%) [3]	2 (20.0%) [3]	7 (7.8%) [9]	2 (5.0%) [3]
**Abdominal pain**	0	1 (2.5%) [1]	1 (5.0%) [1]	1 (10.0%) [1]	3 (3.3%) [3]	0
**Nausea**	2 (10.0%) [2]	1 (2.5%) [1]	2 (10.0%) [2]	1 (10.0%) [1]	6 (6.7%) [6]	2 (5.0%) [2]
**Vomiting**	1 (5.0%) [1]	0	1 (5.0%) [1]	1 (10.0%) [1]	3 (3.3%) [3]	0
**Constipation**	0	0	1 (5.0%) [1]	0	1 (1.1%) [1]	0
**Dental caries**	0	1 (2.5%) [1]	0	0	1 (1.1%) [1]	0
**Dyspepsia**	0	0	0	0	0	1 (2.5%) [1]
**Fecaloma**	0	0	1 (5.0%) [1]	0	1 (1.1%) [1]	0

By System Organ Class and Preferred Term	Low Dose N= 20	Standard N= 40	High Dose N= 20	SC Injection N= 10	Combined N= 90	Control N= 40
**Number (%) of Participants Reporting any TEAE**	**n=8 (40.0%) [30]**	**n=21 (52.5%) [97]**	**n=16 (80.0%) [94]**	**n=7 (70.0%) [33]**	**n=52 (57.8%) [254]**	**n=29 (72.5%) [82]**
**Gastroesophageal reflux disease**	0	0	1 (5.0%) [1]	0	1 (1.1%) [1]	0
**Peptic ulcer**	0	0	1 (5.0%) [1]	0	1 (1.1%) [1]	0
**NERVOUS SYSTEM DISORDERS**	4 (20.0%) [4]	12 (30.0%) [22]	6 (30.0%) [6]	4 (40.0%) [6]	26 (28.9%) [38]	7 (17.5%) [9]
**Headache**	3 (15.0%) [3]	11 (27.5%) [19]	6 (30.0%) [6]	4 (40.0%) [6]	24 (26.7%) [34]	7 (17.5%) [9]
**Dizziness**	1 (5.0%) [1]	3 (7.5%) [3]	0	0	4 (4.4%) [4]	0
**INFECTIONS AND INFESTATIONS**	3 (15.0%) [3]	8 (20.0%) [13]	5 (25.0%) [7]	3 (30.0%) [3]	19 (21.1%) [26]	10 (25.0%) [11]
**Gastroenteritis**	0	1 (2.5%) [1]	1 (5.0%) [1]	1 (10.0%) [1]	3 (3.3%) [3]	0
**Upper respiratory tract infection**	0	2 (5.0%) [3]	1 (5.0%) [1]	1 (10.0%) [1]	4 (4.4%) [5]	0
**Urinary tract infection**	0	0	0	1 (10.0%) [1]	1 (1.1%) [1]	0
**COVID-19**	0	1 (2.5%) [1]	1 (5.0%) [1]	0	2 (2.2%) [2]	0
**Influenza**	1 (5.0%) [1]	3 (7.5%) [6]	1 (5.0%) [1]	0	5 (5.6%) [8]	3 (7.5%) [3]
**Lower respiratory tract infection**	0	0	1 (5.0%) [1]	0	1 (1.1%) [1]	0
**Nasopharyngitis**	1 (5.0%) [1]	0	1 (5.0%) [1]	0	2 (2.2%) [2]	1 (2.5%) [1]
**Orchitis**	0	0	0	0	0	1 (2.5%) [1]
**Otitis media**	0	0	1 (5.0%) [1]	0	1 (1.1%) [1]	0
**Pharyngitis**	0	1 (2.5%) [1]	0	0	1 (1.1%) [1]	2 (5.0%) [2]
**Rhinitis**	1 (5.0%) [1]	0	0	0	1 (1.1%) [1]	0
**Sinusitis**	0	0	0	0	0	2 (5.0%) [2]
**Subcutaneous abscess**	0	1 (2.5%) [1]	0	0	1 (1.1%) [1]	0
**Tonsillitis**	0	0	0	0	0	2 (5.0%) [2]

By System Organ Class and Preferred Term	Low Dose N= 20	Standard N= 40	High Dose N= 20	SC Injection N= 10	Combined N= 90	Control N= 40
**Number (%) of Participants Reporting any TEAE**	**n=8 (40.0%) [30]**	**n=21 (52.5%) [97]**	**n=16 (80.0%) [94]**	**n=7 (70.0%) [33]**	**n=52 (57.8%) [254]**	**n=29 (72.5%) [82]**
**INJURY- POISONING AND PROCEDURAL COMPLICATIONS**	1 (5.0%) [1]	1 (2.5%) [1]	1 (5.0%) [1]	1 (10.0%) [1]	4 (4.4%) [4]	1 (2.5%) [1]
**Injury**	0	0	0	1 (10.0%) [1]	1 (1.1%) [1]	0
**Face injury**	0	1 (2.5%) [1]	0	0	1 (1.1%) [1]	0
**Joint injury**	0	0	0	0	0	1 (2.5%) [1]
**Ligament sprain**	0	0	1 (5.0%) [1]	0	1 (1.1%) [1]	0
**Thermal burn**	1 (5.0%) [1]	0	0	0	1 (1.1%) [1]	0
**MUSCULOSKELETAL AND CONNECTIVE TISSUE DISORDERS**	1 (5.0%) [2]	5 (12.5%) [7]	4 (20.0%) [6]	1 (10.0%) [3]	11 (12.2%) [18]	6 (15.0%) [9]
**Arthralgia**	1 (5.0%) [1]	1 (2.5%) [1]	2 (10.0%) [2]	1 (10.0%) [1]	5 (5.6%) [5]	4 (10.0%) [4]
**Myalgia**	1 (5.0%) [1]	4 (10.0%) [5]	3 (15.0%) [4]	1 (10.0%) [2]	9 (10.0%) [12]	5 (12.5%) [5]
**Soft tissue injury**	0	1 (2.5%) [1]	0	0	1 (1.1%) [1]	0
**BLOOD AND LYMPHATIC SYSTEM DISORDERS**	0	2 (5.0%) [2]	0	0	2 (2.2%) [2]	0
**Neutropenia**	0	2 (5.0%) [2]	0	0	2 (2.2%) [2]	0
**EAR AND LABYRINTH DISORDERS**	0	0	1 (5.0%) [1]	0	1 (1.1%) [1]	0
**Ear pain**	0	0	1 (5.0%) [1]	0	1 (1.1%) [1]	0
**IMMUNE SYSTEM DISORDERS**	1 (5.0%) [1]	0	0	0	1 (1.1%) [1]	0
**Hypersensitivity**	1 (5.0%) [1]	0	0	0	1 (1.1%) [1]	0
**INVESTIGATIONS**	0	0	1 (5.0%) [1]	0	1 (1.1%) [1]	2 (5.0%) [2]
**Aspartate aminotransferase increased**	0	0	0	0	0	1 (2.5%) [1]
**Platelet count decreased**	0	0	1 (5.0%) [1]	0	1 (1.1%) [1]	0

By System Organ Class and Preferred Term	Low Dose N= 20	Standard N= 40	High Dose N= 20	SC Injection N= 10	Combined N= 90	Control N= 40
**Number (%) of Participants Reporting any TEAE**	**n=8 (40.0%) [30]**	**n=21 (52.5%) [97]**	**n=16 (80.0%) [94]**	**n=7 (70.0%) [33]**	**n=52 (57.8%) [254]**	**n=29 (72.5%) [82]**
**White blood cell count decreased**	0	0	0	0	0	1 (2.5%) [1]
**METABOLISM AND NUTRITION DISORDERS**	0	0	1 (5.0%) [1]	0	1 (1.1%) [1]	0
**Dehydration**	0	0	1 (5.0%) [1]	0	1 (1.1%) [1]	0
**PREGNANCY-PUERPERIUM AND PERINATAL CONDITIONS**	0	0	1 (5.0%) [1]	0	1 (1.1%) [1]	0
**Pregnancy**	0	0	1 (5.0%) [1]	0	1 (1.1%) [1]	0
**RESPIRATORY-THORACIC AND MEDIASTINAL DISORDERS**	1 (5.0%) [1]	3 (7.5%) [3]	1 (5.0%) [1]	0	5 (5.6%) [5]	2 (5.0%) [2]
**Allergic sinusitis**	0	1 (2.5%) [1]	0	0	1 (1.1%) [1]	0
**Cough**	0	0	0	0	0	1 (2.5%) [1]
**Dysphonia**	1 (5.0%) [1]	0	0	0	1 (1.1%) [1]	0
**Nasal congestion**	0	1 (2.5%) [1]	0	0	1 (1.1%) [1]	0
**Oropharyngeal pain**	0	1 (2.5%) [1]	0	0	1 (1.1%) [1]	0
**Rhinitis allergic**	0	0	0	0	0	1 (2.5%) [1]
**Throat irritation**	0	0	1 (5.0%) [1]	0	1 (1.1%) [1]	0
**SKIN AND SUBCUTANEOUS TISSUE DISORDERS**	1 (5.0%) [1]	0	1 (5.0%) [1]	0	2 (2.2%) [2]	1 (2.5%) [1]
**Eczema**	0	0	1 (5.0%) [1]	0	1 (1.1%) [1]	0
**Pruritus**	1 (5.0%) [1]	0	0	0	1 (1.1%) [1]	0
**Solar dermatitis**	0	0	0	0	0	1 (2.5%) [1]
**VASCULAR DISORDERS**	0	0	0	0	0	1 (2.5%) [1]
**Hypertension**	0	0	0	0	0	1 (2.5%) [1]

SAS Program: V_ae01 Date: 13APR2023 Unique Number: 8779

By System Organ Class and Preferred Term	Low Dose N= 20	Standard N= 40	High Dose N= 20	SC Injection N= 10	Combined N= 90	Control N= 40
**Number (%) of Participants Reporting any TEAE**	**n=8 (40.0%) [30]**	**n=21 (52.5%) [97]**	**n=16 (80.0%) [94]**	**n=7 (70.0%) [33]**	**n=52 (57.8%) [254]**	**n=29 (72.5%) [82]**

The number in square brackets [x] is the number of individual occurrences of a TEAE

If a participant reports the same event more than once it counts as a single participant event

Where a participant reports two or more events in the same SOC group both events are recorded but only one record is counted in the SOC group

Table 4.2: Related Adverse Events Classified by System Organ Class and Preferred Term – Safety Population

By System Organ Class and Preferred Term	Low Dose N= 20	Standard N= 40	High Dose N= 20	SC Injection N= 10	Combined N= 90	Control N= 40
**Participants n (%) Reporting a Related TEAE**	**n=6 (30.0%) [22]**	**n=16 (40.0%) [72]**	**n=14 (70.0%) [78]**	**n=7 (70.0%) [27]**	**n=43 (47.8%) [199]**	**n=19 (47.5%) [61]**
**GENERAL DISORDERS AND ADMINISTRATION SITE CONDITIONS**	5 (25.0%) [13]	9 (22.5%) [43]	13 (65.0%) [57]	5 (50.0%) [13]	32 (35.6%) [126]	16 (40.0%) [39]
**Injection site reaction**	4 (20.0%) [10]	8 (20.0%) [31]	12 (60.0%) [48]	5 (50.0%) [9]	29 (32.2%) [98]	16 (40.0%) [35]
**Chills**	0	2 (5.0%) [3]	3 (15.0%) [3]	2 (20.0%) [2]	7 (7.8%) [8]	2 (5.0%) [2]
**Fatigue**	1 (5.0%) [1]	5 (12.5%) [7]	5 (25.0%) [5]	2 (20.0%) [2]	13 (14.4%) [15]	1 (2.5%) [1]
**Malaise**	1 (5.0%) [1]	2 (5.0%) [2]	0	0	3 (3.3%) [3]	0
**Pyrexia**	1 (5.0%) [1]	0	1 (5.0%) [1]	0	2 (2.2%) [2]	1 (2.5%) [1]
**NERVOUS SYSTEM DISORDERS**	3 (15.0%) [3]	9 (22.5%) [16]	5 (25.0%) [5]	4 (40.0%) [6]	21 (23.3%) [30]	5 (12.5%) [7]
**Headache**	3 (15.0%) [3]	7 (17.5%) [13]	5 (25.0%) [5]	4 (40.0%) [6]	19 (21.1%) [27]	5 (12.5%) [7]
**Dizziness**	0	3 (7.5%) [3]	0	0	3 (3.3%) [3]	0
**GASTROINTESTINAL DISORDERS**	2 (10.0%) [3]	3 (7.5%) [4]	3 (15.0%) [6]	3 (30.0%) [5]	11 (12.2%) [18]	3 (7.5%) [4]
**Diarrhea**	0	1 (2.5%) [2]	3 (15.0%) [3]	2 (20.0%) [3]	6 (6.7%) [8]	1 (2.5%) [2]
**Nausea**	2 (10.0%) [2]	1 (2.5%) [1]	2 (10.0%) [2]	1 (10.0%) [1]	6 (6.7%) [6]	2 (5.0%) [2]
**Vomiting**	1 (5.0%) [1]	0	1 (5.0%) [1]	1 (10.0%) [1]	3 (3.3%) [3]	0
**Abdominal pain**	0	1 (2.5%) [1]	0	0	1 (1.1%) [1]	0
**MUSCULOSKELETAL AND CONNECTIVE TISSUE DISORDERS**	1 (5.0%) [2]	4 (10.0%) [6]	4 (20.0%) [5]	1 (10.0%) [3]	10 (11.1%) [16]	6 (15.0%) [9]

By System Organ Class and Preferred Term	Low Dose N= 20	Standard N= 40	High Dose N= 20	SC Injection N= 10	Combined N= 90	Control N= 40
**Participants n (%) Reporting a Related TEAE**	**n=6 (30.0%) [22]**	**n=16 (40.0%) [72]**	**n=14 (70.0%) [78]**	**n=7 (70.0%) [27]**	**n=43 (47.8%) [199]**	**n=19 (47.5%) [61]**
**Arthralgia**	1 (5.0%) [1]	1 (2.5%) [1]	2 (10.0%) [2]	1 (10.0%) [1]	5 (5.6%) [5]	4 (10.0%) [4]
**Myalgia**	1 (5.0%) [1]	4 (10.0%) [5]	3 (15.0%) [3]	1 (10.0%) [2]	9 (10.0%) [11]	5 (12.5%) [5]
**BLOOD AND LYMPHATIC SYSTEM DISORDERS**	0	2 (5.0%) [2]	0	0	2 (2.2%) [2]	0
**Neutropenia**	0	2 (5.0%) [2]	0	0	2 (2.2%) [2]	0
**IMMUNE SYSTEM DISORDERS**	1 (5.0%) [1]	0	0	0	1 (1.1%) [1]	0
**Hypersensitivity**	1 (5.0%) [1]	0	0	0	1 (1.1%) [1]	0
**INFECTIONS AND INFESTATIONS**	0	0	2 (10.0%) [3]	0	2 (2.2%) [3]	1 (2.5%) [1]
**Gastroenteritis**	0	0	1 (5.0%) [1]	0	1 (1.1%) [1]	0
**Influenza**	0	0	1 (5.0%) [1]	0	1 (1.1%) [1]	1 (2.5%) [1]
**Nasopharyngitis**	0	0	1 (5.0%) [1]	0	1 (1.1%) [1]	0
**INVESTIGATIONS**	0	0	1 (5.0%) [1]	0	1 (1.1%) [1]	1 (2.5%) [1]
**Platelet count decreased**	0	0	1 (5.0%) [1]	0	1 (1.1%) [1]	0
**White blood cell count decreased**	0	0	0	0	0	1 (2.5%) [1]
**METABOLISM AND NUTRITION DISORDERS**	0	0	1 (5.0%) [1]	0	1 (1.1%) [1]	0
**Dehydration**	0	0	1 (5.0%) [1]	0	1 (1.1%) [1]	0
**RESPIRATORY-THORACIC AND MEDIASTINAL DISORDERS**	0	1 (2.5%) [1]	0	0	1 (1.1%) [1]	0
**Nasal congestion**	0	1 (2.5%) [1]	0	0	1 (1.1%) [1]	0

SAS Program: V_aeRel Date: 13APR2023 Unique Number: 8780

The number in square brackets [x] is the number of individual occurrences of a TEAE

If a participant reports the same event more than once it counts as a single participant event

By System Organ Class and Preferred Term	Low Dose N= 20	Standard N= 40	High Dose N= 20	SC Injection N= 10	Combined N= 90	Control N= 40
Participants n (%) Reporting a Related TEAE	n=6 (30.0%) [22]	n=16 (40.0%) [72]	n=14 (70.0%) [78]	n=7 (70.0%) [27]	n=43 (47.8%) [199]	n=19 (47.5%) [61]

Adverse Events which were Possibly, Probably or Definitely recorded as Related to treatment are included in this table

Table 4.3: Treatment Emergent Adverse Events Summary by Maximum Severity – Safety Population

By System Organ Class and Preferred Term		Low Dose N= 20	Standard N= 40	High Dose N= 20	SC Injection N= 10	Combined N= 90	Control N= 40
**Participants Reporting any TEAE**	**Severity**	**n=8 (40.0%) [30]**	**n=21 (52.5%) [97]**	**n=16 (80.0%) [94]**	**n=7 (70.0%) [33]**	**n=52 (57.8%) [254]**	**n=29 (72.5%) [82]**
**GENERAL DISORDERS AND ADMINISTRATION SITE CONDITIONS**		5 (25.0%)	10 (25.0%)	13 (65.0%)	6 (60.0%)	34 (37.8%)	16 (40.0%)
**Fatigue**	Severe	0	0	1 (5.0%)	0	1 (1.1%)	0
**Pyrexia**	Severe	1 (5.0%)	0	0	0	1 (1.1%)	0
**Chills**	Moderate	0	0	1 (5.0%)	1 (10.0%)	2 (2.2%)	0
**Fatigue**	Moderate	0	1 (2.5%)	3 (15.0%)	0	4 (4.4%)	0
**Influenza like illness**	Moderate	0	0	0	0	0	1 (2.5%)
**Injection site reaction**	Moderate	2 (10.0%)	3 (7.5%)	5 (25.0%)	1 (10.0%)	11 (12.2%)	8 (20.0%)
**Pyrexia**	Moderate	0	0	1 (5.0%)	0	1 (1.1%)	1 (2.5%)
**Chills**	Mild	0	2 (5.0%)	2 (10.0%)	1 (10.0%)	5 (5.6%)	2 (5.0%)
**Fatigue**	Mild	1 (5.0%)	5 (12.5%)	1 (5.0%)	2 (20.0%)	9 (10.0%)	1 (2.5%)
**Influenza like illness**	Mild	0	0	0	1 (10.0%)	1 (1.1%)	0
**Injection site reaction**	Mild	2 (10.0%)	5 (12.5%)	7 (35.0%)	4 (40.0%)	18 (20.0%)	8 (20.0%)
**Malaise**	Mild	1 (5.0%)	2 (5.0%)	0	0	3 (3.3%)	0
**GASTROINTESTINAL DISORDERS**		2 (10.0%)	4 (10.0%)	7 (35.0%)	4 (40.0%)	17 (18.9%)	5 (12.5%)
**Fecaloma**	Severe	0	0	1 (5.0%)	0	1 (1.1%)	0
**Constipation**	Moderate	0	0	1 (5.0%)	0	1 (1.1%)	0
**Dental caries**	Moderate	0	1 (2.5%)	0	0	1 (1.1%)	0
**Diarrhea**	Moderate	0	1 (2.5%)	0	0	1 (1.1%)	2 (5.0%)

By System Organ Class and Preferred Term		Low Dose N= 20	Standard N= 40	High Dose N= 20	SC Injection N= 10	Combined N= 90	Control N= 40
**Participants Reporting any TEAE**	**Severity**	**n=8 (40.0%) [30]**	**n=21 (52.5%) [97]**	**n=16 (80.0%) [94]**	**n=7 (70.0%) [33]**	**n=52 (57.8%) [254]**	**n=29 (72.5%) [82]**
**Gastroesophageal reflux disease**	Moderate	0	0	1 (5.0%)	0	1 (1.1%)	0
**Nausea**	Moderate	1 (5.0%)	1 (2.5%)	0	0	2 (2.2%)	0
**Peptic ulcer**	Moderate	0	0	1 (5.0%)	0	1 (1.1%)	0
**Vomiting**	Moderate	1 (5.0%)	0	0	0	1 (1.1%)	0
**Abdominal pain**	Mild	0	1 (2.5%)	1 (5.0%)	1 (10.0%)	3 (3.3%)	0
**Diarrhea**	Mild	1 (5.0%)	0	3 (15.0%)	2 (20.0%)	6 (6.7%)	0
**Dyspepsia**	Mild	0	0	0	0	0	1 (2.5%)
**Nausea**	Mild	1 (5.0%)	0	2 (10.0%)	1 (10.0%)	4 (4.4%)	2 (5.0%)
**Vomiting**	Mild	0	0	1 (5.0%)	1 (10.0%)	2 (2.2%)	0
**NERVOUS SYSTEM DISORDERS**		4 (20.0%)	12 (30.0%)	6 (30.0%)	4 (40.0%)	26 (28.9%)	7 (17.5%)
**Dizziness**	Moderate	0	1 (2.5%)	0	0	1 (1.1%)	0
**Headache**	Moderate	0	1 (2.5%)	2 (10.0%)	1 (10.0%)	4 (4.4%)	1 (2.5%)
**Dizziness**	Mild	1 (5.0%)	2 (5.0%)	0	0	3 (3.3%)	0
**Headache**	Mild	3 (15.0%)	10 (25.0%)	4 (20.0%)	3 (30.0%)	20 (22.2%)	6 (15.0%)
**INFECTIONS AND INFESTATIONS**		3 (15.0%)	8 (20.0%)	5 (25.0%)	3 (30.0%)	19 (21.1%)	10 (25.0%)
**Lower respiratory tract infection**	Severe	0	0	1 (5.0%)	0	1 (1.1%)	0
**COVID-19**	Moderate	0	1 (2.5%)	0	0	1 (1.1%)	0
**Gastroenteritis**	Moderate	0	0	1 (5.0%)	0	1 (1.1%)	0
**Orchitis**	Moderate	0	0	0	0	0	1 (2.5%)
**Otitis media**	Moderate	0	0	1 (5.0%)	0	1 (1.1%)	0
**Pharyngitis**	Moderate	0	1 (2.5%)	0	0	1 (1.1%)	2 (5.0%)

By System Organ Class and Preferred Term		Low Dose N= 20	Standard N= 40	High Dose N= 20	SC Injection N= 10	Combined N= 90	Control N= 40
**Participants Reporting any TEAE**	**Severity**	**n=8 (40.0%) [30]**	**n=21 (52.5%) [97]**	**n=16 (80.0%) [94]**	**n=7 (70.0%) [33]**	**n=52 (57.8%) [254]**	**n=29 (72.5%) [82]**
**Subcutaneous abscess**	Moderate	0	1 (2.5%)	0	0	1 (1.1%)	0
**Tonsillitis**	Moderate	0	0	0	0	0	1 (2.5%)
**Upper respiratory tract infection**	Moderate	0	2 (5.0%)	1 (5.0%)	1 (10.0%)	4 (4.4%)	0
**Urinary tract infection**	Moderate	0	0	0	1 (10.0%)	1 (1.1%)	0
**COVID-19**	Mild	0	0	1 (5.0%)	0	1 (1.1%)	0
**Gastroenteritis**	Mild	0	1 (2.5%)	0	1 (10.0%)	2 (2.2%)	0
**Influenza**	Mild	1 (5.0%)	3 (7.5%)	1 (5.0%)	0	5 (5.6%)	3 (7.5%)
**Nasopharyngitis**	Mild	1 (5.0%)	0	1 (5.0%)	0	2 (2.2%)	1 (2.5%)
**Rhinitis**	Mild	1 (5.0%)	0	0	0	1 (1.1%)	0
**Sinusitis**	Mild	0	0	0	0	0	2 (5.0%)
**Tonsillitis**	Mild	0	0	0	0	0	1 (2.5%)
**INJURY-POISONING AND PROCEDURAL COMPLICATIONS**		1 (5.0%)	1 (2.5%)	1 (5.0%)	1 (10.0%)	4 (4.4%)	1 (2.5%)
**Injury**	Moderate	0	0	0	1 (10.0%)	1 (1.1%)	0
**Ligament sprain**	Moderate	0	0	1 (5.0%)	0	1 (1.1%)	0
**Thermal burn**	Moderate	1 (5.0%)	0	0	0	1 (1.1%)	0
**Face injury**	Mild	0	1 (2.5%)	0	0	1 (1.1%)	0
**Joint injury**	Mild	0	0	0	0	0	1 (2.5%)
**MUSCULOSKELETAL AND CONNECTIVE TISSUE DISORDERS**		1 (5.0%)	5 (12.5%)	4 (20.0%)	1 (10.0%)	11 (12.2%)	6 (15.0%)
**Arthralgia**	Moderate	0	1 (2.5%)	0	0	1 (1.1%)	2 (5.0%)
**Myalgia**	Moderate	0	0	2 (10.0%)	0	2 (2.2%)	2 (5.0%)

By System Organ Class and Preferred Term		Low Dose N= 20	Standard N= 40	High Dose N= 20	SC Injection N= 10	Combined N= 90	Control N= 40
**Participants Reporting any TEAE**	**Severity**	**n=8 (40.0%) [30]**	**n=21 (52.5%) [97]**	**n=16 (80.0%) [94]**	**n=7 (70.0%) [33]**	**n=52 (57.8%) [254]**	**n=29 (72.5%) [82]**
**Arthralgia**	Mild	1 (5.0%)	0	2 (10.0%)	1 (10.0%)	4 (4.4%)	2 (5.0%)
**Myalgia**	Mild	1 (5.0%)	4 (10.0%)	1 (5.0%)	1 (10.0%)	7 (7.8%)	3 (7.5%)
**Soft tissue injury**	Mild	0	1 (2.5%)	0	0	1 (1.1%)	0
**BLOOD AND LYMPHATIC SYSTEM DISORDERS**		0	2 (5.0%)	0	0	2 (2.2%)	0
**Neutropenia**	Moderate	0	1 (2.5%)	0	0	1 (1.1%)	0
**Neutropenia**	Mild	0	1 (2.5%)	0	0	1 (1.1%)	0
**EAR AND LABYRINTH DISORDERS**		0	0	1 (5.0%)	0	1 (1.1%)	0
**Ear pain**	Moderate	0	0	1 (5.0%)	0	1 (1.1%)	0
**IMMUNE SYSTEM DISORDERS**		1 (5.0%)	0	0	0	1 (1.1%)	0
**Hypersensitivity**	Moderate	1 (5.0%)	0	0	0	1 (1.1%)	0
**INVESTIGATIONS**		0	0	1 (5.0%)	0	1 (1.1%)	2 (5.0%)
**Aspartate aminotransferase increased**	Mild	0	0	0	0	0	1 (2.5%)
**Platelet count decreased**	Mild	0	0	1 (5.0%)	0	1 (1.1%)	0
**White blood cell count decreased**	Mild	0	0	0	0	0	1 (2.5%)
**METABOLISM AND NUTRITION DISORDERS**		0	0	1 (5.0%)	0	1 (1.1%)	0
**Dehydration**	Moderate	0	0	1 (5.0%)	0	1 (1.1%)	0
**PREGNANCY-PUERPERIUM AND PERINATAL CONDITIONS**		0	0	1 (5.0%)	0	1 (1.1%)	0
**Pregnancy**	Severe	0	0	1 (5.0%)	0	1 (1.1%)	0

By System Organ Class and Preferred Term		Low Dose N= 20	Standard N= 40	High Dose N= 20	SC Injection N= 10	Combined N= 90	Control N= 40
**Participants Reporting any TEAE**	**Severity**	**n=8 (40.0%) [30]**	**n=21 (52.5%) [97]**	**n=16 (80.0%) [94]**	**n=7 (70.0%) [33]**	**n=52 (57.8%) [254]**	**n=29 (72.5%) [82]**
**RESPIRATORY-THORACIC AND MEDIASTINAL DISORDERS**		1 (5.0%)	3 (7.5%)	1 (5.0%)	0	5 (5.6%)	2 (5.0%)
**Oropharyngeal pain**	Moderate	0	1 (2.5%)	0	0	1 (1.1%)	0
**Allergic sinusitis**	Mild	0	1 (2.5%)	0	0	1 (1.1%)	0
**Cough**	Mild	0	0	0	0	0	1 (2.5%)
**Dysphonia**	Mild	1 (5.0%)	0	0	0	1 (1.1%)	0
**Nasal congestion**	Mild	0	1 (2.5%)	0	0	1 (1.1%)	0
**Rhinitis allergic**	Mild	0	0	0	0	0	1 (2.5%)
**Throat irritation**	Mild	0	0	1 (5.0%)	0	1 (1.1%)	0
**SKIN AND SUBCUTANEOUS TISSUE DISORDERS**		1 (5.0%)	0	1 (5.0%)	0	2 (2.2%)	1 (2.5%)
**Eczema**	Moderate	0	0	1 (5.0%)	0	1 (1.1%)	0
**Pruritus**	Moderate	1 (5.0%)	0	0	0	1 (1.1%)	0
**Solar dermatitis**	Mild	0	0	0	0	0	1 (2.5%)
**VASCULAR DISORDERS**		0	0	0	0	0	1 (2.5%)
**Hypertension**	Mild	0	0	0	0	0	1 (2.5%)

SAS Program: V_ae08 Date: 17APR2023 Unique Number: 8814

The number in square brackets [x] is the number of individual TEAEs

If a subject reports the same event more than once it only counts once at the greatest severity

Where a subject reports two or more events in the same SOC group both events are recorded but only one record is counted in the SOC group

13.2.2 Listings of Deaths, Other Serious and Clinically Meaningful Adverse Events

There were no fatal AEs and no AESIs during the study.

13.2.3 Data Listings (Each Participant) for Abnormal Clinically Meaningful Laboratory Values, Vital Signs, Physical Examinations and Other Observations Related to Safety

Table 4.4: Local Symptoms of Pain at the Injection Site – Safety Population

	Low Dose N = 20	Standard N= 40	High Dose N= 20	SC Injection N= 10	Combined N= 90	Control N= 40
**Pain on the Day of Injection**						
**n**	20	40	20	10	90	40
**Absent**	19 (95.0%)	33 (82.5%)	16 (80.0%)	9 (90.0%)	77 (85.6%)	23 (57.5%)
**Mild**	0	3 (7.5%)	2 (10.0%)	0	5 (5.6%)	11 (27.5%)
**Moderate**	1 (5.0%)	4 (10.0%)	2 (10.0%)	1 (10.0%)	8 (8.9%)	6 (15.0%)
**Pain on Day 1 Post Injection**						
**n**	20	40	20	10	90	40
**Absent**	19 (95.0%)	34 (85.0%)	17 (85.0%)	9 (90.0%)	79 (87.8%)	28 (70.0%)
**Mild**	0	4 (10.0%)	2 (10.0%)	0	6 (6.7%)	8 (20.0%)
**Moderate**	1 (5.0%)	2 (5.0%)	1 (5.0%)	1 (10.0%)	5 (5.6%)	3 (7.5%)
**Severe**	0	0	0	0	0	1 (2.5%)
**Pain on Day 2 Post Injection**						
**n**	20	39	20	10	89	40
**Absent**	19 (95.0%)	37 (92.5%)	19 (95.0%)	9 (90.0%)	84 (93.3%)	32 (80.0%)
**Mild**	1 (5.0%)	1 (2.5%)	0	1 (10.0%)	3 (3.3%)	5 (12.5%)
**Moderate**	0	1 (2.5%)	1 (5.0%)	0	2 (2.2%)	3 (7.5%)
**Pain on Day 3 Post Injection**						
**n**	20	40	20	10	90	40
**Absent**	19 (95.0%)	38 (95.0%)	18 (90.0%)	9 (90.0%)	84 (93.3%)	37 (92.5%)
**Mild**	1 (5.0%)	0	2 (10.0%)	0	3 (3.3%)	2 (5.0%)
**Moderate**	0	2 (5.0%)	0	1 (10.0%)	3 (3.3%)	1 (2.5%)
**Pain on Day 4 Post Injection**						

	Low Dose N = 20	Standard N= 40	High Dose N= 20	SC Injection N= 10	Combined N= 90	Control N= 40
**n**	20	40	20	10	90	40
**Absent**	20 (100%)	37 (92.5%)	20 (100%)	9 (90.0%)	86 (95.6%)	37 (92.5%)
**Mild**	0	2 (5.0%)	0	0	2 (2.2%)	2 (5.0%)
**Moderate**	0	1 (2.5%)	0	1 (10.0%)	2 (2.2%)	1 (2.5%)
**Pain on Day 5 Post Injection**						
**n**	20	40	20	10	90	40
**Absent**	19 (95.0%)	37 (92.5%)	20 (100%)	9 (90.0%)	85 (94.4%)	39 (97.5%)
**Mild**	1 (5.0%)	2 (5.0%)	0	1 (10.0%)	4 (4.4%)	0
**Moderate**	0	1 (2.5%)	0	0	1 (1.1%)	1 (2.5%)
**Pain on Day 6 Post Injection**						
**n**	20	39	20	10	89	40
**Absent**	19 (95.0%)	38 (95.0%)	20 (100%)	9 (90.0%)	86 (95.6%)	39 (97.5%)
**Mild**	1 (5.0%)	0	0	1 (10.0%)	2 (2.2%)	0
**Moderate**	0	1 (2.5%)	0	0	1 (1.1%)	1 (2.5%)

Program V_pain Date: 13APR2023 Unique Number: 8782

Symptom scores are taken from the participants diary card

Table 4.5: Local Symptoms of Tenderness at the Injection site – Safety Population

	Low Dose N = 20	Standard N= 40	High Dose N= 20	SC Injection N= 10	Combined N= 90	Control N= 40
**Tenderness on the Day of Injection**						
**n**	20	40	20	10	90	40
**Absent**	16 (80.0%)	34 (85.0%)	16 (80.0%)	8 (80.0%)	74 (82.2%)	34 (85.0%)
**Mild**	2 (10.0%)	4 (10.0%)	2 (10.0%)	2 (20.0%)	10 (11.1%)	4 (10.0%)
**Moderate**	1 (5.0%)	2 (5.0%)	2 (10.0%)	0	5 (5.6%)	2 (5.0%)
**Severe**	1 (5.0%)	0	0	0	1 (1.1%)	0
**Tenderness on Day 1 Post Injection**						
**n**	20	40	20	10	90	40
**Absent**	19 (95.0%)	40 (100%)	14 (70.0%)	9 (90.0%)	82 (91.1%)	36 (90.0%)
**Mild**	1 (5.0%)	0	4 (20.0%)	1 (10.0%)	6 (6.7%)	3 (7.5%)
**Moderate**	0	0	2 (10.0%)	0	2 (2.2%)	1 (2.5%)
**Tenderness on Day 2 Post Injection**						
**n**	20	39	20	10	89	40
**Absent**	19 (95.0%)	37 (92.5%)	15 (75.0%)	8 (80.0%)	79 (87.8%)	38 (95.0%)
**Mild**	1 (5.0%)	0	4 (20.0%)	2 (20.0%)	7 (7.8%)	2 (5.0%)
**Moderate**	0	2 (5.0%)	1 (5.0%)	0	3 (3.3%)	0
**Tenderness on Day 3 Post Injection**						
**n**	20	40	20	10	90	40
**Absent**	20 (100%)	39 (97.5%)	17 (85.0%)	9 (90.0%)	85 (94.4%)	39 (97.5%)
**Mild**	0	0	3 (15.0%)	1 (10.0%)	4 (4.4%)	1 (2.5%)
**Moderate**	0	1 (2.5%)	0	0	1 (1.1%)	0
**Tenderness on Day 4 Post Injection**						
**n**	20	40	20	10	90	40
**Absent**	19 (95.0%)	39 (97.5%)	15 (75.0%)	9 (90.0%)	82 (91.1%)	39 (97.5%)
**Mild**	1 (5.0%)	0	4 (20.0%)	1 (10.0%)	6 (6.7%)	1 (2.5%)

	Low Dose N = 20	Standard N= 40	High Dose N= 20	SC Injection N= 10	Combined N= 90	Control N= 40
**Moderate**	0	1 (2.5%)	1 (5.0%)	0	2 (2.2%)	0
**Tenderness on Day 5 Post Injection**						
**n**	20	40	20	10	90	40
**Absent**	19 (95.0%)	38 (95.0%)	20 (100%)	9 (90.0%)	86 (95.6%)	40 (100%)
**Mild**	0	2 (5.0%)	0	1 (10.0%)	3 (3.3%)	0
**Moderate**	1 (5.0%)	0	0	0	1 (1.1%)	0
**Tenderness on Day 6 Post Injection**						
**n**	20	39	20	10	89	40
**Absent**	20 (100%)	38 (95.0%)	19 (95.0%)	9 (90.0%)	86 (95.6%)	40 (100%)
**Mild**	0	1 (2.5%)	1 (5.0%)	1 (10.0%)	3 (3.3%)	0

Program V_tend Date: 13APR2023 Unique Number: 8783

Symptom scores are taken from the participants diary card

Table 4.6: Local Symptoms of Induration at the Injection site – Safety Population

	Low Dose N = 20	Standard N= 40	High Dose N= 20	SC Injection N= 10	Combined N= 90	Control N= 40
**Induration on the Day of Injection**						
**n**	20	40	20	10	90	40
**Absent**	19 (95.0%)	35 (87.5%)	18 (90.0%)	8 (80.0%)	80 (88.9%)	32 (80.0%)
**Mild**	1 (5.0%)	4 (10.0%)	1 (5.0%)	2 (20.0%)	8 (8.9%)	7 (17.5%)
**Moderate**	0	1 (2.5%)	1 (5.0%)	0	2 (2.2%)	1 (2.5%)
**Induration on Day 1 Post Injection**						
**n**	20	40	20	10	90	40
**Absent**	19 (95.0%)	35 (87.5%)	16 (80.0%)	9 (90.0%)	79 (87.8%)	34 (85.0%)
**Mild**	1 (5.0%)	4 (10.0%)	2 (10.0%)	0	7 (7.8%)	5 (12.5%)
**Moderate**	0	1 (2.5%)	2 (10.0%)	1 (10.0%)	4 (4.4%)	1 (2.5%)
**Induration on Day 2 Post Injection**						
**n**	20	39	20	10	89	40
**Absent**	20 (100%)	37 (92.5%)	17 (85.0%)	9 (90.0%)	83 (92.2%)	37 (92.5%)
**Mild**	0	2 (5.0%)	2 (10.0%)	1 (10.0%)	5 (5.6%)	3 (7.5%)
**Moderate**	0	0	1 (5.0%)	0	1 (1.1%)	0
**Induration on Day 3 Post Injection**						
**n**	20	40	20	10	90	40
**Absent**	20 (100%)	39 (97.5%)	18 (90.0%)	9 (90.0%)	86 (95.6%)	40 (100%)
**Mild**	0	0	1 (5.0%)	1 (10.0%)	2 (2.2%)	0
**Moderate**	0	1 (2.5%)	1 (5.0%)	0	2 (2.2%)	0
**Induration on Day 4 Post Injection**						
**n**	20	40	20	10	90	40
**Absent**	20 (100%)	38 (95.0%)	18 (90.0%)	9 (90.0%)	85 (94.4%)	40 (100%)
**Mild**	0	2 (5.0%)	2 (10.0%)	1 (10.0%)	5 (5.6%)	0
**Induration on Day 5 Post Injection**						
**n**	20	40	20	10	90	40

	Low Dose N = 20	Standard N= 40	High Dose N= 20	SC Injection N= 10	Combined N= 90	Control N= 40
**Absent**	20 (100%)	39 (97.5%)	19 (95.0%)	9 (90.0%)	87 (96.7%)	40 (100%)
**Mild**	0	1 (2.5%)	1 (5.0%)	1 (10.0%)	3 (3.3%)	0
**Induration on Day 6 Post Injection**						
**n**	20	39	20	10	89	40
**Absent**	20 (100%)	39 (97.5%)	19 (95.0%)	9 (90.0%)	87 (96.7%)	40 (100%)
**Mild**	0	0	1 (5.0%)	1 (10.0%)	2 (2.2%)	0

Program V_ind Date: 13APR2023 Unique Number: 8784

Symptom scores are taken from the participants diary card

Table 4.7: Local Symptoms of Itching at the Injection site – Safety Population

	Low Dose N = 20	Standard N= 40	High Dose N= 20	SC Injection N= 10	Combined N= 90	Control N= 40
**Itching on the Day of Injection**						
**n**	20	40	20	10	90	40
**Absent**	18 (90.0%)	34 (85.0%)	15 (75.0%)	8 (80.0%)	75 (83.3%)	37 (92.5%)
**Mild**	2 (10.0%)	5 (12.5%)	2 (10.0%)	2 (20.0%)	11 (12.2%)	3 (7.5%)
**Moderate**	0	1 (2.5%)	3 (15.0%)	0	4 (4.4%)	0
**Itching on Day 1 Post Injection**						
**n**	20	40	20	10	90	40
**Absent**	20 (100%)	37 (92.5%)	15 (75.0%)	8 (80.0%)	80 (88.9%)	36 (90.0%)
**Mild**	0	1 (2.5%)	2 (10.0%)	2 (20.0%)	5 (5.6%)	4 (10.0%)
**Moderate**	0	2 (5.0%)	3 (15.0%)	0	5 (5.6%)	0
**Itching on Day 2 Post Injection**						
**n**	20	39	20	10	89	40
**Absent**	20 (100%)	37 (92.5%)	13 (65.0%)	8 (80.0%)	78 (86.7%)	37 (92.5%)
**Mild**	0	1 (2.5%)	4 (20.0%)	1 (10.0%)	6 (6.7%)	3 (7.5%)
**Moderate**	0	1 (2.5%)	3 (15.0%)	1 (10.0%)	5 (5.6%)	0
**Itching on Day 3 Post Injection**						
**n**	20	40	20	10	90	40
**Absent**	20 (100%)	37 (92.5%)	14 (70.0%)	9 (90.0%)	80 (88.9%)	40 (100%)
**Mild**	0	1 (2.5%)	4 (20.0%)	0	5 (5.6%)	0
**Moderate**	0	2 (5.0%)	2 (10.0%)	1 (10.0%)	5 (5.6%)	0
**Itching on Day 4 Post Injection**						
**n**	20	40	20	10	90	40
**Absent**	19 (95.0%)	39 (97.5%)	16 (80.0%)	9 (90.0%)	83 (92.2%)	40 (100%)
**Mild**	1 (5.0%)	1 (2.5%)	3 (15.0%)	1 (10.0%)	6 (6.7%)	0
**Moderate**	0	0	1 (5.0%)	0	1 (1.1%)	0

	Low Dose N = 20	Standard N= 40	High Dose N= 20	SC Injection N= 10	Combined N= 90	Control N= 40
**Itching on Day 5 Post Injection**						
**n**	20	40	20	10	90	40
**Absent**	19 (95.0%)	38 (95.0%)	17 (85.0%)	9 (90.0%)	83 (92.2%)	40 (100%)
**Mild**	1 (5.0%)	2 (5.0%)	2 (10.0%)	1 (10.0%)	6 (6.7%)	0
**Moderate**	0	0	1 (5.0%)	0	1 (1.1%)	0
**Itching on Day 6 Post Injection**						
**n**	20	39	20	10	89	40
**Absent**	19 (95.0%)	38 (95.0%)	17 (85.0%)	9 (90.0%)	83 (92.2%)	40 (100%)
**Mild**	1 (5.0%)	1 (2.5%)	2 (10.0%)	1 (10.0%)	5 (5.6%)	0
**Moderate**	0	0	1 (5.0%)	0	1 (1.1%)	0

Program V_itch Date: 13APR2023 Unique Number: 8785

Symptom scores are taken from the participants diary card

Table 4.8: Local Symptoms of Ecchymosis at the Injection site – Safety Population

	Low Dose N = 20	Standard N= 40	High Dose N= 20	SC Injection N= 10	Combined N= 90	Control N= 40
**Ecchymosis on the Day of Injection**						
**n**	20	40	20	10	90	40
**Absent**	19 (95.0%)	37 (92.5%)	18 (90.0%)	8 (80.0%)	82 (91.1%)	39 (97.5%)
**Mild**	1 (5.0%)	2 (5.0%)	1 (5.0%)	1 (10.0%)	5 (5.6%)	0
**Moderate**	0	1 (2.5%)	1 (5.0%)	1 (10.0%)	3 (3.3%)	1 (2.5%)
**Ecchymosis on Day 1 Post Injection**						
**n**	20	40	20	10	90	40
**Absent**	19 (95.0%)	38 (95.0%)	18 (90.0%)	8 (80.0%)	83 (92.2%)	39 (97.5%)
**Mild**	1 (5.0%)	1 (2.5%)	1 (5.0%)	2 (20.0%)	5 (5.6%)	1 (2.5%)
**Moderate**	0	1 (2.5%)	1 (5.0%)	0	2 (2.2%)	0
**Ecchymosis on Day 2 Post Injection**						
**n**	20	39	20	10	89	40
**Absent**	19 (95.0%)	38 (95.0%)	17 (85.0%)	8 (80.0%)	82 (91.1%)	39 (97.5%)
**Mild**	1 (5.0%)	0	2 (10.0%)	2 (20.0%)	5 (5.6%)	1 (2.5%)
**Moderate**	0	1 (2.5%)	1 (5.0%)	0	2 (2.2%)	0
**Ecchymosis on Day 3 Post Injection**						
**n**	20	40	20	10	90	40
**Absent**	20 (100%)	39 (97.5%)	19 (95.0%)	8 (80.0%)	86 (95.6%)	40 (100%)
**Mild**	0	0	1 (5.0%)	2 (20.0%)	3 (3.3%)	0
**Moderate**	0	1 (2.5%)	0	0	1 (1.1%)	0
**Ecchymosis on Day 4 Post Injection**						
**n**	20	40	20	10	90	40
**Absent**	20 (100%)	38 (95.0%)	19 (95.0%)	8 (80.0%)	85 (94.4%)	40 (100%)
**Mild**	0	2 (5.0%)	1 (5.0%)	2 (20.0%)	5 (5.6%)	0
**Ecchymosis on Day 5 Post Injection**						
**n**	20	40	20	10	90	40

	Low Dose N = 20	Standard N= 40	High Dose N= 20	SC Injection N= 10	Combined N= 90	Control N= 40
**Absent**	19 (95.0%)	39 (97.5%)	19 (95.0%)	8 (80.0%)	85 (94.4%)	40 (100%)
**Mild**	1 (5.0%)	1 (2.5%)	0	2 (20.0%)	4 (4.4%)	0
**Moderate**	0	0	1 (5.0%)	0	1 (1.1%)	0
**Ecchymosis on Day 6 Post Injection**						
**n**	20	39	20	10	89	40
**Absent**	20 (100%)	39 (97.5%)	19 (95.0%)	9 (90.0%)	87 (96.7%)	40 (100%)
**Mild**	0	0	1 (5.0%)	1 (10.0%)	2 (2.2%)	0

Program V_echy Date: 13APR2023 Unique Number: 8786

Symptom scores are taken from the participants diary card

Table 4.9: Headache following Injection – Safety Population

	Low Dose N = 20	Standard N= 40	High Dose N= 20	SC Injection N= 10	Combined N= 90	Control N= 40
**Headache on the Day of Injection**						
**n**	20	40	20	10	90	40
**Absent**	19 (95.0%)	32 (80.0%)	17 (85.0%)	7 (70.0%)	75 (83.3%)	33 (82.5%)
**Mild**	1 (5.0%)	8 (20.0%)	3 (15.0%)	3 (30.0%)	15 (16.7%)	6 (15.0%)
**Moderate**	0	0	0	0	0	1 (2.5%)
**Headache on Day 1 Post Injection**						
**n**	20	40	20	10	90	40
**Absent**	18 (90.0%)	37 (92.5%)	17 (85.0%)	8 (80.0%)	80 (88.9%)	34 (85.0%)
**Mild**	2 (10.0%)	2 (5.0%)	3 (15.0%)	1 (10.0%)	8 (8.9%)	6 (15.0%)
**Moderate**	0	1 (2.5%)	0	1 (10.0%)	2 (2.2%)	0
**Headache on Day 2 Post Injection**						
**n**	20	39	20	10	89	40
**Absent**	18 (90.0%)	32 (80.0%)	17 (85.0%)	7 (70.0%)	74 (82.2%)	37 (92.5%)
**Mild**	2 (10.0%)	7 (17.5%)	2 (10.0%)	3 (30.0%)	14 (15.6%)	3 (7.5%)
**Moderate**	0	0	1 (5.0%)	0	1 (1.1%)	0
**Headache on Day 3 Post Injection**						
**n**	20	40	20	10	90	40
**Absent**	19 (95.0%)	35 (87.5%)	18 (90.0%)	9 (90.0%)	81 (90.0%)	38 (95.0%)
**Mild**	1 (5.0%)	5 (12.5%)	1 (5.0%)	1 (10.0%)	8 (8.9%)	2 (5.0%)
**Moderate**	0	0	1 (5.0%)	0	1 (1.1%)	0
**Headache on Day 4 Post Injection**						
**n**	20	40	20	10	90	40
**Absent**	18 (90.0%)	35 (87.5%)	16 (80.0%)	10 (100%)	79 (87.8%)	39 (97.5%)
**Mild**	2 (10.0%)	5 (12.5%)	4 (20.0%)	0	11 (12.2%)	1 (2.5%)
**Headache on Day 5 Post Injection**						
**n**	20	40	20	10	90	40
**Absent**	19 (95.0%)	35 (87.5%)	16 (80.0%)	8 (80.0%)	78 (86.7%)	37 (92.5%)
**Mild**	1 (5.0%)	5 (12.5%)	3 (15.0%)	2 (20.0%)	11 (12.2%)	3 (7.5%)

	Low Dose N = 20	Standard N= 40	High Dose N= 20	SC Injection N= 10	Combined N= 90	Control N= 40
**Moderate**	0	0	1 (5.0%)	0	1 (1.1%)	0
**Headache on Day 6 Post Injection**						
**n**	20	39	20	10	89	40
**Absent**	19 (95.0%)	36 (90.0%)	17 (85.0%)	8 (80.0%)	80 (88.9%)	38 (95.0%)
**Mild**	1 (5.0%)	3 (7.5%)	1 (5.0%)	2 (20.0%)	7 (7.8%)	2 (5.0%)
**Moderate**	0	0	2 (10.0%)	0	2 (2.2%)	0

Program V_hache Date: 13APR2023 Unique Number: 8787

Symptom scores are taken from the participants diary card

Table 4.10: Chills or Shivering following injection – Safety Population

	Low Dose N = 20	Standard N= 40	High Dose N= 20	SC Injection N= 10	Combined N= 90	Control N= 40
**Chills on the Day of Injection**						
**n**	20	40	20	10	90	40
**Absent**	20 (100%)	39 (97.5%)	19 (95.0%)	9 (90.0%)	87 (96.7%)	39 (97.5%)
**Mild**	0	1 (2.5%)	0	1 (10.0%)	2 (2.2%)	1 (2.5%)
**Moderate**	0	0	1 (5.0%)	0	1 (1.1%)	0
**Chills on Day 1 Post Injection**						
**n**	20	40	20	10	90	40
**Absent**	20 (100%)	39 (97.5%)	17 (85.0%)	9 (90.0%)	85 (94.4%)	39 (97.5%)
**Mild**	0	1 (2.5%)	3 (15.0%)	1 (10.0%)	5 (5.6%)	1 (2.5%)
**Chills on Day 2 Post Injection**						
**n**	20	39	20	10	89	40
**Absent**	20 (100%)	37 (92.5%)	18 (90.0%)	9 (90.0%)	84 (93.3%)	39 (97.5%)
**Mild**	0	2 (5.0%)	1 (5.0%)	0	3 (3.3%)	1 (2.5%)
**Moderate**	0	0	1 (5.0%)	1 (10.0%)	2 (2.2%)	0
**Chills on Day 3 Post Injection**						
**n**	20	40	20	10	90	40
**Absent**	20 (100%)	37 (92.5%)	17 (85.0%)	9 (90.0%)	83 (92.2%)	38 (95.0%)
**Mild**	0	3 (7.5%)	3 (15.0%)	1 (10.0%)	7 (7.8%)	2 (5.0%)
**Chills on Day 4 Post Injection**						
**n**	20	40	20	10	90	40
**Absent**	20 (100%)	38 (95.0%)	18 (90.0%)	8 (80.0%)	84 (93.3%)	40 (100%)
**Mild**	0	2 (5.0%)	2 (10.0%)	2 (20.0%)	6 (6.7%)	0
**Chills on Day 5 Post Injection**						
**n**	20	40	20	10	90	40
**Absent**	20 (100%)	39 (97.5%)	18 (90.0%)	10 (100%)	87 (96.7%)	40 (100%)
**Mild**	0	1 (2.5%)	2 (10.0%)	0	3 (3.3%)	0

	Low Dose N = 20	Standard N= 40	High Dose N= 20	SC Injection N= 10	Combined N= 90	Control N= 40
**Chills on Day 6 Post Injection**						
**n**	20	39	20	10	89	40
**Absent**	20 (100%)	39 (97.5%)	16 (80.0%)	10 (100%)	85 (94.4%)	40 (100%)
**Mild**	0	0	4 (20.0%)	0	4 (4.4%)	0

Program V_chills Date: 13APR2023 Unique Number: 8788

Symptom scores are taken from the participants diary card

Table 4.11: Arthralgia following injection – Safety Population

	Low Dose N= 20	Standard N= 40	High Dose N= 20	SC Injection N= 10	Combined N= 90	Control N= 40
**Arthralgia on the Day of Injection**						
**n**	20	40	20	10	90	40
**Absent**	17 (85.0%)	39 (97.5%)	18 (90.0%)	10 (100%)	84 (93.3%)	38 (95.0%)
**Mild**	2 (10.0%)	1 (2.5%)	2 (10.0%)	0	5 (5.6%)	2 (5.0%)
**Moderate**	1 (5.0%)	0	0	0	1 (1.1%)	0
**Arthralgia on Day 1 Post Injection**						
**n**	20	40	20	10	90	40
**Absent**	18 (90.0%)	38 (95.0%)	17 (85.0%)	9 (90.0%)	82 (91.1%)	34 (85.0%)
**Mild**	1 (5.0%)	2 (5.0%)	2 (10.0%)	1 (10.0%)	6 (6.7%)	5 (12.5%)
**Moderate**	0	0	0	0	0	1 (2.5%)
**Severe**	1 (5.0%)	0	1 (5.0%)	0	2 (2.2%)	0
**Arthralgia on Day 2 Post Injection**						
**n**	20	39	20	10	89	40
**Absent**	18 (90.0%)	38 (95.0%)	19 (95.0%)	8 (80.0%)	83 (92.2%)	36 (90.0%)
**Mild**	1 (5.0%)	1 (2.5%)	1 (5.0%)	2 (20.0%)	5 (5.6%)	3 (7.5%)
**Moderate**	0	0	0	0	0	1 (2.5%)
**Severe**	1 (5.0%)	0	0	0	1 (1.1%)	0
**Arthralgia on Day 3 Post Injection**						
**n**	20	40	20	10	90	40
**Absent**	19 (95.0%)	38 (95.0%)	18 (90.0%)	9 (90.0%)	84 (93.3%)	38 (95.0%)
**Mild**	0	2 (5.0%)	2 (10.0%)	1 (10.0%)	5 (5.6%)	2 (5.0%)
**Moderate**	1 (5.0%)	0	0	0	1 (1.1%)	0
**Arthralgia on Day 4 Post Injection**						
**n**	20	40	20	10	90	40
**Absent**	19 (95.0%)	38 (95.0%)	19 (95.0%)	9 (90.0%)	85 (94.4%)	39 (97.5%)
**Mild**	0	2 (5.0%)	1 (5.0%)	1 (10.0%)	4 (4.4%)	1 (2.5%)

	Low Dose N= 20	Standard N= 40	High Dose N= 20	SC Injection N= 10	Combined N= 90	Control N= 40
**Moderate**	1 (5.0%)	0	0	0	1 (1.1%)	0
**Arthralgia on Day 5 Post Injection**						
**n**	20	40	20	10	90	40
**Absent**	19 (95.0%)	39 (97.5%)	20 (100%)	9 (90.0%)	87 (96.7%)	39 (97.5%)
**Mild**	0	1 (2.5%)	0	1 (10.0%)	2 (2.2%)	0
**Moderate**	1 (5.0%)	0	0	0	1 (1.1%)	1 (2.5%)
**Arthralgia on Day 6 Post Injection**						
**n**	20	39	20	10	89	40
**Absent**	19 (95.0%)	38 (95.0%)	19 (95.0%)	9 (90.0%)	85 (94.4%)	39 (97.5%)
**Mild**	0	1 (2.5%)	1 (5.0%)	1 (10.0%)	3 (3.3%)	0
**Moderate**	1 (5.0%)	0	0	0	1 (1.1%)	1 (2.5%)

Program V_arthr Date: 13APR2023 Unique Number: 8789

Symptom scores are taken from the participants diary card

Table 4.12: Diarrhea following injection – Safety Population

	Low Dose N = 20	Standard N= 40	High Dose N= 20	SC Injection N= 10	Combined N= 90	Control N= 40
**Diarrhea on the Day of Injection**						
**n**	20	40	20	10	90	40
**Absent**	20 (100%)	38 (95.0%)	19 (95.0%)	10 (100%)	87 (96.7%)	40 (100%)
**Mild**	0	2 (5.0%)	1 (5.0%)	0	3 (3.3%)	0
**Diarrhea on Day 1 Post Injection**						
**n**	20	40	20	10	90	40
**Absent**	20 (100%)	38 (95.0%)	17 (85.0%)	8 (80.0%)	83 (92.2%)	39 (97.5%)
**Mild**	0	2 (5.0%)	3 (15.0%)	2 (20.0%)	7 (7.8%)	1 (2.5%)
**Diarrhea on Day 2 Post Injection**						
**n**	20	39	20	10	89	40
**Absent**	20 (100%)	38 (95.0%)	18 (90.0%)	8 (80.0%)	84 (93.3%)	40 (100%)
**Mild**	0	1 (2.5%)	2 (10.0%)	2 (20.0%)	5 (5.6%)	0
**Diarrhea on Day 3 Post Injection**						
**n**	20	40	20	10	90	40
**Absent**	20 (100%)	37 (92.5%)	19 (95.0%)	9 (90.0%)	85 (94.4%)	39 (97.5%)
**Mild**	0	3 (7.5%)	1 (5.0%)	1 (10.0%)	5 (5.6%)	1 (2.5%)
**Diarrhea on Day 4 Post Injection**						
**n**	20	40	20	10	90	40
**Absent**	20 (100%)	38 (95.0%)	18 (90.0%)	9 (90.0%)	85 (94.4%)	38 (95.0%)
**Mild**	0	1 (2.5%)	1 (5.0%)	1 (10.0%)	3 (3.3%)	1 (2.5%)
**Moderate**	0	1 (2.5%)	1 (5.0%)	0	2 (2.2%)	1 (2.5%)
**Diarrhea on Day 5 Post Injection**						
**n**	20	40	20	10	90	40
**Absent**	20 (100%)	39 (97.5%)	18 (90.0%)	9 (90.0%)	86 (95.6%)	38 (95.0%)

	Low Dose N = 20	Standard N= 40	High Dose N= 20	SC Injection N= 10	Combined N= 90	Control N= 40
**Mild**	0	1 (2.5%)	1 (5.0%)	1 (10.0%)	3 (3.3%)	2 (5.0%)
**Moderate**	0	0	1 (5.0%)	0	1 (1.1%)	0
**Diarrhea on Day 6 Post Injection**						
**n**	20	39	20	10	89	40
**Absent**	20 (100%)	38 (95.0%)	17 (85.0%)	8 (80.0%)	83 (92.2%)	39 (97.5%)
**Mild**	0	1 (2.5%)	3 (15.0%)	2 (20.0%)	6 (6.7%)	1 (2.5%)

Program V_diar Date: 13APR2023 Unique Number: 8790

Symptom scores are taken from the participants diary card

Table 4.13: Fatigue following injection – Safety Population

	Low Dose N = 20	Standard N= 40	High Dose N= 20	SC Injection N= 10	Combined N= 90	Control N= 40
**Fatigue on the Day of Injection**						
**n**	20	40	20	10	90	40
**Absent**	19 (95.0%)	35 (87.5%)	16 (80.0%)	9 (90.0%)	79 (87.8%)	39 (97.5%)
**Mild**	1 (5.0%)	5 (12.5%)	4 (20.0%)	1 (10.0%)	11 (12.2%)	1 (2.5%)
**Fatigue on Day 1 Post Injection**						
**n**	20	40	20	10	90	40
**Absent**	20 (100%)	38 (95.0%)	16 (80.0%)	9 (90.0%)	83 (92.2%)	37 (92.5%)
**Mild**	0	2 (5.0%)	4 (20.0%)	1 (10.0%)	7 (7.8%)	3 (7.5%)
**Fatigue on Day 2 Post Injection**						
**n**	20	39	20	10	89	40
**Absent**	18 (90.0%)	37 (92.5%)	17 (85.0%)	9 (90.0%)	81 (90.0%)	40 (100%)
**Mild**	2 (10.0%)	2 (5.0%)	0	1 (10.0%)	5 (5.6%)	0
**Moderate**	0	0	3 (15.0%)	0	3 (3.3%)	0
**Fatigue on Day 3 Post Injection**						
**n**	20	40	20	10	90	40
**Absent**	20 (100%)	36 (90.0%)	17 (85.0%)	10 (100%)	83 (92.2%)	40 (100%)
**Mild**	0	4 (10.0%)	2 (10.0%)	0	6 (6.7%)	0
**Moderate**	0	0	1 (5.0%)	0	1 (1.1%)	0
**Fatigue on Day 4 Post Injection**						
**n**	20	40	20	10	90	40
**Absent**	20 (100%)	36 (90.0%)	19 (95.0%)	10 (100%)	85 (94.4%)	40 (100%)
**Mild**	0	3 (7.5%)	0	0	3 (3.3%)	0
**Moderate**	0	1 (2.5%)	1 (5.0%)	0	2 (2.2%)	0
**Fatigue on Day 5 Post Injection**						
**n**	20	40	20	10	90	40
**Absent**	20 (100%)	38 (95.0%)	15 (75.0%)	10 (100%)	83 (92.2%)	40 (100%)

	Low Dose N = 20	Standard N= 40	High Dose N= 20	SC Injection N= 10	Combined N= 90	Control N= 40
**Mild**	0	2 (5.0%)	2 (10.0%)	0	4 (4.4%)	0
**Moderate**	0	0	2 (10.0%)	0	2 (2.2%)	0
**Severe**	0	0	1 (5.0%)	0	1 (1.1%)	0
**Fatigue on Day 6 Post Injection**						
**n**	20	39	20	10	89	40
**Absent**	20 (100%)	38 (95.0%)	16 (80.0%)	10 (100%)	84 (93.3%)	40 (100%)
**Mild**	0	1 (2.5%)	1 (5.0%)	0	2 (2.2%)	0
**Moderate**	0	0	2 (10.0%)	0	2 (2.2%)	0
**Severe**	0	0	1 (5.0%)	0	1 (1.1%)	0

Program V_fatg Date: 13APR2023 Unique Number: 8791

Symptom scores are taken from the participants diary card

Table 4.14: Malaise following injection – Safety Population

	Low Dose N = 20	Standard N= 40	High Dose N= 20	SC Injection N= 10	Combined N= 90	Control N= 40
**Malaise on the Day of Injection**						
**n**	20	40	20	10	90	40
**Absent**	19 (95.0%)	39 (97.5%)	20 (100%)	10 (100%)	88 (97.8%)	39 (97.5%)
**Mild**	1 (5.0%)	1 (2.5%)	0	0	2 (2.2%)	1 (2.5%)
**Malaise on Day 1 Post Injection**						
**n**	20	40	20	10	90	40
**Absent**	20 (100%)	39 (97.5%)	20 (100%)	10 (100%)	89 (98.9%)	40 (100%)
**Mild**	0	1 (2.5%)	0	0	1 (1.1%)	0
**Malaise on Day 2 Post Injection**						
**n**	20	39	20	10	89	40
**Absent**	19 (95.0%)	36 (90.0%)	20 (100%)	10 (100%)	85 (94.4%)	40 (100%)
**Mild**	1 (5.0%)	3 (7.5%)	0	0	4 (4.4%)	0
**Malaise on Day 3 Post Injection**						
**n**	20	40	20	10	90	40
**Absent**	20 (100%)	38 (95.0%)	19 (95.0%)	10 (100%)	87 (96.7%)	40 (100%)
**Mild**	0	2 (5.0%)	1 (5.0%)	0	3 (3.3%)	0
**Malaise on Day 4 Post Injection**						
**n**	20	40	20	10	90	40
**Absent**	20 (100%)	39 (97.5%)	20 (100%)	10 (100%)	89 (98.9%)	40 (100%)
**Mild**	0	1 (2.5%)	0	0	1 (1.1%)	0
**Malaise on Day 5 Post Injection**						
**n**	20	40	20	10	90	40
**Absent**	20 (100%)	40 (100%)	20 (100%)	10 (100%)	90 (100%)	40 (100%)
**Malaise on Day 6 Post Injection**						

	Low Dose N = 20	Standard N= 40	High Dose N= 20	SC Injection N= 10	Combined N= 90	Control N= 40
**n**	20	39	20	10	89	40
**Absent**	20 (100%)	39 (97.5%)	20 (100%)	10 (100%)	89 (98.9%)	40 (100%)

Program V_mals Date: 13APR2023 Unique Number: 8792

Symptom scores are taken from the participants diary card

Table 4.15: Myalgia following injection – Safety Population

	Low Dose N = 20	Standard N= 40	High Dose N= 20	SC Injection N= 10	Combined N= 90	Control N= 40
**Myalgia on the Day of Injection**						
**n**	20	40	20	10	90	40
**Absent**	17 (85.0%)	35 (87.5%)	17 (85.0%)	9 (90.0%)	78 (86.7%)	34 (85.0%)
**Mild**	3 (15.0%)	5 (12.5%)	3 (15.0%)	1 (10.0%)	12 (13.3%)	6 (15.0%)
**Myalgia on Day 1 Post Injection**						
**n**	20	40	20	10	90	40
**Absent**	18 (90.0%)	36 (90.0%)	18 (90.0%)	8 (80.0%)	80 (88.9%)	35 (87.5%)
**Mild**	2 (10.0%)	4 (10.0%)	2 (10.0%)	2 (20.0%)	10 (11.1%)	4 (10.0%)
**Moderate**	0	0	0	0	0	1 (2.5%)
**Myalgia on Day 2 Post Injection**						
**n**	20	39	20	10	89	40
**Absent**	18 (90.0%)	36 (90.0%)	19 (95.0%)	9 (90.0%)	82 (91.1%)	36 (90.0%)
**Mild**	2 (10.0%)	3 (7.5%)	1 (5.0%)	0	6 (6.7%)	3 (7.5%)
**Moderate**	0	0	0	1 (10.0%)	1 (1.1%)	1 (2.5%)
**Myalgia on Day 3 Post Injection**						
**n**	20	40	20	10	90	40
**Absent**	19 (95.0%)	38 (95.0%)	19 (95.0%)	8 (80.0%)	84 (93.3%)	38 (95.0%)
**Mild**	1 (5.0%)	2 (5.0%)	1 (5.0%)	2 (20.0%)	6 (6.7%)	2 (5.0%)
**Myalgia on Day 4 Post Injection**						
**n**	20	40	20	10	90	40
**Absent**	19 (95.0%)	36 (90.0%)	18 (90.0%)	9 (90.0%)	82 (91.1%)	39 (97.5%)
**Mild**	1 (5.0%)	4 (10.0%)	1 (5.0%)	1 (10.0%)	7 (7.8%)	1 (2.5%)
**Moderate**	0	0	1 (5.0%)	0	1 (1.1%)	0
**Myalgia on Day 5 Post Injection**						
**n**	20	40	20	10	90	40
**Absent**	19 (95.0%)	39 (97.5%)	20 (100%)	9 (90.0%)	87 (96.7%)	39 (97.5%)
**Mild**	1 (5.0%)	1 (2.5%)	0	1 (10.0%)	3 (3.3%)	0
**Moderate**	0	0	0	0	0	1 (2.5%)

	Low Dose N = 20	Standard N= 40	High Dose N= 20	SC Injection N= 10	Combined N= 90	Control N= 40
**Myalgia on Day 6 Post Injection**						
**n**	20	39	20	10	89	40
**Absent**	19 (95.0%)	37 (92.5%)	19 (95.0%)	9 (90.0%)	84 (93.3%)	39 (97.5%)
**Mild**	1 (5.0%)	2 (5.0%)	1 (5.0%)	1 (10.0%)	5 (5.6%)	0
**Moderate**	0	0	0	0	0	1 (2.5%)

Program V_mial Date: 13APR2023 Unique Number: 8793

Symptom scores are taken from the participants diary card

Table 4.16: Nausea following injection – Safety Population

	Low Dose N = 20	Standard N= 40	High Dose N= 20	SC Injection N= 10	Combined N= 90	Control N= 40
**Nausea on the Day of Injection**						
**n**	20	40	20	10	90	40
**Absent**	19 (95.0%)	39 (97.5%)	19 (95.0%)	10 (100%)	87 (96.7%)	37 (92.5%)
**Mild**	1 (5.0%)	1 (2.5%)	1 (5.0%)	0	3 (3.3%)	3 (7.5%)
**Nausea on Day 1 Post Injection**						
**n**	20	40	20	10	90	40
**Absent**	20 (100%)	40 (100%)	17 (85.0%)	9 (90.0%)	86 (95.6%)	39 (97.5%)
**Mild**	0	0	3 (15.0%)	1 (10.0%)	4 (4.4%)	1 (2.5%)
**Nausea on Day 2 Post Injection**						
**n**	20	39	20	10	89	40
**Absent**	19 (95.0%)	37 (92.5%)	19 (95.0%)	9 (90.0%)	84 (93.3%)	39 (97.5%)
**Mild**	0	1 (2.5%)	0	1 (10.0%)	2 (2.2%)	1 (2.5%)
**Moderate**	1 (5.0%)	0	1 (5.0%)	0	2 (2.2%)	0
**Severe**	0	1 (2.5%)	0	0	1 (1.1%)	0
**Nausea on Day 3 Post Injection**						
**n**	20	40	20	10	90	40
**Absent**	20 (100%)	38 (95.0%)	17 (85.0%)	9 (90.0%)	84 (93.3%)	39 (97.5%)
**Mild**	0	1 (2.5%)	3 (15.0%)	1 (10.0%)	5 (5.6%)	1 (2.5%)
**Moderate**	0	1 (2.5%)	0	0	1 (1.1%)	0
**Nausea on Day 4 Post Injection**						
**n**	20	40	20	10	90	40
**Absent**	20 (100%)	38 (95.0%)	17 (85.0%)	10 (100%)	85 (94.4%)	40 (100%)
**Mild**	0	2 (5.0%)	2 (10.0%)	0	4 (4.4%)	0
**Moderate**	0	0	1 (5.0%)	0	1 (1.1%)	0
**Nausea on Day 5 Post Injection**						
**n**	20	40	20	10	90	40

	Low Dose N = 20	Standard N= 40	High Dose N= 20	SC Injection N= 10	Combined N= 90	Control N= 40
**Absent**	20 (100%)	39 (97.5%)	18 (90.0%)	9 (90.0%)	86 (95.6%)	40 (100%)
**Mild**	0	1 (2.5%)	1 (5.0%)	1 (10.0%)	3 (3.3%)	0
**Moderate**	0	0	1 (5.0%)	0	1 (1.1%)	0
**Nausea on Day 6 Post Injection**						
**n**	20	39	20	10	89	40
**Absent**	20 (100%)	39 (97.5%)	19 (95.0%)	10 (100%)	88 (97.8%)	40 (100%)
**Mild**	0	0	1 (5.0%)	0	1 (1.1%)	0

Program V_naus Date: 13APR2023 Unique Number: 8794

Symptom scores are taken from the participants diary card

Table 4.17: Vomiting following injection – Safety Population

	Low Dose N = 20	Standard N= 40	High Dose N= 20	SC Injection N= 10	Combined N= 90	Control N= 40
**Vomiting on the Day of Injection**						
**n**	20	40	20	10	90	40
**Absent**	20 (100%)	40 (100%)	19 (95.0%)	9 (90.0%)	88 (97.8%)	40 (100%)
**Mild**	0	0	1 (5.0%)	1 (10.0%)	2 (2.2%)	0
**Vomiting on Day 1 Post Injection**						
**n**	20	40	20	10	90	40
**Absent**	20 (100%)	40 (100%)	20 (100%)	9 (90.0%)	89 (98.9%)	40 (100%)
**Mild**	0	0	0	1 (10.0%)	1 (1.1%)	0
**Vomiting on Day 2 Post Injection**						
**n**	20	39	20	10	89	40
**Absent**	19 (95.0%)	38 (95.0%)	20 (100%)	10 (100%)	87 (96.7%)	40 (100%)
**Mild**	0	1 (2.5%)	0	0	1 (1.1%)	0
**Moderate**	1 (5.0%)	0	0	0	1 (1.1%)	0
**Vomiting on Day 3 Post Injection**						
**n**	20	40	20	10	90	40
**Absent**	20 (100%)	40 (100%)	19 (95.0%)	10 (100%)	89 (98.9%)	40 (100%)
**Mild**	0	0	1 (5.0%)	0	1 (1.1%)	0
**Vomiting on Day 4 Post Injection**						
**n**	20	40	20	10	90	40
**Absent**	20 (100%)	40 (100%)	19 (95.0%)	10 (100%)	89 (98.9%)	40 (100%)
**Moderate**	0	0	1 (5.0%)	0	1 (1.1%)	0
**Vomiting on Day 5 Post Injection**						
**n**	20	40	20	10	90	40
**Absent**	20 (100%)	40 (100%)	19 (95.0%)	10 (100%)	89 (98.9%)	40 (100%)

	Low Dose N = 20	Standard N= 40	High Dose N= 20	SC Injection N= 10	Combined N= 90	Control N= 40
**Moderate**	0	0	1 (5.0%)	0	1 (1.1%)	0
**Vomiting on Day 6 Post Injection**						
**n**	20	39	20	10	89	40
**Absent**	20 (100%)	38 (95.0%)	19 (95.0%)	10 (100%)	87 (96.7%)	40 (100%)
**Mild**	0	1 (2.5%)	1 (5.0%)	0	2 (2.2%)	0

Program V_vomt Date: 13APR2023 Unique Number: 8795

Symptom scores are taken from the participants diary card

Table 4.18: Erythema following injection – Safety Population

	Low Dose N = 20	Standard N= 40	High Dose N= 20	SC Injection N= 10	Combined N= 90	Control N= 40
**Erythema (mm) on Injection Day**						
**n**	20	39	20	10	89	40
**Mean (SD)**	0.90 (2.65)	1.97 (3.96)	4.45 (7.34)	1.10 (2.18)	2.19 (4.70)	0.90 (2.84)
**Median**	0.00	0.00	0.50	0.00	0.00	0.00
**Min-Max**	0.00 to 10.00	0.00 to 16.00	0.00 to 25.00	0.00 to 7.00	0.00 to 25.00	0.00 to 16.00
**Erythema (mm) on Day 1 Post Injection**						
**n**	20	39	20	10	89	40
**Mean (SD)**	0.50 (2.01)	3.59 (16.03)	3.65 (6.04)	0.70 (1.57)	2.58 (11.05)	0.38 (1.08)
**Median**	0.00	0.00	0.00	0.00	0.00	0.00
**Min-Max**	0.00 to 9.00	0.00 to 100.00	0.00 to 18.00	0.00 to 5.00	0.00 to 100.00	0.00 to 5.00
**Erythema (mm) on Day 2 Post Injection**						
**n**	20	38	20	10	88	40
**Mean (SD)**	0.45 (1.28)	0.50 (1.78)	3.75 (6.23)	0.50 (0.97)	1.23 (3.49)	0.30 (0.97)
**Median**	0.00	0.00	0.00	0.00	0.00	0.00
**Min-Max**	0.00 to 5.00	0.00 to 9.00	0.00 to 18.00	0.00 to 3.00	0.00 to 18.00	0.00 to 4.00
**Erythema (mm) on Day 3 Post Injection**						
**n**	20	38	20	10	88	40
**Mean (SD)**	0.20 (0.70)	0.26 (0.92)	2.00 (3.93)	0.50 (0.97)	0.67 (2.12)	0.05 (0.22)
**Median**	0.00	0.00	0.00	0.00	0.00	0.00
**Min-Max**	0.00 to 3.00	0.00 to 4.00	0.00 to 14.00	0.00 to 3.00	0.00 to 14.00	0.00 to 1.00
**Erythema (mm) on Day 4 Post Injection**						
**n**	20	38	20	10	88	40
**Mean (SD)**	0.05 (0.22)	0.11 (0.45)	2.00 (3.39)	0.40 (0.70)	0.56 (1.81)	0.00 (0.00)
**Median**	0.00	0.00	0.00	0.00	0.00	0.00
**Min-Max**	0.00 to 1.00	0.00 to 2.00	0.00 to 10.00	0.00 to 2.00	0.00 to 10.00	0.00 to 0.00
**Erythema (mm) on Day 5 Post Injection**						
**n**	20	38	20	10	88	40
**Mean (SD)**	0.05 (0.22)	0.03 (0.16)	1.30 (2.58)	0.40 (0.70)	0.36 (1.34)	0.00 (0.00)

	Low Dose N = 20	Standard N= 40	High Dose N= 20	SC Injection N= 10	Combined N= 90	Control N= 40
**Median**	0.00	0.00	0.00	0.00	0.00	0.00
**Min-Max**	0.00 to 1.00	0.00 to 1.00	0.00 to 9.00	0.00 to 2.00	0.00 to 9.00	0.00 to 0.00
**Erythema (mm) on Day 6 Post Injection**						
**n**	20	37	20	10	87	40
**Mean (SD)**	0.05 (0.22)	0.00 (0.00)	1.35 (2.50)	0.30 (0.67)	0.36 (1.32)	0.00 (0.00)
**Median**	0.00	0.00	0.00	0.00	0.00	0.00
**Min-Max**	0.00 to 1.00	0.00 to 0.00	0.00 to 9.00	0.00 to 2.00	0.00 to 9.00	0.00 to 0.00

Program V_eryth Date: 13APR2023 Unique Number: 8796

Measurements of Erythema are taken from the participants diary card

Table 4.19: Body Temperature following injection – Safety Population

	Low Dose N = 20	Standard N= 40	High Dose N= 20	SC Injection N= 10	Combined N= 90	Control N= 40
**Body Temperature on Injection Day**						
**n**	20	40	20	10	90	40
**Mean (SD)**	36.2 (0.56)	36.2 (0.57)	36.2 (0.72)	35.6 (1.97)	36.1 (0.86)	36.3 (0.98)
**Median**	36.2	36.3	36.4	36.4	36.3	36.7
**Min-Max**	35.2 to 37.3	34.4 to 36.9	34.6 to 37.0	30.5 to 37.0	30.5 to 37.3	33.0 to 37.4
**Body Temperature on Day 1 Post Injection**						
**n**	20	40	20	10	90	40
**Mean (SD)**	36.1 (0.89)	36.1 (0.86)	35.9 (1.08)	36.3 (0.61)	36.1 (0.89)	36.1 (1.13)
**Median**	36.3	36.3	36.3	36.4	36.3	36.4
**Min-Max**	33.6 to 37.1	32.0 to 37.0	33.9 to 37.2	35.2 to 36.9	32.0 to 37.2	31.2 to 37.2
**Body Temperature on Day 2 Post Injection**						
**n**	20	39	20	10	89	40
**Mean (SD)**	36.1 (0.64)	36.3 (0.79)	36.3 (0.72)	35.9 (1.23)	36.2 (0.80)	36.3 (0.65)
**Median**	36.2	36.3	36.4	36.3	36.3	36.4
**Min-Max**	34.6 to 37.3	33.8 to 39.2	34.7 to 37.9	33.0 to 37.1	33.0 to 39.2	34.1 to 37.0
**Body Temperature on Day 3 Post Injection**						
**n**	20	40	20	10	90	40
**Mean (SD)**	36.1 (0.87)	36.1 (0.81)	36.2 (0.82)	36.2 (0.96)	36.1 (0.83)	36.1 (0.88)
**Median**	36.2	36.3	36.4	36.3	36.3	36.3
**Min-Max**	33.4 to 37.0	34.0 to 38.7	34.5 to 37.9	34.0 to 37.3	33.4 to 38.7	33.1 to 37.4
**Body Temperature on Day 4 Post Injection**						
**n**	19	40	20	10	89	40
**Mean (SD)**	36.2 (0.54)	36.1 (0.87)	36.4 (1.07)	35.9 (1.38)	36.2 (0.93)	36.1 (1.03)
**Median**	36.3	36.3	36.5	36.3	36.3	36.5
**Min-Max**	35.0 to 37.1	32.0 to 37.2	34.7 to 40.1	32.6 to 37.3	32.0 to 40.1	32.1 to 37.0
**Body Temperature on Day 5 Post Injection**						
**n**	20	40	20	10	90	40
**Mean (SD)**	36.1 (0.65)	36.2 (0.69)	36.2 (0.53)	36.1 (0.69)	36.2 (0.64)	36.1 (0.88)
**Median**	36.3	36.3	36.4	36.3	36.3	36.3

	Low Dose N = 20	Standard N= 40	High Dose N= 20	SC Injection N= 10	Combined N= 90	Control N= 40
**Min-Max**	34.3 to 36.9	33.4 to 37.1	35.0 to 36.7	34.7 to 36.9	33.4 to 37.1	32.0 to 37.8
**Body Temperature on Day 6 Post Injection**						
**n**	20	39	20	10	89	40
**Mean (SD)**	36.2 (0.51)	36.2 (0.55)	35.9 (1.44)	36.3 (0.80)	36.1 (0.85)	36.0 (1.13)
**Median**	36.2	36.2	36.3	36.4	36.2	36.2
**Min-Max**	35.3 to 37.1	34.8 to 36.9	30.1 to 36.9	34.3 to 37.2	30.1 to 37.2	32.3 to 38.5

Program V_temp Date: 13APR2023 Unique Number: 8797

Body Temperatures are taken from the participants diary card

Table 5: Physical Examination at Day 7 – mITT Population

	Low Dose	Standard	High Dose	SC Injection	Combined	Control
	N= 20	N= 40	N= 20	N= 10	N= 90	N= 40
**Was a Physical Exam Performed?**						
**n**	20	40	20	10	90	40
**Yes**	19 (95.0%)	34 (85.0%)	15 (75.0%)	6 (60.0%)	74 (82.2%)	36 (90.0%)
**No**	1 (5.0%)	6 (15.0%)	5 (25.0%)	4 (40.0%)	16 (17.8%)	4 (10.0%)
**Number of Participants showing changes**						
**n**	19	34	15	6	74	36
**Yes**	1 (5.0%)	0	2 (10.0%)	1 (10.0%)	4 (4.4%)	1 (2.5%)
**No**	18 (90.0%)	34 (85.0%)	13 (65.0%)	5 (50.0%)	70 (77.8%)	35 (87.5%)
**Participants with Abnormal changes**						
**n**	0	0	2	1	3	1
**Yes**			2 (10.0%)	1 (10.0%)	3 (3.3%)	1 (2.5%)
**Abnormalities which were Clinically Significant**						
**n**	0	0	2	2	4	0
**Ears, nose, mouth and throat**			1 (5.0%)	1 (10.0%)	2 (2.2%)	
**Lymphatic**			0	1 (10.0%)	1 (1.1%)	
**Skin**			1 (5.0%)	0	1 (1.1%)	
**Abnormalities which were Not Clinically Significant**						
**n**	0	0	0	1	1	1
**Ears, nose, mouth and throat**				0	0	1 (2.5%)
**Skin**				1 (10.0%)	1 (1.1%)	0

Program V_phys02 Date: 13APR2023 Unique Number: 8798

Only abnormal findings are included in the table

If more than one change is noted in the same participant, it is recorded as a single participant change

Table 6: Vital Signs 7 days Post Dose – mITT Population

	Low Dose<br>N= 20	Standard<br>N= 40	High Dose<br>N= 20	SC Injection<br>N= 10	Combined<br>N= 90	Control<br>N= 40
**Systolic Blood Pressure (mmHg)**						
**n**	19	40	20	10	89	40
**Mean (SD)**	122.1 (10.72)	124.3 (9.57)	117.3 (11.51)	119.3 (13.07)	121.7 (10.88)	123.3 (13.27)
**Median**	123.0	123.5	117.5	117.0	123.0	123.0
**Min-Max**	95.0 to 138.0	106.0 to 148.0	97.0 to 136.0	101.0 to 137.0	95.0 to 148.0	100.0 to 154.0
**Diastolic Blood Pressure (mmHg)**						
**n**	19	40	20	10	89	40
**Mean (SD)**	75.5 (8.92)	78.5 (7.20)	77.1 (6.53)	83.4 (8.21)	78.1 (7.77)	80.1 (8.78)
**Median**	78.0	77.5	76.0	84.5	78.0	82.0
**Min-Max**	60.0 to 88.0	60.0 to 100.0	65.0 to 88.0	71.0 to 95.0	60.0 to 100.0	51.0 to 97.0
**Body Temperature (C)**						
**n**	19	40	20	10	89	40
**Mean (SD)**	36.3 (0.38)	36.4 (0.35)	36.2 (0.56)	36.3 (0.69)	36.3 (0.45)	36.4 (0.41)
**Median**	36.3	36.5	36.3	36.2	36.4	36.4
**Min-Max**	35.4 to 36.9	35.5 to 36.9	35.0 to 37.1	35.3 to 37.6	35.0 to 37.6	35.5 to 37.2
**Heart Rate (BPM)**						
**n**	19	40	20	10	89	40
**Mean (SD)**	77.5 (8.28)	74.7 (11.19)	75.2 (11.26)	83.1 (12.32)	76.3 (10.93)	75.3 (11.29)
**Median**	79.0	75.0	71.5	83.0	76.0	74.0
**Min-Max**	64.0 to 90.0	53.0 to 100.0	60.0 to 100.0	61.0 to 100.0	53.0 to 100.0	55.0 to 97.0

Program V_signs03 Date: 13APR2023 Unique Number: 8799

Table 7: Clinical Chemistry data – Safety Population

	Low Dose N= 20	Standard N= 40	High Dose N= 20	SC Injection N= 10	Combined N= 90	Control N= 40
**ALT at Baseline (U/L)**						
**n**	20	40	20	10	90	40
**Mean (SD)**	22.6 (12.14)	19.8 (9.70)	19.8 (11.34)	17.6 (7.17)	20.2 (10.37)	20.0 (10.20)
**Median**	19.5	17.0	15.0	16.5	17.0	17.5
**Min-Max**	10.0 to 62.0	10.0 to 58.0	9.0 to 45.0	10.0 to 30.0	9.0 to 62.0	7.0 to 61.0
**ALT at Day 7 (U/L)**						
**n**	18	39	20	10	87	40
**Mean (SD)**	23.8 (12.74)	17.8 (6.49)	19.0 (11.14)	15.4 (3.31)	19.0 (9.29)	20.4 (11.61)
**Median**	20.5	16.0	15.5	16.0	17.0	18.0
**Min-Max**	10.0 to 62.0	9.0 to 39.0	10.0 to 49.0	10.0 to 21.0	9.0 to 62.0	9.0 to 76.0
**AST at Baseline (U/L)**						
**n**	20	40	20	10	90	40
**Mean (SD)**	24.9 (12.04)	21.1 (5.42)	23.7 (12.26)	21.4 (4.17)	22.5 (8.96)	23.7 (10.10)
**Median**	24.5	20.5	21.5	20.0	21.0	21.5
**Min-Max**	14.0 to 67.0	11.0 to 41.0	13.0 to 67.0	14.0 to 27.0	11.0 to 67.0	12.0 to 59.0
**AST at Day 7 (U/L)**						
**n**	18	39	20	10	87	40
**Mean (SD)**	29.7 (22.47)	21.3 (7.48)	23.1 (10.05)	21.2 (6.96)	23.4 (12.76)	25.9 (20.09)
**Median**	22.0	20.0	21.0	19.0	20.0	21.0
**Min-Max**	13.0 to 98.0	10.0 to 56.0	13.0 to 56.0	12.0 to 37.0	10.0 to 98.0	12.0 to 142.0
**Creatinine at Baseline (umol/L)**						
**n**	20	40	20	10	90	40
**Mean (SD)**	69.7 (11.34)	71.5 (14.64)	67.8 (11.65)	57.8 (12.02)	68.8 (13.50)	73.3 (15.23)
**Median**	70.7	72.5	69.8	57.0	69.8	72.9
**Min-Max**	53.0 to 88.4	38.9 to 108.7	46.9 to 89.3	38.9 to 76.0	38.9 to 108.7	48.6 to 106.1
**Creatinine at Day 7 (umol/L)**						
**n**	18	39	20	10	87	40
**Mean (SD)**	69.3 (10.98)	72.7 (16.13)	72.0 (11.11)	62.0 (12.50)	70.6 (13.92)	76.3 (16.04)

	Low Dose<br>N= 20	Standard<br>N= 40	High Dose<br>N= 20	SC Injection<br>N= 10	Combined<br>N= 90	Control<br>N= 40
**Median**	71.2	74.3	73.4	65.9	72.5	77.8
**Min-Max**	48.6 to 86.6	40.7 to 114.0	55.7 to 91.9	44.2 to 75.1	40.7 to 114.0	46.0 to 114.0
**Total Bilirubin at Baseline (umol/L)**						
**n**	20	40	20	10	90	40
**Mean (SD)**	8.4 (6.48)	6.9 (3.10)	5.9 (2.74)	6.5 (2.37)	7.0 (4.01)	7.9 (5.25)
**Median**	5.0	6.0	5.0	6.5	6.0	6.5
**Min-Max**	2.0 to 23.0	3.0 to 19.0	1.0 to 11.0	3.0 to 10.0	1.0 to 23.0	2.0 to 27.0
**Total Bilirubin at Day 7 (umol/L)**						
**n**	18	39	20	10	87	40
**Mean (SD)**	8.3 (5.18)	6.8 (4.33)	7.2 (3.69)	6.6 (4.72)	7.2 (4.40)	8.5 (6.14)
**Median**	7.0	6.0	6.0	5.0	6.0	6.0
**Min-Max**	1.0 to 18.0	2.0 to 24.0	2.0 to 15.0	3.0 to 18.0	1.0 to 24.0	3.0 to 29.0
**Urea Nitrogen at Baseline (umol/L)**						
**n**	20	40	20	10	90	40
**Mean (SD)**	3.7 (1.00)	3.4 (1.16)	3.8 (1.11)	3.0 (0.60)	3.5 (1.08)	3.5 (0.99)
**Median**	3.4	3.1	3.7	2.9	3.3	3.4
**Min-Max**	2.0 to 6.1	1.6 to 7.4	2.3 to 5.7	1.9 to 3.7	1.6 to 7.4	1.7 to 5.9
**Urea Nitrogen at Day 7 (umol/L)**						
**n**	18	39	20	10	87	40
**Mean (SD)**	3.6 (0.95)	3.3 (1.21)	3.5 (0.91)	3.0 (0.61)	3.4 (1.04)	3.7 (1.22)
**Median**	3.6	3.2	3.3	3.0	3.3	3.4
**Min-Max**	1.8 to 5.8	1.1 to 6.3	2.1 to 6.3	2.1 to 4.1	1.1 to 6.3	2.0 to 7.4

Program V_chem Date: 13APR2023 Unique Number: 8800

Table 7.1: Change from Baseline Clinical Chemistry – Safety Population

	Low Dose N= 20	Standard N= 40	High Dose N= 20	SC Injection N= 10	Combined N= 90	Control N= 40
**Change in ALT (U/L)**						
**n**	18	39	20	10	87	40
**Mean (SD)**	0.4 (11.75)	-2.1 (9.41)	-0.8 (7.59)	-2.2 (5.47)	-1.3 (9.13)	0.4 (7.94)
**Median**	1.5	-2.0	-0.5	-2.0	-1.0	0.0
**Min-Max**	-38.0 to 20.0	-32.0 to 25.0	-23.0 to 10.0	-12.0 to 5.0	-38.0 to 25.0	-27.0 to 30.0
**Change in AST (U/L)**						
**n**	18	39	20	10	87	40
**Mean (SD)**	4.4 (13.31)	0.1 (8.94)	-0.6 (5.79)	-0.2 (4.85)	0.8 (9.15)	2.2 (14.61)
**Median**	0.0	0.0	0.0	-1.0	0.0	1.0
**Min-Max**	-13.0 to 34.0	-22.0 to 37.0	-13.0 to 10.0	-8.0 to 11.0	-22.0 to 37.0	-25.0 to 83.0
**Change in Creatinine (umol/L)**						
**n**	18	39	20	10	87	40
**Mean (SD)**	-0.4 (3.73)	1.0 (5.74)	4.2 (5.91)	4.2 (6.68)	1.8 (5.75)	3.0 (6.45)
**Median**	0.0	0.0	4.0	3.1	0.9	1.3
**Min-Max**	-8.0 to 5.3	-8.8 to 12.4	-6.2 to 16.8	-4.4 to 18.6	-8.8 to 18.6	-9.7 to 16.8
**Change in Total Bilirubin (umol/L)**						
**n**	18	39	20	10	87	40
**Mean (SD)**	-0.61 (2.73)	-0.15 (4.18)	1.35 (2.06)	0.10 (4.28)	0.13 (3.54)	0.58 (5.81)
**Median**	-0.50	0.00	1.00	-0.50	0.00	0.00
**Min-Max**	-8.00 to 3.00	-9.00 to 15.00	-2.00 to 5.00	-4.00 to 11.00	-9.00 to 15.00	-15.00 to 17.00
**Change in Urea Nitrogen (umol/L)**						
**n**	18	39	20	10	87	40
**Mean (SD)**	-0.06 (1.29)	-0.07 (1.08)	-0.34 (1.46)	-0.02 (0.54)	-0.12 (1.17)	0.24 (1.10)
**Median**	0.16	-0.33	-0.68	0.07	0.00	0.03
**Min-Max**	-2.54 to 2.18	-2.79 to 2.25	-3.00 to 2.78	-1.25 to 0.75	-3.00 to 2.78	-1.18 to 4.43

Program V_chem02 Date: 13APR2023 Unique Number: 8801

Table 7.2: Clinical Chem Normal Ranges – Safety Population

	Low Dose<br>N= 20	Standard<br>N= 40	High Dose<br>N= 20	SC Injection<br>N= 10	Combined<br>N= 90	Control<br>N= 40
**ALT at Baseline (U/L)**						
**n**	20	40	20	10	90	40
**Above Normal Range**	2 (10.0%)	1 (2.5%)	2 (10.0%)	0	5 (5.6%)	2 (5.0%)
**Within Normal Range**	18 (90.0%)	39 (97.5%)	18 (90.0%)	10 (100%)	85 (94.4%)	38 (95.0%)
**ALT at Day 7 (U/L)**						
**n**	18	39	20	10	87	40
**Above Normal Range**	2 (10.0%)	0	2 (10.0%)	0	4 (4.4%)	1 (2.5%)
**Within Normal Range**	16 (80.0%)	39 (97.5%)	18 (90.0%)	10 (100%)	83 (92.2%)	39 (97.5%)
**AST at Baseline (U/L)**						
**n**	20	40	20	10	90	40
**Above Normal Range**	2 (10.0%)	1 (2.5%)	2 (10.0%)	0	5 (5.6%)	5 (12.5%)
**Within Normal Range**	18 (90.0%)	39 (97.5%)	18 (90.0%)	10 (100%)	85 (94.4%)	35 (87.5%)
**AST at Day 7 (U/L)**						
**n**	18	39	20	10	87	40
**Above Normal Range**	3 (15.0%)	1 (2.5%)	1 (5.0%)	0	5 (5.6%)	3 (7.5%)
**Within Normal Range**	15 (75.0%)	38 (95.0%)	19 (95.0%)	10 (100%)	82 (91.1%)	37 (92.5%)
**Creatinine at Baseline (umol/L)**						
**n**	20	40	20	10	90	40
**Above Normal Range**	0	1 (2.5%)	0	0	1 (1.1%)	3 (7.5%)
**Within Normal Range**	20 (100%)	35 (87.5%)	20 (100%)	7 (70.0%)	82 (91.1%)	37 (92.5%)
**Below Normal Range**	0	4 (10.0%)	0	3 (30.0%)	7 (7.8%)	0
**Creatinine at Day 7 (umol/L)**						
**n**	18	39	20	10	87	40
**Above Normal Range**	0	1 (2.5%)	0	0	1 (1.1%)	4 (10.0%)
**Within Normal Range**	18 (90.0%)	34 (85.0%)	20 (100%)	9 (90.0%)	81 (90.0%)	36 (90.0%)
**Below Normal Range**	0	4 (10.0%)	0	1 (10.0%)	5 (5.6%)	0

	Low Dose<br>N= 20	Standard<br>N= 40	High Dose<br>N= 20	SC Injection<br>N= 10	Combined<br>N= 90	Control<br>N= 40
**Total Bilirubin at Baseline (umol/L)**						
**n**	20	40	20	10	90	40
**Above Normal Range**	1 (5.0%)	0	0	0	1 (1.1%)	1 (2.5%)
**Within Normal Range**	19 (95.0%)	40 (100%)	20 (100%)	10 (100%)	89 (98.9%)	39 (97.5%)
**Total Bilirubin at Day 7 (umol/L)**						
**n**	18	39	20	10	87	40
**Above Normal Range**	0	1 (2.5%)	0	0	1 (1.1%)	3 (7.5%)
**Within Normal Range**	18 (90.0%)	38 (95.0%)	20 (100%)	10 (100%)	86 (95.6%)	37 (92.5%)
**Urea Nitrogen at Baseline (umol/L)**						
**n**	20	40	20	10	90	40
**Above Normal Range**	0	1 (2.5%)	0	0	1 (1.1%)	0
**Within Normal Range**	19 (95.0%)	36 (90.0%)	20 (100%)	9 (90.0%)	84 (93.3%)	38 (95.0%)
**Below Normal Range**	1 (5.0%)	3 (7.5%)	0	1 (10.0%)	5 (5.6%)	2 (5.0%)
**Urea Nitrogen at Day 7 (umol/L)**						
**n**	18	39	20	10	87	40
**Above Normal Range**	0	0	0	0	0	1 (2.5%)
**Within Normal Range**	17 (85.0%)	34 (85.0%)	19 (95.0%)	9 (90.0%)	79 (87.8%)	38 (95.0%)
**Below Normal Range**	1 (5.0%)	5 (12.5%)	1 (5.0%)	1 (10.0%)	8 (8.9%)	1 (2.5%)

Program V_chem03 Date: 13APR2023 Unique Number: 8802

Table 8: Hematology data – Safety Population

	Low Dose<br>N= 20	Standard<br>N= 40	High Dose<br>N= 20	SC Injection<br>N= 10	Combined<br>N= 90	Control<br>N= 40
**Hemoglobin at Baseline (g/dL)**						
**n**	20	40	19	10	89	40
**Mean (SD)**	14.5 (1.31)	15.0 (1.09)	14.0 (1.05)	14.4 (1.07)	14.6 (1.18)	15.1 (1.58)
**Median**	14.6	15.2	13.7	14.1	14.5	15.3
**Min-Max**	12.1 to 16.6	12.4 to 17.7	12.6 to 16.6	12.8 to 15.8	12.1 to 17.7	11.7 to 18.2
**Hemoglobin at Day 7 (g/dL)**						
**n**	19	40	20	9	88	40
**Mean (SD)**	14.6 (1.44)	14.7 (1.27)	14.2 (1.24)	14.0 (0.93)	14.5 (1.27)	15.1 (1.64)
**Median**	14.4	14.8	14.1	14.1	14.5	15.0
**Min-Max**	12.4 to 16.7	11.3 to 17.5	12.3 to 16.5	12.4 to 15.3	11.3 to 17.5	11.5 to 19.1
**Erythrocytes at Baseline (x10^12/L)**						
**n**	20	40	19	10	89	40
**Mean (SD)**	4.8 (0.60)	5.1 (0.36)	4.6 (0.35)	4.8 (0.47)	4.9 (0.46)	5.1 (0.60)
**Median**	4.7	5.0	4.6	4.8	4.8	5.0
**Min-Max**	4.0 to 6.2	4.4 to 6.1	4.0 to 5.5	4.1 to 5.6	4.0 to 6.2	4.1 to 6.5
**Erythrocytes at Day 7 (x10^12/L)**						
**n**	19	40	20	9	88	40
**Mean (SD)**	4.9 (0.63)	4.9 (0.45)	4.7 (0.41)	4.7 (0.43)	4.8 (0.49)	5.1 (0.62)
**Median**	4.8	4.9	4.6	4.7	4.8	5.0
**Min-Max**	4.1 to 6.5	4.1 to 6.4	4.0 to 5.6	4.1 to 5.4	4.0 to 6.5	4.0 to 7.0
**Platelets at Baseline (x10^9/L)**						
**n**	20	37	19	10	86	40
**Mean (SD)**	282.4 (71.93)	274.3 (77.61)	278.1 (87.18)	294.3 (59.68)	279.3 (75.73)	287.0 (64.88)
**Median**	264.5	263.0	272.0	288.5	274.0	286.0
**Min-Max**	167.0 to 415.0	121.0 to 473.0	145.0 to 458.0	185.0 to 391.0	121.0 to 473.0	169.0 to 416.0
**Platelets at Day 7 (x10^9/L)**						
**n**	17	36	20	9	82	39
**Mean (SD)**	298.9 (71.10)	281.9 (77.38)	272.8 (67.93)	288.2 (68.19)	283.9 (72.18)	288.9 (73.57)

	Low Dose	Standard	High Dose	SC Injection	Combined	Control
	N= 20	N= 40	N= 20	N= 10	N= 90	N= 40
**Median**	292.0	283.0	264.0	271.0	275.5	284.0
**Min-Max**	194.0 to 419.0	127.0 to 476.0	125.0 to 417.0	210.0 to 406.0	125.0 to 476.0	154.0 to 477.0
**Leukocytes at Baseline (x10^9/L)**						
**n**	20	40	19	10	89	40
**Mean (SD)**	6.6 (2.09)	6.1 (1.53)	6.6 (2.21)	5.8 (1.61)	6.3 (1.83)	6.4 (2.16)
**Median**	6.4	6.0	6.0	5.7	6.0	5.8
**Min-Max**	4.0 to 11.0	3.6 to 10.1	3.4 to 12.3	3.9 to 9.1	3.4 to 12.3	3.3 to 11.5
**Leukocytes at Day 7 (x10^9/L)**						
**n**	19	40	20	9	88	40
**Mean (SD)**	6.4 (2.16)	5.7 (1.60)	5.6 (2.02)	6.0 (2.19)	5.8 (1.88)	6.0 (2.14)
**Median**	6.0	5.5	5.0	5.6	5.5	5.6
**Min-Max**	3.4 to 10.2	2.2 to 10.3	3.4 to 11.2	3.3 to 10.3	2.2 to 11.2	2.8 to 10.8
**Lymphocytes at Baseline (x10^9/L)**						
**n**	20	40	19	10	89	40
**Mean (SD)**	2.4 (0.68)	2.3 (0.63)	2.4 (0.79)	2.2 (0.54)	2.4 (0.66)	2.5 (0.73)
**Median**	2.3	2.3	2.3	2.3	2.3	2.3
**Min-Max**	1.0 to 3.7	1.1 to 3.4	1.3 to 4.0	1.1 to 2.8	1.0 to 4.0	1.4 to 4.4
**Lymphocytes at Day 7 (x10^9/L)**						
**n**	19	40	20	9	88	40
**Mean (SD)**	2.2 (0.55)	2.0 (0.52)	2.0 (0.65)	2.1 (0.67)	2.1 (0.57)	2.2 (0.66)
**Median**	2.1	2.1	1.9	2.1	2.1	2.1
**Min-Max**	1.1 to 3.4	1.0 to 3.2	0.7 to 3.2	1.2 to 3.2	0.7 to 3.4	0.7 to 4.3
**Neutrophils at Baseline (x10^9/L)**						
**n**	20	40	19	10	89	40
**Mean (SD)**	3.5 (1.54)	3.2 (1.25)	3.6 (1.74)	3.1 (1.41)	3.3 (1.44)	3.4 (1.65)
**Median**	3.1	2.8	3.2	2.7	2.9	2.7
**Min-Max**	1.6 to 6.7	1.3 to 6.9	1.4 to 8.0	1.4 to 5.9	1.3 to 8.0	1.0 to 7.4
**Neutrophils at Day 7 (x10^9/L)**						
**n**	19	40	20	9	88	40
**Mean (SD)**	3.6 (1.79)	3.1 (1.19)	3.1 (1.81)	3.2 (2.00)	3.2 (1.56)	3.3 (1.66)
**Median**	3.3	2.9	2.3	2.7	2.8	2.7
**Min-Max**	1.2 to 7.5	0.6 to 6.4	1.7 to 8.3	1.1 to 7.7	0.6 to 8.3	1.1 to 6.8

	Low Dose<br>N= 20	Standard<br>N= 40	High Dose<br>N= 20	SC Injection<br>N= 10	Combined<br>N= 90	Control<br>N= 40
**Basophils at Baseline (x10^9/L)**						
**n**	20	40	19	10	89	40
**Mean (SD)**	0.06 (0.04)	0.05 (0.02)	0.07 (0.03)	0.04 (0.03)	0.06 (0.03)	0.05 (0.03)
**Median**	0.06	0.05	0.06	0.04	0.05	0.05
**Min-Max**	0.00 to 0.15	0.00 to 0.10	0.03 to 0.19	0.00 to 0.09	0.00 to 0.19	0.00 to 0.11
**Basophils at Day 7 (x10^9/L)**						
**n**	19	40	20	9	88	40
**Mean (SD)**	0.04 (0.03)	0.05 (0.03)	0.04 (0.03)	0.03 (0.03)	0.04 (0.03)	0.05 (0.03)
**Median**	0.03	0.04	0.03	0.04	0.04	0.05
**Min-Max**	0.00 to 0.09	0.02 to 0.11	0.00 to 0.15	0.00 to 0.07	0.00 to 0.15	0.01 to 0.15
**Monocytes at Baseline (x10^9/L)**						
**n**	20	40	19	10	89	40
**Mean (SD)**	0.39 (0.19)	0.35 (0.14)	0.36 (0.17)	0.30 (0.12)	0.36 (0.16)	0.37 (0.15)
**Median**	0.38	0.34	0.36	0.27	0.34	0.33
**Min-Max**	0.11 to 0.95	0.16 to 0.87	0.17 to 0.82	0.16 to 0.51	0.11 to 0.95	0.18 to 0.72
**Monocytes at Day 7 (x10^9/L)**						
**n**	19	40	20	9	88	40
**Mean (SD)**	0.41 (0.20)	0.36 (0.15)	0.34 (0.17)	0.32 (0.13)	0.36 (0.16)	0.36 (0.14)
**Median**	0.38	0.34	0.30	0.36	0.35	0.35
**Min-Max**	0.15 to 0.96	0.10 to 0.85	0.12 to 0.73	0.07 to 0.49	0.07 to 0.96	0.14 to 0.89
**Eosinophils at Baseline (x10^9/L)**						
**n**	20	40	19	10	89	40
**Mean (SD)**	0.14 (0.11)	0.17 (0.20)	0.20 (0.15)	0.15 (0.11)	0.17 (0.16)	0.14 (0.13)
**Median**	0.10	0.10	0.16	0.13	0.10	0.10
**Min-Max**	0.00 to 0.40	0.00 to 0.87	0.00 to 0.55	0.01 to 0.40	0.00 to 0.87	0.01 to 0.55
**Eosinophils at Day 7 (x10^9/L)**						
**n**	19	40	20	9	88	40

	Low Dose N= 20	Standard N= 40	High Dose N= 20	SC Injection N= 10	Combined N= 90	Control N= 40
**Mean (SD)**	0.14 (0.13)	0.16 (0.20)	0.16 (0.12)	0.18 (0.16)	0.16 (0.17)	0.14 (0.14)
**Median**	0.11	0.10	0.12	0.10	0.10	0.10
**Min-Max**	0.00 to 0.48	0.01 to 0.93	0.00 to 0.49	0.03 to 0.44	0.00 to 0.93	0.01 to 0.76

Program V_haem Date: 13APR2023 Unique Number: 8803

Note: values shown as 0.00 should be interpreted as less than 0.005

Table 8.1: Change from Baseline Hematology – Safety Population

	Low Dose N= 20	Standard N= 40	High Dose N= 20	SC Injection N= 10	Combined N= 90	Control N= 40
**Change in Hemoglobin (g/dL)**						
**n**	19	40	19	9	87	40
**Mean (SD)**	-0.02 (0.79)	-0.36 (0.48)	0.11 (0.73)	-0.38 (0.41)	-0.19 (0.64)	-0.03 (0.62)
**Median**	0.00	-0.40	0.10	-0.30	-0.20	-0.20
**Min-Max**	-1.30 to 1.70	-1.30 to 0.60	-1.30 to 1.80	-1.10 to 0.10	-1.30 to 1.80	-1.20 to 1.60
**Change in Erythrocytes (x10^12/L)**						
**n**	19	40	19	9	87	40
**Mean (SD)**	0.02 (0.28)	-0.13 (0.17)	0.02 (0.25)	-0.08 (0.12)	-0.06 (0.22)	-0.01 (0.21)
**Median**	0.00	-0.10	0.00	-0.10	-0.10	-0.05
**Min-Max**	-0.50 to 0.50	-0.40 to 0.30	-0.60 to 0.50	-0.30 to 0.10	-0.60 to 0.50	-0.40 to 0.50
**Change in Platelets (x10^9/L)**						
**n**	17	35	19	9	80	39
**Mean (SD)**	9.8 (50.65)	5.5 (44.91)	-2.8 (44.83)	-18.2 (66.88)	1.7 (48.67)	-0.3 (51.13)
**Median**	16.0	6.0	9.0	-20.0	7.0	10.0
**Min-Max**	-108.0 to 110.0	-80.0 to 168.0	-112.0 to 72.0	-104.0 to 67.0	-112.0 to 168.0	-187.0 to 88.0
**Change in Leukocytes (x10^9/L)**						
**n**	19	40	19	9	87	40
**Mean (SD)**	-0.12 (1.81)	-0.40 (1.21)	-0.98 (1.08)	0.01 (1.80)	-0.42 (1.42)	-0.43 (1.78)
**Median**	-0.40	-0.35	-0.70	-0.30	-0.50	-0.35
**Min-Max**	-4.10 to 3.80	-3.60 to 2.10	-3.50 to 0.80	-2.40 to 3.90	-4.10 to 3.90	-6.90 to 2.70
**Change in Lymphocytes (x10^9/L)**						
**n**	19	40	19	9	87	40
**Mean (SD)**	-0.25 (0.44)	-0.31 (0.44)	-0.47 (0.47)	-0.20 (0.62)	-0.32 (0.47)	-0.28 (0.56)
**Median**	-0.27	-0.34	-0.50	-0.13	-0.33	-0.20
**Min-Max**	-1.16 to 0.82	-1.43 to 0.76	-1.73 to 0.36	-1.42 to 0.77	-1.73 to 0.82	-1.32 to 1.40

	Low Dose N= 20	Standard N= 40	High Dose N= 20	SC Injection N= 10	Combined N= 90	Control N= 40
**Change in Neutrophils (x10^9/L)**						
**n**	19	40	19	9	87	40
**Mean (SD)**	0.14 (1.66)	-0.12 (1.08)	-0.43 (1.31)	0.05 (1.75)	-0.11 (1.34)	-0.14 (1.49)
**Median**	-0.14	-0.08	-0.25	-0.11	-0.11	-0.28
**Min-Max**	-4.03 to 3.68	-3.14 to 3.02	-2.83 to 2.92	-1.73 to 4.20	-4.03 to 4.20	-5.88 to 2.71
**Change in Basophils (x10^9/L)**						
**N**	19	40	19	9	87	40
**Mean (SD)**	-0.02 (0.04)	-0.00 (0.03)	-0.03 (0.02)	-0.01 (0.05)	-0.01 (0.04)	-0.00 (0.03)
**Median**	-0.01	0.00	-0.03	0.00	-0.01	0.00
**Min-Max**	-0.10 to 0.07	-0.07 to 0.09	-0.07 to 0.00	-0.09 to 0.07	-0.10 to 0.09	-0.07 to 0.09
**Change in Monocytes (x10^9/L)**						
**N**	19	40	19	9	87	40
**Mean (SD)**	0.01 (0.23)	0.01 (0.15)	-0.01 (0.17)	0.01 (0.16)	0.00 (0.17)	-0.02 (0.18)
**Median**	0.04	0.02	-0.01	-0.01	0.01	-0.03
**Min-Max**	-0.57 to 0.54	-0.33 to 0.38	-0.28 to 0.51	-0.25 to 0.28	-0.57 to 0.54	-0.43 to 0.59
**Change in Eosinophils (x10^9/L)**						
**N**	19	40	19	9	87	40
**Mean (SD)**	0.00 (0.07)	-0.00 (0.10)	-0.03 (0.18)	0.02 (0.14)	-0.00 (0.12)	0.01 (0.09)
**Median**	0.00	-0.01	-0.01	0.03	-0.01	0.01
**Min-Max**	-0.18 to 0.13	-0.27 to 0.27	-0.51 to 0.44	-0.18 to 0.31	-0.51 to 0.44	-0.28 to 0.25

Program V_haem02 Date: 13APR2023 Unique Number: 8804

Note: values shown as 0.00 should be interpreted as less than 0.005

Table 8.2: Hematology Normal Ranges – Safety Population

	Low Dose N= 20	Standard N= 40	High Dose N= 20	SC Injection N= 10	Combined N= 90	Control N= 40
**Hemoglobin at Baseline (g/dL)**						
**N**	20	40	19	10	89	40
**Above Normal Range**	0	1 (2.5%)	0	0	1 (1.1%)	2 (5.0%)
**Within Normal Range**	20 (100%)	39 (97.5%)	18 (90.0%)	10 (100%)	87 (96.7%)	38 (95.0%)
**Below Normal Range**	0	0	1 (5.0%)	0	1 (1.1%)	0
**Hemoglobin at Day 7 (g/dL)**						
**N**	19	40	20	9	88	40
**Above Normal Range**	0	0	0	0	0	2 (5.0%)
**Within Normal Range**	19 (95.0%)	39 (97.5%)	19 (95.0%)	9 (90.0%)	86 (95.6%)	37 (92.5%)
**Below Normal Range**	0	1 (2.5%)	1 (5.0%)	0	2 (2.2%)	1 (2.5%)
**Erythrocytes at Baseline (x10^12/L)**						
**N**	20	40	19	10	89	40
**Above Normal Range**	2 (10.0%)	2 (5.0%)	0	0	4 (4.4%)	5 (12.5%)
**Within Normal Range**	18 (90.0%)	38 (95.0%)	19 (95.0%)	10 (100%)	85 (94.4%)	35 (87.5%)
**Erythrocytes at Day 7 (x10^12/L)**						
**N**	19	40	20	9	88	40
**Above Normal Range**	2 (10.0%)	1 (2.5%)	0	0	3 (3.3%)	4 (10.0%)
**Within Normal Range**	17 (85.0%)	39 (97.5%)	20 (100%)	9 (90.0%)	85 (94.4%)	36 (90.0%)
**Platelets at Baseline (x10^9/L)**						
**N**	20	37	19	10	86	40
**Above Normal Range**	0	1 (2.5%)	1 (5.0%)	0	2 (2.2%)	0
**Within Normal Range**	20 (100%)	35 (87.5%)	18 (90.0%)	10 (100%)	83 (92.2%)	40 (100%)
**Below Normal Range**	0	1 (2.5%)	0	0	1 (1.1%)	0
**Platelets at Day 7 (x10^9/L)**						

	Low Dose N= 20	Standard N= 40	High Dose N= 20	SC Injection N= 10	Combined N= 90	Control N= 40
**N**	17	36	20	9	82	39
**Above Normal Range**	0	1 (2.5%)	0	0	1 (1.1%)	1 (2.5%)
**Within Normal Range**	17 (85.0%)	34 (85.0%)	19 (95.0%)	9 (90.0%)	79 (87.8%)	38 (95.0%)
**Below Normal Range**	0	1 (2.5%)	1 (5.0%)	0	2 (2.2%)	0
**Leukocytes at Baseline (x10^9/L)**						
**N**	20	40	19	10	89	40
**Within Normal Range**	19 (95.0%)	37 (92.5%)	17 (85.0%)	8 (80.0%)	81 (90.0%)	37 (92.5%)
**Below Normal Range**	1 (5.0%)	3 (7.5%)	2 (10.0%)	2 (20.0%)	8 (8.9%)	3 (7.5%)
**Leukocytes at Day 7 (x10^9/L)**						
**N**	19	40	20	9	88	40
**Within Normal Range**	16 (80.0%)	36 (90.0%)	16 (80.0%)	7 (70.0%)	75 (83.3%)	32 (80.0%)
**Below Normal Range**	3 (15.0%)	4 (10.0%)	4 (20.0%)	2 (20.0%)	13 (14.4%)	8 (20.0%)
**Lymphocytes at Baseline (x10^9/L)**						
**N**	20	40	19	10	89	40
**Above Normal Range**	2 (10.0%)	2 (5.0%)	3 (15.0%)	0	7 (7.8%)	6 (15.0%)
**Within Normal Range**	18 (90.0%)	38 (95.0%)	16 (80.0%)	10 (100%)	82 (91.1%)	34 (85.0%)
**Lymphocytes at Day 7 (x10^9/L)**						
**N**	19	40	20	9	88	40
**Above Normal Range**	1 (5.0%)	0	0	0	1 (1.1%)	2 (5.0%)
**Within Normal Range**	18 (90.0%)	39 (97.5%)	18 (90.0%)	9 (90.0%)	84 (93.3%)	37 (92.5%)
**Below Normal Range**	0	1 (2.5%)	2 (10.0%)	0	3 (3.3%)	1 (2.5%)
**Neutrophils at Baseline (x10^9/L)**						
**N**	20	40	19	10	89	40
**Within Normal Range**	15 (75.0%)	33 (82.5%)	16 (80.0%)	7 (70.0%)	71 (78.9%)	32 (80.0%)

	Low Dose<br>N= 20	Standard<br>N= 40	High Dose<br>N= 20	SC Injection<br>N= 10	Combined<br>N= 90	Control<br>N= 40
**Below Normal Range**	5 (25.0%)	7 (17.5%)	3 (15.0%)	3 (30.0%)	18 (20.0%)	8 (20.0%)
**Neutrophils at Day 7 (x10^9/L)**						
**N**	19	40	20	9	88	40
**Within Normal Range**	15 (75.0%)	33 (82.5%)	15 (75.0%)	6 (60.0%)	69 (76.7%)	28 (70.0%)
**Below Normal Range**	4 (20.0%)	7 (17.5%)	5 (25.0%)	3 (30.0%)	19 (21.1%)	12 (30.0%)
**Basophils at Baseline (x10^9/L)**						
**N**	20	40	19	10	89	40
**Above Normal Range**	0	0	1 (5.0%)	0	1 (1.1%)	0
**Within Normal Range**	20 (100%)	40 (100%)	18 (90.0%)	10 (100%)	88 (97.8%)	40 (100%)
**Basophils at Day 7 (x10^9/L)**						
**N**	19	40	20	9	88	40
**Within Normal Range**	19 (95.0%)	40 (100%)	20 (100%)	9 (90.0%)	88 (97.8%)	40 (100%)
**Monocytes at Baseline (x10^9/L)**						
**N**	20	40	19	10	89	40
**Above Normal Range**	1 (5.0%)	0	0	0	1 (1.1%)	0
**Within Normal Range**	18 (90.0%)	37 (92.5%)	18 (90.0%)	9 (90.0%)	82 (91.1%)	39 (97.5%)
**Below Normal Range**	1 (5.0%)	3 (7.5%)	1 (5.0%)	1 (10.0%)	6 (6.7%)	1 (2.5%)
**Monocytes at Day 7 (x10^9/L)**						
**N**	19	40	20	9	88	40
**Above Normal Range**	1 (5.0%)	0	0	0	1 (1.1%)	0
**Within Normal Range**	16 (80.0%)	39 (97.5%)	19 (95.0%)	8 (80.0%)	82 (91.1%)	39 (97.5%)
**Below Normal Range**	2 (10.0%)	1 (2.5%)	1 (5.0%)	1 (10.0%)	5 (5.6%)	1 (2.5%)
**Eosinophils at Baseline (x10^9/L)**						

	Low Dose<br>N= 20	Standard<br>N= 40	High Dose<br>N= 20	SC Injection<br>N= 10	Combined<br>N= 90	Control<br>N= 40
**N**	20	40	19	10	89	40
**Above Normal Range**	0	3 (7.5%)	0	0	3 (3.3%)	0
**Within Normal Range**	20 (100%)	37 (92.5%)	19 (95.0%)	10 (100%)	86 (95.6%)	40 (100%)
**Eosinophils at Day 7 (x10^9/L)**						
**N**	19	40	20	9	88	40
**Above Normal Range**	0	3 (7.5%)	0	0	3 (3.3%)	1 (2.5%)
**Within Normal Range**	19 (95.0%)	37 (92.5%)	20 (100%)	9 (90.0%)	85 (94.4%)	39 (97.5%)

Program V_haem03 Date: 13APR2023 Unique Number: 8805

13.3 IMMUNOGENICITY AND EFFICACY DATA

Table 2.2.1: Baseline Values of All Efficacy Parameters – mITT Population

	Low Dose N = 20	Standard N = 40	High Dose N = 20	SC Injection N= 10	Combined N = 90	Control N = 40
**GM Titer of ELISA BA.2 antibody (EC_{50})**						
**N**	20	39	20	9	88	39
**Geometric Mean (SD)**	555.7 (1.64)	561.9 (1.77)	630.5 (1.83)	722.0 (1.74)	590.3 (1.75)	518.0 (1.85)
**Median**	521.5	507.4	605.0	713.0	529.0	459.0
**Min-Max**	247.0 to 1350	248.0 to 2031	265.0 to 2224	333.0 to 1909	247.0 to 2224	243.0 to 2058
**GM Titer of ELISA Nucleocapsid antibody (OD450)**						
**N**	18	34	20	9	81	39
**Geometric Mean (SD)**	0.87 (2.98)	1.12 (2.62)	1.86 (2.02)	2.37 (1.60)	1.30 (2.57)	0.97 (4.07)
**Median**	1.05	1.44	2.34	2.70	1.80	1.80
**Min-Max**	0.05 to 3.38	0.10 to 3.50	0.19 to 3.62	0.85 to 3.50	0.05 to 3.62	0.01 to 3.56
**GM Titer of ELISA Spike 614G antibody (EC_{50})**						
**N**	8	9	0	0	17	15
**Geometric Mean (SD)**	522.6 (1.60)	782.5 (1.52)			647.2 (1.61)	677.9 (2.76)
**Median**	488.0	817.0			722.0	784.0
**Min-Max**	286.0 to 1210	365.0 to 1366			286.0 to 1366	50.0 to 2567
**GM Titer of Neutralizing BA.2 antibody (ID50)**						
**n**	9	11	0	0	20	16
**Geometric Mean (SD)**	532.0 (4.30)	734.2 (4.03)			635.1 (4.03)	483.3 (9.30)
**Median**	409.0	604.0			497.0	403.8
**Min-Max**	46.0 to 5358	157.0 to 13499			46.0 to 13499	20.0 to 9950

Program V_base Date: 27APR2023 Unique Number: 8841

Table 3.0.1: Change from Baseline of ELISA BA.2 Antibody Titer (EC_{50}) – Per Protocol Population

	Low Dose N = 20	Standard N = 40	High Dose N = 20	SC Injection N= 10	Combined N = 90	Control N = 40
**n**	11	20			31	21
**Change in GM Titer at Day 7**	-5.15	-64.52			-43.90	138.04
**n**	12	21			33	20
**Change in GM Titer at Day 14**	48.13	-43.06			-11.69	326.91
**n**	18	35	14	8	75	37
**Change in GM Titer at Day 28**	-19.02	-36.62	-22.01	46.52	-23.47	164.60
**n**	18	35	14	8	75	37
**Fold Change in GM Titer at Day 28**	0.97	0.93	0.96	1.06	0.96	1.31

Program V_effic07 Date: 27APR2023 Unique Number: 8842

Only participants with both a baseline value and a value at the respective visits are included in the calculation of change from baseline

Table 3.1.1: Change from Baseline of ELISA BA.2 Antibody Titer (EC_{50}) – mITT Population

	Low Dose N = 20	Standard N = 40	High Dose N = 20	SC Injection N= 10	Combined N = 90	Control N = 40
**n**	11	20			31	22
**Change in GM Titer at Day 7**	-5.15	-64.52	.	.	-43.90	135.20
**n**	12	21			33	21
**Change in GM Titer at Day 14**	48.13	-43.06	.	.	-11.69	320.22
**n**	20	39	17	8	84	38
**Change in GM Titer at Day 28**	81.07	-35.81	5.71	46.52	5.61	160.88
**n**	20	39	17	8	84	38
**Fold Change in GM Titer at Day 28**	1.15	0.94	1.01	1.06	1.01	1.31

Program V_effic01 Date: 27APR2023 Unique Number: 8843

Only participants with both a baseline value and a value at the respective visits are included in the calculation of change from baseline

Table 3.2.1: Change from Baseline of ELISA Nucleocapsid Antibody Titer (OD450) – mITT Population

	Low Dose N = 20	Standard N = 40	High Dose N = 20	SC Injection N= 10	Combined N = 90	Control N = 40
**n**	11	20			31	21
**Change in GM Titer at Day 7**	-0.05	-0.05	.	.	-0.05	0.02
**n**	12	21			33	20
**Change in GM Titer at Day 14**	-0.02	-0.02	.	.	-0.02	-0.01
**n**	20	39	17	8	84	37
**Change in GM Titer at Day 28**	0.08	-0.00	0.01	-0.03	0.02	0.06
**n**	20	39	17	8	84	37
**Fold Change in GM Titer at Day 28**	1.08	1.00	1.01	0.99	1.02	1.06

Program V_effic01 Date:27APR2023 Unique Number: 8844

Only participants with both a baseline value and a value at the respective visits are included in the calculation of change from baseline

Table 3.3.1: Geometric Mean Titer of ELISA-BA.2 Antibody (EC_{50}) by Study Visit – mITT Population

	Low Dose N = 20	Standard N = 40	High Dose N = 20	SC Injection N= 10	Combined N = 90	Control N = 40
**Geometric Mean Titer at Day 7**						
**n**	11	20	0	0	31	23
**Geometric Mean (SD)**	536.4 (1.73)	489.9 (1.65)			505.9 (1.66)	742.2 (1.63)
**Median**	473.0	413.7			449.0	787.0
**Min-Max**	256.0 to 1608	256.0 to 1543			256.0 to 1608	318.0 to 1588
**Geometric Mean Titer at Day 14**						
**n**	12	21	0	0	33	22
**Geometric Mean (SD)**	595.6 (1.66)	503.1 (1.77)			534.9 (1.73)	910.0 (1.86)
**Median**	563.6	405.0			474.0	790.1
**Min-Max**	287.0 to 1526	255.0 to 2492			255.0 to 2492	341.0 to 3859
**Geometric Mean Titer at Day 28**						
**n**	20	40	17	9	86	39
**Geometric Mean (SD)**	636.8 (1.66)	522.4 (1.65)	605.9 (1.51)	754.0 (1.90)	585.3 (1.66)	690.9 (1.76)
**Median**	642.2	476.4	649.0	801.0	578.5	679.0
**Min-Max**	259.0 to 2335	248.0 to 1785	273.0 to 1353	314.0 to 2756	248.0 to 2756	286.0 to 3555

Program V_effic01 Date: 27APR2023 Unique Number: 8845

Table 3.4.1: Geometric Mean Titer of ELISA-Nucleocapsid Antibody (OD450) by Study Visit – mITT Population

	Low Dose N = 20	Standard N = 40	High Dose N = 20	SC Injection N= 10	Combined N = 90	Control N = 40
**Geometric Mean Titer at Day 7**						
**n**	11	20	0	0	31	23
**Geometric Mean (SD)**	1.07 (2.17)	1.05 (2.62)			1.06 (2.43)	0.98 (3.40)
**Median**	1.34	1.45			1.35	1.54
**Min-Max**	0.33 to 2.82	0.22 to 3.23			0.22 to 3.23	0.04 to 3.16
**Geometric Mean Titer at Day 14**						
**n**	12	21	0	0	33	22
**Geometric Mean (SD)**	1.17 (2.27)	1.05 (2.64)			1.09 (2.48)	0.92 (3.37)
**Median**	1.64	1.22			1.49	1.47
**Min-Max**	0.29 to 2.94	0.19 to 3.48			0.19 to 3.48	0.02 to 2.94
**Geometric Mean Titer at Day 28**						
**n**	20	40	17	9	86	39
**Geometric Mean (SD)**	1.03 (2.44)	1.12 (2.63)	1.73 (2.11)	2.30 (1.58)	1.29 (2.45)	1.06 (3.23)
**Median**	1.25	1.45	2.17	2.88	1.72	1.68
**Min-Max**	0.21 to 3.53	0.14 to 3.55	0.19 to 3.67	0.85 to 3.47	0.14 to 3.67	0.03 to 3.45

Program V_effic01 Date: 27APR2023 Unique Number: 8846

Table 3.5: Clinical Assessment of COVID-19 by Study Day – mITT Population

	Low Dose N= 20	Standard N= 40	High Dose N= 20	SC Injection N= 10	Combined N= 90	Control N= 40
**COVID 19 Symptoms Present at Day 7**						
**n**	20	40	20	10	90	40
**Yes**	0	1 (2.5%)	2 (10.0%)	1 (10.0%)	4 (4.4%)	0
**No**	20 (100%)	39 (97.5%)	18 (90.0%)	9 (90.0%)	86 (95.6%)	40 (100%)
**Assessment of COVID 19 Severity**						
**n**	0	1	2	1	4	0
**0 : Uninfected; no viral RNA detected**		0	1 (5.0%)	1 (10.0%)	2 (2.2%)	
**2 : Symptomatic; independent**		1 (2.5%)	1 (5.0%)	0	2 (2.2%)	
**COVID 19 Symptoms Present at Day 14**						
**n**	20	40	19	10	89	39
**Yes**	0	2 (5.0%)	1 (5.0%)	1 (10.0%)	4 (4.4%)	2 (5.0%)
**No**	20 (100%)	38 (95.0%)	18 (90.0%)	9 (90.0%)	85 (94.4%)	37 (92.5%)
**Assessment of COVID 19 Severity**						
**n**	0	2	1	1	4	2
**0 : Uninfected; no viral RNA detected**		0	0	1 (10.0%)	1 (1.1%)	1 (2.5%)
**1 : Asymptomatic; viral RNA detected**		1 (2.5%)	0	0	1 (1.1%)	0
**2 : Symptomatic; independent**		1 (2.5%)	1 (5.0%)	0	2 (2.2%)	1 (2.5%)
**COVID 19 Symptoms Present at Day 28**						
**n**	20	40	18	9	87	39
**Yes**	0	1 (2.5%)	0	0	1 (1.1%)	0
**No**	20 (100%)	39 (97.5%)	18 (90.0%)	9 (90.0%)	86 (95.6%)	39 (97.5%)

	Low Dose N= 20	Standard N= 40	High Dose N= 20	SC Injection N= 10	Combined N= 90	Control N= 40
**Assessment of COVID 19 Severity**						
**n**	0	1	0	0	1	0
**0 : Uninfected; no viral RNA detected**		1 (2.5%)			1 (1.1%)	

Program V_c19 Date: 13APR2023 Unique Number: 8777

13.4 EXPLORATORY DATA

As noted above, these data were not collected due to amendments made between versions 4.0 and 5.0 of the protocol, owing to a lack of relevant humoral immune response at Study Day 28 in anti-SARS-CoV-2 BA.2 and anti-nucleocapsid antibodies in any of the study cohorts. A subset of samples with data on neutralizing antibody responses confirmed this observation. Therefore, the Sponsor terminated any further laboratory testing at other timepoints.

14 REFERENCE LIST

[1] Lyngse FP, Kirkeby CT, Denwood M, Christiansen LE, Mølbak K, Møller CH, et al. Transmission of SARS-CoV-2 Omicron VOC subvariants BA.1 and BA.2: Evidence from Danish Households. medRxiv 2022.01.28.22270044; doi: https://doi.org/10.1101/2022.01.28.22270044.

[2] Mammen Jr MP, Tebas P, Agnes, J, Giffear M, Kraynyak KA, Blackwood E, et al. Safety and immunogenicity of INO-4800 DNA vaccine against SARS-CoV-2: a preliminary report of a randomized, blinded, placebo-controlled, Phase 2 clinical trial in adults at high risk of viral exposure. medRxiv. 2021.05.07.21256652. doi: https://doi.org/10.1101/2021.05.07.21256652.

[3] World Health Organization (WHO). WHO Coronavirus (COVID-19) Dashboard. https://covid19.who.int/ (Accessed 06 June 2023)

[4] Chen J, Wei GW. Omicron BA.2 (B.1.1.529.2): high potential to becoming the next dominating variant. ArXiv. 2022 Feb 10:arXiv:2202.05031v1.

[5] Pavlin B. Epidemiology of Omicron variant of SARS-CoV-2. Available from: https://cdn.who.int/media/docs/default-source/epi-win/webinar-report-epi-win/presesntation_boris_pavlin.pdf?sfvrsn=4f5a18c0_5 (Accessed 14 March 2023)

[6] McIntosh, K. COVID-19: Epidemiology, virology, and prevention. Available from: https://www.uptodate.com/contents/covid-19-epidemiology-virology-and-prevention (Accessed 14 March 2023)

[7] CoVariants. Overview of Variants in Countries. Available from: https://covariants.org/per-country (Accessed 14 March 2023)

[8] Du P, Gao GF, Wang Q. The mysterious origins of the Omicron variant of SARS-CoV-2. Innovation (Camb). 2022;3(2):100206. doi:10.1016/j.xinn.2022.100206.

[9] Reuters. Omicron subvariant BA.2 likely to have same severity as "original" -WHO. Available from: https://www.reuters.com/business/healthcare-pharmaceuticals/omicron-subvariant-ba2-likely-have-same-severity-original-who-2022-02-01/ (Accessed 14 March 2023)

[10] Edward KM, Orenstein WA. COVID-19: Vaccines. Available from: https://www.uptodate.com/contents/covid-19-vaccines (Accessed 15 March 2023)

[11] Janssen Pharmaceutica (PTY) Ltd. Janssen Ad26.COV2.S COVID-19 Vaccine prescribing information. Updated 14 April 2022. Available from: https://www.covid19vaccinejanssen.com/za-en/download/7 (Accessed 19 May 2023).

[12] Lauring AS, Tenforde MW, Chappell JD, Gaglani M, Ginde AA, McNeal T et al. Clinical severity of, and effectiveness of mRNA vaccines against, covid-19 from omicron, delta, and alpha SARS-CoV-2 variants in the United States: prospective observational study BMJ 2022; 376 :e069761 doi:10.1136/bmj-2021-069761.

[13] Garcia-Beltran WF, Lam EC, St Denis K, Nitido AD, Garcia ZH, Hauser BM, et al. Multiple SARS-CoV-2 variants escape neutralization by vaccine-induced humoral immunity. Cell. 2021 Apr 29;184(9):2372-2383.e9. doi: 10.1016/j.cell.2021.03.013.

[14] Wilhelm A, Widera M, Grikscheit K, Toptan T, Schenk B, Pallas C, et al. Reduced neutralization of SARS-CoV-2 omicron variant by vaccine sera and monoclonal antibodies. medRxiv 2021.12.07.21267432; doi: https://doi.org/10.1101/2021.12.07.21267432.

[15] Hoffmann M, Krüger N, Schulz S, Cossmann A, Rocha C, Kempf A, et al. The Omicron variant is highly resistant against antibody-mediated neutralization: Implications for control of the COVID-19 pandemic. Cell. 2022;185(3):447-456.e11. doi: 10.1016/j.cell.2021.12.032.

[16] Gray GE, Collie S, Garrett N, Goga A, Champion J, Zylstra M, at al. Vaccine effectiveness against hospital admission in South African health care workers who received a homologous booster of Ad26.COV2 during an Omicron COVID19 wave: Preliminary Results of the Sisonke 2 Study. medRxiv 2021.12.28.21268436; doi: https://doi.org/10.1101/2021.12.28.21268436.

[17] Ritchie H, Mathieu E, Rodés-Guirao L, Appel C, Giattino C, Ortiz-Ospina E, et al. Coronavirus Pandemic (COVID-19). Available from: Our World in Data. https://ourworldindata.org/covid-vaccinations. (Accessed 19 May 2023)

[18] Capua I, Giaquinto C. The unsung virtue of thermostability. Lancet. 2021;397(10282):1346. doi: 10.1016/S0140-6736(21)00526-2.

[19] Shafaati M, Saidijam M, Soleimani M, Hazrati F, Mirzaei R, Amirheidari B, et al. A brief review on DNA vaccines in the era of COVID-19. Future Virol. 2021:10.2217/fvl-2021-0170. doi: 10.2217/fvl-2021-0170.

[20] World Health Organization (WHO). WHO Technical Report Series No 941, 2007. Annex 1. Guidelines for assuring the quality and nonclinical safety evaluation of DNA vaccines. 2007. Available from: https://cdn.who.int/media/docs/default-source/biologicals/vaccine-standardization/dna-vaccines/annex-1-dna-vaccines.pdf?sfvrsn=64ac71e3_4&download=true (Accessed 19 May 2023)

[21] Yang B, Jeang J, Yang A, Wu TC, Hung CF. DNA vaccine for cancer immunotherapy. Hum Vaccin Immunother. 2014;10(11):3153-64. doi: 10.4161/21645515.2014.980686.

[22] World Health Organization (WHO). Guidelines for assuring the quality, safety, and efficacy of plasmid DNA vaccines. Proposed revision of Annex 1 of WHO Technical Report Series, No. 941, 2020. 1 September 2020. Available from: https://cdn.who.int/media/docs/default-source/biologicals/ecbs/dna-post-ecbs-1-sept-2020.pdf?sfv (Accessed 19 May 2023)

[23] World Health Organization (WHO). Guidelines on the quality, safety and efficacy of plasmid DNA vaccines. Replacement of Annex 1 of WHO Technical Report Series, No. 941. Annex 2. 10 March 2021. Available from: https://cdn.who.int/media/docs/default-source/biologicals/vaccine-standardization/dna-vaccines/annex-2_dna_who_trs_1028_web-(1).pdf?sfvrsn=9b32c63b_9&download=true (Accessed 19 May 2023)

[24] Modjarrad K, Roberts CC, Mills KT, Castellano AR, Paolino K, Muthumani K, et al. Safety and immunogenicity of an anti-Middle East respiratory syndrome coronavirus DNA vaccine: a phase 1, open-label, single-arm, dose-escalation trial. Lancet Infect Dis. 2019 Sep;19(9):1013-1022. doi: 10.1016/S1473-3099(19)30266-X.

[25] Abbasi J. India's New COVID-19 DNA Vaccine for Adolescents and Adults Is a First. JAMA. 2021;326(14):1365. doi:10.1001/jama.2021.16625. Available from: https://jamanetwork.com/journals/jama/fullarticle/2784978 (Accessed 19 May 2023)

[26] Beasley DWC. New international guidance on quality, safety and efficacy of DNA vaccines. NPJ Vaccines. 2020;5(1):53. doi: 10.1038/s41541-020-0199-0.

[27] Sheets R, Kang HN, Meyer H, Knezevic I; WHO informal consultation on development of guidelines for assuring the quality, safety, and efficacy of DNA vaccine. WHO informal consultation on the guidelines for evaluation of the quality, safety, and

efficacy of DNA vaccines, Geneva, Switzerland, December 2019. NPJ Vaccines. 2020 Jun 18;5(1):52. doi: 10.1038/s41541-020-0197-2.

[28] Ahn JY, Lee J, Suh YS, Song YG, Choi YJ, Lee KH, et al. Safety and immunogenicity of two recombinant DNA COVID-19 vaccines containing the coding regions of the spike or spike and nucleocapsid proteins: an interim analysis of two open-label, non-randomised, phase 1 trials in healthy adults. Lancet Microbe. 2022;3(3):e173-e183. doi: 10.1016/S2666-5247(21)00358-X.

[29] AnGes, Inc. Novel Coronavirus (COVID-19) DNA Vaccine: Results of Phase 1/2 and Phase 2/3 Clinical Trials. 5 November 2021. Available from: https://www.anges.co.jp/pdf_news/public/cqFgW5KW0U5efAL9wJqhdQhRKCKPasRw.pdf (Accessed 14 March 2023)

[30] AnGes, Inc. Phase I/II Study of COVID-19 DNA Vaccine (AG0302-COVID19 High-dose). ClinicalTrials.gov Identifier: NCT04993586. Available from: https://clinicaltrials.gov/ct2/show/NCT04993586 (Accessed 14 March 2023)

[31] Momin T, Kansagra K, Patel H, Sharma S, Sharma B, Patel J, et al. Safety and Immunogenicity of a DNA SARS-CoV-2 vaccine (ZyCoV-D): Results of an open-label, non-randomized phase I part of phase I/II clinical study by intradermal route in healthy subjects in India. EclinicalMedicine. 2021;38:101020. doi: 10.1016/j.eclinm.2021.101020.

[32] Dey A, Chozhavel Rajanathan TM, Chandra H, Pericherla HPR, Kumar S, Choonia HS, et al. Immunogenic potential of DNA vaccine candidate, ZyCoV-D against SARS-CoV-2 in animal models. Vaccine. 2021;39(30):4108-4116. doi: 10.1016/j.vaccine.2021.05.098.

[33] Khobragade A, Bhate S, Ramaiah V, Deshpande S, Giri K, Phophle H, et al. Efficacy, safety, and immunogenicity of the DNA SARS-CoV-2 vaccine (ZyCoV-D): the interim efficacy results of a phase 3, randomised, double-blind, placebo-controlled study in India. Lancet. 2022 Apr 2;399(10332):1313-1321. doi: 10.1016/S0140-6736(22)00151-9.

[34] Tebas P, Yang S, Boyer JD, Reuschel EL, Patel A, Christensen-Quick A, et al. Safety and immunogenicity of INO-4800 DNA vaccine against SARS-CoV-2: A preliminary report of an open-label, Phase 1 clinical trial. EclinicalMedicine. 2021a;31:100689. doi: 10.1016/j.eclinm.2020.100689.

[35] Tebas P, Kraynyak KA, Patel A, Maslow JN, Morrow MP, Sylvester AJ, et al. Intradermal SynCon® Ebola GP DNA Vaccine Is Temperature Stable and Safely Demonstrates Cellular and Humoral Immunogenicity Advantages in Healthy Volunteers. J Infect Dis. 2019;220(3):400-410. doi: 10.1093/infdis/jiz132.

[36] Tebas P, Roberts CC, Muthumani K, Reuschel EL, Kudchodkar SB, Zaidi FI, et al. Safety and Immunogenicity of an Anti-Zika Virus DNA Vaccine. N Engl J Med. 2021b;385(12):e35. doi: 10.1056/NEJMoa1708120.

[37] Huang JH, Su QM, Yang J, Lv YH, He YC, Chen JC, et al. Sample sizes in dosage investigational clinical trials: a systematic evaluation. Drug Des Devel Ther. 2015;9:305-12. doi: 10.2147/DDDT.S76135.

[38] U.S. Food & Drug Administration. Development and Licensure of Vaccines to Prevent COVID-19 Guidance for Industry. June 2020. Docket Number: FDA-2020-D-1137. Available from: https://www.fda.gov/regulatory-information/search-fda-guidance-documents/development-and-licensure-vaccines-prevent-covid-19 (Accessed 23 March 2023)

[39] World Health Organization (WHO). Guidelines on clinical evaluation of vaccines: regulatory expectations. WHO Technical Report Series 1004, Annex 9, 2017. Available from: https://cdn.who.int/media/docs/default-source/prequal/vaccines/who-trs-1004-web-annex-9.pdf?sfvrsn=9c8f4704_2&download=true (Accessed 23 March 2023)

15 APPENDICES

The appendices available for this CSR are listed below.

Appendix number	Content
15.1	Study Information
15.1.1	Protocol and protocol amendments
15.1.2	Sample case report form
15.1.3	List of IECs or IRBs
15.1.4	List and description of Investigators and other important participants in the study
15.1.5	Signatures of Principal or Coordinating investigator(s) and/or Sponsor's responsible Medical Officer
15.1.6	List of Investigational Product(s) batch numbers
15.1.7	Randomization scheme and codes (participant identification and treatment assigned)
15.1.8	Audit certificates (if available)
15.1.9	Documentation of statistical methods
15.1.10	Documentation of inter-laboratory standardization methods and laboratory QA procedures if used
15.2	Participant Data Listings
15.3	Case Report Forms
15.3.1	CRFs for deaths, other SAEs and withdrawals for AE
