# Supplementary material for: Safety and immunogenicity of DNA omicron booster Alveavax-v1.2 in Ad26.COV2.S-vaccinated adults
Source: iScience. 2025 Nov 10;28(12):113970. doi: 10.1016/j.isci.2025.113970 (PMC12704268; doi:10.1016/j.isci.2025.113970)
Supplement: Data S2. Randomization codes [file mmc3.pdf]

Data S2: Randomization Codes

***UNBLINDED* Kit and Randomization Specification**	
**Client:** Alvea LLC / Telis Bioscience Inc.; Micron Research Ltd.	**Protocol:** Alvea-VAX-P00001
**Bioclinica Study Code:** ALV001	**Bioclinica IRT Version:** 2.5.10.02
**Specification version:** 2.0	

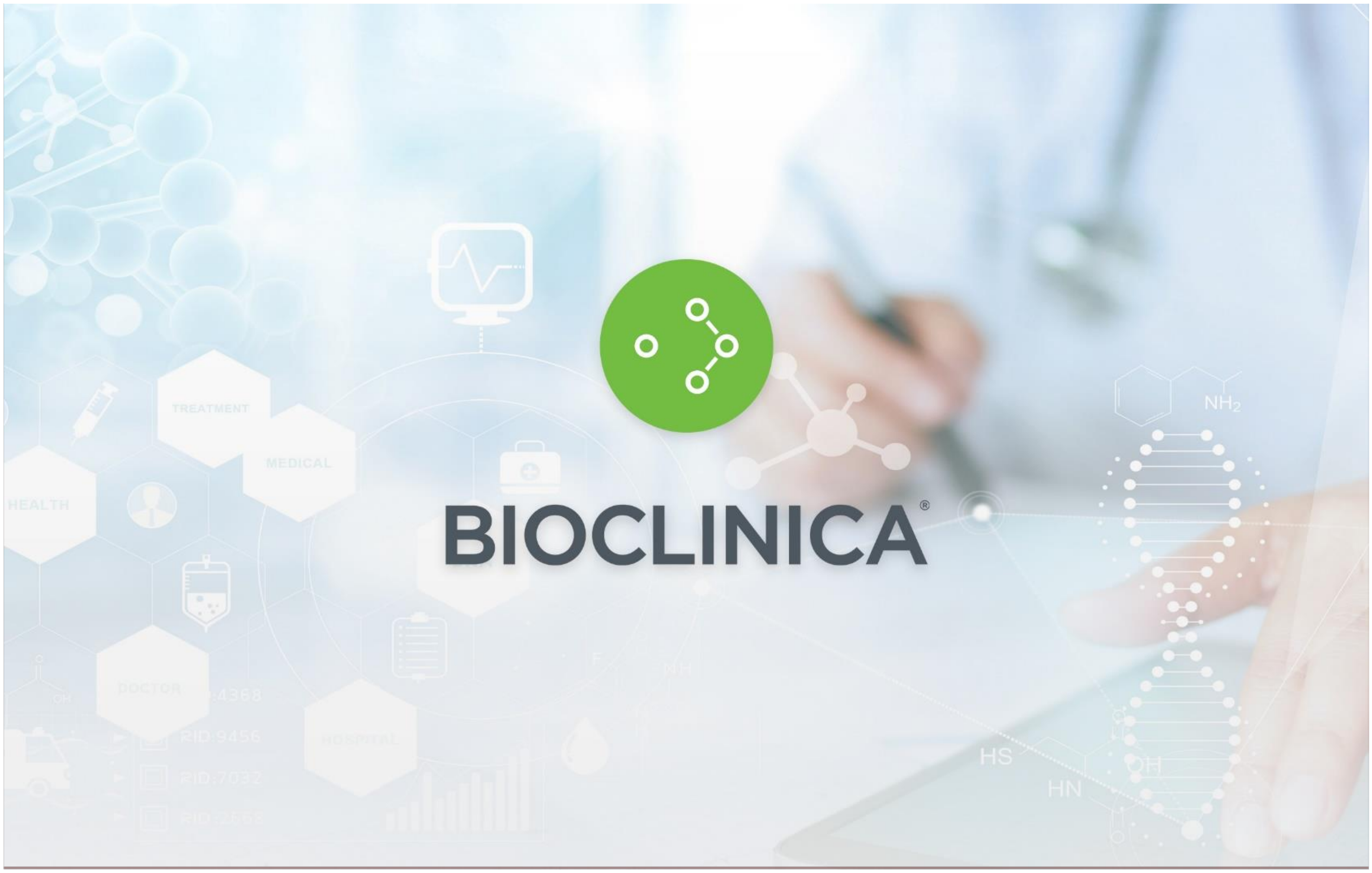

Kit and Randomization Specification (KARS)

Bioclinica – 211 Carnegie Center Drive, Princeton, NJ 08540 USA, +1 877-632-9432, bioclinica.com/contact

Table of Contents

1 General Information 3
1.1 Overview 3
2 Randomization List 3
2.1 Treatment Groups 3
2.2 Blocking Configuration 4
2.3 Randomization Balance and Additional Stratification Factors 4
2.4 Cohorts 5
2.5 Randomization List Management 6
3 Kit List 8
3.1 Kit Type 8
3.2 Kit List Management 8
4 Format and File Type 8
4.1 Randomization Lists 8
5 File Receipt/Distribution 9
5.1 Files Provided by Bioclinica 9
6 KARS Approval Signatures 10
6.1 Bioclinica Signatures 10
6.2 Alvea LLC / Telis Bioscience Inc.; Micron Research Ltd. Signatures 12
7 Revision History 13

1 General Information

1.1 Overview

The purpose of the Unblinded Kit and Randomization Specification (KARS) document is to outline the randomization and/or kit list requirements that will be used to build the Bioclinica IRT system for the Alvea LLC / Telis Biosciences Inc.; Micron Research Ltd. Alvea-VAX-P00001 study.

☐ **Kit and Randomization Specification is Unblinding**

☒ **Kit and Randomization Specification is NOT Unblinding (typically only applicable for Open Label studies)**

2 Randomization List

☒ **Bioclinica** Generated

☐ **Alvea / Micron Research, Ltd.** Generated ***Note:** *Dummy files for initial configuration may be generated by Bioclinica***

☐ Randomization is not applicable to study. Randomization List will not be used.

2.1 Treatment Groups

☐ Treatment Groups will not be used

#	Treatment Group Code	Treatment Group Description	Blind Status	Treatment Ratio (Overall)
2.1.1	1.a	Low Dose (0.5 mg Alveavax-v1.2 in one ID injection)	☒ **Open** ☐ **Blinded**	2
2.1.2	1.b	Standard Dose (2 mg Alveavax-v1.2 in one ID injection)	☒ **Open** ☐ **Blinded**	4
2.1.3	1.c	High Dose (8mg Alveavax-v1.2 in four ID injections)	☒ **Open** ☐ **Blinded**	2
2.1.4	1.d	SC Injection (8mg Alveavax-v1.2 in one SC injection)	☒ **Open** ☐ **Blinded**	1
2.1.5	1.e	Control (Janssen Ad26.COV2.S in one IM injection)	☒ **Open** ☐ **Blinded**	4
2.1.6	2.a	Standard Dose Unvaccinated (2 mg Alveavax-v1.2 in one ID injection)	☒ **Open** ☐ **Blinded**	N/A

Note: Randomization ratios will be specific to the specific cohort/group. See section 2.4 for further detail.

2.2 Blocking Configuration

☐ Blocking Configuration will not be used. Study does not support Randomization.

#	Block Size
2.2.1	☒ **Fixed**<br>☐ **Variable**<br>***Block size(s) should be representative of the number of treatment groups and desired treatment ratio (i.e. ratio of 2:2:1 would result in a minimal block size of 5).**<br>****Block size documented in section 2.5**

2.3 Randomization Balance and Additional Stratification Factors

NOTE: Blocking of the randomization list(s) will be configured within the IRT based on requirements defined as balance factors.

Randomization to be balanced based on the tables below:

#	Balance Factor	Balance Description	Sequence	Criteria
2.3.1	☒ **Yes**<br>☐ **No**	**Study**	N/A	N/A
2.3.2	☐ **Yes**<br>☒ **No**	**Region / Country Group**	N/A	N/A
2.3.3	☐ **Yes**<br>☒ **No**	**Country**	N/A	N/A
2.3.4	☐ **Yes**<br>☒ **No**	**Site**	N/A	N/A

☐ **Additional Stratification Factors are required and defined below:**

☒ **Additional Stratification Factors are NOT required.**

2.4 Cohorts

☐ Cohorts will not be used

#	Client Cohort Number	Cohort Description	Cap*	Treatment Ratio/Participant Allocation	IRT Cohort Number
2.4.1	1	Cohort 1: Vaccinated Group 1 (1.a/1.e)	10	1:1 (1.a = 5; 1.e = 5)	1
2.4.2	2	Cohort 2: Vaccinated Group 1 (1.b)	5	1 (1.b = 5)	2
2.4.3	3	Cohort 3: Vaccinated Group 1 (1.a/1.b/1.e)	85	3:7:7 (1.a = 15; 1.b = 35; 1.e = 35)	3
2.4.4	4	Cohort 4: Vaccinated Group 1 (1.c)	5	1 (1.c = 5)	4
2.4.5	5	Cohort 5: Vaccinated Group 1 (1.c/1.d)	25	3:2 (1.c = 15; 1.d = 10)	5
2.4.6	6	Cohort 6: Unvaccinated Group 2 (2.a)	20	1 (2.a = 20)	6

☐ **Cohorts being used for programmatic purposes only (uncommon)**

*** Note: Cohort Caps will be enforced via Subject Capping to utilize custom error messages for each cohort. See URS section 3.2 for details.**

Dependencies between cohorts:

- Do not start cohort 2 (1.b) before cohort 1 (1.a/1.e) is completely enrolled + safety approved by independent medical monitor / sponsor
- Do not start cohort 3 (1.a/1.b/1.e) and cohort 6 (2.a) before cohort 2 (1.b) is completely enrolled + safety approved by independent medical monitor / sponsor
- We might need to remove a group from cohort 3 (1.a/1.b/1.e) in case safety was not confirmed for this group (unlikely to happen). If a group will need to be removed, the appropriate rows in the Randomization List will be marked as Unavailable via a Parameter Change Request (PCR) submitted by Alvea LLC / Telis Biosciences Inc.; Micron Research Ltd..
- Do not start cohort 4 (1.c) before cohort 3 (1.a/1.b/1.e) started + sponsor approved to start cohort 4 (1.c)
- Do not start cohort 5 (1.c/1.d) before cohort 4 (1.c) is completely enrolled + safety approved by independent medical monitor / sponsor
- Cohort 1, 2, and 4 (all safety cohorts) will need to be enrolled at defined sites. This may be managed by Site Controls and/or Site Group configuration.

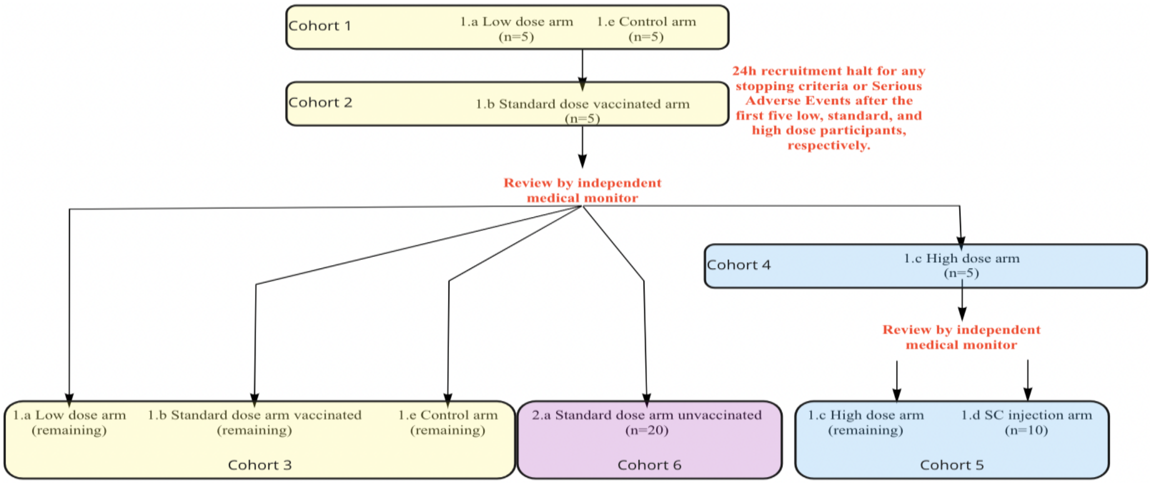

2.5 Randomization List Management

☐ Randomization List will not be used. Study does not support Randomization.

#	Rand List ID	Total Positions***	Randomization Numbers	Starting Position #	Ending Position #	Block Size	Cohort Association	Comments
2.5.1	Dummy 1	100	☒ Sequential ☐ Non-Sequential	9001	9100	2	1	N/A
2.5.2	Dummy 2	100	☒ Sequential ☐ Non-Sequential	9101	9200	1	2	Placeholder Rand List to support Open Enrollment to Treatment Group 1.b
2.5.3	Dummy 3	102	☒ Sequential ☐ Non-Sequential	9201	9302	17	3	N/A
2.5.4	Dummy 4	98	☒ Sequential ☐ Non-Sequential	9303	9400	1	4	Placeholder Rand List to support Open Enrollment to Treatment Group 1.c
2.5.5	Dummy 5	100	☒ Sequential ☐ Non-Sequential	9401	9500	5	5	N/A

#	Rand List ID	Total Positions***	Randomization Numbers	Starting Position #	Ending Position #	Block Size	Cohort Association	Comments
2.5.6	Dummy 6	100	☒ Sequential<br>☐ Non-Sequential	9501	9600	1	6	Placeholder Rand List to support Open Enrollment to Treatment Group 2.a
2.5.7	Live 1	20	☒ Sequential<br>☐ Non-Sequential	1001	1020	2	1	N/A
2.5.8	Live 2	10	☒ Sequential<br>☐ Non-Sequential	2001	2010	1	2	Placeholder Rand List to support Open Enrollment to Treatment Group 1.b
2.5.9	Live 3	170	☒ Sequential<br>☐ Non-Sequential	3001	3170	17	3	N/A
2.5.10	Live 4	10	☒ Sequential<br>☐ Non-Sequential	4001	4010	1	4	Placeholder Rand List to support Open Enrollment to Treatment Group 1.c
2.5.11	Live 5	50	☒ Sequential<br>☐ Non-Sequential	5001	5050	5	5	N/A
2.5.12	Live 6	40	☒ Sequential<br>☐ Non-Sequential	6001	6040	1	6	Placeholder Rand List to support Open Enrollment to Treatment Group 2.a

*__Note:__ ***Dummy and Live Randomization Lists should be unique with no overlapping randomization #s. These numbers should also be unique across randomization and kit lists if applicable***

__Note:__ ***If variable block sizes, all permitted variables must be identified. Any specifics details should be defined within the Comments.*

******Total Positions is the number of randomization records. If you are using stratification, you must make sure that the list is long enough to cover the maximum number of randomizations you expect within any stratum.***

3 Kit List

☐ **Bioclinica** Generated

☐ **Alvea / Micron Research, Ltd.** Generated ***Note:** ***Dummy files for initial configuration may be generated by Bioclinica.***

☒ Inventory Management is not applicable, or Study is using Bulk Supplies, Kit List will not be used.

3.1 Kit Type

☒ Kit Type will not be used

3.2 Kit List Management

☒ Kit List Management will not be used. Study is not using kits or is using bulk supplies.

4 Format and File Type

4.1 Randomization Lists

- File format must contain at minimum the following columns:
    - POSITION #
    - TG CODE
    - BLOCK ID
    - RAND #
    - COHORT
- It is preferred that additional columns are not included
- Files must be in the .csv file format

5 File Receipt/Distribution

5.1 Files Provided by Bioclinica

5.1.1 Dummy Files

- The dummy randomization lists will be sent to Tobias Odendahl, email: tobias.odendahl@alveavax.com, at Alvea LLC / Telis Bioscience Inc., for appropriate distribution, review and approval.
  - Dummy randomization lists may be sent to Maximilian Schons, email: maximilian.schons@alveavax.com, at Alvea LLC / Telis Bioscience Inc., for appropriate distribution, review and approval if the primary contact listed above is out of the office.
  - Dummy randomization lists may be sent to Sonia Sutherland, email: sonia.sutherland@micron-research.com, at Micron Research, Ltd., for appropriate distribution, review and approval if the primary contact listed above is out of the office.
- The files will be zipped and password protected. The passwords will be provided in a separate email.
- Recipient and any other designees will review dummy randomization list files and then provide approval, via the Bioclinica provided approval form, accepting that the files are correct per the requirements defined in this document.
- The dummy files will be used in the DEV, VAL and UAT environments.
- The following recipients may receive the dummy randomization list files during the course of UAT upon request:
  - Dummy randomization lists can be sent to Tobias Odendahl, email: tobias.odendahl@alveavax.com, at Alvea LLC / Telis Bioscience Inc., Maximilian Schons, email: maximilian.schons@alveavax.com, at Alvea LLC / Telis Bioscience Inc. and Sonia Sutherland, email: sonia.sutherland@micron-research.com, at Micron Research, Ltd.
    - Review of dummy randomization files prior to UAT approval is highly recommended by UAT file recipients for expected outcome of randomization file use following UAT data entry.

5.1.2 Live Files

- Once the dummy randomization lists have been approved, the live randomization schedule will be created.
- The live randomization lists ☒ will not be sent for approval.
- The live randomization schedule:
  ☒ can be sent via zipped password protected email to Tobias Odendahl, email: tobias.odendahl@alveavax.com, at Alvea LLC / Telis Bioscience Inc., for their documentation if requested.
  ☒ may be sent via zipped password protected email to Maximilian Schons, email: maximilian.schons@alveavax.com, at Alvea LLC / Telis Bioscience Inc. and/or Sonia Sutherland, email: sonia.sutherland@micron-research.com, at Micron Research, Ltd., for their documentation if the primary contact is out of the office.

6 KARS Approval Signatures

The signatures below indicate approval of this document and its attachments.

6.1 Bioclinica Signatures

**Specification Developer -Author**		**Specification Developer -Reviewer**	
**Name: Kellie Craven**<br>**Title:** Study Implementation Manager		**Name: Matthew Strutt**<br>**Title:** Sr. Solutions Engineer	
**Signature**	**Date**	**Signature**	**Date**
DocuSigned by:<br>Kellie Craven<br>Signer Name: Kellie Craven<br>Signing Reason: I am the author of this document<br>Signing Time: 22-Apr-2022 | 9:59 AM EDT<br>6FF65E8D71114EA49A0B9760A20658F3		DocuSigned by:<br>Matthew Strutt<br>Signer Name: Matthew Strutt<br>Signing Reason: I have reviewed this document<br>Signing Time: 22-Apr-2022 | 10:56 AM EDT<br>CBC559D786F44D1EA7642C3571E09770	
**Study Programming**		**Study QC**	
**Name: Aaron Gunter**<br>**Title:** Sr. Operations Programmer		**Name: Sujatha Pallem**<br>**Title:** Sr. Study QC Analyst	
**Signature**	**Date**	**Signature**	**Date**
DocuSigned by:<br>Aaron Gunter<br>Signer Name: Aaron Gunter<br>Signing Reason: I approve this document<br>Signing Time: 22-Apr-2022 | 11:22 AM EDT<br>F5FE510E7F594C6696D3C475673458B4		DocuSigned by:<br>Sujatha Pallem<br>Signer Name: Sujatha Pallem<br>Signing Reason: I approve this document<br>Signing Time: 22-Apr-2022 | 1:02 PM EDT<br>B76115A4BE474C888E22E8DFF2F1438E	

**RTSM Study Delivery**	
**Name: Kara Williams**<br><br>**Title**: Sr. Technical Project Manager	
**Signature**	**Date**
DocuSigned by:<br>Kara Williams<br>Signer Name: Kara Williams<br>Signing Reason: I approve this document<br>Signing Time: 22-Apr-2022 | 9:54 AM EDT<br>ACAFA07841F74AE68DDA383F64F767EB	

6.2 Alvea LLC / Telis Bioscience Inc.; Micron Research Ltd. Signatures

Electronic signatures must contain Printed Name, Date and Time of Signature, and Meaning of Signature.

Client Approver		Client Approver	
**Name: Maximilian Schons**<br><br>**Title**: Chief Medical Officer (Alvea LLC / Telis Bioscience Inc.)		**Name: Sonia Sutherland**<br><br>**Title**: Head of Clinical Operations (Micron Research, Ltd.)	
**Signature**	**Date**	**Signature**	**Date**
DocuSigned by:<br>Maximilian Schons<br>Signer Name: Maximilian Schons<br>Signing Reason: I approve this document<br>Signing Time: 22-Apr-2022 | 1:44 PM EDT<br>7B42018FD95D4D8285E06EB459AB93E2		DocuSigned by:<br>Sonia Sutherland<br>Signer Name: Sonia Sutherland<br>Signing Reason: I approve this document<br>Signing Time: 22-Apr-2022 | 3:13 PM BST<br>A5B7D38EB0854D8DB7B6A46A5E81A3C9	

7 Revision History

Version	Date	Revised By	Changes Made – Reason for Change
0.1	06Apr2022	Kellie Craven	Initial Draft
0.2	07Apr2022	Kellie Craven	Updated Alvea to Alvea LLC / Telis Bioscience Inc. throughout document.<br>2.4 – updated cohort descriptions; updated text regarding cohort dependencies<br>5.1.2 – updated for Live rand lists to remove option for will be sent for approval.
1.0	07Apr2022	Kellie Craven	Finalized for Approvals
1.1	08Apr2022	Kellie Craven	2.4.1 – updated typo
2.0	22Apr2022	Kellie Craven	6.1 – updated signatories and titles<br>Finalized for Approvals

Subject ID	Study Site	Investigator	Country	Last Visit	Rand Date	Rand ID	Treatment Group
101-001	101	Veronique de Jager	South Africa	Dosing Confirmation	13-Jul-2022	3001	Low Dose (0.5 mg Alveavax-v1.2 in one ID injection)
101-004	101	Veronique de Jager	South Africa	Dosing Confirmation	30-Jun-2022	1002	Low Dose (0.5 mg Alveavax-v1.2 in one ID injection)
101-005	101	Veronique de Jager	South Africa	Dosing Confirmation	30-Jun-2022	1001	Control (Janssen Ad26.COV2.S in one IM injection)
101-006	101	Veronique de Jager	South Africa	Dosing Confirmation	06-Jul-2022	1006	Control (Janssen Ad26.COV2.S in one IM injection)
101-008	101	Veronique de Jager	South Africa	Dosing Confirmation	13-Jul-2022	3002	Standard Dose (2 mg Alveavax-v1.2 in one ID injection)
101-015	101	Veronique de Jager	South Africa	Dosing Confirmation	27-Jul-2022	3037	Control (Janssen Ad26.COV2.S in one IM injection)
101-017	101	Veronique de Jager	South Africa	Dosing Confirmation	28-Jul-2022	3043	Standard Dose (2 mg Alveavax-v1.2 in one ID injection)
101-021	101	Veronique de Jager	South Africa	Dosing Confirmation	27-Jul-2022	3038	Low Dose (0.5 mg Alveavax-v1.2 in one ID injection)
101-022	101	Veronique de Jager	South Africa	Dosing Confirmation	04-Aug-2022	4001	High Dose (8mg Alveavax-v1.2 in four ID injections)
101-025	101	Veronique de Jager	South Africa	Dosing Confirmation	04-Aug-2022	4002	High Dose (8mg Alveavax-v1.2 in four ID injections)
101-026	101	Veronique de Jager	South Africa	Dosing Confirmation	04-Aug-2022	4003	High Dose (8mg Alveavax-v1.2 in four ID injections)
101-036	101	Veronique de Jager	South Africa	Dosing Confirmation	17-Aug-2022	4005	High Dose (8mg Alveavax-v1.2 in four ID injections)
101-037	101	Veronique de Jager	South Africa	Dosing Confirmation	17-Aug-2022	4004	High Dose (8mg Alveavax-v1.2 in four ID injections)
101-039	101	Veronique de Jager	South Africa	Dosing Confirmation	25-Aug-2022	5003	SC Injection (8mg Alveavax-v1.2 in one SC injection)
101-041	101	Veronique de Jager	South Africa	Dosing Confirmation	01-Sep-2022	5016	High Dose (8mg Alveavax-v1.2 in four ID injections)

101-044	101	Veronique de Jager	South Africa	Dosing Confirmation	31-Aug-2022	5013	High Dose (8mg Alveavax-v1.2 in four ID injections)
101-045	101	Veronique de Jager	South Africa	Dosing Confirmation	02-Sep-2022	5018	High Dose (8mg Alveavax-v1.2 in four ID injections)
101-046	101	Veronique de Jager	South Africa	Dosing Confirmation	25-Aug-2022	5002	SC Injection (8mg Alveavax-v1.2 in one SC injection)
101-049	101	Veronique de Jager	South Africa	Dosing Confirmation	25-Aug-2022	5005	High Dose (8mg Alveavax-v1.2 in four ID injections)
103-001	103	Madeli Kruger	South Africa	Dosing Confirmation	20-Jul-2022	3015	Low Dose (0.5 mg Alveavax-v1.2 in one ID injection)
103-003	103	Madeli Kruger	South Africa	Dosing Confirmation	20-Jul-2022	3016	Standard Dose (2 mg Alveavax-v1.2 in one ID injection)
103-004	103	Madeli Kruger	South Africa	Dosing Confirmation	28-Jul-2022	3048	Standard Dose (2 mg Alveavax-v1.2 in one ID injection)
103-009	103	Madeli Kruger	South Africa	Dosing Confirmation	28-Jul-2022	3044	Control (Janssen Ad26.COV2.S in one IM injection)
103-012	103	Madeli Kruger	South Africa	Dosing Confirmation	28-Jul-2022	3049	Standard Dose (2 mg Alveavax-v1.2 in one ID injection)
103-013	103	Madeli Kruger	South Africa	Dosing Confirmation	17-Aug-2022	3085	Low Dose (0.5 mg Alveavax-v1.2 in one ID injection)
103-015	103	Madeli Kruger	South Africa	Dosing Confirmation	03-Aug-2022	3057	Standard Dose (2 mg Alveavax-v1.2 in one ID injection)
103-016	103	Madeli Kruger	South Africa	Dosing Confirmation	17-Aug-2022	3084	Standard Dose (2 mg Alveavax-v1.2 in one ID injection)
103-017	103	Madeli Kruger	South Africa	Dosing Confirmation	25-Aug-2022	5007	SC Injection (8mg Alveavax-v1.2 in one SC injection)
103-018	103	Madeli Kruger	South Africa	Dosing Confirmation	05-Aug-2022	3064	Control (Janssen Ad26.COV2.S in one IM injection)
103-019	103	Madeli Kruger	South Africa	Dosing Confirmation	05-Aug-2022	3061	Standard Dose (2 mg Alveavax-v1.2 in one ID injection)
103-020	103	Madeli Kruger	South Africa	Dosing Confirmation	05-Aug-2022	3062	Control (Janssen Ad26.COV2.S in one IM injection)

103-021	103	Madeli Kruger	South Africa	Dosing Confirmation	05-Aug-2022	3063	Control (Janssen Ad26.COV2.S in one IM injection)
103-022	103	Madeli Kruger	South Africa	Dosing Confirmation	05-Aug-2022	3065	Control (Janssen Ad26.COV2.S in one IM injection)
103-025	103	Madeli Kruger	South Africa	Dosing Confirmation	17-Aug-2022	3083	Standard Dose (2 mg Alveavax-v1.2 in one ID injection)
103-030	103	Madeli Kruger	South Africa	Dosing Confirmation	02-Sep-2022	5019	SC Injection (8mg Alveavax-v1.2 in one SC injection)
103-032	103	Madeli Kruger	South Africa	Dosing Confirmation	25-Aug-2022	5008	High Dose (8mg Alveavax-v1.2 in four ID injections)
103-038	103	Madeli Kruger	South Africa	Dosing Confirmation	14-Sep-2022	5025	High Dose (8mg Alveavax-v1.2 in four ID injections)
103-039	103	Madeli Kruger	South Africa	Dosing Confirmation	14-Sep-2022	5024	High Dose (8mg Alveavax-v1.2 in four ID injections)
104-001	104	Madeli Kruger	South Africa	Dosing Confirmation	15-Jul-2022	3004	Control (Janssen Ad26.COV2.S in one IM injection)
104-002	104	Madeli Kruger	South Africa	Dosing Confirmation	15-Jul-2022	3005	Control (Janssen Ad26.COV2.S in one IM injection)
104-003	104	Madeli Kruger	South Africa	Dosing Confirmation	15-Jul-2022	3007	Control (Janssen Ad26.COV2.S in one IM injection)
104-005	104	Madeli Kruger	South Africa	Dosing Confirmation	15-Jul-2022	3008	Standard Dose (2 mg Alveavax-v1.2 in one ID injection)
104-007	104	Madeli Kruger	South Africa	Dosing Confirmation	15-Jul-2022	3009	Control (Janssen Ad26.COV2.S in one IM injection)
104-008	104	Madeli Kruger	South Africa	Dosing Confirmation	15-Jul-2022	3010	Control (Janssen Ad26.COV2.S in one IM injection)
104-010	104	Madeli Kruger	South Africa	Dosing Confirmation	15-Jul-2022	3006	Standard Dose (2 mg Alveavax-v1.2 in one ID injection)
104-014	104	Madeli Kruger	South Africa	Dosing Confirmation	21-Jul-2022	3023	Standard Dose (2 mg Alveavax-v1.2 in one ID injection)
104-016	104	Madeli Kruger	South Africa	Dosing Confirmation	21-Jul-2022	3025	Control (Janssen Ad26.COV2.S in one IM injection)

104-017	104	Madeli Kruger	South Africa	Dosing Confirmation	27-Jul-2022	3035	Standard Dose (2 mg Alveavax-v1.2 in one ID injection)
104-018	104	Madeli Kruger	South Africa	Dosing Confirmation	27-Jul-2022	3039	Standard Dose (2 mg Alveavax-v1.2 in one ID injection)
104-019	104	Madeli Kruger	South Africa	Dosing Confirmation	27-Jul-2022	3040	Control (Janssen Ad26.COV2.S in one IM injection)
104-021	104	Madeli Kruger	South Africa	Dosing Confirmation	29-Jul-2022	3054	Low Dose (0.5 mg Alveavax-v1.2 in one ID injection)
104-031	104	Madeli Kruger	South Africa	Dosing Confirmation	04-Aug-2022	3060	Control (Janssen Ad26.COV2.S in one IM injection)
104-032	104	Madeli Kruger	South Africa	Dosing Confirmation	29-Jul-2022	3051	Low Dose (0.5 mg Alveavax-v1.2 in one ID injection)
104-033	104	Madeli Kruger	South Africa	Dosing Confirmation	29-Jul-2022	3052	Standard Dose (2 mg Alveavax-v1.2 in one ID injection)
104-034	104	Madeli Kruger	South Africa	Dosing Confirmation	29-Jul-2022	3053	Low Dose (0.5 mg Alveavax-v1.2 in one ID injection)
104-035	104	Madeli Kruger	South Africa	Dosing Confirmation	10-Aug-2022	3070	Standard Dose (2 mg Alveavax-v1.2 in one ID injection)
104-036	104	Madeli Kruger	South Africa	Dosing Confirmation	10-Aug-2022	3071	Standard Dose (2 mg Alveavax-v1.2 in one ID injection)
104-038	104	Madeli Kruger	South Africa	Dosing Confirmation	12-Aug-2022	3074	Control (Janssen Ad26.COV2.S in one IM injection)
104-040	104	Madeli Kruger	South Africa	Dosing Confirmation	12-Aug-2022	3075	Standard Dose (2 mg Alveavax-v1.2 in one ID injection)
104-041	104	Madeli Kruger	South Africa	Dosing Confirmation	12-Aug-2022	3076	Standard Dose (2 mg Alveavax-v1.2 in one ID injection)
105-001	105	Friedrich Petrick	South Africa	Dosing Confirmation	03-Sep-2022	5020	High Dose (8mg Alveavax-v1.2 in four ID injections)
105-002	105	Friedrich Petrick	South Africa	Dosing Confirmation	01-Sep-2022	5017	SC Injection (8mg Alveavax-v1.2 in one SC injection)
106-002	106	Zaheer Hoosain	South Africa	Dosing Confirmation	06-Jul-2022	1007	Low Dose (0.5 mg Alveavax-v1.2 in one ID injection)

106-003	106	Zaheer Hoosain	South Africa	Dosing Confirmation	07-Jul-2022	1010	Low Dose (0.5 mg Alveavax-v1.2 in one ID injection)
106-004	106	Zaheer Hoosain	South Africa	Dosing Confirmation	08-Jul-2022	2004	Standard Dose (2 mg Alveavax-v1.2 in one ID injection)
106-005	106	Zaheer Hoosain	South Africa	Dosing Confirmation	08-Jul-2022	2005	Standard Dose (2 mg Alveavax-v1.2 in one ID injection)
106-006	106	Zaheer Hoosain	South Africa	Dosing Confirmation	08-Jul-2022	2003	Standard Dose (2 mg Alveavax-v1.2 in one ID injection)
106-007	106	Zaheer Hoosain	South Africa	Dosing Confirmation	08-Jul-2022	2002	Standard Dose (2 mg Alveavax-v1.2 in one ID injection)
106-009	106	Zaheer Hoosain	South Africa	Dosing Confirmation	08-Jul-2022	2001	Standard Dose (2 mg Alveavax-v1.2 in one ID injection)
106-010	106	Zaheer Hoosain	South Africa	Dosing Confirmation	14-Jul-2022	3003	Low Dose (0.5 mg Alveavax-v1.2 in one ID injection)
106-011	106	Zaheer Hoosain	South Africa	Dosing Confirmation	21-Jul-2022	3019	Control (Janssen Ad26.COV2.S in one IM injection)
106-013	106	Zaheer Hoosain	South Africa	Dosing Confirmation	22-Jul-2022	3028	Control (Janssen Ad26.COV2.S in one IM injection)
106-014	106	Zaheer Hoosain	South Africa	Dosing Confirmation	27-Jul-2022	3042	Standard Dose (2 mg Alveavax-v1.2 in one ID injection)
106-015	106	Zaheer Hoosain	South Africa	Dosing Confirmation	04-Aug-2022	3059	Standard Dose (2 mg Alveavax-v1.2 in one ID injection)
106-016	106	Zaheer Hoosain	South Africa	Dosing Confirmation	27-Jul-2022	3041	Control (Janssen Ad26.COV2.S in one IM injection)
106-017	106	Zaheer Hoosain	South Africa	Dosing Confirmation	03-Aug-2022	3055	Low Dose (0.5 mg Alveavax-v1.2 in one ID injection)
106-018	106	Zaheer Hoosain	South Africa	Dosing Confirmation	12-Aug-2022	3079	Control (Janssen Ad26.COV2.S in one IM injection)
106-019	106	Zaheer Hoosain	South Africa	Dosing Confirmation	10-Aug-2022	3066	Standard Dose (2 mg Alveavax-v1.2 in one ID injection)
106-020	106	Zaheer Hoosain	South Africa	Dosing Confirmation	10-Aug-2022	3067	Control (Janssen Ad26.COV2.S in one IM injection)

106-021	106	Zaheer Hoosain	South Africa	Dosing Confirmation	17-Aug-2022	3082	Control (Janssen Ad26.COV2.S in one IM injection)
106-022	106	Zaheer Hoosain	South Africa	Dosing Confirmation	17-Aug-2022	3081	Low Dose (0.5 mg Alveavax-v1.2 in one ID injection)
106-023	106	Zaheer Hoosain	South Africa	Dosing Confirmation	31-Aug-2022	5014	High Dose (8mg Alveavax-v1.2 in four ID injections)
106-024	106	Zaheer Hoosain	South Africa	Dosing Confirmation	31-Aug-2022	5015	High Dose (8mg Alveavax-v1.2 in four ID injections)
107-004	107	Zinhle Zwane	South Africa	Dosing Confirmation	05-Jul-2022	1003	Low Dose (0.5 mg Alveavax-v1.2 in one ID injection)
107-006	107	Zinhle Zwane	South Africa	Dosing Confirmation	05-Jul-2022	1004	Control (Janssen Ad26.COV2.S in one IM injection)
107-009	107	Zinhle Zwane	South Africa	Dosing Confirmation	06-Jul-2022	1005	Low Dose (0.5 mg Alveavax-v1.2 in one ID injection)
107-011	107	Zinhle Zwane	South Africa	Dosing Confirmation	06-Jul-2022	1008	Control (Janssen Ad26.COV2.S in one IM injection)
107-012	107	Zinhle Zwane	South Africa	Dosing Confirmation	06-Jul-2022	1009	Control (Janssen Ad26.COV2.S in one IM injection)
107-014	107	Zinhle Zwane	South Africa	Dosing Confirmation	15-Jul-2022	3013	Standard Dose (2 mg Alveavax-v1.2 in one ID injection)
107-015	107	Zinhle Zwane	South Africa	Dosing Confirmation	15-Jul-2022	3014	Control (Janssen Ad26.COV2.S in one IM injection)
107-017	107	Zinhle Zwane	South Africa	Dosing Confirmation	15-Jul-2022	3012	Standard Dose (2 mg Alveavax-v1.2 in one ID injection)
107-019	107	Zinhle Zwane	South Africa	Dosing Confirmation	15-Jul-2022	3011	Standard Dose (2 mg Alveavax-v1.2 in one ID injection)
107-020	107	Zinhle Zwane	South Africa	Dosing Confirmation	27-Jul-2022	3033	Low Dose (0.5 mg Alveavax-v1.2 in one ID injection)
107-022	107	Zinhle Zwane	South Africa	Dosing Confirmation	27-Jul-2022	3031	Standard Dose (2 mg Alveavax-v1.2 in one ID injection)
107-024	107	Zinhle Zwane	South Africa	Dosing Confirmation	21-Jul-2022	3026	Control (Janssen Ad26.COV2.S in one IM injection)

107-025	107	Zinhle Zwane	South Africa	Dosing Confirmation	21-Jul-2022	3024	Low Dose (0.5 mg Alveavax-v1.2 in one ID injection)
107-026	107	Zinhle Zwane	South Africa	Dosing Confirmation	21-Jul-2022	3027	Low Dose (0.5 mg Alveavax-v1.2 in one ID injection)
107-027	107	Zinhle Zwane	South Africa	Dosing Confirmation	21-Jul-2022	3022	Control (Janssen Ad26.COV2.S in one IM injection)
107-028	107	Zinhle Zwane	South Africa	Dosing Confirmation	21-Jul-2022	3021	Standard Dose (2 mg Alveavax-v1.2 in one ID injection)
107-030	107	Zinhle Zwane	South Africa	Dosing Confirmation	21-Jul-2022	3020	Control (Janssen Ad26.COV2.S in one IM injection)
107-032	107	Zinhle Zwane	South Africa	Dosing Confirmation	27-Jul-2022	3030	Control (Janssen Ad26.COV2.S in one IM injection)
107-033	107	Zinhle Zwane	South Africa	Dosing Confirmation	10-Aug-2022	3069	Control (Janssen Ad26.COV2.S in one IM injection)
107-036	107	Zinhle Zwane	South Africa	Dosing Confirmation	10-Aug-2022	3073	Control (Janssen Ad26.COV2.S in one IM injection)
107-038	107	Zinhle Zwane	South Africa	Dosing Confirmation	10-Aug-2022	3072	Low Dose (0.5 mg Alveavax-v1.2 in one ID injection)
107-040	107	Zinhle Zwane	South Africa	Dosing Confirmation	10-Aug-2022	3068	Standard Dose (2 mg Alveavax-v1.2 in one ID injection)
107-041	107	Zinhle Zwane	South Africa	Dosing Confirmation	12-Aug-2022	3078	Control (Janssen Ad26.COV2.S in one IM injection)
107-042	107	Zinhle Zwane	South Africa	Dosing Confirmation	31-Aug-2022	5012	SC Injection (8mg Alveavax-v1.2 in one SC injection)
107-043	107	Zinhle Zwane	South Africa	Dosing Confirmation	12-Aug-2022	3080	Control (Janssen Ad26.COV2.S in one IM injection)
107-044	107	Zinhle Zwane	South Africa	Dosing Confirmation	31-Aug-2022	5010	High Dose (8mg Alveavax-v1.2 in four ID injections)
107-045	107	Zinhle Zwane	South Africa	Dosing Confirmation	31-Aug-2022	5011	SC Injection (8mg Alveavax-v1.2 in one SC injection)
109-001	109	Salim Ahmed	South Africa	Dosing Confirmation	20-Jul-2022	3017	Control (Janssen Ad26.COV2.S in one IM injection)

109-002	109	Salim Ahmed	South Africa	Dosing Confirmation	20-Jul-2022	3018	Standard Dose (2 mg Alveavax-v1.2 in one ID injection)
109-003	109	Salim Ahmed	South Africa	Dosing Confirmation	03-Aug-2022	3058	Control (Janssen Ad26.COV2.S in one IM injection)
109-004	109	Salim Ahmed	South Africa	Dosing Confirmation	27-Jul-2022	3032	Standard Dose (2 mg Alveavax-v1.2 in one ID injection)
109-007	109	Salim Ahmed	South Africa	Dosing Confirmation	12-Aug-2022	3077	Standard Dose (2 mg Alveavax-v1.2 in one ID injection)
109-008	109	Salim Ahmed	South Africa	Dosing Confirmation	29-Jul-2022	3050	Standard Dose (2 mg Alveavax-v1.2 in one ID injection)
109-009	109	Salim Ahmed	South Africa	Dosing Confirmation	22-Jul-2022	3029	Standard Dose (2 mg Alveavax-v1.2 in one ID injection)
109-011	109	Salim Ahmed	South Africa	Dosing Confirmation	27-Jul-2022	3034	Standard Dose (2 mg Alveavax-v1.2 in one ID injection)
109-013	109	Salim Ahmed	South Africa	Dosing Confirmation	27-Jul-2022	3036	Control (Janssen Ad26.COV2.S in one IM injection)
109-015	109	Salim Ahmed	South Africa	Dosing Confirmation	03-Aug-2022	3056	Standard Dose (2 mg Alveavax-v1.2 in one ID injection)
109-017	109	Salim Ahmed	South Africa	Dosing Confirmation	28-Jul-2022	3045	Control (Janssen Ad26.COV2.S in one IM injection)
109-018	109	Salim Ahmed	South Africa	Dosing Confirmation	28-Jul-2022	3046	Low Dose (0.5 mg Alveavax-v1.2 in one ID injection)
109-019	109	Salim Ahmed	South Africa	Dosing Confirmation	28-Jul-2022	3047	Control (Janssen Ad26.COV2.S in one IM injection)
109-021	109	Salim Ahmed	South Africa	Dosing Confirmation	25-Aug-2022	5001	High Dose (8mg Alveavax-v1.2 in four ID injections)
109-022	109	Salim Ahmed	South Africa	Dosing Confirmation	25-Aug-2022	5004	High Dose (8mg Alveavax-v1.2 in four ID injections)
109-023	109	Salim Ahmed	South Africa	Dosing Confirmation	09-Sep-2022	5021	SC Injection (8mg Alveavax-v1.2 in one SC injection)
109-024	109	Salim Ahmed	South Africa	Dosing Confirmation	25-Aug-2022	5006	SC Injection (8mg Alveavax-v1.2 in one SC injection)

109-027	109	Salim Ahmed	South Africa	Dosing Confirmation	25-Aug-2022	5009	High Dose (8mg Alveavax-v1.2 in four ID injections)
109-028	109	Salim Ahmed	South Africa	Dosing Confirmation	14-Sep-2022	5023	High Dose (8mg Alveavax-v1.2 in four ID injections)
109-030	109	Salim Ahmed	South Africa	Dosing Confirmation	14-Sep-2022	5022	SC Injection (8mg Alveavax-v1.2 in one SC injection)

Alvea-VAX-

Position #	TG Code	Block ID	Rand #	Cohort
**1001**	1.e	1	1001	1
**1002**	1.a	1	1002	1
**1003**	1.a	2	1003	1
**1004**	1.e	2	1004	1
**1005**	1.a	3	1005	1
**1006**	1.e	3	1006	1
**1007**	1.a	4	1007	1
**1008**	1.e	4	1008	1
**1009**	1.e	5	1009	1
**1010**	1.a	5	1010	1
**1011**	1.e	6	1011	1
**1012**	1.a	6	1012	1
**1013**	1.a	7	1013	1
**1014**	1.e	7	1014	1
**1015**	1.e	8	1015	1
**1016**	1.a	8	1016	1
**1017**	1.e	9	1017	1
**1018**	1.a	9	1018	1
**1019**	1.a	10	1019	1
**1020**	1.e	10	1020	1

Alvea-VAX-

Position #	TG Code	Block ID	Rand #	Cohort
**2001**	1.b	1	2001	2
**2002**	1.b	2	2002	2
**2003**	1.b	3	2003	2
**2004**	1.b	4	2004	2
**2005**	1.b	5	2005	2
**2006**	1.b	6	2006	2
**2007**	1.b	7	2007	2
**2008**	1.b	8	2008	2
**2009**	1.b	9	2009	2
**2010**	1.b	10	2010	2

Alvea-VAX-

Position #	TG Code	Block ID	Rand #	Cohort
**3001**	1.a	1	3001	3
**3002**	1.b	1	3002	3
**3003**	1.a	1	3003	3
**3004**	1.e	1	3004	3
**3005**	1.e	1	3005	3
**3006**	1.b	1	3006	3
**3007**	1.e	1	3007	3
**3008**	1.b	1	3008	3
**3009**	1.e	1	3009	3
**3010**	1.e	1	3010	3
**3011**	1.b	1	3011	3
**3012**	1.b	1	3012	3
**3013**	1.b	1	3013	3
**3014**	1.e	1	3014	3
**3015**	1.a	1	3015	3
**3016**	1.b	1	3016	3
**3017**	1.e	1	3017	3
**3018**	1.b	2	3018	3
**3019**	1.e	2	3019	3
**3020**	1.e	2	3020	3
**3021**	1.b	2	3021	3
**3022**	1.e	2	3022	3
**3023**	1.b	2	3023	3
**3024**	1.a	2	3024	3
**3025**	1.e	2	3025	3
**3026**	1.e	2	3026	3
**3027**	1.a	2	3027	3
**3028**	1.e	2	3028	3
**3029**	1.b	2	3029	3

**3030**	1.e	2	3030	3
**3031**	1.b	2	3031	3
**3032**	1.b	2	3032	3
**3033**	1.a	2	3033	3
**3034**	1.b	2	3034	3
**3035**	1.b	3	3035	3
**3036**	1.e	3	3036	3
**3037**	1.e	3	3037	3
**3038**	1.a	3	3038	3
**3039**	1.b	3	3039	3
**3040**	1.e	3	3040	3
**3041**	1.e	3	3041	3
**3042**	1.b	3	3042	3
**3043**	1.b	3	3043	3
**3044**	1.e	3	3044	3
**3045**	1.e	3	3045	3
**3046**	1.a	3	3046	3
**3047**	1.e	3	3047	3
**3048**	1.b	3	3048	3
**3049**	1.b	3	3049	3
**3050**	1.b	3	3050	3
**3051**	1.a	3	3051	3
**3052**	1.b	4	3052	3
**3053**	1.a	4	3053	3
**3054**	1.a	4	3054	3
**3055**	1.a	4	3055	3
**3056**	1.b	4	3056	3
**3057**	1.b	4	3057	3
**3058**	1.e	4	3058	3
**3059**	1.b	4	3059	3
**3060**	1.e	4	3060	3
**3061**	1.b	4	3061	3

**3062**	1.e	4	3062	3
**3063**	1.e	4	3063	3
**3064**	1.e	4	3064	3
**3065**	1.e	4	3065	3
**3066**	1.b	4	3066	3
**3067**	1.e	4	3067	3
**3068**	1.b	4	3068	3
**3069**	1.e	5	3069	3
**3070**	1.b	5	3070	3
**3071**	1.b	5	3071	3
**3072**	1.a	5	3072	3
**3073**	1.e	5	3073	3
**3074**	1.e	5	3074	3
**3075**	1.b	5	3075	3
**3076**	1.b	5	3076	3
**3077**	1.b	5	3077	3
**3078**	1.e	5	3078	3
**3079**	1.e	5	3079	3
**3080**	1.e	5	3080	3
**3081**	1.a	5	3081	3
**3082**	1.e	5	3082	3
**3083**	1.b	5	3083	3
**3084**	1.b	5	3084	3
**3085**	1.a	5	3085	3
**3086**	1.e	6	3086	3
**3087**	1.e	6	3087	3
**3088**	1.b	6	3088	3
**3089**	1.a	6	3089	3
**3090**	1.b	6	3090	3
**3091**	1.b	6	3091	3
**3092**	1.a	6	3092	3
**3093**	1.e	6	3093	3

**3094**	1.b	6	3094	3
**3095**	1.b	6	3095	3
**3096**	1.e	6	3096	3
**3097**	1.e	6	3097	3
**3098**	1.a	6	3098	3
**3099**	1.e	6	3099	3
**3100**	1.b	6	3100	3
**3101**	1.e	6	3101	3
**3102**	1.b	6	3102	3
**3103**	1.b	7	3103	3
**3104**	1.a	7	3104	3
**3105**	1.b	7	3105	3
**3106**	1.b	7	3106	3
**3107**	1.b	7	3107	3
**3108**	1.e	7	3108	3
**3109**	1.b	7	3109	3
**3110**	1.a	7	3110	3
**3111**	1.b	7	3111	3
**3112**	1.e	7	3112	3
**3113**	1.e	7	3113	3
**3114**	1.e	7	3114	3
**3115**	1.e	7	3115	3
**3116**	1.a	7	3116	3
**3117**	1.e	7	3117	3
**3118**	1.b	7	3118	3
**3119**	1.e	7	3119	3
**3120**	1.e	8	3120	3
**3121**	1.a	8	3121	3
**3122**	1.e	8	3122	3
**3123**	1.e	8	3123	3
**3124**	1.b	8	3124	3
**3125**	1.b	8	3125	3

**3126**	1.e	8	3126	3
**3127**	1.e	8	3127	3
**3128**	1.b	8	3128	3
**3129**	1.b	8	3129	3
**3130**	1.e	8	3130	3
**3131**	1.b	8	3131	3
**3132**	1.a	8	3132	3
**3133**	1.a	8	3133	3
**3134**	1.e	8	3134	3
**3135**	1.b	8	3135	3
**3136**	1.b	8	3136	3
**3137**	1.a	9	3137	3
**3138**	1.a	9	3138	3
**3139**	1.e	9	3139	3
**3140**	1.e	9	3140	3
**3141**	1.b	9	3141	3
**3142**	1.e	9	3142	3
**3143**	1.e	9	3143	3
**3144**	1.e	9	3144	3
**3145**	1.a	9	3145	3
**3146**	1.b	9	3146	3
**3147**	1.e	9	3147	3
**3148**	1.b	9	3148	3
**3149**	1.e	9	3149	3
**3150**	1.b	9	3150	3
**3151**	1.b	9	3151	3
**3152**	1.b	9	3152	3
**3153**	1.b	9	3153	3
**3154**	1.a	10	3154	3
**3155**	1.e	10	3155	3
**3156**	1.e	10	3156	3
**3157**	1.e	10	3157	3

**3158**	1.e	10	3158	3
**3159**	1.b	10	3159	3
**3160**	1.b	10	3160	3
**3161**	1.e	10	3161	3
**3162**	1.b	10	3162	3
**3163**	1.e	10	3163	3
**3164**	1.b	10	3164	3
**3165**	1.b	10	3165	3
**3166**	1.b	10	3166	3
**3167**	1.e	10	3167	3
**3168**	1.b	10	3168	3
**3169**	1.a	10	3169	3
**3170**	1.a	10	3170	3

Alvea-VAX-

Position #	TG Code	Block ID	Rand #	Cohort
**4001**	1.c	1	4001	4
**4002**	1.c	2	4002	4
**4003**	1.c	3	4003	4
**4004**	1.c	4	4004	4
**4005**	1.c	5	4005	4
**4006**	1.c	6	4006	4
**4007**	1.c	7	4007	4
**4008**	1.c	8	4008	4
**4009**	1.c	9	4009	4
**4010**	1.c	10	4010	4

Alvea-VAX-

Position #	TG Code	Block ID	Rand #	Cohort
**5001**	1.c	1	5001	5
**5002**	1.d	1	5002	5
**5003**	1.d	1	5003	5
**5004**	1.c	1	5004	5
**5005**	1.c	1	5005	5
**5006**	1.d	2	5006	5
**5007**	1.d	2	5007	5
**5008**	1.c	2	5008	5
**5009**	1.c	2	5009	5
**5010**	1.c	2	5010	5
**5011**	1.d	3	5011	5
**5012**	1.d	3	5012	5
**5013**	1.c	3	5013	5
**5014**	1.c	3	5014	5
**5015**	1.c	3	5015	5
**5016**	1.c	4	5016	5
**5017**	1.d	4	5017	5
**5018**	1.c	4	5018	5
**5019**	1.d	4	5019	5
**5020**	1.c	4	5020	5
**5021**	1.d	5	5021	5
**5022**	1.d	5	5022	5
**5023**	1.c	5	5023	5
**5024**	1.c	5	5024	5
**5025**	1.c	5	5025	5
**5026**	1.d	6	5026	5
**5027**	1.c	6	5027	5
**5028**	1.c	6	5028	5
**5029**	1.c	6	5029	5

**5030**	1.d	6	5030	5
**5031**	1.c	7	5031	5
**5032**	1.d	7	5032	5
**5033**	1.c	7	5033	5
**5034**	1.c	7	5034	5
**5035**	1.d	7	5035	5
**5036**	1.c	8	5036	5
**5037**	1.d	8	5037	5
**5038**	1.d	8	5038	5
**5039**	1.c	8	5039	5
**5040**	1.c	8	5040	5
**5041**	1.c	9	5041	5
**5042**	1.d	9	5042	5
**5043**	1.d	9	5043	5
**5044**	1.c	9	5044	5
**5045**	1.c	9	5045	5
**5046**	1.d	10	5046	5
**5047**	1.c	10	5047	5
**5048**	1.d	10	5048	5
**5049**	1.c	10	5049	5
**5050**	1.c	10	5050	5

Alvea-VAX-

Position #	TG Code	Block ID	Rand #	Cohort
**6001**	2.a	1	6001	6
**6002**	2.a	2	6002	6
**6003**	2.a	3	6003	6
**6004**	2.a	4	6004	6
**6005**	2.a	5	6005	6
**6006**	2.a	6	6006	6
**6007**	2.a	7	6007	6
**6008**	2.a	8	6008	6
**6009**	2.a	9	6009	6
**6010**	2.a	10	6010	6
**6011**	2.a	11	6011	6
**6012**	2.a	12	6012	6
**6013**	2.a	13	6013	6
**6014**	2.a	14	6014	6
**6015**	2.a	15	6015	6
**6016**	2.a	16	6016	6
**6017**	2.a	17	6017	6
**6018**	2.a	18	6018	6
**6019**	2.a	19	6019	6
**6020**	2.a	20	6020	6
**6021**	2.a	21	6021	6
**6022**	2.a	22	6022	6
**6023**	2.a	23	6023	6
**6024**	2.a	24	6024	6
**6025**	2.a	25	6025	6
**6026**	2.a	26	6026	6
**6027**	2.a	27	6027	6
**6028**	2.a	28	6028	6
**6029**	2.a	29	6029	6

**6030**	2.a	30	6030	6
**6031**	2.a	31	6031	6
**6032**	2.a	32	6032	6
**6033**	2.a	33	6033	6
**6034**	2.a	34	6034	6
**6035**	2.a	35	6035	6
**6036**	2.a	36	6036	6
**6037**	2.a	37	6037	6
**6038**	2.a	38	6038	6
**6039**	2.a	39	6039	6
**6040**	2.a	40	6040	6
