# Supplementary material for: Safety and immunogenicity of DNA omicron booster Alveavax-v1.2 in Ad26.COV2.S-vaccinated adults
Source: iScience. 2025 Nov 10;28(12):113970. doi: 10.1016/j.isci.2025.113970 (PMC12704268; doi:10.1016/j.isci.2025.113970)
Supplement: Methods S1. Study protocol [file mmc4.pdf]

# Methods S1: Study Protocol

DocuSign Envelope ID: 2B72DF38-444D-4144-86F5-185A92D63B30

# Study protocol

**Alveavax-v1.2 Phase 1**

| | |
|---|---|
| **Latest Update** | 2022-11-16 9am CEST |
| **Protocol Number** | Alvea-VAX-P00001 |
| **Protocol Version** | 5.0 |
| **Previous Version** | 4.0 |
| **Substance** | Alveavax-v1.2 |
| **Scientific Title** | A Phase 1 open-label, active-controlled, randomized dose-finding study to evaluate safety, tolerability, and immunogenicity of intradermal and subcutaneous application of the plasmid DNA SARS-CoV-2 Omicron BA.2 vaccine Alveavax-v1.2 in primary Ad26.COV2.S vaccinated healthy individuals. |
| **Study Phase** | 1 |
| **Sponsor** | Grigory Khimulya, Chief Executive Officer of Telis Bioscience Inc. and Alvea, LLC<br>Telis Bioscience Inc.<br>19 Blackstone St, Cambridge MA, 02139 |
| **Study Centers** | Seven (+/- 3) sites in South Africa with access to local or centralized laboratories for protocol-mandated safety and immunogenicity evaluation |
| **Data Management and Biostatistics** | Micron Research Ltd. 109b Lancaster Business Park, Ely, CB6 3NX, UK. |
| **Independent medical monitor** | An independent medical monitor (IMM) and deputy IMM will be appointed for the duration of the study to evaluate, on an ongoing basis, the accumulating safety assessments to ensure the ongoing safety of study |


This document is confidential.

| | |
|---|---|
| **and internal review committee** | subjects. Based on the results of the monitoring activities, the central responsibility of an IMM is to make recommendations on further study conduct. |
| **Primary Registry and Trial Identifying Number** | South African National Clinical Trials Registry / DOH 27-062022-5157 |
| **Date of Registration** | 14 March 2022 |
| **Source(s) of Monetary or Material Support** | Telis Bioscience Inc. and Alvea, LLC |
| **Contact for Public Queries** | info@alveavax.com<br>Telis Bioscience Inc.<br>19 Blackstone St, Cambridge MA, 02139 |
| **Contact for Scientific Queries** | Maximilian Schons, MD<br>Chief Medical Officer<br>scientific-contact@alveavax.com<br>Telis Bioscience Inc.<br>19 Blackstone St, Cambridge MA, 02139 |
| **Public Title** | Safety, tolerability and ability to generate an immune response of Alveavax-v1.2, a BA.2/Omicron optimized DNA vaccine for the prevention of COVID-19 |
| **Countries of Recruitment** | South Africa |
| **Health Condition(s) or Problem(s) Studied** | Coronavirus disease 2019 (COVID-19) |
| **Interventions** | Intervention arm(s): Alveavax-v1.2:<br>• 2mg intradermal injection<br>• 0.5mg intradermal injection<br>• 8mg intradermal injection (4 x 2mg) |

| | |
|---|---|
| | • 8mg subcutaneous injection<br>Control arm:<br>• COVID-19 Vaccine Janssen (Ad26.COV2.S) |
| **Key Inclusion and Exclusion Criteria** | Inclusion:<br>• Healthy adult male and female volunteers between 18 and 65 years of age, inclusive.<br>• Participants who received a primary Janssen Ad26.COV2.S vaccine ≥ 60 days prior to receiving the study vaccine prior to receiving the study vaccination (Day 1) in this study.<br>Exclusion:<br>• Received any SARS-CoV-2 vaccination other than a single Janssen Ad26.COV2.S vaccine or plans to receive any additional SARS-CoV-2 vaccination within 90 days after the study vaccine administration (Day 1). |
| **Date of First Enrollment** | April 2022 |
| **Target Sample Size** | 130 |
| **Recruitment Status** | Enrolment completed |
| **Primary Outcome(s)** | To evaluate the safety and tolerability of Alveavax-v1.2 in healthy participants compared to a control booster vaccine |
| **Key Secondary Outcome(s)** | To evaluate the immunogenicity as humoral immune response against SARS-CoV-2 BA.2/Omicron after a booster dose of Alveavax-v1.2.<br>To evaluate the clinical efficacy against SARS-CoV-2 after a booster dose of Alveavax-v1.2.<br>To evaluate the success rate of intradermal injections. |

DocuSign Envelope ID: 2B72DF38-444D-4144-86F5-185A92D63B30

# CONFIDENTIALITY STATEMENT

The information contained in this document is the property of Alvea and Telis Bioscience and is provided to you in confidence as an investigator, potential investigator, or consultant, for review by you, your staff, and an applicable Institutional Review Board/Independent Ethics Committee. It is understood that this information will not be disclosed to others without written authorization from Alvea / Telis Bioscience, except to the extent necessary to obtain consent from those persons to whom the investigational product may be administered.

# Table of Contents

**Table of Contents** 5
List of Tables 10
**1. Protocol summary** 11
1.1. Synopsis 11
1.2 Schema 18
1.3 Schedule of Activities (SoA, Table 1) 19
1.4 Abbreviations (Table 2) 22
**2. Introduction** 25
2.1 Study Rationale 25
2.2 Background 25
2.2.1 Background on COVID-19 Omicron 25
2.2.2 Protection from Omicron in low- and middle-income countries 26
2.2.3 DNA vaccines 27
2.3 Risk/Benefit Assessment 28
2.3.1 Risk Assessment 28
2.3.2 Benefit Assessment 30
2.3.3 Overall Benefit/Risk Conclusion 30
**3. Objectives and Endpoints** 30
**4. Study Design** 33
4.1 Overall Design 33
4.2 Scientific Rationale for Study Design 34
4.3 Justification for Dose and Route of Administration 35
4.4 End of Study Definition 36
4.4.1 Termination of the Clinical Study 36
4.4.2 Study Stopping Criteria 36
**5. Study Population** 37
5.1 Number of Participants Planned 37
5.2 Inclusion Criteria 37
5.3 Exclusion Criteria 38
5.4 Lifestyle Considerations 39
5.5 Screen Failure 39
**6. Study Treatments / Investigational Product Management** 40
6.1 Study Intervention 40
6.1.1 Investigational Product 40


This document is confidential.

6.1.2 Control Vaccine 41
6.1.3 Study intervention(s) administered 41
6.2 Dosage and Treatment Schedule 42
Table 3: Dosing Schema 42
6.3 Supply and Administration of Investigational Product 42
6.4 Storage and Preparation 43
6.4.1 Storage 43
6.4.2 Preparation 43
6.4.3 Accountability 43
6.5 Measures to Minimize Bias: Randomization and Blinding 44
6.5.1 Procedures for Randomization and Stratification 44
6.5.2 Blinding 44
6.6 Study Vaccination Compliance 44
6.7 Concomitant and Prohibited Therapies 45
6.7.1 Concomitant Medication 45
6.7.2 Prohibited Therapies 45
6.8 Possible Vaccine Interactions 46

**7. Discontinuation of Study Vaccination and Participation Discontinuation/Withdrawal 46**
7.1 Discontinuation of Study Intervention 46
7.2 Participant Discontinuation/Withdrawal From the Study 47
7.3 Lost to Follow-Up 47

**8. Study Assessments and Procedures 48**
8.1 Study Procedures 48
8.1.1 Informed Consent Procedure 48
8.1.2 Medical History and Demographic Data 48
8.1.3 Physical Examination and Vital Signs 49
8.1.4 Nasopharyngeal (NP) Swab 49
8.1.5 Clinical Laboratory Evaluation 49
8.1.6 Diary and Measurement Devices 50
8.1.7 Telephonic Safety Follow-up 51
8.1.8 COVID-19 assessment 52
Table 4: COVID-19 Assessments 52
8.2 Visit Schedules 53
Screening Visit (within 28 days of enrollment) 53
Check-in (within 48 hours before dosing) 53
Dosing (Day 1) 53
Day 3 53

Day 7 (± 2 days) 54
Day 14 (± 2 days), Day 28 (± 2 days), and Day 84 (± 7 days) 54
Day 168 (± 14 days) (End-of-Study) 54
8.3 Safety Assessments 54
8.3.1 Abnormal Findings 55
8.4 Immunogenicity Assessments 55
8.4.1 Binding Antibody Assay 56
8.4.2 Virus Neutralization Assay 56
8.4.3 Fc assays 57
8.4.4 Analysis for Cellular Response 57
8.5 Adverse Events 57
8.5.1 Definitions 57
Adverse Events (AEs) 57
Treatment-Emergent Adverse Event (TEAE) 57
Solicited Adverse Events 58
Table 5: Solicited Local and General Adverse Events 58
Intensity of Solicited Adverse Events 58
Other adverse events 59
Unsolicited Adverse Events 59
Serious Adverse Events (SAEs) 59
Adverse Events of Special Interest (AESIs) 60
AEs Leading to Study Drug Withdrawal 60
8.5.2 Recording and Handling of Adverse Events 60
Table 6: Collection and Timelines for Reporting of Adverse Events (for the Investigative Site) 61
8.5.3 Documentation of Adverse Events 61
8.5.4 Evaluating Adverse Events 62
Severity 62
Causality 63
Outcome 63
8.5.5 Reporting Serious Adverse Events 64
Reporting Requirements 64
SAE Contact Information 64
Reporting of SAEs to Health Authorities and IRB 64
Discontinuation of the Study due to Adverse Events 65
8.5.6 Pregnancy 65
8.6 Adherence to Protocol 66
8.7 Protocol Deviations 67

8.8 Independent Medical Monitor 67

**9. Statistical considerations 67**

9.1 Statistical Hypotheses 67
9.2 Sample Size Determination 67
9.3 Populations for Analyses 68
9.3.1 Safety Population 68
9.3.2 Modified Intent-to-Treat Population (mITT) 68
9.3.3 Per-Protocol (PP) Population 68
9.4 Statistical Analysis 68
9.4.1 General Approach 68
9.4.2 Primary Endpoint Analysis 69
9.4.3 Secondary Endpoints Analysis 69
9.4.4 Safety and Tolerability Analysis 69
9.4.5 Baseline Characteristics 70
9.4.6 Exploratory Analyses 70
9.4.7 Analysis timepoints 71
9.5 Handling of Missing Data 71

**10. Administrative Matters 71**

10.1 Ethics 72
10.1.1 Ethical Conduct of the Study 72
10.1.2 Independent Ethics Committee Review and Communications 72
10.1.3 Informed Consent and Participant Information 72
10.1.4 Clinical Trial Insurance and Compensation to Participants 73
10.1.5 Participant Confidentiality and Disclosure 73
10.2 Records 74
10.2.1 Monitoring, Quality Assurance, and Inspection by Health Authorities 74
Monitoring 74
Quality Control and Quality Assurance 74
Audit and Inspection 74
10.2.2 Case-Report Forms 75
10.2.4 Recruitment Procedures 75
10.2.5 Source Documents 76
10.2.6 Direct Access to Source Data/Documents 76
10.2.7 Trial Monitoring 76
10.2.8 Investigator Site File and Archiving 76
10.2.9 Dissemination of Clinical Study Data 76
10.3 Quality Assurance Audit 78


This document is confidential.

DocuSign Envelope ID: 2B72DF38-444D-4144-86F5-185A92D63B30

10.4 Rules for Amending the Protocol 78
10.5 Discontinuation of the Trial by the Sponsor 78

**11. References 79**

**12. Signature Page 86**

**13. Appendices 88**
Appendix 1: Contraception 88
Appendix 2: Blood collection 88
Table 7: Blood Collection at Study Visit 89
Appendix 3: Toxicity Grading Scale 89
Table 8: Toxicity Grading Scale 89
Appendix 4: Composition of investigational product 98
Table 9: Composition of Investigational Product 98
**Appendix 5: Protocol amendments 99**


This document is confidential.

# List of Tables

*Table 1: 1.3 Schedule of Activities*
*Table 2: 1.4 Abbreviations*
*Table 3: Dosing Schema*
*Table 4: COVID-19 Assessments*
*Table 5: Solicited Local and General Adverse Events*
*Table 6: Collection and Timelines for Reporting of Adverse Events (for the Investigative Site)*
*Table 7: Blood Collection at Study Visit*
*Table 8: Toxicity Grading Scale*
*Table 9: Composition of Investigational Product*

# 1. Protocol summary

### 1.1. Synopsis

| | |
|---|---|
| **Protocol number** | Alvea-VAX-P00001 |
| **Investigational product** | Alveavax-v1.2 |
| **Title** | A Phase 1 open-label, active-controlled, randomized dose-finding study to evaluate safety, tolerability, and immunogenicity of intradermal and subcutaneous application of the plasmid DNA SARS-CoV-2 Omicron BA.2 vaccine Alveavax-v1.2 in primary Ad26.COV2.S vaccinated healthy individuals. |
| **Study Phase** | 1 |
| **Study Duration** | Approximately 10 months |
| **Objectives** | Primary:<br>● To evaluate the safety and tolerability of Alveavax-v1.2 from Alvea, LLC, in healthy participants, compared to a control booster vaccine.<br>Secondary:<br>● To evaluate the immunogenicity as humoral immune response against SARS-CoV-2 BA.2/Omicron after a booster dose of Alveavax-v1.2<br>● To evaluate the clinical efficacy against SARS-CoV-2 after a booster dose of Alveavax-v1.2<br>● To evaluate success rate of ID injections<br>Exploratory<br>● To evaluate the cell-mediated immune response against SARS-CoV-2 BA.2/Omicron after a booster dose of Alveavax-v1.2<br>● To evaluate fc effector functions against SARS-CoV-2 after a booster dose of Alveavax-v1.2<br>● To evaluate the humoral immune response against additional SARS-CoV-2 variants and sub-lineages after a booster dose of Alveavax-v1.2<br>● To correlate clinical efficacy with neutralizing antibody response<br>● To correlate clinical efficacy and immunogenicity with anti-nucleocapsid protein antibodies |


This document is confidential.

| | |
|---|---|
| **Characteristics and Number of Participants** | 130 healthy individuals (between 18 and 65 years of age, both inclusive), previously having received a primary Ad26.COV2.S vaccination series against SARS-CoV-2, satisfying all the eligibility criteria will be eligible to participate in the study. Screening for eligible participants will be performed within 28 days of vaccination in the study.<br><br>The full lists of inclusion and exclusion criteria are provided in the body of the protocol. |
| **Investigational Product, Dosage, and Route of Administration** | Alveavax-v1.2 is a preservative-free, sterile formulation of plasmid DNA in an isotonic Phosphate Buffer Saline. The plasmid DNA substance is the only active substance in this product. The product is a concentrate for injection and filled at 5 ± 0.5 mg/ml. Isotonic Phosphate Buffer Saline solution is sourced as an approved medicinal product.<br><br>The composition of the investigational product and the functions of the respective components are given in Appendix 4. More detailed information about the study drug can be found in the Investigator's Brochure (IB) Alveavax-v1.2.<br><br>Dose Level: 0.5 mg, 2 mg or 8 mg<br>Routes of Administration: ID and SC injection<br><br>The ID and SC injections will be given to the outer surface of the upper arm (or front of the thigh, or lower back). |
| **Study design** | This is a first in human, open-label, active-controlled, randomized dose-finding study to evaluate safety, tolerability, and immunogenicity of ID and SC application of the plasmid DNA SARS-CoV-2 Omicron BA.2 vaccine Alveavax-v1.2 in primary Ad26.COV2.S vaccinated healthy individuals.<br><br>Primary Ad26.COV2.S vaccinated participants will be randomized into one of 5 treatment arms to receive Alveavax-v1.2 or a Ad26.COV2.S control booster vaccine.<br><br>Participants will be enrolled at multiple sites in South Africa within 28 days after the initial screening to ensure they meet all the inclusion criteria and none of the exclusion criteria. |


This document is confidential.

| | |
|---|---|
| | Each participant will be administered a booster vaccine on Day 1 of the study and will be monitored afterwards as described below and in 8.3. Solicited local/systemic reactions will be recorded after vaccination in the participant's diary card for up to 7 days (the vaccine administration day and 6 days later) as described in 8.1.6. Adverse events (AE) as defined in 8.5, and concomitant medications as defined in 6.7 will be collected throughout the study.<br>A total of 130 male and female participants aged between 18 and 65 years who satisfy the inclusion and exclusion criteria are planned to be enrolled in five groups and with vaccine administered according to Table 3:<br><br>1. Individuals with a primary vaccination will get either:<br>a. **Low dose**: 0.5 mg Alveavax-v1.2 in one ID injection<br>b. **Standard dose**: 2 mg Alveavax-v1.2 in one ID injection<br>c. **High dose**: 8mg Alveavax-v1.2 in four ID injections*<br>d. **SC injection**: 8mg Alveavax-v1.2 in one SC injection<br>e. **Control**: Janssen Ad26.COV2.S in one IM injection<br><br>* Four ID injections shall be administered immediately one after the other, preferably at the same anatomic site (e.g. upper arm) with a few centimeters distance between each injection.<br><br>Dependent upon safety and/or immunogenicity data generated during the course of this study it is possible that groups may be started at the next highest dose, groups may not be started, groups may be terminated early, and/or groups may be added with dose levels below the lowest stated dose or intermediate between the lowest and highest stated doses.<br><br>The enrollment will start with the first five participants in the low dose (1.a) arm and the vaccinated standard dose (1.b) arm, followed by a review of 24h safety data by the independent medical monitor. Then, the remaining participants from the low and standard dose arms, as well as the control arm (1.e) will be recruited. In parallel, the first five participants of the high dose (1.c) arm are enrolled. Further recruitment and the SC injection arm (1.d) start as soon as the independent medical monitor has reviewed 24h safety data of the high dose arm (see schema in 1.2).<br><br>For each dose level the following apply:<br>● Additional safety assessments (see 8.3)<br>● Controlled enrollment (for new dose levels low / standard and high dose):<br>○ No more than 5 participants to be vaccinated on the first day |


This document is confidential.

| | |
|---|---|
| | ○ The first 5 participants must be observed for at least 4 hours after vaccination for any acute reactions<br>○ Vaccination of the remaining participants (participant 6 and above) will commence no sooner than 24 hours after the fifth participant received his or her vaccination<br>● Application of stopping rules (see 4.4.2)<br>● Escalation between dose levels will be allowed only after independent medical monitor review of at least 24 hours post–dose safety data in this study |
| **Intervention Groups** | (see table below) |
| **Randomization** | Following the enrollment and monitoring of the first 5 participants in the 1.a low dose arm, 1.e control arm and 1.b standard dose arm, the remaining participants in these arms will be randomly assigned into one of these arms.<br><br>Following the enrollment and monitoring of the first 5 participants in the 1.c high dose arm, the remaining participants in the high dose and SC injection arm will be randomly assigned into one of these arms.<br><br>Randomization will be performed centrally. Further detail will be provided in a separate randomization plan. |
| **Safety Assessments** | Safety assessments will include the following:<br>● Number of participants with solicited local and systemic AEs within 7 days of dose administration |

Intervention Groups:

| **Arms** | **No. of participants** | **Dose** | **Route** | **Dose administration** |
|---|---|---|---|---|
| **Group 1: Primary vaccinated individuals** | | | | |
| **1.A: Low dose** | 20 | 0.5 mg of Alveavax-v1.2 | ID | Single injection |
| **1.B: Standard dose** | 40 | 2 mg of Alveavax-v1.2 | ID | Single injection |
| **1.C: High dose** | 20 | 8 mg of Alveavax-v1.2 | ID | Four injections (4x 2 mg) |
| **1.D: SC injection** | 10 | 8 mg of Alveavax-v1.2 | SC | Single injection |
| **1.E: Control** | 40 | Control booster vaccine | IM | Single injection |


This document is confidential.

| | |
|---|---|
| | • Number of participants with unsolicited AEs within 28 days of vaccination<br>• Number of participants with any SAEs, adverse events of special interest (AESIs), and AEs leading to withdrawal during the entire period of study |
| **Immunogenicity Assessments** | Immunogenicity assessments will include the following:<br>• Change in geometric mean titer (GMT) of serum anti-SARS-CoV-2 BA.2 neutralizing antibody titers<br>• GMT of serum anti-SARS-CoV-2 BA.2 neutralizing antibody titers<br>• Geometric mean fold rise (GMFR) of serum anti-SARS-CoV-2 BA.2 neutralizing antibody<br>• Four-fold increase rate of anti-SARS-CoV-2 BA.2 neutralizing antibody<br>• Change in GMT of anti-spike protein (S) immunoglobulin G (IgG) antibody<br>• GMT of BA.2 anti-spike protein (S) IgG antibody<br>• GMFR of BA.2 anti-spike protein (S) IgG<br>• BA.2 Spike-specific CD4+ T-cell response<br>• Change in BA.2 spike-specific CD4+ T-cell response<br>• BA.2 Spike-specific CD8+ T-cell response<br>• Change in BA.2 spike-specific CD8+ T-cell response<br>• Activity against Omicron/BA.2, Ancestral variants (such as Alpha, Beta, Delta), and additional Omicron Subvariants (such as BA.1) as change in relative light units (RLU), and RLU of Antibody dependent cellular cytotoxicity (ADCC), Antibody dependent cellular phagocytosis (ADCP), Antibody dependent cellular trogocytosis, Complement deposition, Fc dimer receptor binding<br>• Characterization of humoral immune response of booster vaccinations against Ancestral, existing and yet to be defined variants (such as Alpha, Beta, Delta), and existing and yet to be defined sub-lineage (such as Omicron BA.1) SARS-CoV-2 strains measured using by change in GMT, GMT, and GMFR of serum anti-SARS-CoV-2 neutralizing antibodies, serum anti-S IgG antibodies, serum anti-S-RBD IgG antibodies |
| **Efficacy Assessments** | Efficacy assessments will include the following:<br>• Clinical efficacy measured using the WHO clinical progression scale for COVID-19<br>• Absolute number and fraction of ID injections which generated a $\geq 1$ mm and $\geq 7$ mm in diameter clearly demarcated bleb, clearly visible for |


This document is confidential.

| | |
|---|---|
| | at least 20 seconds, for 0.5 mg and 2 mg Alveavax-v1.2 respectively<br>● Percentage of participants with neutralization IC50>64 IU/mL against the respective strain<br>● Percentage of participants with neutralization IC50>128 IU/mL against the respective strain<br>● Serologic change in GMT and GMFR between baseline and other blood samples for anti-nucleocapsid protein (N) IgG antibodies |
| **Sample Size** | Due to a number of exploratory analyses built into the study design, we used a heuristic approach to select group sample sizes sufficient to assess safety across the five dosing groups and explore immunogenicity of the vaccine under study compared with the active control vaccine.<br>40 is the median group size in a recent systematic analysis of clinical trial sample sizes in viral diseases.<br><br>Groups of 40 participants each will be enrolled in the standard dose and control arms of vaccinated individuals to assess safety and variability in immune response relative to baseline immunogenicity. Forty is the median group size in a recent systematic analysis of clinical trial sample sizes in viral diseases[52].<br><br>The safety and immune response of the standard dose in vaccinated individuals will be compared with groups of 20 participants each in the low dose and high dose arm in vaccinated individuals.<br><br>Ten participants will be enrolled to assess safety and confirm seroconversion for SC injections. |
| **Statistical Methods** | Since the study is a phase 1 study primarily assessing safety, all data will be analyzed descriptively without a formal statistical hypothesis.<br><br>**Primary endpoints**<br>The overall number of patients and incidence proportion (number of participants experiencing any AEs by the total number of participants) of any solicited local and systemic AEs within seven days of dose administration will be presented. The incidence of each solicited AE (at MedDRA preferred term - PT and System Organ Class - SOC level) will also be shown.<br><br>The overall number and incidence proportion of unsolicited AEs within 28 days of vaccination will be analyzed in a similar fashion. |

| | |
|---|---|
| | SAEs, AESIs, and AEs leading to participant discontinuation will be presented in the form of a listing, with number and proportion for each one. In addition, the overall incidence proportion of these categories of AE will be shown.<br><br>For patient level tabulation a maximum intensity and highest relationship to investigational drug will be presented in separate tables.<br><br>**Secondary endpoints**<br>Description of GMT, change in GMT, and GMFR of serum anti-spike protein (S) IgG antibody titers and the change in GMT and GFR of anti-N protein (N) IgG antibody at Day 28.<br><br>The number and percentage of participants being found to be SARS-CoV-2 positive by external testing (score 1-10), number and percentage of participants with ambulatory mild disease (score 1-3), number and percentage of participants hospitalized with moderate disease (score 4-5), number and percentage of participants hospitalized with severe disease (score 6-9), number and percentage of participants who died (score 10) while being SARS-CoV-2 positive with the exact 95% CI will also be presented separately for each day: Day 7, Day 14, Day 28, Day 84, Day 168.<br><br>**Exploratory analysis**<br>Characterization of humoral antibody immune response of booster vaccinations against Ancestral and Variant (Alpha, Beta, Delta, BA.1, and any newly discovered VOC) SARS-CoV-2 Strains measured using GMT of anti-SARS-CoV-2 neutralizing antibody at each timepoint (Pre-vaccination, Day 7, Day 14, Day 28, Day 84, and Day 168) will be made using the approach described for the primary endpoint analyses.<br><br>Characterization of the cellular immune response following booster vaccinations against SARS-CoV-2 BA.2/Omicron measured using cellular immune responses CD4+ and CD8+ T-cell response.<br><br>The RLU and change in RLU of serum antibody-dependent cell-mediated cytotoxicity (ADCC) against existing and yet to be defined variants and sub-lineages at the defined time points will be presented unadjusted in tabular form. |

## 1.2 SCHEMA

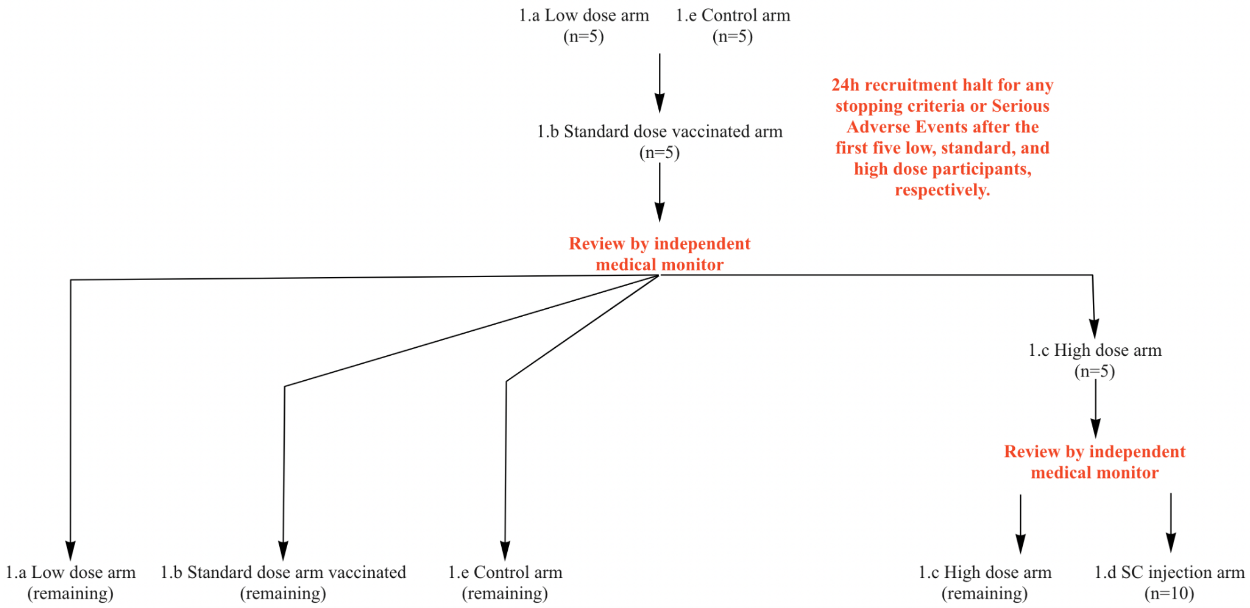


*Note: The enrollment will start with the first ten participants randomized into the low dose (1.a) arm, the control arm (1.e), subsequently at least 24 hours apart five participants to the vaccinated standard dose (1.b) arm, followed by a review of 24h safety data by the independent medical monitor. Then, the remaining participants assigned to the low and standard dose cohorts, in addition to those assigned to the control arm (1.e) will be recruited. In parallel, the first five participants of the high dose (1.c) arm will be enrolled. Further recruitment and the SC injection arm (1.d) start as soon as the independent medical monitor has reviewed 24h safety data of the high dose arm.*


This document is confidential.

## 1.3 Schedule of Activities (SoA, Table 1)

| Sr. No. | Assessment | Screen - ing | Enrollment and vaccine administration | | Follow-up Period | | | |
|---|---|---|---|---|---|---|---|---|
| | | D-28 to D-1 | Check In[G] | Dosing (D1) | D3 (call)[A] | D7 ± 2d | D14 ±2d, D28 ±2d, D84 ±7d | D168 ±14d (EOS) |
| | VISIT # | V-1 | V1 | | V2 | V3 | V4, 5, 6 | V7 |
| 1. | Informed consent process | X | | | | | | |
| 2. | Inclusion / Exclusion criteria | X | X | | | | | |
| 3. | Demographics (age, sex, height, and weight) | X | | | | | | X (Weight) |
| 4. | Medical history | X | X[F] | | | | | X |
| 5. | Provision of emergency contact card, thermometer, and measuring device/ruler | | X | | | | | |
| 6. | Randomization | | | X | | | | |
| Safety Assessments | | | | | | | | |
| 7. | Physical examination[E] | X | X | | | X | | X |
| 8. | Vital signs | X | X | X[D] | | X | | X |

| | | | | | | | | |
|---|---|---|---|---|---|---|---|---|
| 9. | Adverse Events Assessment | X | X | X | X | X | X | X |
| 10. | COVID-19 Assessment | | | | | X | X | X |
| Laboratory Assessments | | | | | | | | |
| 11. | Hematology | X | X[C] | | | X | | |
| 12. | Chemistry | X | X[C] | | | X | | |
| 13. | Urine pregnancy test | | X[B] | | | | X | |
| 14. | Serology | X | | | | | | |
| 15. | Blood sample for immunology | | X | | | X | X | X |
| 16. | Nasopharyngeal swab sample for PCR for SARS-CoV-2 | | X | | | X | X[H] | |
| Other Procedures | | | | | | | | |
| 17. | Vaccination | | | X | | | | |
| 18. | Participant diary card issue and / or review | | | X | | X | | |
| 19. | Inquire pregnancy[I], COVID-19 infections and vaccinations | | | | | | X | X |

A. Telephonic safety follow-up will be performed on day 3.
B. Urine pregnancy test will be performed for female participants of childbearing potential on screening, pre-dose, on day 28, and on day 84.


This document is confidential.

C. Clinical lab investigations will be performed if more than 7 days have passed since the screening.
D. Vitals (including blood pressure, pulse rate, and temperature) will be performed at pre-dose and 1, 2, 3, and 4 hours (±30 minutes) post-dose for the first 5 participants of each dose level; for all other participants at pre-dose and 30 minutes post-dose.
E. A history-directed physical examination will be done at baseline, followed by an abbreviated, symptom-directed physical examination at subsequent time points.
F. Medical history will be updated from the screening visit.
G. Check-in must be no more than 48 hours prior to dosing.
H. Nasopharyngeal (NP) swab sample on Day 14 and 28 only.
I. Confirm with female participants of childbearing potential and male participants who have a female partner if they/their partner have become pregnant since Day 1 of the study.


This document is confidential.

## 1.4 Abbreviations (Table 2)

| | |
|---|---|
| AE | Adverse Event |
| AESI | Adverse Event of Special Interest |
| ALT | Alanine Transaminase |
| AST | Aspartate Transaminase |
| AV | Audio Video |
| BMI | Body Mass Index |
| BUN | Blood Urea Nitrogen |
| COVID-19 | Coronavirus Disease 2019 |
| CRF | Case Report Form |
| CRO | Contract Research Organization |
| DNA | Deoxyribonucleic Acid |
| eCRF | Electronic Case Report Form |
| ECMO | Extracorporeal Membrane Oxygenation |
| ELISA | Enzyme-Linked Immunosorbent Assay |
| EUA | Emergency Use Authorization |
| FDA | Food and Drug Administration |
| FiO2 | Fraction of Inspired Oxygen |
| GCP | Good Clinical Practice |
| GLP | Good Laboratory Practice |
| GMFR | Geometric Mean Fold Rise |
| GMP | Good Manufacturing Practices |
| GMT | Geometric Mean Titer |
| GPE | General Physical Examination |

| | |
|---|---|
| HBV | Hepatitis B Virus |
| HCV | Hepatitis C Virus |
| HIV | Human Immunodeficiency Virus |
| ICH | International Council for Harmonisation |
| ICMRA | International Coalition of Medicines Regulatory Authorities |
| ID | Intradermal |
| IEC | Independent Ethics Committee |
| IFN g | Interferon Gamma |
| IgG | Immunoglobulin G |
| IM | Intramuscular |
| IMM | Independent Medical Monitor |
| IMP | Investigational Medicinal Product |
| IRB | Institutional Review Board |
| IRC | Internal Review Committee |
| LMIC | Low- and Middle-Income Countries |
| MedDRA | Medical Dictionary for Regulatory Activities |
| MERS | Middle East Respiratory Syndrome |
| mITT | Modified Intention-to-Treat |
| mRNA | Messenger Ribonucleic Acid |
| NIMP | Non-Investigational Medicinal Product |
| NIV | Non-Invasive Ventilation |
| NP | Nasopharyngeal |
| NSAIDs | Nonsteroidal Anti-Inflammatory Drugs |

| | |
|---|---|
| OPD | Outpatient Department |
| PCR | Polymerase Chain Reaction |
| PI | Principal Investigator |
| pO2 | Partial Pressure of Oxygen |
| PP | Per Protocol |
| RBC | Red Blood Cell |
| RBD | Receptor-Binding Domain |
| SAE | Serious Adverse Event |
| SAHPRA | South African Health Products Regulatory Authority |
| SAP | Statistical Analysis Plan |
| SARS-CoV-2 | Severe Acute Respiratory Syndrome Coronavirus 2 |
| SC | Subcutaneous |
| SpO2 | Oxygen Saturation |
| TEAE | Treatment-Emergent Adverse Events |
| UPT | Urine Pregnancy Test |
| WBC | White Blood Cell |
| WHO | World Health Organization |
| WOCBP | Women of Childbearing Potential |
| β-HCG | Beta Human Chorionic Gonadotropin |


This document is confidential.

# 2. Introduction

## 2.1 Study Rationale

The objective of this first in human Phase 1 dose-finding trial is to describe the safety, tolerability, and immunogenicity of Alveavax-v1.2 in primary vaccinated individuals. The investigated product is a Severe Acute Respiratory Syndrome Coronavirus 2 (SARS-CoV-2) Booster Vaccine candidate optimized for Omicron/BA.2. There are currently no licensed, variant-optimized booster vaccines to prevent infection with SARS-CoV-2 Omicron/BA.2. Approved or authorized booster vaccines are expensive, require a stringent cold chain, and have large-scale manufacturing issues, resulting in very limited availability in low- and middle-income countries (LMICs). Given the rapid global spread of the Omicron variant, the rapid development of an easily distributable and affordable booster vaccine is of great importance.

We will assess overall safety, tolerability and immunogenicity, including humoral and cell-mediated immunity against SARS-CoV-2 variants. In a first in human dose-finding study design we will investigate the immune responses of three dose intradermal (ID) levels in vaccinated individuals. These results will be used to inform a potential Phase 2 immunobridging trial. In addition, the feasibility and ease of ID vaccine administration will be assessed as well as the safety and immune response of subcutaneously (SC) administered ID injections. As a comparator the approved Janssen Ad26.COV2.S vaccine will be administered which has been proven to be effective against SARS-CoV-2 Omicron infections, and can be sourced for the purposes of this trial.

## 2.2 Background

### 2.2.1 Background on COVID-19 Omicron

A novel coronavirus SARS-CoV-2 was first reported in December 2019. The disease caused by SARS-CoV-2 was officially named by the World Health Organization (WHO) as coronavirus disease 2019 (COVID-19). The virus is highly transmissible between humans and has spread rapidly, causing the ongoing COVID-19 pandemic[4,5]. As of February 28, 2022, almost 430 million cases have been confirmed and close to 6 million lives claimed worldwide[48].

The SARS-CoV-2 Omicron variant (B.1.1.529 lineage) was reported by the WHO in November 2021 as a novel variant of concern (VOC) with a number of mutations and immune evasive potential[6]. This variant harbors up to 59 mutations throughout its genome[7], over 30 of which are in the spike protein: the mediator of host cell entry and main target of neutralizing antibodies[8]. The Omicron variant is highly transmissible and has quickly become dominant in many parts of the world, increasing infections and straining healthcare systems. The current


This document is confidential.

global epidemiology of SARS-CoV-2 is characterized by the dominance of the Omicron variant on a global scale[9].

The Omicron variant has a significant replication advantage, a higher secondary attack rate, and evades humoral immunity induced by either infection or currently existing vaccination to a greater extent than previous variants[10]. Although the individual risk for severe disease with Omicron is lower than with other variants, the increased number of infections still translates to high absolute numbers of hospitalizations and deaths due to COVID-19[10]. Omicron BA.1 became the dominant strain worldwide in January 2022, and now the Omicron BA.2 variant is rapidly rising as a fraction of cases in all countries for which there is strain typing capacity[11].

BA.2's growth advantage is caused by higher transmission than BA.1, and potentially increased immune escape[12]. BA.2 has a similar number of mutations to BA.1 in the spike protein, spike receptor-binding domain (RBD), and overall[13], although it only shares 32/60 of its mutations with the BA.1 strain[14]. This means that BA.2 has a greater divergence from its sister lineage, BA.1, than most other designated variants from the wild-type virus. Early data indicate that BA.2 has similar severity to BA.1[15].

### 2.2.2 Protection from Omicron in low- and middle-income countries

Vaccines are generally considered the most promising approach to mitigate the pandemic[16]. Rapid identification and sequencing of the virus allowed scientists to begin developing candidate vaccines quickly. In August 2021, the U.S. Food and Drug Administration (FDA) approved a messenger ribonucleic acid (mRNA) vaccine (Pfizer-BioNTech/Comirnaty) as a two-dose series for prevention of symptomatic COVID-19 in persons aged ≥16 years and an Emergency Use Authorization (EUA) in those aged 12-15 years. A second mRNA vaccine (Moderna), as well as a recombinant, replication-incompetent adenovirus serotype 26 (Ad26) vector vaccine (Janssen vaccine [Johnson & Johnson]), is authorized under an EUA for use in persons aged ≥18 years. Various regulatory agencies worldwide have approved these and other SARS-CoV-2 vaccines. All approved or authorized COVID-19 vaccines demonstrated efficacy (range 51% to 95%) against symptomatic, laboratory-confirmed COVID-19 in adults ≥18 years old. Available evidence suggests that the currently approved or authorized COVID-19 vaccines are highly effective against severe disease, hospitalization, and death for a variety of strains, including Alpha (B.1.1.7), Beta (B.1.351), Gamma (P.1), and Delta (B.1.617.2).

Studies of SARS-CoV-2 variants have demonstrated that mutations within the RBD mediate escape from vaccine-induced neutralizing antibodies[17,18,19]. While primary vaccination series with vaccines against the ancestral strain have shown severely reduced efficacy against Omicron, recent studies for the Janssen and mRNA boosters have demonstrated efficacy against hospitalization[20,21].

However, more than fifteen months after the EUA of the first COVID-19 vaccine, only 13.7% of people in low-income countries have had at least one vaccine dose (as of 12th March 2022)[22]. Besides cost and dose availability, worldwide distribution of mRNA vaccines is limited by the requirement for cold chain storage and shipment[23]. To our knowledge, no emergency-use listed COVID-19 vaccine yet is intended for transport at room temperature.

There is a pressing need for vaccines that protect against the BA.2 Omicron strain and can be quickly and widely distributed. While we cannot confidently predict the epidemiology or variants of SARS-CoV-2, the higher reproductive number of Omicron BA.2 coupled with breakthrough infections in those vaccinated may lead to future waves. New variants, should they arise, may be antigenically similar to the higher fitness Omicron lineage. These observations suggest that Omicron-optimized vaccination may be valuable. A cheap, easy to manufacture, and shelf-stable BA.2 vaccine could reduce the burden of the ongoing wave of Omicron SARS-CoV-2 and protect those in LMICs.

### 2.2.3 DNA vaccines

Deoxyribonucleic acid (DNA) vaccines were first developed in the early 1990s and have shown safety and immunogenicity in both preclinical and clinical trials[24,25]. DNA vaccines are being developed and tested for a diverse set of infectious diseases like human immunodeficiency virus (HIV), malaria, and tuberculosis, and they have demonstrated promising potential in cancer immunotherapy[26]. While the immunogens themselves are not infectious, they can induce both cellular and humoral immune responses[27,28].

DNA vaccines have been used in vaccine development for SARS-CoV-2-related viruses, such as Middle East Respiratory Syndrome (MERS) virus and SARS-CoV-1[27,29]. The DNA vaccines were found to be well tolerated in humans and induced an antibody response in >80% of the participants. Using a similar approach, a SARS-CoV-2 vaccine candidate (ZyCoV-D) has been developed by Zydus Cadila[30]. The vaccine was recently approved for emergency use in India, based on a 28,000-person trial.

As a platform technology, DNA vaccines have great potential to address priority pathogens during public health emergencies[31,32]. Like mRNA vaccines, DNA vaccines can be designed quickly. The gene insert of a DNA vaccine can be rapidly adjusted in response to emerging global health threats, while the manufacturing and control of the new product may remain the same[28]. The greatest benefit of DNA vaccines, especially in resource limited settings, is that these are more stable than mRNA vaccines and can even be stored at ambient temperatures[28].


This document is confidential.

To address vaccine delivery challenges and ensure equitable and rapid access to much needed COVID-19 booster vaccines, Alvea LLC has developed a plasmid DNA booster vaccine, Alveavax-v1.2. The vaccine comprises double-stranded plasmid DNA carrying the gene for the SARS-Cov-2 spike protein containing Omicron/BA.2-specific mutations, as well as two proline mutations to stabilize the prefusion conformation of the protein in a pVAX1 backbone.

## 2.3 Risk/Benefit Assessment

The Omicron variant demonstrates immune escape and increased transmissibility in previously vaccinated or infected individuals. There are no licensed human SARS-CoV-2 vaccines available optimized for the Omicron variant currently.

No safety or immunogenicity information in humans is currently available for the Omicron-optimized Alveavax-v1.2 vaccine. Non-clinical safety of Alveavax-v1.2 was confirmed in Good Laboratory Practice (GLP) studies in mice. Clinical studies with other approved/experimental vaccines have not identified any undesirable side effects with the use of DNA vaccines developed for SARS-CoV. Common events associated with vaccines such as injection site pain, tenderness or pruritus and general side effects such as pyrexia, arthralgia, and diarrhea following vaccination were seen in <10% of participants[33].

All mild to severe adverse events (SAEs) will be monitored during this study. Measures will be in place to monitor participants. Safety will be ensured through the review and appraisal of clinical data, laboratory profile and risk management documentation. Adverse events (AEs) after vaccination are expected to be mild and manageable using routine symptom-driven standard of care as determined by the investigators.

### 2.3.1 Risk Assessment

| Potential Risk of Clinical Significance | Summary of Data/Rationale for Risk | Mitigation Strategy |
|---|---|---|
| Potential for local reactions (injection site redness, injection site swelling, and injection site pain) and systemic events (fever, fatigue, headache, chills, vomiting, diarrhea, muscle pain, and joint pain) following vaccination. | These are common adverse reactions seen with other vaccines, as noted in the FDA Center for Biologics Evaluation and Research (CBER) guidelines on toxicity grading scales for healthy adult volunteers enrolled in preventive vaccine clinical | The study design includes the use of controlled vaccination to closely monitor and ensure participant safety. A reactogenicity diary will be used to monitor local reactions and systemic events. Stopping rules are in |


This document is confidential.

| | | |
|---|---|---|
| | trials. | place. The first 5 participants treated who received a low, standard and high dose will be observed for 4 hours after vaccination and all other participants will be observed for 30 minutes after vaccination to assess any immediate AEs. |
| Unknown AEs and laboratory abnormalities with a novel vaccine. | Studying vaccine candidates in humans can uncover previously unknown or unexpected AEs. The safety of plasmid DNA delivery has been extensively validated and the WHO recognizes that "To date, published data from clinical trials indicate that DNA vaccines are safe and have acceptable reactogenicity profiles."[41] | The study design includes the use of controlled vaccination to closely monitor and ensure participant safety. An independent medical Monitor (IMM) will also review safety data. Stopping rules are in place. The first 5 participants treated who received a low, standard and high dose will be observed for 4 hours after vaccination and all other participants will be observed for 30 minutes after vaccination to assess any immediate AEs. |
| Participants will be required to attend healthcare facilities during the global SARS-CoV-2 pandemic. | Without appropriate social distancing and PPE, there is a potential for increased exposure to SARS-CoV-2. | The Sponsor will work with sites to ensure an appropriate COVID-19 prevention strategy. |
| Venipuncture, ID, SC and IM injections will be performed during the study. | There is the risk of bleeding, bruising, hematoma formation, and infection at the injection site, and pain associated with the injection(s). | Only appropriately qualified personnel will obtain the blood draw, and perform ID, SC and IM injection. |

### 2.3.2 Benefit Assessment

Benefits to individual participants may include:

- Receipt of a potentially efficacious Omicron-optimized COVID-19 booster vaccine during a global pandemic and Omicron wave
- Access to extensive diagnostic testing and medical check-ups
- Early access to vaccine if efficacious and approved
- Contributing to research to help others during a global pandemic

### 2.3.3 Overall Benefit/Risk Conclusion

Based on available nonclinical data of Alveavax-v1.2, data from human studies with related DNA vaccines, and taking into account the measures taken to minimize risk to participants participating in this study, the potential risks identified in association with the Alveavax-v1.2 SARS-CoV-2 vaccine are justified by the anticipated benefits that may be afforded to healthy participants.

# 3. Objectives and Endpoints

The objective of the study is to assess the tolerability, safety, and immunogenicity of different doses and routes of administration of the Alveavax-v1.2 vaccine in healthy individuals. Exploratory endpoints will or will not be tested based on findings from secondary endpoints and sponsor's determination accordingly.

| *Objectives* | *Endpoints* | *Timepoints* |
|---|---|---|
| **Primary** | | |
| To evaluate the safety and tolerability of Alveavax-v1.2 from Alvea, LLC, in healthy participants, compared to a control booster vaccine. | Safety measured using the following:<br>1. Number of participants with solicited local and systemic AEs within 7 days of dose administration<br>2. Number of participants with unsolicited AEs within 28 days of vaccination<br>3. Number of participants with any SAEs, adverse events of | Day 1, 7, 28, after 3, and 6 months |


This document is confidential.

| | | |
|---|---|---|
| | special interest (AESIs), and AEs leading to withdrawal during the entire period of study | |
| **Secondary** | | |
| To evaluate the immunogenicity as humoral immune response against SARS-CoV-2 BA.2/Omicron after a booster dose of Alveavax-v1.2 | **Humoral immune response**<br>Characterization of humoral immune response of booster vaccinations against SARS-CoV-2 BA.2/Omicron measured using the following:<br>1. Change in GMT of anti-spike protein (S) immunoglobulin G (IgG) antibody<br>2. GMT of anti-spike protein (S) IgG antibody<br>3. GMFR of anti-spike protein (S) IgG | Baseline (pre-vaccination; except for Point 1), Day 28 |
| To evaluate the clinical efficacy against SARS-CoV-2 after a booster dose of Alveavax-v1.2 | **Clinical efficacy**<br>Clinical efficacy measured using the WHO clinical progression scale for COVID-19. | Day 7, Day 14, Day 28, Day 84, Day 168 |
| To evaluate success rate of ID injections | **Bleb success rate**<br>Absolute number and fraction of ID injections which generated a ≥ 1 mm and ≥ 7 mm in diameter clearly demarcated bleb, clearly visible for at least 20 seconds, for 0.5 mg and 2 mg Alveavax-v1.2 respectively | Dosing |
| **Exploratory** | | |
| To evaluate the cell-mediated immune response against SARS-CoV-2 BA.2/Omicron after a booster dose of Alveavax-v1.2 | **Cellular immune response**<br>Characterization of cellular immune response of booster vaccinations against SARS-CoV-2 ancestral and BA.2/Omicron measured using the following:<br>1. Spike-specific CD4+ T-cell response<br>2. Change in spike-specific CD4+ T-cell response | Baseline (pre-vaccination), Day 28 |


This document is confidential.

| | | |
|---|---|---|
| | 3. Spike-specific CD8+ T-cell response<br>4. Change in spike-specific CD8+ T-cell response | |
| To evaluate fc effector functions against SARS-CoV-2 after a booster dose of Alveavax-v1.2 | Activity against Omicron/BA.2, Ancestral variants (such as Alpha, Beta, Delta), and additional Omicron Subvariants (such as BA.1) as<br>1. Change in relative light units (RLU), and<br>2. RLU of<br><br>A. Antibody dependent cellular cytotoxicity (ADCC)<br>B. Antibody dependent cellular phagocytosis (ADCP)<br>C. Antibody dependent cellular trogocytosis<br>D. Complement deposition<br>E. Fc dimer receptor binding | Baseline (pre-vaccination; except for Point 1), Day 7, Day 14, Day 28, Day 84, Day 168 |
| To evaluate the humoral immune response against additional SARS-CoV-2 variants and sub-lineages after a booster dose of Alveavax-v1.2 | Characterization of humoral immune response of booster vaccinations against Ancestral, existing and yet to be defined variants (such as Alpha, Beta, Delta), and existing and yet to be defined sub-lineage (such as Omicron BA.1) SARS-CoV-2 strains measured using by:<br>1. Change in GMT,<br>2. GMT, and<br>3. GMFR of<br><br>A. serum anti-SARS-CoV-2 neutralizing antibodies<br>B. serum anti-S IgG antibodies<br>C. serum anti-S-RBD IgG antibodies | Baseline (pre-vaccination; except for Point 1), Day 7, Day 14, Day 28, Day 84, Day 168 |
| To correlate clinical efficacy with neutralizing antibody response | For each neutralizing antibody type measured:<br>1. Percentage of participants with neutralization IC50>64 IU/mL against the respective strain | Day 7, Day 14, Day 28, Day 84, Day 168 |


This document is confidential.

| | | |
|---|---|---|
| | 2. Percentage of participants with neutralization IC50>128 IU/mL against the respective strain | |
| To correlate clinical efficacy and immunogenicity with anti-nucleocapsid protein antibodies | Serologic change in GMT and GMFR between baseline and other blood samples for anti-nucleocapsid protein (N) IgG antibodies | Baseline, Day 7, Day 14, Day 28, Day 84, Day 168 |

# 4. Study Design

## 4.1 Overall Design

This is a first in human, open-label, active-controlled, randomized dose-finding study to evaluate safety, tolerability, and immunogenicity of ID and SC application of the plasmid DNA SARS-CoV-2 Omicron BA.2 vaccine Alveavax-v1.2 in primary Ad26.COV2.S vaccinated healthy individuals.

Primary Ad26.COV2.S vaccinated participants will be randomized into one of 5 treatment arms to receive Alveavax-v1.2 or a Ad26.COV2.S control booster vaccine.
Participants will be enrolled at seven (+/- 3) sites in South Africa within 28 days after the initial screening to ensure they meet all the inclusion criteria and none of the exclusion criteria.

Each participant will be administered a booster vaccine on Day 1 of the study and will be monitored afterwards as described below and in 8.3. Solicited local/systemic reactions will be recorded after vaccination in the participant's diary card for up to 7 days (the vaccine administration day and 6 days later) as described in 8.1.6. Adverse events (AE) as defined in 8.5, and concomitant medications as defined in 6.7 will be collected throughout the study.
A total of 130 male and female participants aged between 18 and 65 years who satisfy the inclusion and exclusion criteria are planned to be enrolled in five groups and with vaccine administered according to Table 3:

1. Individuals with a primary vaccination will get either:
    a. **Low dose**: 0.5 mg Alveavax-v1.2 in one ID injection
    b. **Standard dose**: 2 mg Alveavax-v1.2 in one ID injection
    c. **High dose**: 8mg Alveavax-v1.2 in four ID injections*
    d. **SC injection**: 8mg Alveavax-v1.2 in one SC injection
    e. **Control**: Janssen Ad26.COV2.S in one IM injection

* Four ID injections shall be administered immediately one after the other, preferably at the same anatomic site (e.g. upper arm) with a few centimeters distance between each injection.

Dependent upon safety and/or immunogenicity data generated during the course of this study it is possible that groups may be started at the next highest dose, groups may not be started, groups may be terminated early, and/or groups may be added with dose levels below the lowest stated dose or intermediate between the lowest and highest stated doses.

The enrollment will start with the first ten participants randomized into the low dose (1.a) arm, the control arm (1.e), subsequently at least 24 hours apart five participants to the vaccinated standard dose (1.b) arm, followed by a review of 24h safety data by the independent medical monitor. Then the remaining participants assigned to the low and standard dose cohorts, in addition to those assigned to the control arm (1.e) will be recruited. In parallel, the first five participants of the high dose (1.c) arm will be enrolled. In parallel, the first five participants of the high dose (1.c) arm are enrolled. Further recruitment and the SC injection arm (1.d) start as soon as the independent medical monitor has reviewed 24h safety data of the high dose arm (see schema in 1.2).

For each dose level the following apply:

- Additional safety assessments (see 8.3)
- Controlled enrollment (**for new dose levels** low / standard and high dose):
    - No more than 5 participants to be vaccinated on the first day
    - The first 5 participants must be observed for at least 4 hours after vaccination for any acute reactions
    - Vaccination of the remaining participants (participant 6 and above) will commence no sooner than 24 hours after the fifth participant received his or her vaccination
- Application of stopping rules (see 4.4.2)
- Escalation between dose levels will be allowed only after independent medical monitor review of at least 24 hours post–dose safety data in this study

## 4.2 Scientific Rationale for Study Design

The Alveavax-v1.2 vaccine is being developed to prevent severe cases of COVID-19, the disease resulting from SARS-CoV-2 infection. The study is designed to primarily evaluate the tolerability and safety of Alveavax-v1.2 for up to 6 months after the booster dose with Alveavax-v1.2. Additionally the study will determine immunogenicity endpoints and descriptive clinical efficacy against SARS-CoV-2 infection as secondary objectives.

Human reproductive safety data are not available for Alveavax-v1.2, but there is no suspicion of human teratogenicity based on the intended mechanism of action of the compound. Nevertheless, the use of a highly effective method of contraception is required (see Appendix 1) and pregnant and breastfeeding women will be excluded from the trial.

## 4.3 Justification for Dose and Route of Administration

The current study will be the first time Alveavax-v1.2 is administered to humans. Safety and tolerability of chosen doses of Alveavax-v1.2 are supported by preclinical studies.

Previous phase I, II and III clinical trials have shown DNA vaccines for SARS-CoV-2 to be safe and well-tolerated in humans in a variety of doses and routes of administration [5][45][46].

The selected standard dose of 2 mg of Alveavax-v1.2 is based on trial results of the first emergency authorized DNA vaccine against COVID-19, ZyCoV-D. ZyCoV-D is the closest extensively studied product to Alveavax-v1.2: it utilizes the same plasmid backbone - pVAX1 - and a similar antigen - SARS-CoV-2 spike protein - and is also administered intradermally. Preclinical studies with ZyCoV-D showed that a 2 mg dose is generally safe and well-tolerated[34]. The Phase I study showed that a dose of 2 mg, injected either by needle or with Pharmajet Tropis needle-free device, resulted in significantly higher immunogenicity than a dose of 1 mg in unvaccinated and SARS-CoV-2 naïve healthy individuals. No deaths or serious adverse reactions were reported in this study of 126 participants[5]. Subsequently, ZyCoV-D ID primary 3 dose regimen at 2mg / dose received emergency use authorization in India on the basis of interim Phase III study data[44]. Supporting this choice, ID administration of a 2mg dose of DNA (followed by electroporation) was also safe and well tolerated in a Phase I trial for a different vaccine against SARS-CoV-2[49], as well as in previous human Phase I trials for Ebola[50] and Zika[51] vaccines.

As these studies were conducted in SARS-CoV-2 naïve individuals we will additionally test a 4x lower dose of 0.5 mg to assess whether a lower dose may cause a sufficient immune response in pre-immunized subjects.

DNA vaccines have been administered at higher doses with acceptable safety and tolerability. Human studies for the GX-19 pDNA COVID-19 vaccine showed a 3 mg dose to be safe and well tolerated[45]. AnGes DNA vaccine studies against COVID-19 suggest higher doses might be required to elicit a sufficient antibody response[46] and are now enrolling a clinical trial testing single doses of up to 8 mg and total doses up to 16 mg[47]. We therefore selected 8 mg as our maximum dose. As ID injections can erroneously be administered subcutaneously, safety and immunogenicity data for both ID and SC injections with 8 mg will be collected. A dose equivalent to at least 25 times the maximum dose in humans has been shown to be safe in preclinical studies in mice.


This document is confidential.

## 4.4 End of Study Definition

### 4.4.1 Termination of the Clinical Study

The Sponsor, the Investigator (following consultation with the Sponsor), SAHPRA (South African Health Products Regulatory Authority), or IRB/IEC (Institutional Review Board / Independent Ethics Committee) have the right to discontinue this study at any time after appropriate consultation among the involved parties. The Sponsor has the right to suspend or discontinue their investigational product development at any time.

Conditions that may warrant termination of the study include but are not limited to the following:

- The discovery of unexpected and relevant conditions or events that suggest a possible hazard or unacceptable risk to the participants enrolled in the clinical study.
- Early definitive results/data proving no effect

If the study is terminated and/or the site closed for any reason previously listed or for other factors, copies of study and study product documentation must be returned to the Sponsor. The study site will keep an archive of site-specific documents.

### 4.4.2 Study Stopping Criteria

Throughout the study period, if any SAE or serious complaint occurs, Investigators are obligated to review the participant's data immediately and make a medical judgment of whether the SAE or serious complaint is vaccine related or not. If any of the following situations occur, PIs must inform the Sponsor / Contract Research Organization (CRO), and study recruitment may be suspended until further notice.

The AE review is as per USFDA guidance, Toxicity Grading Scale for Healthy Adult and Adolescent Volunteers Enrolled in Preventive Vaccine Clinical Trials.

In the event of such an occurrence, the Sponsor reserves the right to halt and review this study and will discuss it with the Investigators (including the reasons for taking such action).

- Vaccination will be placed on hold if (see 8.5.4 for definitions)
    - Two or more participants report the same or similar severe (Grade 4) AE after vaccination **possibly related** to the study investigational product by the Investigator, or

    - Any participant vaccinated develops a severe (Grade 4) AE event after vaccination **probably or definitely related** to the study investigational product by the Investigator.
- Opinions of IEC and IMM will be collected before enrolling further participants.

# 5. Study Population

Healthy adult individuals, previously having received a primary Ad26.COV2.S vaccination series against SARS-CoV-2, satisfying all the eligibility criteria will be eligible to participate in the study. Screening for eligible participants will be performed within 28 days of vaccination in the study.

The inclusion and exclusion criteria for enrolling participants in this study are described below. If there is a question about the inclusion or exclusion criteria, the Investigator must consult with the appropriate Sponsor representative and resolve any issues before enrolling a participant in the study. Waivers are not allowed, unless otherwise specified in the inclusion / exclusion criteria.

## 5.1 Number of Participants Planned

A total of 130 healthy participants will be enrolled in five groups.

## 5.2 Inclusion Criteria

Each potential participant must satisfy all of the following criteria to be enrolled in the study:

1. Healthy adult male and female volunteers between 18 and 65 years of age, inclusive.
2. Participants who received a primary Janssen Ad26.COV2.S vaccine ≥ 60 days prior to receiving the study vaccine (Day 1) in this study.
3. Body mass index (BMI) within the range 18 - 32 $kg/m^2$ (both inclusive).
4. Participants who, judged by the Investigator, are in stable health as determined by their pre-study medical history, physical examination, and clinical laboratory tests.
5. Female participants must be either of non-childbearing potential, i.e., surgically sterilized (defined as having undergone hysterectomy and/or bilateral oophorectomy and/or bilateral salpingectomy; tubal ligation alone is not considered sufficient) or one year postmenopausal; or, if of childbearing potential, they must be abstinent or have used adequate contraceptive precautions (see Appendix 1: Contraception) for 30 days prior to receiving the study vaccination and 84 days post-vaccine.
6. Sexually active male participants who are considered sexually fertile must agree to use a barrier method of contraception during sexual activity with a female of childbearing potential from the time of vaccination until at least 84 days after the vaccination.

7. Participants must provide written informed consent or their legal representative must understand and give written consent to the procedure.
8. Participants must be willing and able to comply with all the required study visits and follow-up required by this protocol, and be able to complete the diary card after vaccination or have a caregiver available to assist with these matters.

## 5.3 Exclusion Criteria

Any potential participant who meets any of the following criteria will be excluded from participating in the study:

1. Received any other SARS-CoV-2 vaccination than a single Janssen Ad26.COV2.S vaccine or plans to receive any additional SARS-CoV-2 vaccination within 90 days after the study vaccine (Day 1).
2. Recovered from SARS-CoV-2 infection determined by history of a positive SARS-CoV-2 test (e.g. PCR, rapid antigen test, etc.) or suspicion of a SARS-CoV-2 infection based on the (verbal) medical history within less than 60 days from the day of vaccination (Day 1) in this study.
3. History of close contact (face-to-face contact within 1 meter or contact in a closed space for more than 15 minutes) without wearing a face-mask with a confirmed active SARS-CoV-2-positive patient within 5 days prior to Day 1.
4. Have received any live-virus vaccine within 4 weeks or inactivated vaccine, including influenza vaccine, within 2 weeks (both licensed and investigational vaccines) prior to the study vaccine (Day 1).
5. Previous participation in any clinical trial of a SARS-CoV-2 vaccine candidate.
6. Have any febrile illness (temperature ≥ 38°C/100.4°F) or any active acute illness or infection (including a positive SARS-CoV-2 PCR test) within 7 days prior to administration of vaccination (Day 1) in this study. Participants may be re-evaluated once all symptoms have resolved.
7. History of severe adverse reaction associated with a vaccine and/or severe allergic reaction (eg, anaphylaxis) or contraindications to any component of the study intervention(s).
8. History of, or positive screening test for human immunodeficiency virus I or II.
9. Any clinically significant finding during screening or check-in that, in the Investigator's judgment, results in an increased safety risk.
10. History of cerebral venous sinus thrombosis, antiphospholipid syndrome, or a history of heparin-induced thrombocytopenia and thrombosis (HITT or HIT type 2).
11. Any confirmed or suspected immunosuppressive or immunodeficient state; asplenia; recurrent severe infections and use of immunosuppressant medication within the past 3 months, except topical and inhaled steroids, or short-term oral steroids (course lasting ≤14 days or ≤20 mg/day).


This document is confidential.

12. History of receiving blood transfusion, blood products, immunoglobulin, or immune stimulants within 3 months prior to Day 1.
13. Is currently participating in any other study or has received any investigational drug in the last 6 weeks or 5× the half-life of the drug (whichever is longer) prior to screening.
14. For female participants of childbearing potential who are pregnant (positive pregnancy test at the screening or check-in), currently breastfeeding, or attempting to conceive.
15. Any addiction that may interfere with the participant's ability to comply with trial procedures.
16. Inability to be venipunctured or tolerate venous, IM, SC, or ID puncture.
17. Have a rash, dermatological condition, tattoo, or any other abnormality at the injection site that may interfere with injection site reaction rating. Investigator discretion will be permitted with this exclusion criterion.
18. Use of prophylactic medications (e.g., antihistamines [H1 receptor antagonists], nonsteroidal anti-inflammatory drugs [NSAIDs], systemic glucocorticoids, non-opioid and opioid analgesics) within 24 hours prior to the vaccination to prevent or pre-empt symptoms due to vaccination.
19. Any condition or abnormal baseline findings or any other unspecified reason, which in the Investigator's judgment might increase the risk to the participant or decrease the chance of obtaining satisfactory data needed to achieve the objective of the study.
20. Participants identified as an Investigator or employee of the Investigator or clinical site with direct involvement in the proposed study, or identified as an immediate family member (i.e., parent, spouse, natural or adopted child) of the Investigator, or employee with direct involvement in the proposed study, or any employees of the Sponsor company.

## 5.4 Lifestyle Considerations

Potential participants must be willing and able to adhere to the following lifestyle restrictions during the course of the study to be eligible for participation:

1. Refer to Section 6.7, Concomitant and Prohibited Therapies, for details regarding prohibited and restricted therapy during the study.
2. Agree to follow all requirements that must be met during the study as noted in the Inclusion and Exclusion Criteria.

## 5.5 Screen Failure

A participant is considered to be a screen failure if the participant signs the informed consent form but is ineligible at the screening visit or withdraws before receiving trial medication. The Investigators must account for all participants who sign an informed consent form for the study. All potential participants who are screened for enrollment in this study, including screening failures, will be listed on the participant screening list. The primary reason for screen failure will

be recorded in the electronic Case Report Form (eCRF). Participant identification numbers assigned to participants who fail screening will not be reused.

The Investigators are responsible for all participants who sign an informed consent form for the study. If a participant is found not to be eligible to participate in the study at the screening visit, appropriate eCRFs must be completed by the Investigators. The eCRF will include the primary reason for the screen failure. Identification numbers assigned to ineligible participants will not be reused.

# 6. Study Treatments / Investigational Product Management

## 6.1 Study Intervention

The study will evaluate a single-dose schedule of multiple dose levels and application variations of an investigational plasmid DNA booster vaccine candidate (Alveavax-v1.2) compared with a COVID-19 booster vaccine as control for active immunization optimized for COVID-19 Omicron.

The investigational plasmid DNA booster vaccine candidate and the control vaccine are the potential study interventions that may be administered to a study participant:

- Alveavax-v1.2: 0.5 mg, 2 mg or 8 mg
- Janssen Ad26.COV2.S COVID-19 vaccine: 0.5 mL

### 6.1.1 Investigational Product

Alveavax-v1.2 is a preservative-free, sterile formulation of plasmid DNA in an isotonic Phosphate Buffer Saline. The plasmid DNA substance is the only active substance in this product. The product is a concentrate for injection and filled at 5 ± 0.5 mg/ml. Isotonic Phosphate Buffer Saline solution is sourced as an approved medicinal product.

The composition of the investigational product and the functions of the respective components are given in Appendix 4. More detailed information about the study drug can be found in the Investigator's Brochure (IB) Alveavax-v1.2.


This document is confidential.

### 6.1.2 Control Vaccine

The active control vaccine for the study, Janssen Ad26.COV2.S, is a COVID-19 booster vaccine authorized for emergency use / approved. It is available as a single booster dose for individuals who have completed the primary series of vaccinations. This booster vaccine has been demonstrated to be effective against Omicron and is available for use in this study.

A single booster dose of the control vaccine will be administered via IM injection in dosages as per the prescribing information.

### 6.1.3 Study intervention(s) administered

| **Intervention Name** | **Alveavax-v1.2** | **Ad26.COV2.S** |
|---|---|---|
| **Type** | Vaccine | Vaccine |
| **Vaccine Type** | Plasmid DNA | Adenovirus vector |
| **Unit Dose Strength** | 5 mg / 1 mL | 8.92 $\log_{10}$ infectious units / 0.5 mL |
| **Dose Level** | 0.5 mg, 2 mg or 8 mg | 8.92 $\log_{10}$ infectious units |
| **Route(s) of Administration** | ID and SC injection | IM injection |
| **Use** | Experimental | Control |
| **IMP or NIMP** | IMP | NIMP |
| **Sourcing** | Provided centrally by the Sponsor | Provided centrally by the Sponsor |
| **Packaging and Labeling** | Study intervention will be provided in a glass vial as open-label supply. Each vial will be labeled as required per country requirement | 2.5 mL suspension in a multi-dose vial (type I glass) with a rubber stopper, aluminum crimp and blue plastic cap. |

## 6.2 Dosage and Treatment Schedule

The participants will receive a total of up to four injections, either one to four ID, one SC or one IM via needle as per Table 3 at ambient temperature.

The ID and SC injections will be given to the outer surface of the upper arm (or front of the thigh, or lower back). The IM injections will be handled and administered following the protocol for the control booster vaccine.

Administration of study interventions should be performed by an appropriately qualified, GCP-trained, and vaccine-experienced member of the study staff (eg, physician, nurse, physician's assistant, nurse practitioner, pharmacist, or medical assistant) as allowed by local, state, and institutional guidance.

Table 3: Dosing Schema

| Arms | No. of participants * | Dose | Delivery method | Route | Dose administration |
|---|---|---|---|---|---|
| **Group 1: Primary vaccinated individuals** | | | | | |
| **1.A: Low dose** | 20 | 0.5 mg of Alveavax-v1.2 | Needle | ID | Single injection |
| **1.B: Standard dose** | 40 | 2 mg of Alveavax-v1.2 | Needle | ID | Single injection |
| **1.C: High dose** | 20 | 8 mg of Alveavax-v1.2 | Needle | ID | Four injections (4x 2 mg) |
| **1.D: SC injection** | 10 | 8 mg of Alveavax-v1.2 | Needle | SC | Single injection |
| **1.E: Control** | 40 | Control booster vaccine | Needle | IM | Single injection |

* Sample size determination in Section 9.2

## 6.3 Supply and Administration of Investigational Product

The investigational product will be provided as a solution for injection. No further dilution is required. Each vial contains two standard doses. Detailed instructions for storage and handling are provided in the respective trial-specific Pharmacy Manuals.


This document is confidential.

## 6.4 Storage and Preparation

### 6.4.1 Storage

All study vaccine must be stored in a secured location with no access for unauthorized personnel and at controlled temperatures as indicated on the clinical labels. If study vaccine is exposed to temperatures outside the specified temperature range, all relevant data will be sent to the Sponsor to determine if the affected supplies can be used or will be replaced. The affected study vaccine must be quarantined and not used until further instruction from the Sponsor is received.

The vaccines must not be frozen, and must be stored separately from the normal hospital/ practice stocks, accessible only to authorized personnel.

Refer to the pharmacy manual for further information.

### 6.4.2 Preparation

See the pharmacy manual for instructions on how to prepare the study intervention for administration and the prescribing information on how to prepare the control vaccine.
A pharmacist or other qualified individual (eg, physician, nurse, physician's assistant, nurse practitioner, pharmacy assistant/technician, or pharmacist) as allowed by local, state, and institutional guidance will prepare the appropriate vial and syringe, labeled with the participant's identification number, and provide the syringe to unblinded administrator who will perform the injection.

### 6.4.3 Accountability

The Investigator is responsible for ensuring that all study vaccine vials received at the site are inventoried and accounted for throughout the study. The study vaccine administered to the participant must be documented on the vaccine accountability form. All study vaccine vials will be stored and disposed of according to the Sponsor's instructions. Study-site personnel must not combine contents of the study vaccine containers.

Study vaccine must be handled in strict accordance with the protocol and the container label, and must be stored at the study site in a limited-access area or in a locked cabinet under appropriate environmental conditions. Unused study vaccine vials must be available for verification by the Sponsor's study site monitor during on-site monitoring visits. The return to the Sponsor of unused study vaccine vials will be documented on the vaccine accountability form. When the


This document is confidential.

study site is an authorized destruction unit and study vaccine supplies are destroyed on-site, this must also be documented on the vaccine accountability form.

Potentially hazardous materials containing hazardous liquids, should be disposed of immediately in a safe manner and therefore will not be retained for vaccine accountability purposes.
Study vaccine vials should be dispensed under the supervision of the Investigator or a qualified member of the study-site personnel, or by a hospital/clinic pharmacist. Study vaccine will be administered only to participants participating in the study. Returned study vaccine vials must not be dispensed again, even to the same participant. Study vaccine vials may not be relabeled or reassigned for use by other participants. The Investigator agrees neither to dispense the study vaccine from, nor store it at, any site other than the study sites agreed upon with the Sponsor.

## 6.5 Measures to Minimize Bias: Randomization and Blinding

### 6.5.1 Procedures for Randomization and Stratification

Following the enrollment and monitoring of the first 5 participants in each of the 1.a low dose arm, 1.e control arm and 1.b standard dose arm, the remaining participants in these arms will be randomly assigned into one of these arms.

Following the enrollment and monitoring of the first 5 participants in the 1.c high dose arm, the remaining participants in the high dose and SC injection arm will be randomly assigned into one of these arms.

Randomization will be performed centrally. Further detail will be provided in a separate randomization plan.

### 6.5.2 Blinding

The trial will be conducted open-label.

## 6.6 Study Vaccination Compliance

Study vaccines will be administered by unblinded qualified study site personnel at the study site according to the Pharmacy Manual. Appropriate personnel must be adequately trained to ensure proper ID administration of the product, prior to injection into participants.

Every injection will be checked by the study person who has administered the vaccine immediately after administration to confirm that the syringe is completely empty. Additionally, for ID injections, to confirm that a clearly demarcated bleb ≥ 1 mm and ≥ 7 mm in diameter is generated and is clearly visible for at least 20 seconds, for 0.5 mg and 2 mg Alveavax-v1.2


This document is confidential.

respectively. The anatomical site of the bleb, the bleb size and if the bleb remained for at least 20 seconds will be documented in the electronic Case Report Form eCRF (including date and time of injection and location used for IM, SC, or ID injection).

## 6.7 Concomitant and Prohibited Therapies

### 6.7.1 Concomitant Medication

Concomitant medication given to, or taken by, the participant during the study must be clearly documented on the eCRF, as follows:

- From one year to 30 days before the vaccination all immunoglobulin, immune stimulants, and vaccines received.
- From 30 days before the vaccination (Day 1) to study Day 28 (Visit 5) - all prescription, non-prescription, or over-the-counter medications (such as aspirin or antacids), vitamins, mineral supplements, and herbal remedies; all medication used to treat conditions reported as medical history; and medication used to treat AEs (solicited AEs, unsolicited AEs, AESI, SAE, or AE leading to withdrawal).
- From Day 29 to the end of the study, inclusive: any concomitant medication(s) administered to treat AEs: on-going medications for AE that occurred before Day 28; and medication for AESI, SAE, or AE leading to withdrawal.

Information about the medication's generic name / trade name (or names, if combination medication), indication, total daily dose, route of administration, and start and end dates of treatment should be entered in eCRF.

A note will be made of whether the medication being taken is prophylactic. A prophylactic medication is a medication administered in the absence of any symptom and in anticipation of a reaction to treatment. Concomitant medication administered for the treatment of an AE or SAE must be recorded on the Concomitant Medication Log of the CRF and SAE Report.

Medications considered necessary for the participants, and which are not known to interfere with the study vaccine, may be allowed at the discretion of the Investigator, and appropriate records of the same will be kept in the CRF. Furthermore, the concomitant use of medications for the treatment of other concomitant diseases not known to interact with the study vaccine would be permitted as deemed necessary by the Investigator. A list of all the concomitant medications consumed by the participant would be recorded in the CRF.

### 6.7.2 Prohibited Therapies

Use of the following medications and therapies within the specified time is not permitted:


This document is confidential.

1. SARS-CoV-2 vaccination 90 days after Day 1.
2. Any live-virus vaccine (both licensed and investigational) within 4 weeks prior to and 4 weeks after Day 1.
3. Any inactivated vaccine (both licensed and investigational), including influenza vaccine, within 2 weeks prior to and 2 weeks after Day 1.
4. Any blood products, including immunoglobulin, or immune stimulants within 3 months prior to Day 1.
5. Immunosuppressant or other immune modifying drugs within 3 months prior to Day 1.
6. Use of prophylactic medications (e.g., antihistamines [H1 receptor antagonists], antipyretics, nonsteroidal anti-inflammatory drugs [NSAIDs], systemic and topical glucocorticoids, non-opioid and opioid analgesics) within 24 hours prior to the vaccination to prevent or pre-empt symptoms due to vaccination.

Depending on the time of the occurrence, any participant who receives a prohibited concomitant medication will not be included in the immunogenicity analyses.

### 6.8 Possible Vaccine Interactions

The following medications can lead to drug interactions with Alveavax-v1.2 vaccine and might influence immunogenicity results:

1. Immunoglobulins, blood, or blood products:
    a. These can affect study evaluations related to immunogenicity data for the vaccine.
2. Immunostimulant agents:
    a. These could change the immunological response of the vaccine.
3. Immunosuppressive agents:
    a. These may result in the participant not developing optimum immunologic responses to the vaccine.
4. Antiviral medications:
    a. These could change the immunological response of the vaccine.
5. Anti-coagulants:
    a. These could put the participant at increased risk of formation of injection site hematoma.

# 7. Discontinuation of Study Vaccination and Participation Discontinuation/Withdrawal

### 7.1 Discontinuation of Study Intervention

Discontinuation from the study is defined as non-completion of the study-defined visits and examinations. Participants may voluntarily withdraw from the study for any reason at any time.


This document is confidential.

A participant may be discontinued from the study if there is a serious or intolerable AE, or if it would not be in the participant's best interest to continue further in the study according to the Investigator.

## 7.2 Participant Discontinuation/Withdrawal From the Study

The Investigator may withdraw a participant from the study if they meet any of the following withdrawal criteria:

- The participant wishes to withdraw their consent for participation
- Severe (≥ Grade 3) AE or laboratory abnormality related to vaccines
- The participant suffers from significant intercurrent illness or undergoes major surgical intervention during the study
- Lost to follow-up
- Administration of any vaccine or prohibited therapies within 28 days of administration of the study vaccine
- Participant in non-compliance to protocol (including violation of enrollment criteria)
- When it is not in the participant's best interest to continue, in the Investigator's opinion

The date and the reason the participant is withdrawn from the study will be documented in the eCRF. Participants who are withdrawn because of AEs must be clearly distinguished from participants who are withdrawn for other reasons. Withdrawn participants who have been administered the study vaccine will be considered for safety analysis.

If randomized participants are withdrawn from vaccination before the study vaccine is administered, additional participants may be recruited to replace these participants at the discretion of the Sponsor. Any replacement participant will be assigned to the same group as the original (discontinued) participant. If randomized participants are withdrawn after the study vaccine is administered, they will not be replaced.

## 7.3 Lost to Follow-Up

A participant will be considered lost to follow-up if he or she repeatedly fails to return for scheduled visits and is unable to be contacted by the study site. A participant cannot be deemed lost to follow-up until all reasonable efforts made by the study-site personnel to contact the participant are deemed futile. The following actions must be taken if a participant fails to return to the study site for a required study visit:

Every attempt will be made to contact study participants who are lost to follow-up. Sites should attempt to collect and save a secondary contact number and email for each participant. At least four attempts at contact will be made and recorded in the source documents on three distinct days by phone, text message and email [if available]. As a last resort, any participant with whom the


This document is confidential.

clinic staff no longer has contact will be notified of a request to be contacted either by registered letter or, failing that, by such other means as the site determines as the most reliable method. All attempts at contact will be documented in the participant's source documents.

Should a study site close, e.g., for operational, financial, or other reasons, and the Investigator cannot reach the participant to inform them, their contact information will be transferred to another study site.

# 8. Study Assessments and Procedures

## 8.1 Study Procedures

The study procedures to be conducted for each enrolled participant are detailed below.

### 8.1.1 Informed Consent Procedure

Written informed consent must be obtained from each participant prior to them entering the study and before any protocol-directed procedures are performed.

A unique participant identification number (participant number) will be assigned to each participant at the time; this participant number will be used throughout the study.

### 8.1.2 Medical History and Demographic Data

Demographic data and complete medical history for the participants will be recorded in the eCRF at screening (day -28 to -1). The demographics include date of birth, gender, and race. Any medical conditions developing during the screening period from day -28 to -1 will be collected at study Day 1 (Visit 1), and recorded in the medical history section in eCRF.

Relevant history/conditions include all those present prior to the administration of the study vaccine, which are listed below:

- Relevant medical history
- All current medical conditions
- Allergy history

Whenever possible, diagnoses should be recorded along with their related symptoms.


This document is confidential.

### 8.1.3 Physical Examination and Vital Signs

General physical examination (GPE) and determination of vital signs should be conducted by the Investigator or designated medically trained clinician. A history-directed GPE is done at screening and prior to administration of vaccination at Visit 1. An abbreviated, symptom-directed examination based on any clinically relevant issues or symptoms, and medical history will be done at all other subsequent time points. Any abnormality or change in severity found will be judged as clinically significant / non-significant by the Investigator and will be documented in the eCRF.

Vital signs (including systolic/diastolic blood pressure in sitting position preceded by at least 5 minutes of rest in a quiet setting without distractions [e.g. television, cell phones], pulse rate, and body temperature [tympanic measurement preferred, or in accordance with the local standard of care]) will be performed according to Table 1. Participants with fever defined as tympanic temperature ≥ 38.0°C (100.4°F) should not receive a study vaccine.

Body weight will be recorded in kilograms (kg) to 1 decimal place (lightly clothed without coat and footwear), and body height (without footwear) will be measured in centimeters (cm) without decimal places at the screening visit.

### 8.1.4 Nasopharyngeal (NP) Swab

A nasopharyngeal (NP) swab will be collected by the study staff on check in. Specimens should be transported to the local laboratory on the same day and as soon as possible. Before transportation, specimens must be stored properly in a designated container (as per the corresponding laboratory-specific requirements). The analysis by COVID-19 PCR test will be performed as per local regulations and institutional guidelines.

### 8.1.5 Clinical Laboratory Evaluation

The safety laboratory testing will be performed at the local laboratory following the laboratory's guidelines. Copies of laboratory accreditation certificates and reference ranges will be provided to the Sponsor prior to the analysis of the first patient sample. Blood samples for the laboratory tests for hematology and biochemistry will be collected as per Table 1 (schedule of activities). The volume of blood to be collected at each visit is described in Appendix 2. Lab results will be directly imported via electronic data transfer into the main database. The following is a list of laboratory evaluations to be performed during the study according to the local laboratory instructions:


This document is confidential.

- **Hematology**: hemoglobin, red blood cell (RBC), white blood cell (WBC) with differential count and platelet count
- **Biochemistry**: total bilirubin, creatinine, blood urea nitrogen (BUN), creatinine, alanine transaminase (ALT), aspartate aminotransferase (AST)
- **Serology**: Blood samples will be taken at the screening visit only for determination of active infections for hepatitis B virus (HBV), hepatitis C virus (HCV), and HIV antibody testing
- **Urine pregnancy test** (WOCBP only): hCG

Pregnancy tests may be urine or serum tests and must have a sensitivity of at least 25 mIU/mL. Pregnancy tests will be performed in WOCBP at the times listed in the SoA (Table 1). A negative pregnancy test result will be required prior to the participant's receiving the study intervention. Pregnancy tests may also be repeated if requested by IRBs/ECs or if required by local regulations. In the case of a positive confirmed pregnancy before the study vaccination, the participant must be promptly withdrawn from the study.

### 8.1.6 Diary and Measurement Devices

During the observation period, participants will be provided with a measurement device template for measuring (in millimeters) solicited local AEs of erythema (redness) and swelling and an oral digital thermometer for recording daily temperature (in degrees Celsius). Participants will also be provided with a diary and will be shown how to enter their data in the diary. Each participant will be provided with the following instructions / information on the measurements they are to make:

- From the evening of Day 1 to the evening of Day 7, the participant will measure their oral temperature at approximately the same time each evening and will record the results.
- The oral temperature should not be collected immediately after consumption of a hot or cold beverage or after smoking.
- The participant is to also take their temperature if they feel feverish and to record the highest temperature of the day. In the event that a temperature ≥ 38.0°C or ≥ 100.4°F is obtained (defined as fever), the participant will be allowed to take over-the-counter antipyretics (e.g., acetaminophen/paracetamol, aspirin, naproxen, or ibuprofen) and will be advised to increase the frequency of oral temperature measurements, until they are no longer febrile. The participant is to document medication intake, which will be reviewed by the site personnel.
- How to measure any solicited local AEs, including erythema (redness) and swelling diameter at the injection site using the measurement template supplied for this purpose; participants will also be requested to evaluate pain at the injection site. Local AEs will be assessed every day starting in the evening of Day 1 and up to the evening of Day 7, and the results will be recorded.

- How to grade, daily from the evening of Day 1 through to the evening of Day 7, each of the solicited systemic AEs and their severity and to record the worst grade of the day for each of these solicited systemic AEs. The instructions will include how to record any unusual feeling and/or swelling.
- Participants will be advised that they will be asked about the occurrence of any symptoms or events requiring medical attention and the use of any concomitant medication during the 28-day post-vaccination period, after the vaccination, and until the end of the study.
- Participants will be instructed to contact the clinical site for any unsolicited AEs and/or solicited local and systemic AEs greater than Grade 2 (moderate). Based on their condition, the Investigator may request that the participant return to the clinic for evaluation.
- Participants will be advised on emergency contact information (phone contact number) and instructions for contacting study personnel. Participants will be advised to immediately contact the Investigator (or their designee) if any suspected reaction to the vaccination is felt to be significant or of concern, in the event of an SAE, or in the event of a medical emergency.
- Participants will be advised to notify their health care professional(s) (e.g., primary care physician) that they are participating in a clinical research study of a SARS-CoV-2 / COVID-19 vaccine.
- Participants will be advised to report any COVID-19-like symptoms they may be experiencing, or if they are tested positive for COVID-19, to the clinical site and follow all the recommended actions (e.g., quarantine, re-testing) by local Public Health authorities.

### 8.1.7 Telephonic Safety Follow-up

For all phone contacts the following will apply:

- Ask the participants about any difficulties in recording their data, any change in health, any visits to health care facilities and/or medical practitioners, and any use of concomitant medications. Record the information in the source documents.
- For any unsolicited AEs and/or solicited local and systemic AEs greater than Grade 2 (moderate), the Investigator should be informed within 24 hours of the time the clinical site is made aware of the event. The Investigator may request that the participant return to the clinic for evaluation.
- Advise participants to immediately contact the Investigator (or their designee) in the event of any AE that requires medical attention.


This document is confidential.

### 8.1.8 COVID-19 assessment

- Ask the participants if they have experienced any COVID-19-like symptoms or were tested positive for COVID-19.
- Instruct the participants to contact the clinical site immediately if they experience any COVID-19-like symptoms, or are tested positive for COVID-19.
- Results are recorded in the CRF using the WHO clinical progression scale for COVID-19[37]:

Table 4: COVID-19 Assessments

| Patient State | Descriptor | Score |
|---|---|---|
| Uninfected | Uninfected; no viral RNA detected | 0 |
| Ambulatory mild disease | Asymptomatic; viral RNA detected | 1 |
| | Symptomatic; independent | 2 |
| | Symptomatic; assistance needed | 3 |
| Hospitalized: moderate disease | Hospitalized; no oxygen therapy* | 4 |
| | Hospitalized; oxygen by mask or nasal prongs | 5 |
| Hospitalized: severe diseases | Hospitalized; oxygen by NIV or high flow | 6 |
| | Intubation and mechanical ventilation, pO2/FiO2 ≥150 or SpO2/FiO2 ≥200 | 7 |
| | Mechanical ventilation pO2/FIO2 <150 (SpO2/FiO2 <200) or vasopressors | 8 |
| | Mechanical ventilation pO2/FiO2 <150 and vasopressors, dialysis, or ECMO | 9 |
| Dead | Dead | 10 |

ECMO=extracorporeal membrane oxygenation. FiO2=fraction of inspired oxygen. NIV=non-invasive ventilation. pO2=partial pressure of oxygen. SpO2=oxygen saturation. *If hospitalized for isolation only, record status as for ambulatory patient.


This document is confidential.

## 8.2 Visit Schedules

A detailed description of study assessments is provided in Table 1.

**Screening Visit (within 28 days of enrollment)**

- Informed consent
- Inclusion/exclusion criteria
- Demographic details including age, sex, height, and weight
- Medical history
- History-directed physical examination
- Vitals signs
- Recording of AEs
- Clinical laboratory test (hematology, chemistry, and serology)

**Check-in (within 48 hours before dosing)**

- Inclusion/exclusion criteria
- Update of medical history
- Provision of emergency contact card, thermometer, and measuring device/ruler
- History-directed physical examination
- Vitals signs
- Recording of adverse events
- Blood sample collection for immunogenicity analysis
- Clinical laboratory test (hematology and chemistry; only if more than 7 days have passed since screening)
- Urine pregnancy test for female participants of childbearing potential only
- NP swab sample for COVID-19 PCR

**Dosing (Day 1)**

- Randomization
- Dosing
- Vitals (includes blood pressure, pulse rate, and temperature) will be performed at pre-dose, 1, 2, 3, and 4 hours (±30 minutes) post-dose for the first 5 participants in the standard and high dose group and at pre-dose and 30 minutes post-dose for all other participants
- Recording of adverse events
- A diary card will be issued to the participants

**Day 3**

A telephonic safety follow-up will be performed on Day 3.


This document is confidential.

**Day 7 (± 2 days)**

- Abbreviated, symptom-directed physical examination
- Vital signs
- Recording of adverse events
- Clinical laboratory evaluation (hematology and chemistry)
- Blood sample collection for immunogenicity and cellular response analysis
- NP swab sample for COVID-19 PCR
- Review and collection of Diary Card
- Recording of COVID-19 events

**Day 14 (± 2 days), Day 28 (± 2 days), and Day 84 (± 7 days)**

- Recording of adverse events
- Blood sample collection for immunogenicity and cellular response analysis
- Confirm with female participants of childbearing potential and male participants who have a female partner if they/their partner became pregnant since Day 1
- Recording of COVID-19 infections and vaccinations since Day 1
- NP swab sample for COVID-19 PCR on Day 14 and Day 28

**Day 168 (± 14 days) (End-of-Study)**

- Abbreviated, symptom-directed physical examination
- Vital signs and weight
- Review of medical history
- Recording of adverse events
- Blood sample collection for immunogenicity analysis
- Recording of COVID-19 infections and vaccinations since Day 1
- Confirm with female participants of childbearing potential and male participants who have a female partner if they/their partner became pregnant since Day 1 of the study.

## 8.3 Safety Assessments

Planned time points for all safety assessments can be found in the respective schedule of activities (Table 1). Unscheduled clinical laboratory measurements may be obtained at any time during the study to assess any perceived safety issues.

- Acute reactions within the first 4 hours after administration of the study intervention (for the first 5 participants vaccinated in each dose level), and within the first 30 minutes (for the remainder of participants), will be assessed and documented in the AE CRF.
- Physical examination and vital signs of the participants will be done at various time points during the housing period and on all outpatient visit days defined in the respective schedule of events.
- Procedures to avoid injury from fainting while dosing will be in place.


This document is confidential.

- Emergency equipment will be available on site, and appropriate treatment will be instituted as soon as possible in the event of anaphylaxis or any other immediate hypersensitivity reaction. The Investigator will be on-site on vaccine administration days and for the duration of the observation period for the last participant dosed on that day. The Investigator will be available on call for the remainder of the study. A physician will be immediately available at the clinical site to administer treatment or to apply procedures for any immediate AEs/SAEs.
- All participants will be monitored specifically for solicited local and systemic adverse events as defined in Table 5 on diary card review, physical examination during visits, and telephonic follow-ups.
- Participants will be questioned about their wellbeing at the time of physical examination and recording of vital signs.
- Hematology and biochemical tests will be done at screening, check-in (if more than 7 days have past since screening), Day 7.
- For female participants of childbearing potential, a urine pregnancy test will be performed on the day of enrollment.

### 8.3.1 Abnormal Findings

Medical laboratory or vital signs and other regular safety checks will be screened for results outside normal limits or the reference range that could indicate adverse findings.

In addition to the safety checks listed in this protocol, some listed checks may be repeated and others may be added in case of abnormal findings at the Investigator's discretion. If the aberration is clinically significant to the Investigator, it should be recorded as an AE. If the data support a clinical diagnosis (e.g., hepatitis in the event of elevated liver enzymes), the diagnosis should be documented as AE.

The severity, duration, resolution, action taken, and relationship to the immunization will be reviewed for each adverse event. Depending on the severity of the adverse effects, the research participant may be withdrawn.

## 8.4 Immunogenicity Assessments

Blood and nasal swab samples will be used only for scientific research. Each sample will be labeled with a code so that the laboratory personnel testing the samples will not know the participant's identity. Samples that remain after performing assays outlined in the protocol may be stored by Alvea.

Unless a time limitation is required by local regulations or ethical requirements, the samples will be stored for up to 10 years after the end of the study and then destroyed. If allowed by the ICD, stored samples may be used for additional testing to better understand the immune responses to the vaccine(s) under study in this protocol, to understand differential intervention responders, and/or for vaccine-related assay work supporting vaccine programs. No testing of the participant's DNA will be performed.

The participant may request that his or her samples, if still identifiable, be destroyed at any time; however, any data already collected from those samples will still be used for this research. The biological samples may be shared with other researchers as long as confidentiality is maintained and no testing of the participant's DNA is performed.

Venous blood samples will be collected for measurement of humoral immune response (serum IgG antibodies, viral neutralization assay, fc effector functions) and analysis for cellular response (T-cells). Immunogenicity assessments for all participants of the study will be performed as per the Schedule of Activities (Table 1).

All immunogenicity assessments will be based on samples analyzed at the central laboratories. The amount of blood to be collected is described in Appendix 2.

### 8.4.1 Binding Antibody Assay

Measurement of SARS-CoV-2 anti-spike and anti-N protein serum IgG for several variants and sub-lineages will be performed by enzyme-linked immunosorbent assay (ELISA).

### 8.4.2 Virus Neutralization Assay

SARS-CoV-2 virus-specific serum neutralizing antibody titers for several variants and sub-lineages will be determined in a pseudotyped or live-virus neutralization assay on thawed frozen serum samples. The sera will be heat-inactivated prior to being used without further treatment in the assays being performed. Each serum will be tested in duplicate.

If available in the central laboratory, neutralizing antibody titers will be determined using World Health Organization (WHO)-certified reference standards and values reported in standardized World Health Organization international units.


This document is confidential.

### 8.4.3 Fc assays

Antigen-specific antibodies will be captured on antigen-conjugated beads and their functional capacity will be probed via an array of Fc-binding proteins.

### 8.4.4 Analysis for Cellular Response

The frequency of SARS-CoV-2 antigen-specific CD4+ and CD8+ T-cells will be measured by flow cytometry. Variant or sub-lineage specific antibodies will be used to capture and detect analytes of activated immune T-cells. A positive control (stimulation with the polyclonal mitogen phytohemagglutinin) and a negative control (cells cultured in the absence of any stimulus) will be carried out with each batch of assays.

## 8.5 Adverse Events

### 8.5.1 Definitions

**Adverse Events (AEs)**

An adverse event (AE) is defined as any undesired medical occurrence in a patient or clinical investigation patient receiving a pharmaceutical product, which does not necessarily have a causal relationship with this treatment. An AE can therefore be any unfavorable sign and unintended sign (including an abnormal laboratory finding), symptom, or disease temporarily associated with the use of a vaccine, whether related to the vaccine or not. This definition includes intercurrent illnesses or injuries and exacerbation of pre-existing conditions. Abnormal laboratory values or changes are not reported as AEs if they are not clinically significant, and they will only be recorded as AEs if a therapeutic action is needed or judged by the Investigators.

Planned hospital admissions and/or surgical operations pre-scheduled prior to the participation in this study for a pre-existing illness or disease from before the vaccine performed during the participation of this study are not to be considered as AEs.

**Treatment-Emergent Adverse Event (TEAE)**

A treatment-emergent adverse event (TEAE) is defined as any event at or after the time of exposure to study vaccine or any event already present that worsens in either intensity or frequency following exposure to the study vaccine, until the end of the participant's participation in the study (i.e., Visit 7, day 168 in Table 1, participant withdrawal, or participant lost to follow-up).

**Solicited Adverse Events**

Solicited AEs will be collected during a seven-day follow-up period (i.e., the day of vaccination and six subsequent days) after the vaccination (Table 1). Each participant will be asked to record the information about any particular local or systemic adverse event and its intensity experienced by them daily in the diary cards provided. Participants' AE information in the diary cards will be reviewed and reported by the Investigator's team.

Each participant will be instructed to contact the Investigator immediately should they manifest any adverse signs or symptoms after administration of vaccines.

Table 5: Solicited Local and General Adverse Events

| Local (at injection site) | Systemic |
|---|---|
| • Pain<br>• Erythema (Redness)[A]<br>• Swelling[A]<br>• Induration[A]<br>• Ecchymosis (bruising)[A]<br>• Tenderness<br>• Itching | • Fever [$\geq$ 38.0°C or $\geq$100.4$^0$F][B]<br>• Headache<br>• Chills/shivering<br>• Fatigue<br>• Nausea<br>• Vomiting<br>• Diarrhea<br>• Malaise<br>• Myalgia (muscle pain)<br>• Arthralgia (joint pain) |

A. Record surface diameter in mm
B. Record tympanic or oral temperature

All other AEs are recorded as grades defined in section 8.5.4

**Intensity of Solicited Adverse Events**

The intensity of the local and systemic solicited adverse events will be assessed as described in Appendix 3, Toxicity Grading Scale (Adapted from FDA's published Guidance Toxicity Grading Scale, 2007[38]).


This document is confidential.

Other adverse events

- AEs other than those listed under the solicited AEs will be considered as AEs and should be reported as AEs even when occurred during a seven-day follow-up period after the vaccination
- AEs listed under solicited AEs but reported seven days after vaccination will also be considered as AEs (i.e., onset of fever seven days after vaccination). These AEs will be assessed throughout the study.

**Unsolicited Adverse Events**

AEs other than those listed under “Solicited Adverse Events” will be considered as unsolicited AEs. In addition, if AEs listed under solicited AEs are reported seven days after vaccination they will also be considered as unsolicited AEs (e.g., onset of fever 7, 28, or 84 days after vaccination). Unsolicited AEs will be assessed throughout the study duration.

**Serious Adverse Events (SAEs)**

Any untoward medical occurrence that:

1. results in death,
2. is life threatening,
3. results in persistent or significant disability/incapacity,
4. results in inpatient hospitalization or prolongs an existing inpatient hospitalization,
5. leads to a congenital anomaly or birth defect, or
6. requires medical or surgical intervention to prevent permanent impairment of function or permanent damage to a body structure

will be considered as an SAE.

Examples of such events are intensive treatments in an emergency room or at home for allergic bronchospasm; blood dyscrasias or convulsions that do not result in hospitalization; other important medical events that may not be immediately life threatening or require hospitalization but may jeopardize the patient or may require intervention to prevent any other of the outcomes listed in the definition above. Medical and scientific judgment may be required.

An AE fulfilling any one or more of these criteria must be reported as a SAE, irrespective of the dose of drug given, and even if it is the result of an interaction or drug abuse. A distinction should be drawn between serious and severe AEs. A severe AE is a major event of its type. A severe AE is not necessarily considered serious. For example, nausea that persists for several hours may be considered severe nausea, but not a SAE. On the other hand, a stroke that results in only a limited degree of disability may be considered a mild stroke but would constitute a SAE.


This document is confidential.

**Adverse Events of Special Interest (AESIs)**

AESIs, based on the Safety Platform for Emergency vACcines (SPEAC)[39], are reported until the end of the safety follow-up period:

1. Cardiovascular: Myocarditis, pericarditis
2. Dermatologic: cutaneous vasculitis
3. Hematologic: Coagulopathy, stroke, venous thromboembolism including pulmonary embolism, other thrombosis, thrombocytopenia, and endothelial dysfunction
4. Neurologic: Acute disseminated encephalomyelitis (ADEM), aseptic meningitis, encephalitis, facial nerve palsy including Bell's palsy, Generalized convulsive seizure, Guillain Barre Syndrome (GBS), myelitis, Sensorineural hearing loss (SNHL)
5. Respiratory: Acute Respiratory Distress Syndrome (ARDS)
6. Systemic: Anaphylaxis, Multisystem Inflammatory Syndrome in Children and Adults (MIS-C/A), Vaccine Associated Enhanced Disease (VAED)
7. Vasculitis: Single organ cutaneous vasculitis
8. Rhabdomyolysis
9. Subacute thyroiditis
10. Acute pancreatitis

Adverse events of special interest are to be recorded by the Investigator on the AESI page of the eCRF and should be transmitted to the Sponsor within 24 hours by phone, fax, or email.

**AEs Leading to Study Drug Withdrawal**

AEs leading to study drug withdrawal are to be recorded by the Investigator on the AE page of the eCRF during the entire period of the study and should be transmitted to the Sponsor within 24 hours by phone, fax, or email.

### 8.5.2 Recording and Handling of Adverse Events

AEs and SAEs will be continually monitored for or asked about at all visits starting from screening after the participant provided informed consent. The occurrence of AEs should be sought by non-directive questioning (i.e., Have you noticed any change in your health since your last visit?).

AEs should be collected starting from screening (as follows), and given below in Table 6:

- Solicited local and systemic adverse events are collected from Day 1 until Day 7 after the vaccination


This document is confidential.

- Unsolicited adverse events (all other AEs) are collected from screening until Day 28 (Visit 5) after the administered vaccine
- SAEs, AESIs, and AEs leading to study drug withdrawal should be collected starting from screening until the end of study (Day 168, Visit 7 in Table 1) and reported at each visit during the study.

AEs may also be detected when volunteered by the participant during or between visits or through study assessments (examinations, vitals, laboratory tests, etc.).

Table 6: Collection and Timelines for Reporting of Adverse Events (for the Investigative Site)

| Type of Adverse Event | Duration of Collection* | Reporting requirements (for the Site) |
|---|---|---|
| Solicited AEs (local or systemic) | 7 days (Day 1 and 6 subsequent days) after the vaccination | routine in eCRF Solicited AEs page |
| Non serious AEs (unsolicited) | Screening and 28 days (Visit 5) after the vaccination | routine in eCRF AEs page |
| SAEs (all) | during the whole study (Screening until Day 168) | within 24 hours SAE page |
| AESIs | during the whole study (Screening until Day 168) | within 24 hours AESI page |
| AEs leading to study drug withdrawal | during the whole study (Screening until Day 168) | routine in eCRF AEs page |
| Pregnancy | After the vaccination (Day 1 until Day 168) | within 24 hours Pregnancy Report Form |

Note: AEs should be collected starting from screening.

It is the Investigators' responsibility to proactively follow the outcome of each AE or SAE. All non-serious AEs and SAEs will be followed up until the event is resolved or stabilized, study completion, or the participant is lost to follow-up.

### 8.5.3 Documentation of Adverse Events

AEs must be documented on the Adverse Events pages of the eCRF and SAE report form (when applicable) with the following information where appropriate:

1. AE number, term, and description


This document is confidential.

2. Type of AE (AE / SAE / AESI)
3. Site of AE (when applicable)
4. Duration (onset and resolution dates or if continuing at final visit)
5. Severity grade 1-5 (see below 8.5.4 Severity)
6. Whether it constitutes a serious adverse event (SAE)
7. Relationship to study or control vaccine
8. Action taken / treatment required
9. Outcome

### 8.5.4 Evaluating Adverse Events

Each AE will be assessed by the Investigator and / or medically qualified staff with regard to the following:

**Severity**

The Investigator or their designee will provide an assessment of the severity of each AE by recording a severity rating on the respective CRF.

The intensity of the local and systemic solicited adverse events will be assessed as described in USFDA guideline, Toxicity Grading Scale for Healthy Adult and Adolescent Volunteers Enrolled in Preventive Vaccine Clinical Trials (2007) Appendix 2.

For those parameters (solicited and other AEs) not included in the Toxicity Grading Scale, the following definition of severity grading applies:

- **Grade 1 (Mild):**
  Transient or mild discomfort (< 48 hours); no medical intervention/therapy required
- **Grade 2 (Moderate):**
  Mild to moderate limitation in activity - some assistance may be needed; no or minimal medical intervention/therapy required
- **Grade 3 (Severe):**
  Marked limitation in activity, some assistance usually required; medical intervention/therapy required, hospitalization possible or medically significant but not immediately life-threatening; hospitalization or prolongation of hospitalization indicated; disabling; limiting self-care activities of daily living.
- **Grade 4 (Life-threatening):**
  Extreme limitation in activity, significant assistance required; significant medical intervention/therapy required, hospitalization or hospice care probable.

- **Grade 5 (Death):**
  Death related to AE.

**Causality**

The Investigator will be asked to assess all AEs with respect to their causal relationship to the IPs (test and control vaccine). The Investigator will use clinical judgment in their assessment to determine the reasonable possibility that the AE may have been caused by the test and control vaccines. Evaluation of the relationship between the AE and the IPs should be considered based on the following criteria by the Investigator:

- **Definite** – Temporal sequence exists. No other possible causative factor(s) exists and more than a likely possibility that the study drugs/vaccines contributed to the AE. Confirmed by improvement on stopping/reducing the study drugs.
- **Probable** – A reasonable temporal sequence exists. AE is more likely explained by the study drugs than another cause.
- **Possible** – A reasonable temporal sequence exists but some other possible causative factor(s) may exist that could have contributed to the adverse event.
- **Unlikely** – Temporal sequence is non-existent or doubtful and/or it is certain or probable that other factor(s) have been causative. A causal relationship to the study drugs is improbable.
- **Not Related** – No temporal sequence exists. There are other, more likely causes. Administration of the study drugs is not suspected to have contributed to the adverse event

For any SAE where the Investigator has minimal information for the initial report, the Investigator will assess the causality for every event according to the available information at the time. The Investigator may change their opinion of causality after considering follow-up information, amending the SAE report form accordingly. There may be other possible contributing factors applicable to each serious adverse event that will be recorded in the eCRF as well.

**Outcome**

The outcome of AEs at the time of last observation will be defined according to the International Council for Harmonisation (ICH) Topic E2B, ICH Guideline, as follows:

- Recovered/resolved
- Recovered/resolved with sequelae
- Recovering/resolving

- Not recovered/not resolved
- Fatal/results in death
- Unknown

### 8.5.5 Reporting Serious Adverse Events

**Reporting Requirements**

It is imperative for the Investigators to report any SAE that happens during the study, in accordance with the procedures specified in this protocol, to the Sponsor (designee). Any AE that falls under the SAE definition (Section 9.4.1) needs to be immediately reported by the Investigator, even if the PI does not consider the AE to be clinically significant or IP related. The SAE will be recorded in an additional 'Serious Adverse Event Reporting Form'. It is vital to follow these guidelines because all SAEs must be reported to health authorities in a timely manner.

Medical and scientific judgment is to be exercised in deciding whether expedited reporting is appropriate in other situations, such as for important medical events that were not immediately life-threatening or did not result in death or hospitalization but are jeopardizing the participant or require intervention to prevent one of the outcomes listed above.

**SAE Contact Information**

The Investigator must report information on all SAEs within 24 hours of being aware of their occurrence by e-mail (preferred) or by fax to the contact information listed below:

Safety Contact Information:
safety.alvea-vax-p00001@alveavax.com

**Reporting of SAEs to Health Authorities and IRB**

The Sponsor or its designee will be responsible for reporting SAEs that are deemed both possibly related to the study vaccine and unexpected ('unexpected' refers to events that do not appear in the package labeling or in the study vaccine IB) to the regulatory authorities in an expedited manner.

The Investigator will be responsible for reporting the SAEs that meet IRB reporting requirements directly to the relevant Ethical Review Board as soon as possible, and will also provide the Ethical Review Board with any safety reports prepared by the Sponsor or its designee.

All SAEs that are suspected, unexpected serious adverse reactions (SUSARs) should be reported to regulatory authorities by phone or by facsimile as soon as possible but not later than seven calendar days for deaths and life-threatening events, and 15 calendar days for other SAEs after the Sponsor's (or its designee's) initial receipt of the information. These events should be followed to resolution, stabilization, or return to baseline, regardless of the conclusion of the study.

The Principal Investigator must report all SAEs to the Sponsor's representative/medical expert and to the IEC that has given approval to the study protocol at that center within 24 hours of the occurrence.

**Discontinuation of the Study due to Adverse Events**

The reason for a participant being discontinued from the study will be recorded in the CRF. A discontinuation occurs when an enrolled participant ceases participation in the study, regardless of the circumstances, prior to the completion of the study. A discontinuation must be reported immediately to the Sponsor if it is due to a SAE. The final evaluation as required by the protocol (see Table 1 Visit 7, day 168) will be performed at the time of discontinuation if medically acceptable or as soon as possible after that. The Investigator will record the reason for study discontinuation and, if possible, provide or arrange for appropriate follow-up and document the cause of the participant's condition.

### 8.5.6 Pregnancy

Females of childbearing potential must be confirmed to be not pregnant before vaccine administration and must be instructed that an effective contraceptive method must be used from screening until at least 84 days after vaccination. A pregnancy test will be conducted at Visit 1 before vaccination.

Pregnancy information for female participants should be collected following administration of the first dose of vaccine at Day 1 until the end of the study. Pregnancy information for the female partner of male participants should be collected following administration of the first dose of vaccine at Day 1 until Day 84. Pregnancy, if occurring during the defined time frame, should for reporting purposes be recorded as a non-serious adverse event with the severity grade as severe on the Adverse Event eCRF and Pregnancy Form and reported by the Investigator within 24

hours of learning of its occurrence to the Sponsor / CRO using the same timelines as those for SAEs. Pregnancy as such should not be reported as a SAE. Each pregnancy should be followed up by the Investigator utilizing the follow-up Pregnancy Report.

All pregnancies should be followed up until completion and the outcome assessed in the shortest possible time, but not more than 30 days after completion of the pregnancy. Follow-up should be to determine the pregnancy status as the outcome and the details of the birth. Any SAE experienced during pregnancy (including spontaneous / therapeutic / voluntary termination, birth defects/congenital abnormalities, stillbirth, neonatal death, or any maternal and/or new-born complications) within the study period must be reported on the SAE Report Form using the same timelines as those for reporting of SAEs, by phone and / or fax to the Sponsor (or designee) within 24 hours of knowledge of the event.

Following vaccination, information on all pregnancies in female participants will be gathered until the follow-up appointment. All pregnancy instances will be recorded from the beginning of IP therapy (whenever reported or observed) until collection of the last sample in the study and completion of all post-treatment procedures. All pregnancies will be tracked until they are finished or terminated. Any female participant who becomes pregnant while participating in this study will have her pregnancy information collected by the Investigator. Within 24 hours of learning of a participant's pregnancy, information will be documented in the Pregnancy Reporting Form and forwarded to the Sponsor. The result of the pregnancy (how the pregnancy takes its course) will be monitored by the Investigator. The Investigator or Sponsor can request additional information on the participant’s pregnancy; however, requests will likely not be asked for after 6-8 weeks postpartum. If the pregnancy is terminated, it must be reported, regardless of the rationale for the termination. Data on babies born after the pregnancy must be recorded if the pregnancy is carried to term.

Pregnancy complications or terminations will be recorded as AEs or SAEs, provided that it is not considered an AE for a participant to electively terminate their pregnancy if it is not related to a medical rationale. Spontaneous abortions, fetal death, stillbirth, congenital anomalies, and ectopic pregnancies will be considered SAEs and the Sponsor must be notified of them.

The Investigator can reasonably consider a post-study SAE related to a pregnancy as being related to the study treatment and report the findings to the Sponsor. There is no formal obligation of the Investigator to find information on former participants; they may identify SAEs post-study through random reporting.

## 8.6 Adherence to Protocol

Good Clinical Practice (GCP) and all guidelines outlined in this document must be followed by the Investigator. Guidelines given to participants in this document must also be followed. The


This document is confidential.

Investigator has the right to withdraw the participant from the study if they do not follow said guidelines.

### 8.7 Protocol Deviations

Any actions not compliant with the protocol guidelines are considered a deviation. These actions could be the deviation of the participant, Investigator, or other clinical trial staff. Remedial actions may be created and enacted by the site as they see fit for any deviations.

Protocol deviations must be outlined and documented. This document should be kept in two places: (1) the participant's source document and (2) the site file. Local IECs should be notified of protocol deviations, as recommended by respective IECs. It is the duty of the Investigator and all its employees to understand and act upon the recommended notification process to IECs for deviations.

### 8.8 Independent Medical Monitor

The IMM will routinely monitor safety data. The role of the IMM is that of an advisory, reviewing the safety of participants throughout the trial. To review the safety of the participants, aggregate and individual study data will be shared with the IMM. If the IMM identifies any safety concerns after his / her review, they will notify the Sponsor and outline a suggested rectification strategy.

Primary endpoint immune response data will be reviewed by the IMM following the completion of the Day 28 visit (or early discontinuation) for all participants enrolled in order to determine whether participants who got the investigational product shall be advised to get a standard of care booster vaccination.

## 9. Statistical considerations

### 9.1 Statistical Hypotheses

Since the study is a phase 1 study primarily assessing safety, all data will be analyzed descriptively without a formal statistical hypothesis.

### 9.2 Sample Size Determination

Due to a number of exploratory analyses built into the study design, we used a heuristic approach to select group sample sizes sufficient to assess safety across the five dosing groups and explore immunogenicity of the vaccine under study compared with the active control vaccine.


This document is confidential.

Groups of 40 participants each will be enrolled in the standard dose and control arms of vaccinated individuals to assess safety and variability in immune response relative to baseline immunogenicity. Forty is the median group size in a recent systematic analysis of clinical trial sample sizes in viral diseases[52].

The safety and immune response of the standard dose in vaccinated individuals will be compared with groups of 20 participants each in the low dose and high dose arm in vaccinated individuals.

Ten participants will be enrolled to assess safety and confirm seroconversion for SC injections.

## 9.3 Populations for Analyses

### 9.3.1 Safety Population

The safety population is the set of all enrolled participants who have been administered a dose of the investigational product. Participants will be grouped as treated.

### 9.3.2 Modified Intent-to-Treat Population (mITT)

All enrolled participants who have been administered with the vaccine and experience at least one post-baseline immunogenicity readout will comprise the mITT population. Missing or non-evaluable measurements will not be replaced. Participants will be grouped as treated.

### 9.3.3 Per-Protocol (PP) Population

The per-protocol population analysis includes all enrolled participants who meet all the inclusion/exclusion criteria and do not have any major protocol deviations. Participants will be grouped as treated.

## 9.4 Statistical Analysis

### 9.4.1 General Approach

A statistical analysis plan (SAP) will be prepared and will include details of the immunogenicity and safety analysis. The SAP will be finalized prior to the first analysis of study data. Statistical analysis will be performed using SAS® software (version 9.4 or higher; SAS Institute Inc., USA) or R (version 4.0 or higher, R Foundation for Statistical Computing).

A detailed data management plan will be available for the study.


This document is confidential.

For medical information, the following dictionaries will be used:

- The latest version of the Medical Dictionary for Regulatory Activities (MedDRA) for adverse events and medical history
- The WHO Drug Dictionary for prior and concomitant medications

### 9.4.2 Primary Endpoint Analysis

The overall number of patients and incidence proportion (number of participants experiencing any AEs by the total number of participants) of any solicited local and systemic AEs within seven days of dose administration will be presented. The incidence of each solicited AE (at MedDRA preferred term - PT and System Organ Class - SOC level) will also be shown.

The overall number and incidence proportion of unsolicited AEs within 28 days of vaccination will be analyzed in a similar fashion.

SAEs, AESIs, and AEs leading to participant discontinuation will be presented in the form of a listing, with number and proportion for each one. In addition, the overall incidence proportion of these categories of AE will be shown.

For patient level tabulation a maximum intensity and highest relationship to investigational drug will be presented in separate tables.

### 9.4.3 Secondary Endpoints Analysis

Description of GMT, change in GMT, and GMFR of anti-spike protein (S) IgG antibody and the change in GMT and GFR of anti-N protein (N) IgG antibody at Day 28.

The number and percentage of participants being found to be SARS-CoV-2 positive by external testing (score 1-10), number and percentage of participants with ambulatory mild disease (score 1-3), number and percentage of participants hospitalized with moderate disease (score 4-5), number and percentage of participants hospitalized with severe disease (score 6-9), number and percentage of participants who died (score 10) while being SARS-CoV-2 positive with the exact 95% CI will also be presented separately for each day: Day 7, Day 14, Day 28, Day 84, and Day 168.

### 9.4.4 Safety and Tolerability Analysis

The safety and tolerability of the medicine will be determined using the safety analysis population, which will include all enrolled participants who are known to have received a dosage


This document is confidential.

of the study vaccine or control vaccine. The following descriptive statistics will be used to summarize all safety parameters (clinical laboratory testing, physical examinations, and vital signs): number, mean, median, standard deviation, and minimum and maximum for continuous variables, and participant counts and percentages for categorical variables.

Visit-by-visit tabulations of aberrant clinical laboratory values for the parameters will be reported. Shift tables will be created, displaying the number and percentage of patients who have changed their result category from baseline (check-in visit or screening).

Concomitant medication taken during the study period will be described.

Shift tables presenting numbers and percentages of patients that have changes in category of result in comparison to baseline (check-in visit or screening - for electrocardiogram data) will be produced. Concomitant medications taken during the study period by participants will be detailed separately.

### 9.4.5 Baseline Characteristics

Participant disposition, reasons for withdrawal, or any other factors, as appropriate, will be used to summarize demographic and baseline data. All continuous variables, such as age, will be represented by number, mean, standard deviation, minimum, median, and maximum unless otherwise noted. Gender will be one of the categorical variables given as counts and percentages along with other categorical variables.

### 9.4.6 Exploratory Analyses

Characterization of humoral antibody immune response of booster vaccinations against Ancestral and Variant (Alpha, Beta, Delta, BA.1, and any newly discovered VOC) SARS-CoV-2 Strains measured using GMT of anti-SARS-CoV-2 neutralizing antibody at each timepoint (Pre-vaccination, Day 7, Day 14, Day 28, Day 84, and Day 168) will be made using the approach described for the primary endpoint analyses.

Characterization of the cellular immune response following booster vaccinations against SARS-CoV-2 BA.2/Omicron measured using cellular immune responses CD4+ and CD8+ T-cell response.

DocuSign Envelope ID: 2B72DF38-444D-4144-86F5-185A92D63B30

The RLU and change in RLU of serum antibody-dependent cell-mediated cytotoxicity (ADCC) against existing and yet to be defined variants and sub-lineages at the defined time points will be presented unadjusted in tabular form.

Additional exploratory analysis will be described in the statistical analysis plan.

### 9.4.7 Analysis timepoints

Results will be analyzed upon primary endpoint and the completion of the study.

Interim review of safety findings will occur during the study. Interim summary of immune response may occur periodically through the study for phase 2 planning. No modification to this study will occur based on these analyses. No corrections for multiple testing are planned as these analyses will be used only to inform phase 2 study design.

Additional, supplemental safety and immune response results will be provided upon the completion of the trial.

## 9.5 Handling of Missing Data

Any missing immunogenicity and safety data will not be imputed and will be considered as missing only. Measurable antibody titers below the cut-off for quantification of the assay will be given an arbitrary value of half the cut-off value of the assay for the purpose of GMT and GMFR calculations. Unmeasurable antibody titers will be given a value of zero for the purpose of GMT and GMFR calculations.

# 10. Administrative Matters

This Study Protocol is designed to ensure that Alvea, LLC and the Investigator act in accordance with the principles of the WHO GCP for the conduct, evaluation, and documentation of this study. The trial will be registered on the Clinical Trial Registry of the respective National Regulatory Authority (NRA), i.e. the South African National Clinical Trials Register, before enrollment of the first participant in the trial. Independent Ethics Committee notifications as per GCP guidelines issued by the WHO will also be followed during the conduct of the study.


This document is confidential.

## 10.1 Ethics

### 10.1.1 Ethical Conduct of the Study

The study will be conducted in accordance with the protocol of the ICH Good Clinical Practice (GCP) guidelines ethical principles, which have their origin in the Declaration of Helsinki, 2008 version, US Investigational New Drug (IND) regulations (21 Code of Federal Regulations [CFR] 56), or all local regulations (as applicable).

The Investigator must assure that the study is conducted in accordance with the provisions as stated in the ICH GCP guidelines and complies with prevailing local laws and regulations. Furthermore, the participation of participants in this study should be reported to the appropriate local data protection agencies, in accordance with GCP. The principal Investigator is to assure that appropriate training relevant to the study is given to the medical, nursing, and other staff involved. Any information relevant to the performance of this study is to be forwarded to the co-investigators and other staff involved.

### 10.1.2 Independent Ethics Committee Review and Communications

In accordance with local requirements, this study will be submitted to the regulatory authorities for approval/notification. This Protocol, corresponding CRF and Informed Consent Form, will be reviewed by the IEC prior to initiating the study. The first participant must be enrolled only after the IEC approves the Protocol and CRF for the study. IEC notifications as per GCP guidelines issued by the WHO will be followed during the conduct of the study.

### 10.1.3 Informed Consent and Participant Information

Written informed consent will be obtained from each participant prior to enrollment in the study.

Before the study begins, the Investigator and/or designated study site personnel will explain the purpose, procedures to be followed, potential hazards, available alternative therapeutic modalities, and rights of the participants to the participant (in English or in a language understandable to the participant). Prior to enrollment of the participant in the study, the participants will be required to understand and sign the ICF (in English or in a language understandable to the participants), and a signature of an impartial witness (if applicable) and the study personnel obtaining consent will be required. Each person's signature, as well as the date, must be included.


This document is confidential.

If the participant is illiterate and/or their legal representative(s) or guardian(s) is illiterate, verbal consent should be obtained in the presence of and countersigned by a literate witness. The participant and/or the participant's legal representative(s) or guardian(s), the PI or person designated by the PI, and if applicable, a literate witness must personally sign the ICF. Further, the SA-GCP states that the participant should indicate willingness to participate by making a mark (either a cross or a fingerprint). The witness signs to affirm that the participant willingly consented to participate. The witness dates the mark and their signature. For participants deemed vulnerable, an audio-video recording of the informed consent process may be used as the Investigator sees fit.

No participant should be enrolled without their explicit informed consent. Because Alveavax-v1.2 is a new medicine, the Regulatory Authority has yet to authorize it anywhere in the country.

Participants who voluntarily discontinue or who are withdrawn from the study will be advised to receive a standard of care licensed booster vaccine within up to 90 days where appropriate.

### 10.1.4 Clinical Trial Insurance and Compensation to Participants

Insurance will be in place before enrollment of trial participants. The trial will not be initiated until the insurance certificate is provided. In the event of study-related injury or death, insurance for the participants and indemnity of the Investigators and those of their employees, servants, or agents, whose participation in this study has been documented, are provided. Insurance and liability will be in accordance with the applicable law and GCP.

If it can be demonstrated that a participant suffers any significant deterioration in health or well-being or any harmful susceptibility or toxicity as a direct result of their participation in this study, the Sponsor will agree to abide by GCP guidance and legal requirements, in accordance with local regulations, with regard to compensation payable to the participant. The amount of compensation will be calculated by reference to the level of damages commonly awarded in law in the participating countries for similar injuries at the time when such injury occurred.

Participants will receive no benefit except for compensation for the time and inconveniences that may arise from participation in the study.

### 10.1.5 Participant Confidentiality and Disclosure

All information and data collected in the study will be kept confidential by the Investigator and other site staff. It is to be ensured by the Investigator that all documents that are given to any


This document is confidential.

third party do not contain the names or addresses of the participants, or other information that would affect the anonymity of the participants. All persons involved in the study are bound by this confidentiality clause.

Participants should be informed in writing that their confidentiality will be maintained in accordance with local laws when their data and results are stored and analyzed by a computer, or when hospital records relevant to the study are reviewed or audited by authorized representatives of the company and/or regulatory authority.

## 10.2 Records

### 10.2.1 Monitoring, Quality Assurance, and Inspection by Health Authorities

**Monitoring**

Monitoring will be performed by a qualified monitor, who will ensure that the study is conducted and documented properly according to the protocol, current SOPs, GCP, and other applicable guidelines, manuals, and regulatory requirements. The monitor will be in contact with the site before, during, and after the study.

The CRO's monitor assigned to the trial will undertake routine site-monitoring visits. The timing, frequency, and duration of the monitoring visits will be determined by the Sponsor and based on the protocol requirements and any applicable contract/agreement(s) with the Sponsor and/or institution.

**Quality Control and Quality Assurance**

The Sponsor will implement and maintain quality assurance and quality control systems with written SOPs to ensure that trials are conducted and data are generated, documented (record), and reported in compliance with the protocol, GCP, and applicable regulatory requirement(s).

The Sponsor or Sponsor's designee may arrange to inspect/audit the clinical study at the investigational site. The auditor is independent from the clinical monitoring and project management team at the Sponsor. The audit may include on-site review of regulatory documents, case report forms, and source documents. Direct access to these documents will be required by the auditors.

**Audit and Inspection**

To ensure compliance with GCP and all applicable regulatory requirements, the Sponsor (or its designee) may conduct quality assurance audits at any time during the study or after study completion. Regulatory agencies may also conduct a regulatory inspection of this study.

If an audit or inspection occurs, the Investigator will allow the auditor/inspector direct access to all relevant documents (source data, eCRFs, study files) and allocate their time and the time of their staff to the auditor/inspector to discuss findings and any relevant issues.

If the Investigator is notified of a scheduled competent authority inspection, they must notify the Sponsor immediately (within 24 hours of notice). The same rule applies to unannounced audits and inspections.

### 10.2.2 Case-Report Forms

Paper or electronic CRFs will be used in the study. If paper CRFs are utilized in the study, all entries must be written in a legible manner with a ballpoint pen (not pencil, felt tip, or fountain pen). The Investigator must enter all data obtained during the trial on the CRFs produced by Alvea, LLC. The Investigator must provide a fair justification for any missing data. The Investigator must sign the completed CRFs. If the Investigator authorizes others to make entries in the CRF, Alvea, LLC must obtain the names, roles in the study, signatures, and initials of those individuals. If the findings are entered in the CRF by someone other than the Investigator, that individual must also sign the CRF. If the Investigator or designate(s) makes changes to entries in the CRF, the words or figures must be crossed out, keeping the original entry readable. Then the adjustment must be dated and initialed. Incorrect entries must not be erased or otherwise rendered unreadable. CRFs must be updated on a regular basis. The Sponsor will make arrangements to obtain a copy of the CRFs once they are completed. Missing or incorrect data input in the CRF should be double-checked with the Investigator and rectified accordingly before data entry. The Sponsor will make arrangements to collect a copy of those CRFs.

If the study uses an electronic CRF (eCRF), the module will be built around the CRF and the unscheduled visit form. The site workers will use their individual login IDs and passwords to enter the data into the eCRF. The CRA will then compare the data entered in the eCRF to the source papers. If there is a mismatch between the data provided in the eCRF module and the data in the source document, the discrepancy must be addressed. The Principal Investigators at each site will be accountable for electronically validating the data recorded in each participant's eCRF. After the clinical study is finished, a print copy of all the participants' completed eCRFs will be kept at the clinical trial locations and at the Sponsor's site.

### 10.2.4 Recruitment Procedures

Advertisements to be used for the recruitment of study participants, and any other written information regarding this study to be provided to the participant should be submitted to the Sponsor for approval. All documents must be approved by the IRB.

### 10.2.5 Source Documents

Source documents are proof of the participant’s involvement in the study and ensure that the collected data is reliable. The Investigator’s site will keep the source documents.

Data reported on the CRF or entered in the eCRF that are from source documents must be consistent with the source documents or the discrepancies must be explained. The Investigator may need to request previous medical records or transfer records, depending on the study. Also, current medical records must be available.

### 10.2.6 Direct Access to Source Data/Documents

Monitoring, audits, IEC review, and regulatory inspection for the trial are permitted by the Investigator. This allows Investigators to have direct access to necessary source data/documents. Source documents, including but not limited to progress updates and copies of laboratory and clinical test results, must be accessible for the Sponsor to review at any given time.

### 10.2.7 Trial Monitoring

The Investigator will permit the Sponsor / their designees to monitor the study as frequently as deemed necessary to determine that data recording and protocol adherence are satisfactory.

### 10.2.8 Investigator Site File and Archiving

The eCRFs will be printed and sent to the Sponsor for archiving upon request after the statistical report is finalized. All study documentation pertaining to the conduct of the study at the study site must be retained by the Investigator for a period to be mutually agreed in writing by the Investigator and the Sponsor. The Sponsor will notify the Investigator in writing when retention is no longer necessary.

### 10.2.9 Dissemination of Clinical Study Data

All information, including but not limited to information regarding Alveavax-v1.2 or the Sponsor's operations (eg, patent application, formulas, manufacturing processes, basic scientific data, prior clinical data, formulation information) supplied by the Sponsor to the Investigator and not previously published, and any data, including exploratory biomarker research data, generated as a result of this study, are considered confidential and remain the sole property of the Sponsor.


This document is confidential.

DocuSign Envelope ID: 2B72DF38-444D-4144-86F5-185A92D63B30

The Investigator agrees to maintain this information in confidence and use this information only to accomplish this study and will not use it for other purposes without the Sponsor's prior written Consent.

The Investigator understands that the information developed in the study will be used by the Sponsor in connection with the continued development of Alveavax-v1.2, and thus may be disclosed as required to other clinical investigators or regulatory agencies. To permit the information derived from the clinical studies to be used, the Investigator is obligated to provide the Sponsor with all data obtained in the study.

The results of the study will be reported in a Clinical Study Report generated by the Sponsor and will contain data from all study sites that participated in the study as per protocol. Recruitment performance or specific expertise related to the nature and the key assessment parameters of the study will be used to determine a coordinating Investigator for the study. Results of exploratory biomarker analyses performed after the Clinical Study Report has been issued will be reported in a separate report and will not require a revision of the Clinical Study Report.

Study participant identifiers will not be used in publication of results. Any work created in connection with performance of the study and contained in the data that can benefit from copyright protection (except any publication by the Investigator as provided for below) shall be the property of the Sponsor as author and owner of copyright in such work.

Consistent with Good Publication Practices and International Committee of Medical Journal Editors (ICMJE) guidelines, the Sponsor shall have the right to publish such primary data and information without approval from the Investigator. The Investigator has the right to publish study site-specific data after the primary data are published. If an Investigator wishes to publish information from the study, a copy of the manuscript must be provided to the Sponsor for review at least 60 days before submission for publication or presentation. Expedited reviews will be arranged for abstracts, poster presentations, or other materials. If requested by the Sponsor in writing, the Investigator will withhold such publication for up to an additional 60 days to allow for filing of a patent application. In the event that issues arise regarding scientific integrity or regulatory compliance, the Sponsor will review these issues with the Investigator. The Sponsor will not mandate modifications to scientific content and does not have the right to suppress information.

For multicenter study designs and sub-study approaches, secondary results generally should not be published before the primary endpoints of a study have been published. Similarly, Investigators will recognize the integrity of a multicenter study by not submitting for publication data derived from the individual study site until the combined results from the completed study have been submitted for publication, within 18 months after the study end date, or the Sponsor

confirms there will be no multicenter study publication. Authorship of publications resulting from this study will be based on the guidelines on authorship, such as those described in the ICMJE Recommendations for the Conduct, Reporting, Editing and Publication of Scholarly Work in Medical Journals, which state that the named authors must have made a significant contribution to the conception or design of the work; or the acquisition, analysis, or interpretation of the data for the work; and drafted the work or revised it critically for important intellectual content; and given final approval of the version to be published; and agreed to be accountable for all aspects of the work in ensuring that questions related to the accuracy or integrity of any part of the work are appropriately investigated and resolved.

### 10.3 Quality Assurance Audit

The Sponsor or CRO will complete an assurance audit to ensure the quality of the trial. All records, including medical records, the Investigator's trial-related files/correspondence, and informed consent forms relevant to the trial, will be available for the auditor to access.

### 10.4 Rules for Amending the Protocol

Extensive conversations with Sponsors are a prerequisite for any amendments to the protocol. These amendments are to be recorded, dated, and signed by all signatories (or their successors) of the premier protocol design. Prior written approval of the Ethics Committee and SAHPRA is another prerequisite for changes to or deviations from the protocol. Changes made to prevent immediate hazards to participants or minor logistical changes do not need written approval; however, the Ethics Committee and SAHPRA must be notified of them within 30 days.

### 10.5 Discontinuation of the Trial by the Sponsor

If there is a well-documented justification, the Sponsor has the right to discontinue the trial at any time. If there is a safety justification, the Principal Investigator has the right to discontinue the trial at any time and must inform the Sponsor of such a decision. If there is a major ethical violation related to any SAE(s), the IRB/IEC has the right to terminate the trial while providing justification to the participants. The IRB/IEC must also submit a summary report to SAHPRA.

The reasons for such action may include, but are not limited to, the following:

- Any safety concerns, considering the rights, safety, and well-being of the participant(s)
- Death, assessed as possibly, probably, or definitely related to investigational product
- If two or more participants experience acute anaphylaxis or bronchospasm, the study will be suspended until a full safety review is performed
- The study is not conducted in accordance with International Council for Harmonisation (ICH) Good Clinical Practice (GCP) Guidelines and local regulations
- The required number of participants have completed the study


This document is confidential.

DocuSign Envelope ID: 2B72DF38-444D-4144-86F5-185A92D63B30

# 11. References

1 Gray GE, Collie S, Garrett N, *et al.* Vaccine effectiveness against hospital admission in South African health care workers who received a homologous booster of Ad26.COV2 during an Omicron COVID19 wave: Preliminary Results of the Sisonke 2 Study. medRxiv, 2021.

2 Access Consortium: Alignment with ICMRA consensus on immunobridging for authorising new COVID-19 vaccines. GOV.UK. https://www.gov.uk/government/publications/access-consortium-alignment-with-icmra-consensus-on-immunobridging-for-authorising-new-covid-19-vaccines (accessed Feb 16, 2022).

3 Guidelines on clinical evaluation of vaccines: regulatory expectations. World Health Organization, 2016.

4 Edupuganti, S., C De Rosa, S., Elizaga, M., Lu, Y., Han, X., Huang, Y., Swann, E., Polakowski, L., A Kalams, S., Keefer, M., Maenza, J., C Wise, M., Yan, J., Morrow, M. P., Khan, A. S., Boyer, J. D., Humeau, L., White, S., Sardesai, N. Y., … The Niaid-Funded Hiv Vaccine Trials Network, null. (2020). Intramuscular and Intradermal Electroporation of HIV-1 PENNVAX-GP® DNA Vaccine and IL-12 Is Safe, Tolerable, Acceptable in Healthy Adults. Vaccines, 8(4), E741. https://doi.org/10.3390/vaccines8040741

5 Mammen, M. P., Tebas, P., Agnes, J., Giffear, M., Kraynyak, K. A., Blackwood, E., Amante, D., Reuschel, E. L., Purwar, M., Christensen-Quick, A., Liu, N., Andrade, V. M., Carter, J., Garufi, G., Diehl, M. C., Sylvester, A., Morrow, M. P., Pezzoli, P., Kulkarni, A. J., … Humeau, L. M. (2021). Safety and immunogenicity of INO-4800 DNA vaccine against SARS-CoV-2: A preliminary report of a randomized, blinded, placebo-controlled, Phase 2 clinical trial in adults at high risk of viral exposure (p. 2021.05.07.21256652). medRxiv. https://doi.org/10.1101/2021.05.07.21256652

6 Morishita, R., Makino, H., Aoki, M., Hashiya, N., Yamasaki, K., Azuma, J., Taniyama, Y., Sawa, Y., Kaneda, Y., & Ogihara, T. (2011). Phase I/IIa clinical trial of therapeutic angiogenesis using hepatocyte growth factor gene transfer to treat critical limb ischemia. Arteriosclerosis, Thrombosis, and Vascular Biology, 31(3), 713–720. https://doi.org/10.1161/ATVBAHA.110.219550


This document is confidential.

DocuSign Envelope ID: 2B72DF38-444D-4144-86F5-185A92D63B30

7 Garcia-Beltran, W. F., Lam, E. C., St Denis, K., Nitido, A. D., Garcia, Z. H., Hauser, B. M., Feldman, J., Pavlovic, M. N., Gregory, D. J., Poznansky, M. C., Sigal, A., Schmidt, A. G., Iafrate, A. J., Naranbhai, V., & Balazs, A. B. (2021). Multiple SARS-CoV-2 variants escape neutralization by vaccine-induced humoral immunity. Cell, 184(9), 2372-2383.e9. https://doi.org/10.1016/j.cell.2021.03.013

8 Garcia-Beltran, W. F., Lam, E. C., St Denis, K., Nitido, A. D., Garcia, Z. H., Hauser, B. M., Feldman, J., Pavlovic, M. N., Gregory, D. J., Poznansky, M. C., Sigal, A., Schmidt, A. G., Iafrate, A. J., Naranbhai, V., & Balazs, A. B. (2021). Multiple SARS-CoV-2 variants escape neutralization by vaccine-induced humoral immunity. Cell, 184(9), 2372-2383.e9. https://doi.org/10.1016/j.cell.2021.03.013

9 Pavlin, B., (2022) Epidemiology of Omicron variant of SARS-CoV-2. World Health Organization. Retrieved February 16, 2022, from https://cdn.who.int/media/docs/default-source/epi-win/webinar-report-epi-win/presesntation_boris_pavlin.pdf?sfvrsn=4f5a18c0_5

10 McIntosh, K. (2022). Coronaviruses. Up To Date. https://www.uptodate.com/contents/coronaviruses

11 McIntosh, K. (2022). COVID-19: Epidemiology, virology, and prevention. Up To Date. https://www.uptodate.com/contents/covid-19-epidemiology-virology-and-prevention

11 CoVariants. (n.d.). Retrieved February 15, 2022, from https://covariants.org/per-country

12 Lyngse, F. P., Kirkeby, C. T., Denwood, M., Christiansen, L. E., Mølbak, K., Møller, C. H., Skov, R. L., Krause, T. G., Rasmussen, M., Sieber, R. N., Johannesen, T. B., Lillebaek, T., Fonager, J., Fomsgaard, A., Møller, F. T., Stegger, M., Overvad, M., Spiess, K., & Mortensen, L. H. (2022). Transmission of SARS-CoV-2 Omicron VOC subvariants BA.1 and BA.2: Evidence from Danish Households (p. 2022.01.28.22270044). medRxiv. https://doi.org/10.1101/2022.01.28.22270044https://doi.org/10.1016/j.cell.2021.02.037

13 Du, P., Gao, G. F., & Wang, Q. (2022). The mysterious origins of the Omicron variant of SARS-CoV-2. The Innovation, 3(2), 100206. https://doi.org/10.1016/j.xinn.2022.100206



This document is confidential.

DocuSign Envelope ID: 2B72DF38-444D-4144-86F5-185A92D63B30

14 Chen, J., & Wei, G.-W. (2022). Omicron BA.2 (B.1.1.529.2): High potential to becoming the next dominating variant. ArXiv:2202.05031 [q-Bio]. http://arxiv.org/abs/2202.05031

15 Omicron subvariant BA.2 likely to have same severity as "original" -WHO | Reuters. (n.d.). Retrieved February 16, 2022, from https://www.reuters.com/business/healthcare-pharmaceuticals/omicron-subvariant-ba2-likely-have-same-severity-original-who-2022-02-01/

16 Edward, K. M., Orenstein, W. A., COVID-19: Vaccines. Up To Date. https://www.uptodate.com/contents/covid-19-vaccines

17 Tebas, P., Roberts, C. C., Muthumani, K., Reuschel, E. L., Kudchodkar, S. B., Zaidi, F. I., White, S., Khan, A. S., Racine, T., Choi, H., Boyer, J., Park, Y. K., Trottier, S., Remigio, C., Krieger, D., Spruill, S. E., Bagarazzi, M., Kobinger, G. P., Weiner, D. B., & Maslow, J. N. (2017). Safety and Immunogenicity of an Anti-Zika Virus DNA Vaccine—Preliminary Report. The New England Journal of Medicine. https://doi.org/10.1056/NEJMoa1708120

18 Tebas, P., Kraynyak, K. A., Patel, A., Maslow, J. N., Morrow, M. P., Sylvester, A. J., Knoblock, D., Gillespie, E., Amante, D., Racine, T., McMullan, T., Jeong, M., Roberts, C. C., Park, Y. K., Boyer, J., Broderick, K. E., Kobinger, G. P., Bagarazzi, M., Weiner, D. B., … White, S. M. (2019). Intradermal SynCon® Ebola GP DNA Vaccine Is Temperature Stable and Safely Demonstrates Cellular and Humoral Immunogenicity Advantages in Healthy Volunteers. The Journal of Infectious Diseases, 220(3), 400–410. https://doi.org/10.1093/infdis/jiz132

19 Muthumani, K., Falzarano, D., Reuschel, E. L., Tingey, C., Flingai, S., Villarreal, D. O., Wise, M., Patel, A., Izmirly, A., Aljuaid, A., Seliga, A. M., Soule, G., Morrow, M., Kraynyak, K. A., Khan, A. S., Scott, D. P., Feldmann, F., LaCasse, R., Meade-White, K., … Weiner, D. B. (2015). A synthetic consensus anti-spike protein DNA vaccine induces protective immunity against Middle East respiratory syndrome coronavirus in nonhuman primates. Science Translational Medicine, 7(301), 301ra132. https://doi.org/10.1126/scitranslmed.aac7462

20 Lauring, A. S., Tenforde, M. W., Chappell, J. D., Gaglani, M., Ginde, A. A., McNeal, T., Ghamande, S., Douin, D. J., Talbot, H. K., Casey, J. D., Mohr, N. M., Zepeski, A., Shapiro, N. I., Gibbs, K. W., Files, D. C., Hager, D. N., Shehu, A., Prekker, M. E., Erickson, H. L., … Network, F. the I. and O. V. in the A. I. (IVY). (2022). Clinical Severity and mRNA Vaccine Effectiveness for Omicron, Delta, and Alpha SARS-CoV-2 Variants in the United States: A Prospective



This document is confidential.

DocuSign Envelope ID: 2B72DF38-444D-4144-86F5-185A92D63B30

Observational Study (p. 2022.02.06.22270558). medRxiv. https://doi.org/10.1101/2022.02.06.22270558

21 Gray, G. E., Collie, S., Garrett, N., Goga, A., Champion, J., Zylstra, M., Reddy, T., Yende, N., Seocharan, I., Takalani, A., Sanne, I., Mayat, F., Odhiambo, J., Bamford, L., Moultrie, H., Fairall, L., Bekker, L.-G., & on behalf of the Sisonke Study Team. (2021). Vaccine effectiveness against hospital admission in South African health care workers who received a homologous booster of ad26.cov2 during an omicron covid19 wave: Preliminary results of the Sisonke 2 Study. medRxiv. Retrieved February 16, 2022, from https://www.medrxiv.org/content/10.1101/2021.12.28.21268436v1.full

22 Ritchie, H., Mathieu, E., Rodés-Guirao, L., Appel, C., Giattino, C., Ortiz-Ospina, E., Hasell, J., Macdonald, B., Beltekian, D., & Roser, M. (2020). Coronavirus Pandemic (COVID-19). Our World in Data. https://ourworldindata.org/covid-vaccinations

23 Capua, I., & Giaquinto, C. (2021). The unsung virtue of thermostability. The Lancet, 397(10282), 1346. https://doi.org/10.1016/S0140-6736(21)00526-2

24 Shafaati, M., Saidijam, M., Soleimani, M., Hazrati, F., Mirzaei, R., Amirheidari, B., Tanzadehpanah, H., Karampoor, S., Kazemi, S., Yavari, B., Mahaki, H., Safaei, M., Rahbarizadeh, F., Samadi, P., & Ahmadyousefi, Y. (n.d.). A brief review on DNA vaccines in the era of COVID-19. Future Virology, 10.2217/fvl-2021–0170. https://doi.org/10.2217/fvl-2021-0170

25 WHO Technical Report Series No 941, Annex 1: WHO Guidelines for assuring the quality and nonclinical safety evaluation of DNA vaccines, 2007.

26 Yang, B., Jeang, J., Yang, A., Wu, T. C., & Hung, C.-F. (2015). DNA vaccine for cancer immunotherapy. Human Vaccines & Immunotherapeutics, 10(11), 3153–3164. https://doi.org/10.4161/21645515.2014.980686

27 WHO Technical Report Series No. 941, WHO Guidelines for assuring the quality, safety, and efficacy of plasmid DNA vaccines, 2020.

28 WHO Technical Report Series No. 1028, Annex 2: WHO Guidelines on the quality, safety and efficacy of plasmid DNA vaccines. 2021.


This document is confidential.

DocuSign Envelope ID: 2B72DF38-444D-4144-86F5-185A92D63B30

29 Guidance for Industry: Considerations for Plasmid DNA Vaccines for Infectious Disease Indications. Biotechnology Law Report. 2007.

30 Momin, T., Kansagra, K., Patel, H., Sharma, S., Sharma, B., Patel, J., Mittal, R., Sanmukhani, J., Maithal, K., Dey, A., Chandra, H., Rajanathan, C. T., Pericherla, H. P., Kumar, P., Narkhede, A., & Parmar, D. (2021). Safety and Immunogenicity of a DNA SARS-CoV-2 vaccine (ZyCoV-D): Results of an open-label, non-randomized phase I part of phase I/II clinical study by intradermal route in healthy subjects in India. EClinicalMedicine, 38, 101020. https://doi.org/10.1016/j.eclinm.2021.101020

31 Beasley, D. W. C. (2020). New international guidance on quality, safety and efficacy of DNA vaccines. Npj Vaccines, 5(1), 1–2. https://doi.org/10.1038/s41541-020-0199-0

32 Sheets, R., Kang, H.-N., Meyer, H., & Knezevic, I. (2020). WHO informal consultation on the guidelines for evaluation of the quality, safety, and efficacy of DNA vaccines, Geneva, Switzerland, December 2019. Npj Vaccines, 5(1), 1–5. https://doi.org/10.1038/s41541-020-0197-2

33 Momin T, Kansagra K, Patel H, *et al.* Safety and Immunogenicity of a DNA SARS-CoV-2 vaccine (ZyCoV-D): Results of an open-label, non-randomized phase I part of phase I/II clinical study by intradermal route in healthy subjects in India. *EClinicalMedicine* 2021; **38**: 101020.

34 Dey A, Chozhavel Rajanathan TM, Chandra H, *et al.* Immunogenic potential of DNA vaccine candidate, ZyCoV-D against SARS-CoV-2 in animal models. *Vaccine* 2021; **39**: 4108–16.

35 Janssen Ad26.COV2.S COVID-19 Vaccine prescribing information. https://www.covid19vaccinejanssen.com/za-en/download/7 (accessed Feb 16, 2022).

36 Create a blocked randomisation list | Sealed Envelope. https://www.sealedenvelope.com/simple-randomiser/v1/lists (accessed Feb 16, 2022).

37 Marshall JC, Murthy S, Diaz J, *et al.* A minimal common outcome measure set for COVID-19 clinical research. *The Lancet Infectious Diseases* 2020; **20**: e192–7.

38 Research C for BE and. Toxicity Grading Scale for Healthy Adult and Adolescent Volunteers Enrolled in Preventive Vaccine Clinical Trials. U.S. Food and Drug Administration.


This document is confidential.

2019; published online May 17. https://www.fda.gov/regulatory-information/search-fda-guidance-documents/toxicity-grading-scale-healthy-adult-and-adolescent-volunteers-enrolled-preventive-vaccine-clinical (accessed Feb 16, 2022).

39 Brighton Collaboration Publications: SPEAC COVID-19 AESI https://docs.google.com/spreadsheets/d/1QgF35nYcsaFN3DZTOtV_lP0TYqQzsDMUQBAd5M9brrM/edit#gid=0&fvid=1937681344 (accessed Mar 12, 2022)

40 Eckardt K-U, Kasiske BL. Kidney disease: improving global outcomes. *Nat Rev Nephrol* 2009; **5**: 650–7.

41 WHO. Guidelines for assuring the quality, safety, and efficacy of plasmid DNA vaccines, Geneva, Switzerland, August 2020. https://www.who.int/biologicals/DNA_vaccines_R_WHO.BS.2020.2380_12_May_2020.pdf (accessed Feb 16, 2022)

42 Mallapaty, S. (2021). India’s DNA COVID vaccine is a world first – more are coming. Nature, 597(7875), 161–162. https://doi.org/10.1038/d41586-021-02385-x

43 Sheridan, C. (2021). First COVID-19 DNA vaccine approved, others in hot pursuit. Nature Biotechnology, 39(12), 1479–1482. https://doi.org/10.1038/d41587-021-00023-5

44 Cadila healthcare limited (2021). Zydus receives EUA from DCGI for ZyCoV-D, the only needle-free COVID vaccine in the world, Ahmedabad, India, August 2021. http://rxdx.in/wp-content/uploads/2021/09/Press-Release-Zydus-receives-EUA-from-DCGI-for-ZyCoV-D.pdf (accessed Mar 10, 2022)

45 Ahn JY, Lee J, Suh YS, Song YG, Choi YJ, Lee KH, Seo SH, Song M, Oh JW, Kim M, Seo HY, Kwak JE, Youn JW, Woo JW, Shin EC, Sung YC, Park SH, Choi JY. Safety and immunogenicity of two recombinant DNA COVID-19 vaccines containing the coding regions of the spike or spike and nucleocapsid proteins: an interim analysis of two open-label, non-randomised, phase 1 trials in healthy adults. Lancet Microbe. 2022 Mar;3(3):e173-e183. doi: 10.1016/S2666-5247(21)00358-X. Epub 2022 Feb 8. PMID: 35156068; PMCID: PMC8824525.



This document is confidential.

DocuSign Envelope ID: 2B72DF38-444D-4144-86F5-185A92D63B30

46 Anges. Novel Coronavirus (COVID-19) DNA Vaccine: Results of Phase 1/2 and Phase 2/3 Clinical Trials, November 2021. https://www.anges.co.jp/pdf_news/public/cqFgW5KW0U5efAL9wJqhdQhRKCKPasRw.pdf (accessed Mar 10, 2022)

47 Phase I/II Study of COVID-19 DNA Vaccine (AG0302-COVID19 High-dose). https://clinicaltrials.gov/ct2/show/NCT04993586 (accessed Mar 10, 2022)

48 WHO, WHO Coronavirus (COVID-19) Dashboard. https://web.archive.org/web/20220228110424/https://covid19.who.int/ (accessed Mar 13, 2022)

49 Tebas, Pablo, Shuping Yang, Jean D. Boyer, Emma L. Reuschel, Ami Patel, Aaron Christensen-Quick, Viviane M. Andrade, et al. 2021. "Safety and Immunogenicity of INO-4800 DNA Vaccine against SARS-CoV-2: A Preliminary Report of an Open-Label, Phase 1 Clinical Trial." EClinicalMedicine 31 (January): 100689.

50 Tebas, Pablo, Kimberly A. Kraynyak, Ami Patel, Joel N. Maslow, Matthew P. Morrow, Albert J. Sylvester, Dawson Knoblock, et al. 2019. "Intradermal SynCon® Ebola GP DNA Vaccine Is Temperature Stable and Safely Demonstrates Cellular and Humoral Immunogenicity Advantages in Healthy Volunteers." *The Journal of Infectious Diseases* 220 (3): 400–410.

51 Tebas, Pablo, Christine C. Roberts, Kar Muthumani, Emma L. Reuschel, Sagar B. Kudchodkar, Faraz I. Zaidi, Scott White, et al. 2021. "Safety and Immunogenicity of an Anti-Zika Virus DNA Vaccine." *The New England Journal of Medicine* 385 (12): e35.

52 Huang, Ji-Han, Qian-Min Su, Juan Yang, Ying-Hua Lv, Ying-Chun He, Jun-Chao Chen, Ling Xu, Kun Wang, and Qing-Shan Zheng. 2015. "Sample Sizes in Dosage Investigational Clinical Trials: A Systematic Evaluation." Drug Design, Development and Therapy 9 (January): 305–12.



This document is confidential.

# 12. Signature Page

**Study Title:** A Phase 1 open-label, active-controlled, randomized dose-finding study to evaluate safety, tolerability, and immunogenicity of intradermal and subcutaneous plasmid DNA based SARS-CoV-2 Omicron BA.2 vaccine Alveavax-v1.2 in primary Ad26.COV2.S vaccinated healthy individuals.

| | |
|---|---|
| Clinical Trial Manager | DocuSigned by Tobias Odendahl<br>Tobias Odendahl \| I approve this document<br>2022-11-23 \| 7:24:35 AM EST<br>D0C26EF3B7F44E9F99E597F88D435A05<br><br>Head of Clinical |
| Chief Medical Officer | DocuSigned by Maximilian Schons<br>Maximilian Schons \| I approve this document<br>2022-11-23 \| 8:59:00 AM EST<br>7B42018FD95D4D8285E06EB459AB93E2 |

**Sponsor Approval**

*I agree to comply with all requirements regarding the obligations of Sponsor and all other pertinent requirements of Declaration of Helsinki (Fortaleza, 2013) and ICH E6 (R2) the guidelines on Good Clinical Practice (GCP) and any other applicable regulatory requirements.*

DocuSigned by Maximilian Schons
Maximilian Schons | I approve this document
2022-11-23 | 8:59:07 AM EST
7B42018FD95D4D8285E06EB459AB93E2

AUTHORIZED SIGNATORY

**Declaration of Investigator**

*I, the undersigned, have read and understood this protocol and hereby agree to conduct the study in accordance with this protocol and to comply with all requirements regarding the obligations of Investigators and all other pertinent requirements of the ICH E6 (R2) 'Guidelines on Good Clinical Practice', Declaration of Helsinki and other applicable regulatory authorities. All documentation for this study that is supplied to me, and that has not been previously published, will be kept in the strictest confidence. This documentation includes this study protocol, Investigator's Brochure, Case Report Forms, and other scientific data. Copying, disclosing and publishing without written consent of the Sponsor is prohibited. The study will not be commenced without the prior written approval of a properly constituted Institutional Review Board or Independent Ethics Committee. No changes will be made to the study protocol without the prior written approval of the Sponsor and the IRB or IEC, except where necessary to eliminate an immediate hazard to the participants. I further agree to ensure that all associates assisting in the conduct of this study are well informed regarding their obligations and confirm to conduct this study under my direction at the following address:*

Name of the Investigator: Dr Veronique de Jager

Signed and dated:

# 13. Appendices

1. Contraception
2. Blood collection
3. Toxicity Grading Scale
4. Composition of investigational product

## Appendix 1: Contraception

1. Females of childbearing potential should use highly effective methods of contraception from the time of screening until Day 84. Conception whilst on treatment must be avoided. Acceptable methods of contraception include the following:
    a. Combined (estrogen- and progesterone-containing) hormonal contraception associated with inhibition of ovulation:
        i. Oral
        ii. Intravaginal
        iii. Transdermal
    b. Progesterone-only hormonal contraception associated with inhibition of ovulation
        i. Oral
        ii. Injectable
        iii. Implantable
    c. Intrauterine device
    d. Intrauterine hormone-releasing system
    e. Bilateral tubal occlusion
    f. Vasectomized partner
    g. Sexual abstinence
2. Male participants who are considered sexually fertile must agree to use a barrier method of contraception during sexual activity with a female of childbearing potential from the time of vaccination until at least 84 days after the vaccination. Patients should refrain from donating sperm from the start of dosing and for at least 84 days after the vaccination.

## Appendix 2: Blood collection

At each on-site visit, blood will be collected as per Schedule of Activities (Table 1). Blood for serology tests, hematology and chemistry, and for the measurement of humoral and cell-mediated immune responses, will be collected as follows (Table 7):

The vaccination time and sampling times should be recorded accurately in the appropriate section of the eCRF.

DocuSign Envelope ID: 2B72DF38-444D-4144-86F5-185A92D63B30

The total blood volume to be taken from each participant over the study period is about 120 mL, and will be as follows:

Table 7: Blood Collection at Study Visit

| **Study Visits** | **Type of analysis** | **Approximate blood volume** |
|---|---|---|
| Visit -1<br>Screening Visit | Hematology, chemistry<br>Serology (HIV, HbsAg, and HCV) | 7 mL<br>5 mL |
| Visit 1<br>Check-in | Hematology and chemistry*<br>Humoral immunology<br>Cellular immunology | 7 mL<br>5 mL<br>30 mL |
| Visit 3<br>Follow-up: Day 7 | Hematology and chemistry<br>Humoral immunology | 7 mL<br>5 mL |
| Visit 4<br>Follow-up: Day 14 | Humoral immunology | 5 mL |
| Visit 5<br>Follow-up: Day 28 | Humoral immunology<br>Cellular immunology | 5 mL<br>30 mL |
| Visit 6<br>Follow-up: Day 84 | Humoral immunology | 5 mL |
| Visit 7<br>End of study: Day 168 | Humoral immunology | 5 mL |

## Appendix 3: Toxicity Grading Scale

Adapted from the FDA's published Guidance Toxicity Grading Scale (2007).

Table 8: Toxicity Grading Scale

| **Local Reaction to Injectable Product** | **Mild (Grade 1)** | **Moderate (Grade 2)** | **Severe (Grade 3)** | **Potentially Life Threatening (Grade 4)** |
|---|---|---|---|---|


This document is confidential.

DocuSign Envelope ID: 2B72DF38-444D-4144-86F5-185A92D63B30

| | | | | |
|---|---|---|---|---|
| Pain | Does not interfere with activity | Repeated use of non-narcotic pain reliever for > 24 hours, or interferes with activity | Any use of narcotic pain reliever, or prevents daily activity | Emergency room (ER) visit or hospitalization |
| Tenderness | Mild discomfort to touch | Discomfort with movement | Significant discomfort at rest | ER visit or hospitalization |
| Erythema/Redness* | 2.5 – 5 cm | 5.1 – 10 cm | > 10 cm | Necrosis or exfoliative dermatitis |
| Induration/Swelling** | 2.5 – 5 cm and does not interfere with activity | 5.1 – 10 cm or interferes with activity | > 10 cm or prevents daily activity | Necrosis |

* In addition to grading the measured local reaction at the greatest single diameter, the measurement should be recorded as a continuous variable.
** Induration/swelling should be evaluated and graded using the functional scale as well as the actual measurement.

| **Vital Signs *** | **Mild (Grade 1)** | **Moderate (Grade 2)** | **Severe (Grade 3)** | **Potentially Life Threatening (Grade 4)** |
|---|---|---|---|---|
| Fever (°C) **<br>(°F) ** | 38.0 – 38.4<br>100.4 – 101.1 | 38.5 – 38.9<br>101.2 – 102.0 | 39.0 – 40<br>102.1 – 104 | > 40<br>> 104 |
| Tachycardia - beats per minute | 101 – 115 | 116 – 130 | > 130 | ER visit or hospitalization for arrhythmia |
| Bradycardia - beats per minute*** | 50 – 54 | 45 – 49 | < 45 | ER visit or hospitalization for arrhythmia |

| | | | | |
|---|---|---|---|---|
| Hypertension (systolic) - mmHg | 141 – 150 | 151 – 155 | > 155 | ER visit or hospitalization for malignant hypertension |
| Hypertension (diastolic) - mmHg | 91 – 95 | 96 – 100 | > 100 | ER visit or hospitalization for malignant hypertension |
| Hypotension (systolic) – mm Hg | 85 – 89 | 80 – 84 | < 80 | ER visit or hospitalization for hypotensive shock |
| Respiratory Rate – breaths per minute | 17 – 20 | 21 – 25 | > 25 | Intubation |

* Participant should be at rest for all vital sign measurements.
** Oral temperature; no recent hot or cold beverages or smoking.
*** When the resting heart rate is between 60 and 100 beats per minute. Use clinical judgment when characterizing bradycardia among some healthy participant populations; for example, conditioned athletes.

| **Systemic (General)** | **Mild (Grade 1)** | **Moderate (Grade 2)** | **Severe (Grade 3)** | **Potentially Life Threatening (Grade 4)** |
|---|---|---|---|---|
| Nausea/vomiting | No interference with activity or 1 – 2 episodes/24 hours | Some interference with activity or > 2 episodes/24 hours | Prevents daily activity, requires outpatient IV hydration | ER visit or hospitalization for hypotensive shock |

| | | | | |
|---|---|---|---|---|
| Diarrhea | 2 – 3 loose stools or < 400 g/24 hours | 4 – 5 stools or 400 – 800 g/24 hours | 6 or more watery stools or > 800 g/24 hours or requires outpatient IV hydration | ER visit or hospitalization |
| Headache | No interference with activity | Repeated use of non-narcotic pain reliever > 24 hours or some interference with activity | Significant; any use of narcotic pain reliever or prevents daily activity | ER visit or hospitalization |
| Fatigue | No interference with activity | Some interference with activity | Significant; prevents daily activity | ER visit or hospitalization |
| Myalgia | No interference with activity | Some interference with activity | Significant; prevents daily activity | ER visit or hospitalization |

| **Systemic Illness** | **Mild (Grade 1)** | **Moderate (Grade 2)** | **Severe (Grade 3)** | **Potentially Life Threatening (Grade 4)** |
|---|---|---|---|---|
| Illness or clinical adverse event (as defined according to applicable regulations) | No interference with activity | Some interference with activity not requiring medical intervention | Prevents daily activity and requires medical intervention | ER visit or hospitalization |

| **Serum *** | **Mild (Grade 1)** | **Moderate (Grade 2)** | **Severe (Grade 3)** | **Potentially Life Threatening (Grade 4)**** |
|---|---|---|---|---|
| Sodium – hyponatremia mEq/L | 132 – 134 | 130 – 131 | 125 – 129 | < 125 |
| Sodium – hypernatremia mEq/L | 144 – 145 | 146 – 147 | 148 – 150 | > 150 |
| Potassium – hyperkalemia mEq/L | 5.1 – 5.2 | 5.3 – 5.4 | 5.5 – 5.6 | > 5.6 |
| Potassium – hypokalemia mEq/L | 3.5 – 3.6 | 3.3 – 3.4 | 3.1 – 3.2 | < 3.1 |
| Glucose – hypoglycemia mg/dL | 65 – 69 | 55 – 64 | 45 – 54 | < 45 |
| Glucose – hyperglycemia<br>fasting – mg/dL<br>random – mg/dL | 100 – 110<br>110 – 125 | 111 – 125<br>126 – 200 | >125<br>>200 | Insulin requirements or hyperosmolar coma |
| Blood urea nitrogen BUN mg/dL | 23 – 26 | 27 – 31 | > 31 | Requires dialysis |
| Creatinine – mg/dL | 1.5 – 1.7 | 1.8 – 2.0 | 2.1 – 2.5 | > 2.5 or requires dialysis |
| Calcium – hypocalcemia mg/dL | 8.0 – 8.4 | 7.5 – 7.9 | 7.0 – 7.4 | < 7.0 |

| | | | | |
|---|---|---|---|---|
| Calcium – hypercalcemia mg/dL | 10.5 – 11.0 | 11.1 – 11.5 | 11.6 – 12.0 | > 12.0 |
| Magnesium – hypomagnesemia mg/dL | 1.3 – 1.5 | 1.1 – 1.2 | 0.9 – 1.0 | < 0.9 |
| Phosphorous – hypophosphatemia mg/dL | 2.3 – 2.5 | 2.0 – 2.2 | 1.6 – 1.9 | < 1.6 |
| CPK – mg/dL | 1.25 – 1.5 x ULN*** | 1.6 – 3.0 x ULN | 3.1 –10 x ULN | > 10 x ULN |
| Albumin – hypoalbuminemia g/dL | 2.8 – 3.1 | 2.5 – 2.7 | < 2.5 | -- |
| Total Protein – hypoproteinemia g/dL | 5.5 – 6.0 | 5.0 – 5.4 | < 5.0 | -- |
| Alkaline phosphatase – increase by factor | 1.1 – 2.0 x ULN | 2.1 – 3.0 x ULN | 3.1 – 10 x ULN | > 10 x ULN |
| Liver Function Tests – ALT, AST increase by factor | 1.1 – 2.5 x ULN | 2.6 – 5.0 x ULN | 5.1 – 10 x ULN | > 10 x ULN |
| Bilirubin – when accompanied by any increase in Liver Function Test increase by factor | 1.1 – 1.25 x ULN | 1.26 – 1.5 x ULN | 1.51 – 1.75 x ULN | > 1.75 x ULN |


This document is confidential.

DocuSign Envelope ID: 2B72DF38-444D-4144-86F5-185A92D63B30

| | | | | |
|---|---|---|---|---|
| Bilirubin – when Liver Function Test is normal; increase by factor | 1.1 – 1.5 x ULN | 1.6 – 2.0 x ULN | 2.0 – 3.0 x ULN | > 3.0 x ULN |
| Cholesterol | 201 – 210 | 211 – 225 | > 226 | --- |
| Pancreatic enzymes – amylase, lipase | 1.1 – 1.5 x ULN | 1.6 – 2.0 x ULN | 2.1 – 5.0 x ULN | > 5.0 x ULN |

* The laboratory values provided in the tables serve as guidelines and are dependent upon institutional normal parameters. Institutional normal reference ranges should be provided to demonstrate that they are appropriate.
** The clinical signs or symptoms associated with laboratory abnormalities might result in characterization of the laboratory abnormalities as potentially life threatening (Grade 4). For example, a low sodium value that falls within a Grade 3 parameter (125-129 mE/L) should be recorded as a Grade 4 hyponatremia event if the participant had a new seizure associated with the low sodium value.
***ULN: upper limit of the normal range.

| **Hematology *** | **Mild (Grade 1)** | **Moderate (Grade 2)** | **Severe (Grade 3)** | **Potentially Life Threatening (Grade 4)** |
|---|---|---|---|---|
| Hemoglobin (female) – g/dL | 11.0 – 12.0 | 9.5 – 10.9 | 8.0 – 9.4 | < 8.0 |
| Hemoglobin (female) change from baseline value – g/dL | Any decrease – 1.5 | 1.6 – 2.0 | 2.1 – 5.0 | > 5.0 |
| Hemoglobin (male) – gm/dL | 12.5 – 13.5 | 10.5 – 12.4 | 8.5 – 10.4 | < 8.5 |
| Hemoglobin (male) change from baseline value – g/dL | Any decrease – 1.5 | 1.6 – 2.0 | 2.1 – 5.0 | > 5.0 |


This document is confidential.

| | | | | |
|---|---|---|---|---|
| WBC increase – cells/mm$^3$ | 10,800 – 15,000 | 15,001 – 20,000 | 20,001 – 25, 000 | > 25,000 |
| WBC decrease – cells/mm$^3$ | 2,500 – 3,500 | 1,500 – 2,499 | 1,000 – 1,499 | < 1,000 |
| Lymphocyte decrease – cells/mm$^3$ | 750 – 1,000 | 500 – 749 | 250 – 499 | < 250 |
| Neutrophil decrease – cells/mm$^3$ | 1,500 – 2,000 | 1,000 – 1,499 | 500 – 999 | < 500 |
| Eosinophils – cells/mm$^3$ | 650 – 1500 | 1501 - 5000 | > 5000 | Hypereosinophilic |
| Platelet decrease – cells/mm$^3$ | 125,000 – 140,000 | 100,000 – 124,000 | 25,000 – 99,000 | < 25,000 |
| PT (prothrombin time) – increase by factor | 1.0 – 1.10 x ULN** | 1.11 – 1.20 x ULN | 1.21 – 1.25 x ULN | > 1.25 ULN |
| PTT (partial thromboplastin time) – increase by factor | 1.0 – 1.2 x ULN | 1.21 – 1.4 x ULN | 1.41 – 1.5 x ULN | > 1.5 x ULN |
| Fibrinogen increase – mg/dL | 400 – 500 | 501 – 600 | > 600 | -- |
| Fibrinogen decrease – mg/dL | 150 – 200 | 125 – 149 | 100 – 124 | < 100 or associated with gross bleeding or disseminated intravascular coagulation (DIC) |


This document is confidential.

* The laboratory values provided in the tables serve as guidelines and are dependent upon institutional normal parameters. Institutional normal reference ranges should be provided to demonstrate that they are appropriate.
** "ULN: upper limit of the normal range.


This document is confidential.

# APPENDIX 4: COMPOSITION OF INVESTIGATIONAL PRODUCT

Table 9: Composition of Investigational Product

| Chemical name | Quality Standard | Concentration | Function |
|---|---|---|---|
| Drug substance | GMP (VGXI, TX, US) | 5 ± 0.5 mg/mL | Active |
| Potassium chloride, KCl, 7447-40-7 | Multi-compendial | 2.7 mM | Buffer |
| Sodium chloride, NaCl, 7647-14-5 | Multi-compendial | 137 mM | Buffer |
| Potassium phosphate, monobasic, $KH_2PO_4$, 7778-77-0 | Multi-compendial | 1.4 mM | Buffer |
| Sodium phosphate, dibasic, $Na_2HPO_4$, 7558-79-4 | Multi-compendial | 8.1 mM | Buffer |
| Sterile water for injection (sWFI), 7732-18-5 | USP | N/A | Solvent |


This document is confidential.

# Appendix 5: Protocol amendments

| Date | Protocol version | Section | Reason | Old text | New text |
|---|---|---|---|---|---|
| 13-May-22 | v2.0 18March2022 | Study title | Drop unvaccinated arm (2.a) | A Phase 1 open-label, active-controlled, randomized dose-finding study to evaluate safety, tolerability, and immunogenicity of intradermal and subcutaneous application of the plasmid DNA SARS-CoV-2 Omicron BA.2 vaccine Alveavax-v1.2 in primary Ad26.COV2.S vaccinated or SARS-CoV-2 recovered unvaccinated healthy individuals. | A Phase 1 open-label, active-controlled, randomized dose-finding study to evaluate safety, tolerability, and immunogenicity of intradermal and subcutaneous application of the plasmid DNA SARS-CoV-2 Omicron BA.2 vaccine Alveavax-v1.2 in primary Ad26.COV2.S vaccinated healthy individuals. |
| 13-May-22 | v2.0 18March2022 | Synopsis: Target Sample Size | Drop unvaccinated arm (2.a) | 150 | 130 |
| 13-May-22 | v2.0 18March2022 | Synopsis: Characteristics and Number of Participants | Drop unvaccinated arm (2.a) | 150 healthy individuals (between 18 and 65 years of age, both inclusive), previously having received a primary Ad26.COV2.S vaccination series against SARS-CoV-2 or being unvaccinated and recovered from SARS-CoV-2, satisfying all the eligibility criteria will be eligible to participate in the study. Screening for eligible participants will be performed within 28 days of vaccination in the study. | 130 healthy individuals (between 18 and 65 years of age, both inclusive), previously having received a primary Ad26.COV2.S vaccination series against SARS-CoV-2, satisfying all the eligibility criteria will be eligible to participate in the study. Screening for eligible participants will be performed within 28 days of vaccination in the study. |


This document is confidential.

| | | | | | |
|---|---|---|---|---|---|
| 13-May-22 | v2.0 18March2022 | 1.2 Schema | Drop unvaccinated arm (2.a) | Note: The enrollment will start with the first ten participants randomized into the low dose (1.a) arm, the control arm (1.e), subsequently at least 24 hours apart five participants to the vaccinated standard dose (1.b) arm, followed by a review of 24h safety data by the independent medical monitor. Then, the remaining participants assigned to the low and standard dose cohorts, in addition to those assigned to the control arm (1.e) and unvaccinated standard dose arm (2.a) will be recruited. In parallel, the first five participants of the high dose (1.c) arm will be enrolled. Further recruitment and the SC injection arm (1.d) start as soon as the independent medical monitor has reviewed 24h safety data of the high dose arm. | *Removed arm 2.a from image.*<br><br>Note: The enrollment will start with the first ten participants randomized into the low dose (1.a) arm, the control arm (1.e), subsequently at least 24 hours apart five participants to the vaccinated standard dose (1.b) arm, followed by a review of 24h safety data by the independent medical monitor. Then, the remaining participants assigned to the low and standard dose cohorts, in addition to those assigned to the control arm (1.e) will be recruited. In parallel, the first five participants of the high dose (1.c) arm will be enrolled. Further recruitment and the SC injection arm (1.d) start as soon as the independent medical monitor has reviewed 24h safety data of the high dose arm. |
| 13-May-22 | v2.0 18March2022 | 2.1 Study Rationale | Drop unvaccinated arm (2.a) | The objective of this first in human Phase 1 dose-finding trial is to describe the safety, tolerability, and immunogenicity of Alveavax-v1.2 in primary vaccinated as well as recovered individuals.<br>[...]<br>In a first in human dose-finding study design we will investigate the immune responses of three dose intradermal (ID) levels in vaccinated individuals, and one ID dose in recovered individuals. | The objective of this first in human Phase 1 dose-finding trial is to describe the safety, tolerability, and immunogenicity of Alveavax-v1.2 in primary vaccinated individuals.<br>[...]<br>In a first in human dose-finding study design we will investigate the immune responses of three dose intradermal (ID) levels in vaccinated individuals. |


This document is confidential.

| | | | | | |
|---|---|---|---|---|---|
| 13-May-22 | v2.0 18March2022 | 4.1 Overall design | Drop unvaccinated arm (2.a) | This is a first in human, open-label, active-controlled, randomized dose-finding study to evaluate safety, tolerability, and immunogenicity of ID and SC application of the plasmid DNA SARS-CoV-2 Omicron BA.2 vaccine Alveavax-v1.2 in primary Ad26.COV2.S vaccinated or SARS-CoV-2 recovered unvaccinated healthy individuals.<br><br>Primary Ad26.COV2.S vaccinated participants will be randomized into one of 5 treatment arms to receive Alveavax-v1.2 or a Ad26.COV2.S control booster vaccine. Unvaccinated participants will receive a predetermined dose of Alveavax-v1.2. | This is a first in human, open-label, active-controlled, randomized dose-finding study to evaluate safety, tolerability, and immunogenicity of ID and SC application of the plasmid DNA SARS-CoV-2 Omicron BA.2 vaccine Alveavax-v1.2 in primary Ad26.COV2.S vaccinated healthy individuals.<br><br>Primary Ad26.COV2.S vaccinated participants will be randomized into one of 5 treatment arms to receive Alveavax-v1.2 or a Ad26.COV2.S control booster vaccine. |
| 13-May-22 | v2.0 18March2022 | 4.1 Overall design | Drop unvaccinated arm (2.a) | A total of 150 male and female participants aged between 18 and 65 years who satisfy the inclusion and exclusion criteria are planned to be enrolled in six groups and with vaccine administered according to Table 3:<br><br>Individuals with a primary vaccination will get either:<br>Low dose: 0.5 mg Alveavax-v1.2 in one ID injection<br>Standard dose: 2 mg Alveavax-v1.2 in one ID injection<br>High dose: 8mg Alveavax-v1.2 in four ID injections*<br>SC injection: 8mg Alveavax-v1.2 in one SC injection<br>Control: Janssen Ad26.COV2.S in one IM injection<br>Unvaccinated individuals will get Standard dose: 2 mg | A total of 130 male and female participants aged between 18 and 65 years who satisfy the inclusion and exclusion criteria are planned to be enrolled in five groups and with vaccine administered according to Table 3:<br><br>Individuals with a primary vaccination will get either:<br>Low dose: 0.5 mg Alveavax-v1.2 in one ID injection<br>Standard dose: 2 mg Alveavax-v1.2 in one ID injection<br>High dose: 8mg Alveavax-v1.2 in four ID injections*<br>SC injection: 8mg Alveavax-v1.2 in one SC injection<br>Control: Janssen Ad26.COV2.S in one IM injection |



This document is confidential.

| | | | | | |
|---|---|---|---|---|---|
| | | | | Alveavax-v1.2 in one ID injection | |
| 13-May-22 | v2.0 18March2022 | 4.1 Overall design | Drop unvaccinated arm (2.a) | The enrollment will start with the first five participants in the low dose (1.a) arm and the vaccinated standard dose (1.b) arm, followed by a review of 24h safety data by the independent medical monitor. Then, the remaining participants from the low and standard dose arms, as well as the control arm (1.e) and unvaccinated standard dose arm (2.a) will be recruited. In parallel, the first five participants of the high dose (1.c) arm are enrolled. Further recruitment and the SC injection arm (1.d) start as soon as the independent medical monitor has reviewed 24h safety data of the high dose arm (see schema in 1.2). | The enrollment will start with the first five participants in the low dose (1.a) arm and the vaccinated standard dose (1.b) arm, followed by a review of 24h safety data by the independent medical monitor. Then, the remaining participants from the low and standard dose arms, as well as the control arm (1.e) will be recruited. In parallel, the first five participants of the high dose (1.c) arm are enrolled. Further recruitment and the SC injection arm (1.d) start as soon as the independent medical monitor has reviewed 24h safety data of the high dose arm (see schema in 1.2). |
| 13-May-22 | v2.0 18March2022 | 5. Study Population | Drop unvaccinated arm (2.a) | Healthy adult individuals, previously having received a primary Ad26.COV2.S vaccination series against SARS-CoV-2 or being unvaccinated and recovered from SARS-CoV-2, satisfying all the eligibility criteria will be eligible to participate in the study. Screening for eligible participants will be performed within 28 days of vaccination in the study. | Healthy adult individuals, previously having received a primary Ad26.COV2.S vaccination series against SARS-CoV-2, satisfying all the eligibility criteria will be eligible to participate in the study. Screening for eligible participants will be performed within 28 days of vaccination in the study. |
| 13-May-22 | v2.0 18March2022 | 5.1 Number of Participants Planned | Drop unvaccinated arm (2.a) | A total of 150 healthy participants will be enrolled in six groups. | A total of 130 healthy participants will be enrolled in five groups. |
| 13-May-22 | v2.0 18March2022 | 5.2 Inclusion Criteria | Drop unvaccinated arm (2.a) | Participants who received a primary Janssen Ad26.COV2.S vaccine ≥ 60 days prior to receiving the study vaccine and/or are recovered from SARS-CoV-2, defined as a history of a positive SARS-CoV-2 test | Participants who received a primary Janssen Ad26.COV2.S vaccine ≥ 60 days prior to receiving the study vaccine (Day 1) in this study. |


This document is confidential.

| | | | | | |
|---|---|---|---|---|---|
| | | | | (e.g. PCR, rapid antigen test, etc.) ≥ 60 days prior to receiving the study vaccination (Day 1) in this study. | |
| 13-May-22 | v2.0 18March2022 | 6.2 Dosage and Treatment Schedule | Drop unvaccinated arm (2.a) | | Table 3: Dosing Schema Remove Group 2 |
| 13-May-22 | v2.0 18March2022 | 6.5.1 Procedures for Randomization and Stratification | Drop unvaccinated arm (2.a) | Group 2 will not be randomized as it contains only a single intervention. | [*removed*] |
| 13-May-22 | v2.0 18March2022 | 9.2 Sample size determination | Drop unvaccinated arm (2.a) | The safety and immune response of the standard dose in vaccinated individuals will be compared with groups of 20 participants each in the low dose and high dose arm in vaccinated individuals as well as the standard dose in unvaccinated, recovered individuals. | The safety and immune response of the standard dose in vaccinated individuals will be compared with groups of 20 participants each in the low dose and high dose arm in vaccinated individuals. |
| 13-May-22 | v2.0 18March2022 | 12. Signature Page | Drop unvaccinated arm (2.a) | | [*removed date*] |
| 13-May-22 | v2.0 18March2022 | Appendix 5: Protocol amendments | Drop unvaccinated arm (2.a) | | [*Added appendix*] |
| 13-May-22 | v2.0 18March2022 | Previous Version | Drop unvaccinated arm (2.a) | [*none*] | 2.0 |

| | | | | | |
|---|---|---|---|---|---|
| 13-May-22 | v2.0 18March2022 | Protocol Version | Drop unvaccinated arm (2.a) | 2.0 | 3.0 |
| 25-Aug-22 | v3.0 16May2022 | **1.3 Schedule of Activities (SoA, Table 1)** | Updated schedule of activities | Urine pregnancy Test done at Check in | Urine pregnancy test will be performed for female participants of childbearing potential on screening, pre-dose, on day 28, and on day 84 |
| 25-Aug-22 | v3.0 16May2022 | 8.5.4 Evaluating Adverse Events | Change safety email address | safety.alvea@bioclinica.com | safety.alvea-vax-p00001@alveavax.com |
| 25-Aug-22 | v3.0 16May2022 | Protocol Version | Change safety email address | 3.0 | 4.0 |
| 16-Nov-22 | v4.0 13-Sep-2022 | Protocol Version | End study after 6 months | 2022-09-13 7pm CEST | 2022-11-16 9am CEST |
| 16-Nov-22 | v4.0 13-Sep-2022 | Protocol Version | End study after 6 months | 4.0 | 5.0 |
| 16-Nov-22 | v4.0 13-Sep-2022 | Protocol Version | End study after 6 months | 3.0 | 4.0 |

| | | | | | |
|---|---|---|---|---|---|
| 16-Nov-22 | v4.0<br>13-Sep-2022 | Protocol Version | End study after 6 months | April 2022 (planned) | April 2022 |
| 16-Nov-22 | v4.0<br>13-Sep-2022 | Protocol Version | End study after 6 months | Recruiting | Enrolment completed |
| 16-Nov-22 | v4.0<br>13-Sep-2022 | Protocol Version | End study after 6 months | 4.0 | 5.0 |
| 16-Nov-22 | v4.0<br>13-Sep-2022 | 1.1 Synopsis | End study after 6 months | 16 months | 10 months |
| 16-Nov-22 | v4.0<br>13-Sep-2022 | 1.1 Synopsis | End study after 6 months | Efficacy assessments will include the following:<br>Clinical efficacy measured using the WHO clinical progression scale for COVID-19<br>Percentage of participants with BA.2 neutralization IC50>100 IU/mL<br>Percentage of participants with BA.2 neutralization IC50>200 IU/mL<br>Percentage of participants with BA.2 neutralization IC50>400 IU/mL<br>Percentage of participants with BA.2 neutralization IC50>800 IU/mL<br>Absolute number and fraction of ID injections which generated a ≥ 1 mm and ≥ 7 mm in diameter clearly | Efficacy assessments will include the following:<br>Clinical efficacy measured using the WHO clinical progression scale for COVID-19<br>Absolute number and fraction of ID injections which generated a ≥ 1 mm and ≥ 7 mm in diameter clearly demarcated bleb, clearly visible for at least 20 seconds, for 0.5 mg and 2 mg Alveavax-v1.2 respectively<br>Percentage of participants with neutralization IC50>64 IU/mL against the respective strain<br>Percentage of participants with neutralization IC50>128 IU/mL against the respective strain<br>Serologic change in GMT and GMFR between baseline and other blood samples for |

| | | | | | |
|---|---|---|---|---|---|
| | | | | demarcated bleb, clearly visible for at least 20 seconds, for 0.5 mg and 2 mg Alveavax-v1.2 respectively<br>Percentage of participants with neutralization IC50>64 IU/mL against the respective strain<br>Percentage of participants with neutralization IC50>128 IU/mL against the respective strain<br>Serologic change in GMT and GMFR between baseline and other blood samples for anti-nucleocapsid protein (N) IgG antibodies | anti-nucleocapsid protein (N) IgG antibodies |
| 16-Nov-22 | v4.0<br>13-Sep-2022 | 1.1 Synopsis | End study after 6 months | Description of GMT, change in GMT, and GMFR of serum anti-SARS-CoV-2 neutralizing antibody titers and the change in GMT and GFR of anti-spike protein (S) IgG antibody and anti-N protein (N) IgG antibody at Day 7, Day 14, Day 28, Day 84, and Day 168 and Day 365.<br><br>Tabulation of the number and percentage of participants with neutralization IC50>100 IU/ml, IC50>200 IU/mL, IC50>400 IU/mL, and IC50>800 IU/mL against the Omicron variant with corresponding 95% CI. | Description of GMT, change in GMT, and GMFR of serum anti-spike protein (S) IgG antibody titers and the change in GMT and GFR of anti-N protein (N) IgG antibody at Day 28. |
| 16-Nov-22 | v4.0<br>13-Sep-2022 | 1.1 Synopsis | End study after 6 months | The number and percentage of participants being found to be SARS-CoV-2 positive by external testing (score 1-10), number and percentage of participants with ambulatory mild disease (score 1-3), number and percentage of participants hospitalized with moderate disease (score 4-5), | The number and percentage of participants being found to be SARS-CoV-2 positive by external testing (score 1-10), number and percentage of participants with ambulatory mild disease (score 1-3), number and percentage of participants hospitalized with moderate disease (score 4-5), number and percentage of participants |

| | | | | | |
|---|---|---|---|---|---|
| | | | | number and percentage of participants hospitalized with severe disease (score 6-9), number and percentage of participants who died (score 10) while being SARS-CoV-2 positive with the exact 95% CI will also be presented separately for each day: Day 7, Day 14, Day 28, Day 84, Day 168, Day 365. | hospitalized with severe disease (score 6-9), number and percentage of participants who died (score 10) while being SARS-CoV-2 positive with the exact 95% CI will also be presented separately for each day: Day 7, Day 14, Day 28, Day 84, Day 168. |
| 16-Nov-22 | v4.0<br>13-Sep-2022 | 1.1 Synopsis | End study after 6 months | Characterization of humoral antibody immune response of booster vaccinations against Ancestral and Variant (Alpha, Beta, Delta, BA.1, and any newly discovered VOC) SARS-CoV-2 Strains measured using GMT of anti-SARS-CoV-2 neutralizing antibody at each timepoint (Pre-vaccination, Day 7, Day 14, Day 28, Day 84, and Day 168 and Day 365) will be made using the approach described for the primary endpoint analyses. | Characterization of humoral antibody immune response of booster vaccinations against Ancestral and Variant (Alpha, Beta, Delta, BA.1, and any newly discovered VOC) SARS-CoV-2 Strains measured using GMT of anti-SARS-CoV-2 neutralizing antibody at each timepoint (Pre-vaccination, Day 7, Day 14, Day 28, Day 84, and Day 168) will be made using the approach described for the primary endpoint analyses. |
| 16-Nov-22 | v4.0<br>13-Sep-2022 | 1.3 Schedule of Activities (assessment) | End study after 6 months | D14 ±2d,<br>D28 ±2d,<br>D84 ±7d,<br>D168 ±14d | D14 ±2d,<br>D28 ±2d,<br>D84 ±7d |
| 16-Nov-22 | v4.0<br>13-Sep-2022 | 1.3 Schedule of Activities (assessment) | End study after 6 months | D365 ±14d<br>(EOS) | D168 ±14d<br>(EOS) |



This document is confidential.

| | | | | | |
|---|---|---|---|---|---|
| 16-Nov-22 | v4.0<br>13-Sep-2022 | 1.3 Schedule of Activities (visits) | End study after 6 months | V4, 5, 6, 7<br>V8 | V4, 5, 6<br>V7 |
| 16-Nov-22 | v4.0<br>13-Sep-2022 | 3. Objectives and Endpoints | End study after 6 months | The objective of the study is to assess the tolerability, safety, and immunogenicity of different doses and routes of administration of the Alveavax-v1.2 vaccine in healthy individuals. | The objective of the study is to assess the tolerability, safety, and immunogenicity of different doses and routes of administration of the Alveavax-v1.2 vaccine in healthy individuals.<br>Exploratory endpoints will or will not be tested based on findings from secondary endpoints and sponsor's determination accordingly. |
| 16-Nov-22 | v4.0<br>13-Sep-2022 | 3. Objectives and Endpoints | End study after 6 months | Day 1, 7, 28, after 3, and 6, and 12 months | Day 1, 7, 28, after 3, and 6 months |
| 16-Nov-22 | v4.0<br>13-Sep-2022 | 3. Objectives and Endpoints (Humoral immune response) | End study after 6 months | **Humoral immune response**<br>Characterization of humoral immune response of booster vaccinations against SARS-CoV-2 BA.2/Omicron measured using the following:<br>1. Change in geometric mean titer (GMT) of serum anti-SARS-CoV-2 BA.2 neutralizing antibody titers<br>2.GMT of serum anti-SARS-CoV-2 BA.2 neutralizing antibody titers<br>3.Geometric mean fold rise (GMFR) of serum anti-SARS-CoV-2 BA.2 neutralizing antibody<br>4. Four-fold increase rate of anti-SARS-CoV-2 BA.2 neutralizing antibody<br>5. Change in GMT of anti-spike | **Humoral immune response**<br>Characterization of humoral immune response of booster vaccinations against SARS-CoV-2 BA.2/Omicron measured using the following:<br>1. Change in GMT of anti-spike protein (S) immunoglobulin G (IgG) antibody<br>2. GMT of anti-spike protein (S) IgG antibody<br>3. GMFR of anti-spike protein (S) IgG |

| | | | | | |
|---|---|---|---|---|---|
| | | | | protein (S) immunoglobulin G (IgG) antibody<br>6. GMT of anti-spike protein (S) IgG antibody<br>7.GMFR of anti-spike protein (S) IgG<br>8. Percentage of participants with neutralization IC50≥100 IU/mL<br>9. Percentage of participants with neutralization IC50>200 IU/mL<br>10. Percentage of participants with neutralization IC50>400 IU/mL<br>11. Percentage of participants with neutralization IC50>800 IU/mL | |
| 16-Nov-22 | v4.0<br>13-Sep-2022 | 3. Objectives and Endpoints (Humoral immune response) | End study after 6 months | Humoral immune response Baseline (pre-vaccination; except for Point 1), Day 7,<br>Day 14,<br>Day 28,<br>Day 84,<br>Day 168,<br>Day 365 | Baseline (pre-vaccination; except for Point 1),<br>Day 28 |
| 16-Nov-22 | v4.0<br>13-Sep-2022 | 3. Objectives and Endpoints (Clinical efficacy) | End study after 6 months | Day 7,<br>Day 14,<br>Day 28,<br>Day 84,<br>Day 168,<br>Day 365 | Day 7,<br>Day 14,<br>Day 28,<br>Day 84,<br>Day 168 |
| 16-Nov-22 | v4.0<br>13-Sep-2022 | 3. Objectives and Endpoints (Cellular immune response) | End study after 6 months | Baseline (pre-vaccination),<br>Day 28,<br>Day 168 | Baseline (pre-vaccination),<br>Day 28 |


This document is confidential.

| | | | | | |
|---|---|---|---|---|---|
| 16-Nov-22 | v4.0<br>13-Sep-2022 | 3. Objectives and Endpoints (Cellular immune response) | End study after 6 months | Baseline (pre-vaccination; except for Point 1),<br>Day 7,<br>Day 14,<br>Day 28,<br>Day 84,<br>Day 168,<br>Day 365 | Baseline (pre-vaccination; except for Point 1),<br>Day 7,<br>Day 14,<br>Day 28,<br>Day 84,<br>Day 168 |
| 16-Nov-22 | v4.0<br>13-Sep-2022 | 3. Objectives and Endpoints (Cellular immune response) | End study after 6 months | Baseline (pre-vaccination; except for Point 1),<br>Day 7,<br>Day 14,<br>Day 28,<br>Day 84,<br>Day 168,<br>Day 365 | Baseline (pre-vaccination; except for Point 1),<br>Day 7,<br>Day 14,<br>Day 28,<br>Day 84,<br>Day 168 |
| 16-Nov-22 | v4.0<br>13-Sep-2022 | 3. Objectives and Endpoints (Cellular immune response) | End study after 6 months | Day 7,<br>Day 14,<br>Day 28,<br>Day 84,<br>Day 168,<br>Day 365 | Day 7,<br>Day 14,<br>Day 28,<br>Day 84,<br>Day 168 |
| 16-Nov-22 | v4.0<br>13-Sep-2022 | 3. Objectives and Endpoints (Cellular immune response) | End study after 6 months | Baseline, Day 7,<br>Day 14,<br>Day 28,<br>Day 84,<br>Day 168,<br>Day 365 | Baseline, Day 7,<br>Day 14,<br>Day 28,<br>Day 84,<br>Day 168 |
| 16-Nov-22 | v4.0<br>13-Sep-2022 | 4.2 Scientific Rationale for Study Design | End study after 6 months | The Alveavax-v1.2 vaccine is being developed to prevent severe cases of COVID-19, the disease resulting from SARS-CoV-2 infection. The study is designed to primarily evaluate the tolerability and safety of Alveavax-v1.2 for up to 612 months after the booster dose with Alveavax-v1.2. Additionally the | The Alveavax-v1.2 vaccine is being developed to prevent severe cases of COVID-19, the disease resulting from SARS-CoV-2 infection. The study is designed to primarily evaluate the tolerability and safety of Alveavax-v1.2 for up to 6 months after the booster dose with Alveavax-v1.2. Additionally the study will determine |

| | | | | | |
|---|---|---|---|---|---|
| | | | | study will determine immunogenicity endpoints and descriptive clinical efficacy against SARS-CoV-2 infection as secondary objectives. | immunogenicity endpoints and descriptive clinical efficacy against SARS-CoV-2 infection as secondary objectives. |
| 16-Nov-22 | v4.0<br>13-Sep-2022 | 8.2 Visit Schedules | End study after 6 months | **Day 14 (± 2 days), Day 28 (± 2 days), and Day 84 (± 7 days), and Day 168 (± 14 days)**<br>- Recording of adverse events<br>- Blood sample collection for immunogenicity and cellular response analysis<br>- Confirm with female participants of childbearing potential and male participants who have a female partner if they/their partner became pregnant since Day 1<br>- Recording of COVID-19 infections and vaccinations since Day 1<br>- NP swab sample for COVID-19 PCR on Day 14 and Day 28 | **Day 14 (± 2 days), Day 28 (± 2 days), and Day 84 (± 7 days)**<br>- Recording of adverse events<br>- Blood sample collection for immunogenicity and cellular response analysis<br>- Confirm with female participants of childbearing potential and male participants who have a female partner if they/their partner became pregnant since Day 1<br>- Recording of COVID-19 infections and vaccinations since Day 1<br>- NP swab sample for COVID-19 PCR on Day 14 and Day 28 |
| 16-Nov-22 | v4.0<br>13-Sep-2022 | 8.2 Visit Schedules | End study after 6 months | **Day 365 (± 14 days) (End-of-Study)**<br>- Abbreviated, symptom-directed physical examination<br>- Vital signs and weight<br>- Recording of adverse events<br>- Blood sample collection for immunogenicity and cellular response analysis<br>- Recording of COVID-19 infections and vaccinations since Day 1<br>- Confirm with female participants of childbearing potential and male participants who have a female partner if they/their partner became | **Day 168 (± 14 days) (End-of-Study)**<br>- Abbreviated, symptom-directed physical examination<br>- Vital signs and weight<br>- Recording of adverse events<br>- Blood sample collection for immunogenicity response analysis<br>- Recording of COVID-19 infections and vaccinations since Day 1<br>- Confirm with female participants of childbearing potential and male participants who have a female partner if they/their partner became pregnant since Day 1 of the |


This document is confidential.

| | | | | | |
|---|---|---|---|---|---|
| | | | | pregnant since Day 1 of the study. | study. |
| 16-Nov-22 | v4.0<br>13-Sep-2022 | 8.5 Adverse Events | End study after 6 months | **Treatment-Emergent Adverse Event (TEAE)**<br>A treatment-emergent adverse event (TEAE) is defined as any event at or after the time of exposure to study vaccine or any event already present that worsens in either intensity or frequency following exposure to the study vaccine, until the end of the participant's participation in the study (i.e., Visit 8, day 365 in Table 1, participant withdrawal, or participant lost to follow-up). | **Treatment-Emergent Adverse Event (TEAE)**<br>A treatment-emergent adverse event (TEAE) is defined as any event at or after the time of exposure to study vaccine or any event already present that worsens in either intensity or frequency following exposure to the study vaccine, until the end of the participant's participation in the study (i.e., Visit 7, day 168 in Table 1, participant withdrawal, or participant lost to follow-up). |
| 16-Nov-22 | v4.0<br>13-Sep-2022 | 8.5.2 Recording and Handling of Adverse Events | End study after 6 months | SAEs, AESIs, and AEs leading to study drug withdrawal should be collected starting from screening until the end of study (Day 365, Visit 8 in Table 1) and reported at each visit during the study. | SAEs, AESIs, and AEs leading to study drug withdrawal should be collected starting from screening until the end of study (Day 168, Visit 7 in Table 1) and reported at each visit during the study. |
| 16-Nov-22 | v4.0<br>13-Sep-2022 | 8.5.2 Recording and Handling of Adverse Events (Table 6) | End study after 6 months | **Duration of Collection***<br>7 days (Day 1 and 6 subsequent days) after the vaccination<br>Screening and 28 days (Visit 5) after the vaccination<br>during the whole study (Screening until Day 365)<br>during the whole study (Screening until Day 365)<br>during the whole study (Screening until Day 365)<br>After the vaccination (Day 1 until Day 365) | **Duration of Collection***<br>7 days (Day 1 and 6 subsequent days) after the vaccination<br>Screening and 28 days (Visit 5) after the vaccination<br>during the whole study (Screening until Day 168)<br>during the whole study (Screening until Day 168)<br>during the whole study (Screening until Day 168)<br>After the vaccination (Day 1 until Day 168) |


This document is confidential.

| | | | | | |
|---|---|---|---|---|---|
| 16-Nov-22 | v4.0<br>13-Sep-2022 | 8.5.5 Reporting Serious Adverse Events | End study after 6 months | **Discontinuation of the Study due to Adverse Events**<br>The reason for a participant being discontinued from the study will be recorded in the CRF. A discontinuation occurs when an enrolled participant ceases participation in the study, regardless of the circumstances, prior to the completion of the study. A discontinuation must be reported immediately to the Sponsor if it is due to a SAE. The final evaluation as required by the protocol (see Table 1 Visit 8, day 365) will be performed at the time of discontinuation if medically acceptable or as soon as possible after that. The Investigator will record the reason for study discontinuation and, if possible, provide or arrange for appropriate follow-up and document the cause of the participant's condition. | **Discontinuation of the Study due to Adverse Events**<br>The reason for a participant being discontinued from the study will be recorded in the CRF. A discontinuation occurs when an enrolled participant ceases participation in the study, regardless of the circumstances, prior to the completion of the study. A discontinuation must be reported immediately to the Sponsor if it is due to a SAE. The final evaluation as required by the protocol (see Table 1 Visit 7, day 168) will be performed at the time of discontinuation if medically acceptable or as soon as possible after that. The Investigator will record the reason for study discontinuation and, if possible, provide or arrange for appropriate follow-up and document the cause of the participant's condition. |
| 16-Nov-22 | v4.0<br>13-Sep-2022 | 9.4.3. Secondary Endpoints Analysis | End study after 6 months | Description of GMT, change in GMT, and GMFR of serum anti-SARS-CoV-2 neutralizing antibody titers and the change in GMT and GFR of anti-spike protein (S) IgG antibody and anti-N protein (N) IgG antibody at Day 7, Day 14, Day 28, Day 84, and Day 168 and Day 365.<br><br>Table showing the number and percentage of participants with neutralization IC50>100 IU/ml, IC50>200 IU/mL, IC50>400 IU/mL, and IC50>800 IU/mL against the Omicron variant | Description of GMT, change in GMT, and GMFR of anti-spike protein (S) IgG antibody and the change in GMT and GFR of anti-N protein (N) IgG antibody at Day 28. |


This document is confidential.

| | | | | | |
|---|---|---|---|---|---|
| | | | | with corresponding 95% CI. | |
| 16-Nov-22 | v4.0<br>13-Sep-2022 | 9.4.3. Secondary Endpoints Analysis | End study after 6 months | The number and percentage of participants being found to be SARS-CoV-2 positive by external testing (score 1-10), number and percentage of participants with ambulatory mild disease (score 1-3), number and percentage of participants hospitalized with moderate disease (score 4-5), number and percentage of participants hospitalized with severe disease (score 6-9), number and percentage of participants who died (score 10) while being SARS-CoV-2 positive with the exact 95% CI will also be presented separately for each day: Day 7, Day 14, Day 28, Day 84, Day 168, and Day 365. | The number and percentage of participants being found to be SARS-CoV-2 positive by external testing (score 1-10), number and percentage of participants with ambulatory mild disease (score 1-3), number and percentage of participants hospitalized with moderate disease (score 4-5), number and percentage of participants hospitalized with severe disease (score 6-9), number and percentage of participants who died (score 10) while being SARS-CoV-2 positive with the exact 95% CI will also be presented separately for each day: Day 7, Day 14, Day 28, Day 84, and Day 168. |
| 16-Nov-22 | v4.0<br>13-Sep-2022 | 9.4.6. Exploratory Analyses | End study after 6 months | Characterization of humoral antibody immune response of booster vaccinations against Ancestral and Variant (Alpha, Beta, Delta, BA.1, and any newly discovered VOC) SARS-CoV-2 Strains measured using GMT of anti-SARS-CoV-2 neutralizing antibody at each timepoint (Pre-vaccination, Day 7, Day 14, Day 28, Day 84, Day 168 and Day 365) will be made using the approach described for the primary endpoint analyses. | Characterization of humoral antibody immune response of booster vaccinations against Ancestral and Variant (Alpha, Beta, Delta, BA.1, and any newly discovered VOC) SARS-CoV-2 Strains measured using GMT of anti-SARS-CoV-2 neutralizing antibody at each timepoint (Pre-vaccination, Day 7, Day 14, Day 28, Day 84, and Day 168) will be made using the approach described for the primary endpoint analyses. |


This document is confidential.

| | | | | | |
|---|---|---|---|---|---|
| 16-Nov-22 | v4.0<br>13-Sep-2022 | Appendix 2: Blood collection | End study after 6 months | At each on-site visit, blood will be collected as per Schedule of Activities (Table 1). Blood for serology tests, hematology and chemistry, and for the measurement of humoral and cell-mediated immune responses, will be collected as follows (Table 7):<br><br>The vaccination time and sampling times should be recorded accurately in the appropriate section of the eCRF.<br><br>The total blood volume to be taken from each participant over the study period is about 150 mL, and will be as follows: | At each on-site visit, blood will be collected as per Schedule of Activities (Table 1). Blood for serology tests, hematology and chemistry, and for the measurement of humoral and cell-mediated immune responses, will be collected as follows (Table 7):<br><br>The vaccination time and sampling times should be recorded accurately in the appropriate section of the eCRF.<br><br>The total blood volume to be taken from each participant over the study period is about 120 mL, and will be as follows: |
| 16-Nov-22 | v4.0<br>13-Sep-2022 | Appendix 2: Blood collection (Table 7 Blood collection at Study Visit) | End study after 6 months | Visit 7<br>Follow-up: Day 168<br><br>Humoral immunology<br>Cellular immunology<br><br>5 mL<br>30 mL | [*removed row*] |
| 16-Nov-22 | v4.0<br>13-Sep-2022 | Appendix 2: Blood collection (Table 7 Blood collection at Study Visit) | End study after 6 months | Visit 8<br>End of study: Day 365<br><br>Humoral immunology<br><br>5 mL | Visit 7<br>End of study: Day 168<br><br>Humoral immunology<br><br>5 mL |


This document is confidential.
