# Supplementary material for: Safety and immunogenicity of DNA omicron booster Alveavax-v1.2 in Ad26.COV2.S-vaccinated adults
Source: iScience. 2025 Nov 10;28(12):113970. doi: 10.1016/j.isci.2025.113970 (PMC12704268; doi:10.1016/j.isci.2025.113970)
Supplement: Methods S2. Statistical analysis plan [file mmc5.pdf]

Methods S2: Statistical Analysis Plan

STATISTICAL ANALYSIS PLAN

Protocol No.:	Alvea-VAX-P00001
Protocol Title:	A phase 1 open-label, active-controlled, ramdomised dose-finding study to evaluate the safety, tolerability and immunogenicity of intradermal and subcutaneous application of the plasmid DNA SARS-CoV-2 Omicron BA.2 vaccine Alveavax-v1.2 in primary Ad26.CoV2.S vaccinated individuals
Drug:	Alveavax-v1.2
Sponsor:	Alvea, LLC Telis Bioscience Inc. 19 Blackstone St., Cambridge MA, 02139
Prepared by (Statistician):<br>Vaughan Reed<br>Micron Research Ltd.	DocuSigned by:<br>Vaughan Reed<br>Signer Name: Vaughan Reed<br>Signing Reason: I am the author of this document<br>Signing Time: 21-Oct-2022 | 12:32:46 PM BST<br>18090C283AFA4807AC7B9D79E4FE2D4E<br>21-Oct-2022<br>Signature : Date:
Approved by:<br>Russell Outlaw<br>Head of Data Management	DocuSigned by:<br>Russell Outlaw<br>Signer Name: Russell Outlaw<br>Signing Reason: I approve this document<br>Signing Time: 20-Oct-2022 | 7:22:31 PM BST<br>D999F3ACBEE343B0ACD2F02CB29F5117<br>20-Oct-2022<br>Signature : Date:
Approved by:<br>Sonia Sutherland<br>Head of Clinical Operations	DocuSigned by:<br>Sonia Sutherland<br>Signer Name: Sonia Sutherland<br>Signing Reason: I approve this document<br>Signing Time: 21-Oct-2022 | 10:08:07 AM BST<br>A5B7D38EB0854D8DB7B6A46A5E81A3C9<br>21-Oct-2022<br>Signature : Date:
Approved by:<br>Anemone Franz<br>Clinical Trial Physician	DocuSigned by:<br>Anemone Franz<br>Name des Unterzeichners: Anemone Franz<br>Signiergrund: Ich habe dieses Dokument geprüft<br>Signierzeit: 21-Oct-2022 | 5:30:11 AM EDT<br>E295BD4401BB45CA8E1B65F189E69436<br>21-Okt-2022<br>Signature : Date:
Statistical Analysis Plan: Version No. and Date	Version 1.0 20th October 2022

TABLE OF CONTENTS

STUDY OBJECTIVES 5

STUDY DESIGN 5

General Study Design and Plan 5
Randomization and Blinding 6
Sample Size Estimation 6
Study Schedules and Assessments 6
General Statistical Considerations 7

SUMMARY OF STUDY POPULATION DATA 8

Participant Disposition 8
Demographics and Baseline Characteristic 8

STUDY POPULATIONS 9

Safety Population 9
Modified Intent to Treat Population (mITT) 9
Per Protocol Population 9

STUDY VARIABLES AND ANALYSIS 9

Primary Efficacy Variable and Analysis 9
Secondary Efficacy Variables 9
Examination of Subgroups 11
Efficacy Measurements 12
Culture Results 12
Safety Variables 12
Adverse Events 12
Adverse Events of Special Interest (AESIs) 13
Medical History 14
Dosing and Extent of Exposure 14
Non-study Medications and Therapies 14
Vital Signs 15
Physical Examination 15
Clinical Laboratory Tests 15
Adjustments for Covariates 15
Handling of Missing Data 15
Interim Analyses and Data Monitoring 16
Multi-center Studies 16
Protocol Violations and Deviations 16
Other Safety Measures 16

CLINICAL PHARMACOLOGY ANALYSES 16

OTHER ANALYSES (EG, HEALTH RESOURCE UTILIZATION) 16

CHANGES IN THE STATISTICAL METHODS FROM THOSE STATED IN THE PROTOCOL 16

REFERENCES 16

TABLES, FIGURES, LISTINGS & OUTPUT 16

LIST OF ABBREVIATIONS

AE	adverse event
AESI	adverse event of special interest
AST	aspartate aminotransferase
BUN	blood urea nitrogen
CRO	contract research organization
ECG	electrocardiogram
eCRF	electronic case report form
EDC	Electronic Data Capture
hCG	human chorionic gonadotropin
ITT	Intent-to-Treat
kg	kilogram
MedDRA	Medical Dictionary for Regulatory Activities
mg	milligram
mITT	modified Intent-to-Treat
PI	Principal Investigator
RBC	red blood cell
SAE	serious adverse event
SAP	Statistical Analysis Plan
SOC	system organ class
TEAE	Treatment Emergent Adverse Events
WBC	white blood cell
WHO	World Health Organization

STUDY OBJECTIVES

This SAP is written with reference to Protocol Phase 1 Alveavax v1.2 trial (Alvea-VAX-P00001) version 3.0_2022-05-16.pdf and assumes that the reader has access to the protocol.

The only change between versions 2.0 and 3.0 of the protocol is the removal of treatment group 2A. This was participants who were unvaccinated but had been infected by Covid-19. This group was removed following protocol review by the safety committee, SAHPRA.

The primary objective is to evaluate the safety and tolerability of Alveavax-v1.2 in healthy participants compared to a control booster vaccine.

Secondary objectives are to evaluate the immunogenicity as a humoral immune response against SARS-CoV-2 BA.2/Omicron after a booster dose of Alveavax-v1.2 and to evaluate Clinical efficacy against SARS-CoV-2 after a booster dose of Alveavax-v1.2.

The effectiveness of intradermal vaccination will be compared with that of subcutaneous vaccination.

Exploratory objectives are to evaluate the cell-mediated immune response against SARS-CoV-2 BA.2/Omicron after a booster dose of Alveavax-v1.2

As a further exploratory endpoint Fc effector functions against SARS-CoV-2 after a booster dose of Alveavax-v1.2 may be evaluated. An Fc receptor is a protein found on the surface of certain cells, including natural killer cells, mast cells and macrophages. The Fc receptor binds to a specific region of gamma globulin antibodies that are attached to infected cells or invading pathogens. Their activity stimulates phagocytic cells, cytotoxic cells or complement to destroy microbes or infected cells. There are specific assays available for Fc effector receptors.

Furthermore, the humoral (gamma globulin antibodies) immune response against additional SARS-CoV-2 variants and sub-lineages after a booster dose of Alveavax-v1.2 will be evaluated.

The clinical efficacy will be correlated with the neutralizing antibody response.

In addition, the clinical efficacy and immunogenicity will be correlated with anti-nucleocapsid protein antibodies. The nucleocapsid protein is a very abundant protein of corona virus. It is highly immunogenic and is found in the cytoplasm of infected cells. Antibodies to the nucleocapsid protein are likely to be effective in destroying the virus.

STUDY DESIGN

General Study Design and Plan

130 healthy participants aged 18 to 65 will be included. They must have previously received a primary vaccination with the Janssen Ad26.COV2.S vaccine. They must satisfy all other inclusion and exclusion criteria set out in detail in the protocol.

The study is open label so there is no need for the treatment packs to match in the way that they would have to for a double-blind study. The vaccinated participants will be randomly allocated to one of five treatment groups as follows:

Group A: Low dose. 0.5 mg Alveavax-v1.2 in one ID injection
Group B: Standard dose. 2 mg Alvevax-v1.2 in one ID injection
Group C: High dose. 8mg Alvevax-v1.2 as four ID injections of 2mg
Group D: SC injection. 8mg Alvevax-v1.2 as a single SC injection
Group E: Control booster as a single IM injection

Groups A and C will have 20 participants each.
Groups B and E will have 40 participants each.
Group D will have 10 participants.

Recruitment will start with the low dose, Group A, and only progress to higher dose levels following safety assessments. No more than 5 participants will be vaccinated on the first day and escalation between dose levels will be allowed only after an independent medical monitor has reviewed at least 24-hour post-dose safety data. More detail of the randomisation and dose escalation process is given in the protocol.

Each participant will be administered a booster vaccine on Day 1 of the study and will be monitored afterwards for local or systemic reactions to the vaccine.

Participants will be given a diary card, thermometer and measuring template used to estimate the diameter of local reactions such as erythema or swelling. For the first seven days they will monitor temperature daily, record any local pain at the injection site and the size of swelling or erythema. Any other adverse events will also be recorded along with severity measured with a scale provided to them.

If there are symptoms of fever the participant is to report the maximum temperature each day until the temperature has returned to normal.

Randomization and Blinding

The study is open label so no blinding is required. The randomisation will be performed centrally and a separate randomisation plan will be drawn up to detail the methods.

Sample Size Estimation

There is no formal sample size calculation. The planned recruitment is based on previous experiences of similar vaccine studies.

Study Schedules and Assessments

The follow up assessment points are summarised below:

Day 7 (± 2 days)

- Abbreviated, symptom-directed physical examination
- Vital signs
- Recording of adverse events

- Clinical laboratory evaluation (haematology and chemistry)
- Blood sample collection for immunogenicity and cellular response analysis
- NP swab sample for COVID-19 PCR
- Review and collection of Diary Card
- Recording of COVID-19 event

Day 14 (± 2 days), Day 28 (± 2 days), Day 84 (± 7 days), and Day 168 (± 14 days)

- Recording of adverse events
- Blood sample collection for immunogenicity and cellular response analysis
- Confirm with female participants of childbearing potential and male participants who have a female partner if they/their partner became pregnant since Day 1
- Recording of COVID-19 infections and vaccinations since Day 1
- NP swab sample for COVID-19 PCR on Day 14 and Day 28

Day 365 (± 14 days) (End-of-Study)

- Abbreviated, symptom-directed physical examination
- Vital signs and weight
- Recording of adverse events
- Blood sample collection for immunogenicity and cellular response analysis
- Recording of COVID-19 infections and vaccinations since Day 1
- Confirm with female participants of childbearing potential and male participants who have a female partner if they/their partner became pregnant since Day 1 of the study.

General Statistical Considerations

All tables, listings and graphs will be produced by Micron Research (Ely, UK) using SAS® Version 9.4 or later (SAS Institute, Cary, NC 27513). Specifications for tables, graphs, and data listing formats can be found in the Tables Manual for this study. All data collected on the eCRF will be listed by participant; all randomised participants will be included in the listings.

Unless otherwise stated, all summaries will be presented by treatment group using the column headings Group A, B, C, D and E plus a Total column of groups A to D combined.

Categorical variables will be summarised as frequencies and percentages. Unless otherwise stated, the denominator for percentages will be the number of participants in each treatment group in the analysis population. Percentages will be presented to 1 decimal place. However, percentages will not be presented against zero counts in the tables. A "Missing" category will only be presented on the categorical summaries if the Investigator actually recorded Missing as an outcome.

Continuous variables will be summarised using descriptive statistics (number of participants with an observation (n), mean, standard deviation (SD), median, minimum (min) and maximum (max). Unless otherwise specified, all continuous variables will be given to one decimal place. No hypothesis tests are planned but where confidence intervals are reported they will be at the 95% level. In general, where partial dates are recorded on the eCRF

(missing day or missing day and month) and where these cannot be resolved by queries, dates will be estimated for the purpose of calculating durations. Where a start/onset date is partial, the first day of the month will be assumed if the day is missing and the first month of the study will be assumed if the month is missing. Where a stop/end date is partial, the last day of the month will be assumed if the day is missing and the final month of the study will be assumed if the month is missing. Partial concomitant medication and AE dates may need to be dealt with on a case by case basis and this will be discussed with the sponsor where applicable.

SUMMARY OF STUDY POPULATION DATA

Data listings will include all participants. Only the reason for discontinuation will be listed for participants classed as screening failures (i.e., a participant who had signed informed consent and had failed any inclusion or exclusion criteria or withdrew before any treatment had started).

Participant Disposition

The number of participants screened, the number of participants enrolled, including the number of participants enrolled but not treated and the number of participants enrolled and treated, will be summarised by region and overall. Treated participants will be defined as those that received at least one dose of study medication. The number and percentage of participants treated, the number and percentage of participants in each analysis population (defined under Study Populations below), the number and percentage of participants completing the study and the number and percentage of participants withdrawing from the study, including the reasons for withdrawal, will be summarised for all enrolled participants.

Demographics and Baseline Characteristic

Descriptive summaries of participants' demographic and baseline characteristics will be presented for the intent to treat (ITT) population by treatment group, region and overall. Demographics will include: age (years), sex, race, height (m), weight (kg) and BMI (kg/m^2). Age will be calculated from the date of birth (or year of birth) relative to the screening visit date if DOB (or year of birth) is recorded. The following baseline values of the immunogenicity parameters will be summarised by study group:

Geometric mean titer (GMT) of serum anti-SARS-CoV-2 BA.2 antibody.
GMT of BA.2 anti-spike protein (S) antibody titers.
BA.2 Spike specific CD4+ T-cell count
BA.2 Spike specific CD8+ T-cell count
GMT of anti-nucleocapsid protein (N) antibody titers.
Percentage of participants with neutralization IC50≥100 IU/mL
Percentage of participants with neutralization IC50>200 IU/mL
Percentage of participants with neutralization IC50>400 IU/mL
Percentage of participants with neutralization IC50>800 IU/mL
Baseline assessments of antibody dependent cellular cytotoxicity, phagocytosis and trogocytosis.
Baseline measures of complement fixation
Baseline measures of Fc receptor binding.

Baseline RLU of

- Antibody dependent cellular cytotoxicity (ADCC)
- Antibody dependent cellular phagocytosis (ADCP)
- Antibody dependent cellular trogocytosis
- Complement deposition
- Fc dimer receptor binding

STUDY POPULATIONS

Safety Population

The safety population is the set of all enrolled participants who have been administered a dose of the investigational product. Participants will be grouped as treated.

Modified Intent to Treat Population (mITT)

All enrolled participants who have been administered with the vaccine and experience at least one post-baseline immunogenicity readout will comprise the mITT population. Missing or non-evaluable measurements will not be replaced. Participants will be grouped as treated.

Per Protocol Population

The per-protocol analysis population includes all enrolled participants who meet all the inclusion/exclusion criteria and do not have any major protocol deviations. Participants will be grouped as treated.

STUDY VARIABLES AND ANALYSIS

Primary Efficacy Variable and Analysis

The primary efficacy variable is the change from baseline of the geometric mean of serum anti-SARS-CoV-2 BA.2 antibody titers. These data will be summarised by treatment group at every post-dose assessment. The analysis will be performed on the Per Protocol population, the conservative approach in a positive control or non-inferiority study.

The primary aim of the study is to assess tolerability to Alvevax-v1.2 and to establish the optimum dose and so the efficacy analyses will all be purely descriptive with no statistical comparisons.

Secondary Efficacy Variables

The Primary efficacy analysis will be repeated with the mITT population and the actual geometric mean titration values (as opposed to the change from baseline) will be summarised by study visit.

The fold rise in geometric mean serum anti-SARS-CoV-2 BA.2 antibody titers will be summarised as continuous data by study visit and the number and percentage of participants having a 4 fold or greater rise in serum anti-SARS-CoV-2 BA.2 antibody titers will be summarised as frequencies and percentages at each study visit.

The number and percentage of participants having an IC50 of neutralizing BA.2 antibodies >100 IU/ml, >200 IU/ml, >400 IU/ml and > 800 IU/ml will all be summarised as frequencies and percentages with 95% confidence limits, at each study visit.

The change from baseline in geometric mean of serum anti-SARS-CoV-2 BA.2 anti-spike protein (S) antibody titers, will be summarised by treatment group at each study visit. The actual geometric mean of the anti-spike protein (S) antibody will be summarised at each visit. The fold rise anti-spike protein (S) will be summarised as continuous data at each study visit and the number and percentage of participants having a 4 fold or greater rise in anti-spike protein antibody titers will be summarised by study visit.

The serum anti-SARS-CoV-2 BA.2 Spike-specific CD4+ and CD8+ cell counts and the change from baseline will be summarised as continuous data by study visit.

The change from baseline in geometric mean of serum anti-SARS-CoV-2 BA.2 anti-nucleocapsid protein (N) antibody titers, will be summarised by treatment group at each study visit. The actual geometric mean of the anti-nucleocapsid protein (N) antibody will be summarised at each visit. The fold rise anti-nucleocapsid protein will be summarised as continuous data at each study visit and the number and percentage of participants having a 4 fold or greater rise in anti-nucleocapsid protein antibody titers will be summarised by study visit.

Assay methods for antibody dependent cellular cytotoxicity, phagocytosis, trogocytosis (defined as nibbling at infected cells) as well as complement fixation and Fc receptor binding make use of firefly luciferin to emit light in direct linear response to the antibody activity. These assays will be used to test ancestral variants of covid such as Alpha, Beta and Delta and any new sub-variants that might arise.

The number of light units for each parameter will be summarised as continuous data by visit for the respective strain. Where baseline values are available the change from baseline will also be summarised by study visit.

The antibody titers against ancestral variants such as Alpha, Beta and Delta and any new sub-variants that might arise will be summarised as change from baseline in geometric mean titer, the actual geometric mean titer and the fold rise of serum anti-SARS-CoV-2 antibodies. Titers of serum anti-S and anti-S-RBD antibodies will be reported similarly.

The number and percentage of participants with neutralisation IC50 >64 IU/ml and >128 IU/ml against the respective strain, will be summarised by study visit.

Clinical Assessment

At each study visit, participants will be checked for symptoms of COVID-19 and where symptoms are present, they will be scored on the WHO clinical progression scale.

Patient State	Descriptor	Score
Uninfected	Uninfected; no viral RNA detected	0
Ambulatory mild disease	Asymptomatic; viral RNA detected	1
	Symptomatic; independent	2
	Symptomatic; assistance needed	3
Hospitalised: moderate disease	Hospitalised; no oxygen therapy*	4
	Hospitalised; oxygen by mask or nasal prongs	5
Hospitalised: severe diseases	Hospitalised; oxygen by NIV or high flow	6
	Intubation and mechanical ventilation, pO2/FiO2 ≥150 or SpO2/FiO2 ≥200	7
	Mechanical ventilation pO2/FIO2 <150 (SpO2/FiO2 <200) or vasopressors	8
	Mechanical ventilation pO2/FiO2 <150 and vasopressors, dialysis, or ECMO	9
Dead	Dead	10

ECMO=extracorporeal membrane oxygenation. FiO2=fraction of inspired oxygen. NIV=non-invasive ventilation. pO2=partial pressure of oxygen. SpO2=oxygen saturation. *If hospitalised for isolation only, record status as for ambulatory patient.

The scores will be summarised at each study visit as frequencies and percentages.

Following vaccination participants recorded the size of the bleb produced if it was clearly visible for at least 20 seconds. The number and percentage of participants recording a bleb will be summarised as frequencies and percentages. The maximum size recorded by each participant will be summarised as continuous data.

Examination of Subgroups

No analysis of sub-groups is planned. Any additional analysis, to clarify existing results for example, requested by the Sponsor will be included in the final report.

Efficacy Measurements

Much of the efficacy data will be reported as geometric means. The calculation of geometric means is as follows:
The $\log_2$ values are calculated for all the titration results.
The log mean value is calculated as the mean of the log values.
The geometric mean is calculated as $2^{\text{log mean}}$

Any log and corresponding anti-log could be used for the calculation but assuming the titration is of halving dilutions there is a basis for using $\log_2$.

Changes from baseline will be by simple subtraction of the baseline value from the visit value of the geometric mean. No statistical analysis is planned on these comparisons.

Culture Results

Not appropriate

Safety Variables

The investigator will evaluate safety by adverse events monitoring, vital signs, physical examination, clinical laboratory tests and concomitant medications.

Adverse Events

Participants will be assessed for the occurrence of adverse events (AEs) throughout the study but with special emphasis on the first seven days following vaccination. Adverse events specifically related to vaccination will be documented by the investigator. All other adverse events reported by the participants will also be recorded. All adverse events encountered during the study will be reported in the eCRF. If possible, a diagnosis should be documented rather than signs and symptoms. All summary tables of safety data will be presented for the Safety population.

AEs will be coded using MedDRA version 24.1 or later. Coding will include the system organ class and preferred term. Verbatim descriptions and coded terms will be listed for all AEs. One listing will be produced for all AEs (serious and not serious) and a separate listing will also be produced for serious AEs only. For each participant, multiple occurrences of the same event will be counted only once within a system organ class and preferred term i.e., as patient events. Summaries will be presented by decreasing frequency of participants with each event in the C, High Dose group. The denominator for percentages will be the number of participants in the respective treatment groups.

The following adverse event tables will be produced:

All adverse events throughout the study.
All serious adverse events throughout the study.
All adverse events regarded as possibly or probably related to the vaccination.
All adverse events leading to withdrawal from the study.
All adverse events where the outcome was fatal.

All adverse events by severity, Mild, Moderate, Severe or Life Threatening. In the event that a participant has more than one report of the same AE only the greatest severity will be recorded in this table.

During the first seven days (day of vaccination and next 6 days) following vaccination, the participants were to record in their diary cards the occurrence and severity of the following signs and symptoms:

**Local (at injection site)**	**Systemic**
• Pain<br>• Erythema (Redness)[A]<br>• Swelling[A]<br>• Induration[A]<br>• Ecchymosis (bruising)[A]<br>• Tenderness<br>• Itching	• Fever [≥ 38.0°C or ≥100.4^{0}F][B]<br>• Headache<br>• Chills/shivering<br>• Fatigue<br>• Nausea<br>• Vomiting<br>• Diarrhoea<br>• Malaise<br>• Myalgia (muscle pain)<br>• Arthralgia (joint pain)

A. Record surface diameter in mm
B. Record tympanic or oral temperature

A table will be produced for each of these signs and symptoms by treatment group and study day. The tables will show the total number of participants reporting the respective event and by study day the maximum severity reported by each participant as frequencies and percentages.

For signs that have been measured, the maximum size rather than severity will be reported and for fever the maximum temperature each day will be reported, both as continuous data.

Adverse Events of Special Interest (AESIs)

AESIs, based on the Safety Platform for Emergency vACcines (SPEAC), are reported until the end of the safety follow-up period:

1. Cardiovascular: Myocarditis, pericarditis
2. Dermatologic: cutaneous vasculitis
3. Hematologic: Coagulopathy, stroke, venous thromboembolism including pulmonary embolism, other thrombosis, thrombocytopenia, and endothelial dysfunction
4. Neurologic: Acute disseminated encephalomyelitis (ADEM), aseptic meningitis, encephalitis, facial nerve palsy including Bell's palsy, Generalised convulsive seizure, Guillain Barre Syndrome (GBS), myelitis, Sensorineural hearing loss (SNHL)

5. Respiratory: Acute Respiratory Distress Syndrome (ARDS)
6. Systemic: Anaphylaxis, Multisystem Inflammatory Syndrome in Children and Adults (MIS-C/A), Vaccine Associated Enhanced Disease (VAED)
7. Vasculitis: Single organ cutaneous vasculitis
8. Rhabdomyolysis
9. Subacute thyroiditis
10. Acute pancreatitis

Adverse events of special interest are to be recorded by the Investigator on the AESI page of the eCRF but will also be reported in the main AE tables.

The following tables will report the AESIs:
All AESIs throughout the study.
All serious AESIs throughout the study.
All AESIs regarded as possibly or probably related to the vaccination.
All AESIs leading to withdrawal from the study.
All AESIs where the outcome was fatal.

[Note: if only a small number of AESIs are reported the tables listed above will be replaced with individual participant listings]

Medical History

All medical/surgical history will be summarised for the mITT population by category showing the number and percentage of participants reporting each category. All verbatim descriptions will be listed.

Dosing and Extent of Exposure

Any deviations from the planned vaccination program will be recorded and summarised as frequencies and percentages. In the event that a participant received the wrong vaccination, they will be included in the treatment group of the vaccination that they actually received.

Non-study Medications and Therapies

Concomitant medications will be coded using the World Health Organization Drug Dictionary (WHO-DRUG Global March 2019) and the Anatomical Therapeutic Chemical (ATC) classification system. Coding will include the drug class and generic drug name for single active constituents.

Concomitant medications will be defined as:

- Non-study medications received from the time of randomization up to the final visit
- Non-study medications that started prior to the time of randomization and are ongoing during the study

All concomitant medications will be summarised as frequencies and percentages by ATC class.

From 1 year until 30 days before vaccination all immunoglobulins, immunostimulants and vaccines received by the participants will be recorded. These will be summarised as a separate table, classified in the same way as concomitant medication.

All non-study medication will be listed including the verbatim terms and indication where it is recorded.

Vital Signs

Systolic and diastolic blood pressure, heart rate (pulse) and temperature will be assessed at Screening and at all study visits. The data will be summarised as continuous data by study visit.

Physical Examination

A history-directed physical examination will be done at baseline, followed by an abbreviated, symptom-directed physical examination at subsequent time points. Any abnormalities will be summarised as frequencies and percentages by study visit.

Clinical Laboratory Tests

Blood and urine will be collected at specified visits for laboratory testing (clinical chemistry, hematology). Urine collected at Screening/Baseline will be used for pregnancy testing. The specific tests to be performed are listed below:
Clinical Chemistry: Alanine aminotransferase (ALT), Aspartate aminotransferase (AST), Blood urea nitrogen (BUN), Creatinine and Total bilirubin.
Hematology: Hemoglobin, Platelets, Red blood cells (RBCs), Total white blood cells (WBCs) and differential.
Urinalysis: hCG pregnancy test.

Where multiple laboratories have been used the values will be standardised as SI units. The data will be summarised as means, SD, medians, Minimum and Maximum values and also as number and percentage of results above and below the normal range.

Blood samples for clinical chemistry and hematology will be taken at screening, enrolment (if more than seven days after screening) and at study day 7.

All parameters will be summarised as continuous data at all three visits. Further tables will show the change from baseline, also as continuous data, and values which were outside the laboratory normal range will be summarised as frequencies and percentages.

Adjustments for Covariates

Not relevant to this study

Handling of Missing Data

No imputation of missing data will be performed for this study.

Interim Analyses and Data Monitoring

Interim review of safety findings will occur during the study. Interim summary of immune response may occur periodically through the study for phase 2 planning. No modification to this study will occur based on these analyses. No corrections for multiple testing are planned as these analyses will be used only to inform phase 2 study design.

Multi-center Studies

Tests of homogeneity across centres are not relevant to this study.

Protocol Violations and Deviations

Protocol violations will be recorded during the study and will be used to determine whether participants should be included in the Per Protocol Population.

Other Safety Measures

There are no other safety measures.

CLINICAL PHARMACOLOGY ANALYSES

Not relevant to this study.

OTHER ANALYSES (EG, HEALTH RESOURCE UTILIZATION)

None.

CHANGES IN THE STATISTICAL METHODS FROM THOSE STATED IN THE PROTOCOL

No changes have been made from the methods outlined in the protocol.

REFERENCES

None.

TABLES, FIGURES, LISTINGS & OUTPUT

All tables, listings, figures (and any other output, where applicable) will be described in a separate “Tables Manual” document (Alvea Tables Manual) which will form an attachment to this SAP.
