# Supplementary material for: Safety and immunogenicity of DNA omicron booster Alveavax-v1.2 in Ad26.COV2.S-vaccinated adults
Source: iScience. 2025 Nov 10;28(12):113970. doi: 10.1016/j.isci.2025.113970 (PMC12704268; doi:10.1016/j.isci.2025.113970)
Supplement: Methods S3. Informed consent sample storage [file mmc6.pdf]

Methods S3: Informed Consent Sample Storage

INFORMATION AND INFORMED CONSENT DOCUMENTFOR FUTURE USE IN SUB-STUDIES

Human Biological Samples and Data Collected and Stored for Future Use

Title:	A Phase 1 open-label, active-controlled, randomized dose-finding study to evaluate safety, tolerability, and immunogenicity of intradermal and subcutaneous application of the plasmid DNA SARS-CoV-2 Omicron BA.2 vaccine Alveavax-v1.2 in primary Ad26.COV2.S vaccinated healthy individuals.
Sponsor:	Telis Bioscience Inc.<br>19 Blackstone St, Cambridge, MA 02139
Principal Investigator:	XXXXXXXXX Phone : XXXXXXXX

This consent document is in addition to the Informed Consent Document and Authorisation for the Alveavax-v1.2 Phase 1 trial. You have already signed the main consent document. This additional consent is not intended to replace or modify any information contained in the main consent document. Now we are asking you to also take part in a sub-study for your biological samples (samples which may contain cells from your body, such as blood or urine) and data to be collected and stored for future use. A sub-study is a part of another study. You can choose to take part in this sub-study or not.

This sub-study document may have words that you do not understand. Please ask the study doctor and study staff to explain any words that you do not know, or anything that you do not understand. You must not sign this sub-study document until you understand all of the information on these pages and your questions have been answered. You should keep a signed copy of this consent document.

The study doctor will be in control of doing the sub-study. The sponsor, called Alvea, LLC. / Telis Bioscience Inc., will pay for this sub-study.

A description of this clinical trial will be available on http://sanctr.samrc.ac.za/. This website will not include information that can identify you. You can search this website(s) at any time.

Background and reasons

New medicines are being made that are safer, work better, or have other advantages against the SARS-CoV-2 virus. There may be some new questions that we did not think about at the time we did the main study. In the main study, we took blood and nasal swab samples from you, and we will use some of these leftover samples of blood in the sub-study.

This may help us to do more studies in the future. We may be able to find out why we get sick (diseases) or why things in our body don't work properly. We may find out how medicines work to cure diseases and why some people get better, and other people don't with the same medicine.

In a future research study, we may use your leftover sample(s) to learn about one or more of the following:

1. to better understand the immune responses to the vaccine(s) under study in this study,
2. to understand how different people respond to different vaccines,
3. for vaccine-related tests supporting vaccine programs,
4. to learn about other diseases or other medicines.

The sponsor will only test your leftover sample(s) to help answer some questions which are part of a research study we will do in the future, and not for any other reasons. Nothing else will be done with your samples in this sub-study. Only your blood and nasal swab samples collected during the main study will be used in the sub-study.

If you agree to your leftover samples being used, then the sponsor would like to keep some personal information about you. This information will be collected from your medical file or your other health records, and from the results from examining your sample(s). This information is called "data".

Your agreement to give sample(s) for this sub-study and for the sponsor to use your information is important for research but ***is entirely up to you.*** You can still take part in the main study even if you decide not to take part in this sub-study.

Are there any dangers when giving sample(s)?

There is no danger for you if you agree to be in the sub-study.

Will I be paid?

No. There is no payment for taking part in this sub-study.

What are the benefits of taking part in this sub-study?

There are no special advantages for you to take part in this sub-study. Your participation in this sub-study may help us to understand the disease better and the ways in which we can treat it.

When you agree to give us your leftover sample(s), you also give up your right to own those sample(s) and all your rights to those sample(s). However, you still have the right to cancel this agreement (consent) if you want to. This would mean that your sample(s) and information won't be stored and used.

Data privacy and confidentiality

How will my data be protected?

Some other people who do not work on this study may get hold of your information and use it in the wrong way. The sponsor has put protections in place to help stop this from happening. Your name and other information that can identify you will not be a part of your sample(s). This will help to prevent other people from finding out who you are if they get hold of your sample(s). A number code instead of your name or any other information about you will be used on your sample(s). Only this number code will be linked to

your sample(s) at the study centre. The study doctor and study staff will be the only people who will be able to connect your information to this number code on your sample(s) at the study centre.

In case the sponsor will send your sample(s) to another company, this company has to keep your sample(s) confidential. All information that can identify you will be taken away from your sample(s) before any testing or storage is done. However, if a problem is found in the main study, for example if there is a serious safety problem, then some people may have to be allowed to see information about you that shows your identity. These are people with legal permission, or people who belong to a regulatory board (people who check that the study is done properly) or health authorities. If this happens, the information that identifies you may have to be connected back to the sample(s) in the sub-study.

Will I be informed about the results of the sub-study?

You will not get the results of this sub-study unless the law says that you should. This sub-study is only for trying to find out about new things (exploratory research). It is not for collecting any special information that is useful to you or to your doctor. These research tests are done differently than the kinds of laboratory tests that your doctor orders for clinical tests. It would not be useful or appropriate to give you or your doctor these results. The sponsor will not give the results to your family, your insurance company, or your employer. The results from this sub-study will not become part of your medical records. The results will only be used as described in this sub-study consent form.

The sponsor will contact all the study doctors in this study if a serious problem (safety result) is found in the sub-study. The sponsor will also offer to pay for clinical tests for this serious safety problem while you are still a part of the main study. If important results are found after you have stopped taking part in the main study, the sponsor will publish these results and discuss them at public meetings. The results will be made open to the public on a public website. This is so that this information can quickly reach the doctors and people, like you, who may have been in the sub-study. All of the information that can identify you will be removed from any document or presentation of results.

What will happen to my sample(s)?

Your sample(s) will be sent to Cytespace Africa Laboratories, Centurion, Gauteng and BARC SA Pty Ltd, Richmond, Johannesburg, Gauteng to be stored. Researchers and the people who work with the sponsor have to get special permission from the sponsor to work with your sample(s). All these researchers will have to use and protect your sample(s) according to the laws, correct guidelines and this sub-study consent document.

Your sample(s) will be safely stored for up to 10 years. This could be changed by an independent medical board or ethics committee, and be kept for longer. Your sample(s) will be kept that long because of certain regulatory authorities' laws. At the end of that time, your sample(s) will be destroyed properly. The sponsor may also destroy your stored sample(s) for any reason without asking if this is okay. Sometimes the sponsor has to answer questions from authorities about your sample(s). Then your samples may have to be stored for a longer time. In this special case, sample(s) will be stored until all of these questions have been properly answered.

You will not be asked to sign any other agreement/consent about your leftover sample(s) being used in research. You will also not be told when the tests are done on your sample(s). Any possible research that uses your sample(s) in the future will be checked by a committee of the sponsor's scientists and doctors.

An outside group of people called "biomedical ethicists" will also watch over the research (people who check that things are done in the right way).

Changes to participation

How can I leave or stop the sub-study?

You can say no to joining the sub-study, or you can leave this sub-study at any time. Nothing will happen to you, and you will not lose any advantages. If you decide not to be a part of this sub-study anymore, you must tell your study doctor that no one can use your sub-study sample(s) and information. If you choose not to join, or to leave the sub-study, your study doctor and the study staff will carry on taking care of you. If you stop your permission for people to use your leftover sample(s), no more information will be collected from these samples. Your sample(s) will then be destroyed. However, the information that has been collected before you leave the sub-study will still be used. We will not be able to take back your information that has already been used or has been given to other people. We can't remove your information that is already part of a large set of information that is being shared and used for future research.

You may stop your permission for using your leftover samples. But if your samples can't be identified, or have been made anonymous (no information about you) by accident (for example because one of the key codes can't be found), then we will still continue to use these anonymous sample(s). This is in line with the agreements in this sub-study consent document.

Your sample(s) and information used for this sub-study will not automatically be destroyed if you leave the main study. If you want to leave this sub-study, you will have to ask for your sample(s) from this sub-study to be destroyed after you leave the main study.

Contact details

If you have questions about this trial, you should first discuss them with your doctor or the South African Medical Association Research Ethics Committee (SAMAREC):

Address: Block F, Castle Walk office Park, Nossob Street, Erasmuskloof Ext 3 Pretoria

Tel: (012) 481 2082
Fax: (012) 481 2095
E-mail: samarec@samedical.org

After you have consulted your doctor or the Ethics Committee and if they have not provided you with answers to your satisfaction, you should write to the South African Health Products Regulatory Authority (SAHPRA) at:

The Chief Executive Officer
South African Health Products Regulatory Authority
Department of Health
Private Bag X828
PRETORIA

0001
E-mail: Boitumelo.Semete@sahpra.org.za Tel: 012 842 7629/7626

Informed Consent

- I hereby confirm that I have been informed by the study doctor about the nature, conduct, benefits and risks of this clinical trial.
- I am aware that the results of the trial, including personal details regarding my sex, age, date of birth, initials and diagnosis will be anonymously processed into a trial report, but that some of my health information may be reasonably disclosed to the Sponsor and/or authorities under certain circumstances.
- I may, at any stage, without prejudice, withdraw my consent and end my participation in the trial.
- I have had sufficient opportunity to ask questions and (of my own free will) declare myself prepared to participate in the trial.
- I have read and understood the contents of the document.
- I understand that I shall receive a signed copy of this document.

Participant:		
Printed name	Signature	Date

I, Dr____ herewith confirm that the above participant has been informed fully about the nature, conduct and risks of the above trial.

Study Doctor:		
Printed name	Signature	Date

Verbal Participant Informed Consent

(This section is applicable when participants cannot read or write and should replace the previous Informed Consent section)

I, the undersigned study doctor, Dr________________________________ , hereby confirm that:

- I have read and explained fully, to the participant, named ________________________________ as well as the witness who signed below, the content of this document, indicating the nature and purpose of the trial in which I have asked the participant to participate.
- Verbal consent of the participant was obtained for the witness to be present during the consenting process.
- I have explained both the possible risks and benefits of the trial and the alternative treatments available for his/her illness.
- The participant has indicated that he/she understands the contents of the document and also that he/she will be free to withdraw from the trial at any time without giving any reason or jeopardising his/her subsequent treatment.
- I have informed the participant on the existence of relevant compensation arrangements in case of an injury attributable to the drug(s) used in the clinical trial, to which he/she agrees.
- The participant has had sufficient opportunity to ask questions.
- The participant has voluntarily agreed to participate in this trial.

Participant:		
Printed name	Signature (if possible)	Date

Study Doctor:		
Printed name	Signature	Date

I, the witness who signed below, confirm that the study doctor has explained fully the content of this document to the participant.

Witness:		
Printed name	Signature	Date

(Witness' signature confirms that he/she has witnessed the relevant signatures at the time of signing. Witness name, signature and date must be completed by the witness at the same time that this document is signed and dated by the participant and the Study Doctor. A competent witness is a person 16 years or older and of sound mind and not involved with the trial.)
