# Supplementary material for: Safety and immunogenicity of DNA omicron booster Alveavax-v1.2 in Ad26.COV2.S-vaccinated adults
Source: iScience. 2025 Nov 10;28(12):113970. doi: 10.1016/j.isci.2025.113970 (PMC12704268; doi:10.1016/j.isci.2025.113970)
Supplement: Methods S4. Informed consent HIV test [file mmc7.pdf]

Methods S4: Informed Consent HIV Test

HUMAN IMMUNODEFICIENCY VIRUS (HIV) TEST PARTICIPANT INFORMATION AND INFORMED CONSENT DOCUMENT

Title: A Phase 1 open-label, active-controlled, randomized dose-finding study to evaluate safety, tolerability, and immunogenicity of intradermal and subcutaneous application of the plasmid DNA SARS-CoV-2 Omicron BA.2 vaccine Alveavax-v1.2 in primary Ad26.COV2.S vaccinated healthy individuals.

Sponsor: Telis Bioscience Inc.
19 Blackstone St, Cambridge, MA 02139

Principal Investigator: XXXXXXXXX Phone : XXXXXXXX

This consent document is in addition to the Informed Consent Document and Authorisation for the Alveavax-v1.2 Phase 1 trial. You have already signed the main consent document. This additional consent is not intended to replace or modify any information contained in the main consent document. You should keep a signed copy of this consent document.

To take part in this research project, we will test your blood for evidence of HIV infection. Your HIV test results will be returned to you. Before agreeing to participate in this research study, you must understand the implications of HIV testing. You are free to refuse this test, but you will not be allowed to participate in this research project if you refuse.

Infection with HIV leads to acquired immune deficiency syndrome (AIDS). It can be transmitted through unprotected sex (vaginal, anal, or oral sex) with someone who has HIV and through contact with blood, as in sharing needles (piercing, tattooing, reusing needles used to inject drugs). HIV-infected pregnant women can transmit to their infants during pregnancy, delivery, or breastfeeding.

There are treatments for HIV/AIDS that can help an individual stay healthy. Individuals with HIV/AIDS can adopt safe practices to protect uninfected people in their lives from becoming infected and to protect themselves or infected people in their lives from being infected with different strains of HIV.

Purpose of HIV testing

This test is performed to exclude any HIV-positive participants from participating in this study as these participants are more likely to have increased risks. Additionally, the nature of the disease is such that it may interfere with the assessment of the study vaccine.

Procedure

The HIV test conducted for this study will be carried out on a blood sample collected from a vein. This test can detect antibodies made by your immune system when HIV is present.

A negative test means that it is extremely unlikely that you are infected with HIV. If you had a recent exposure (less than three months ago), an additional test would be done to confirm whether you are in the "window" period of HIV infection.

A positive HIV test means that it is very likely that you have been infected with HIV. This test does not determine how advanced the illness is, and it is not a test for AIDS. A positive HIV test makes you ineligible for the study. You will be referred for further testing and counselling.

If you test positive for HIV, you will not hold the sponsor or any personnel at the study site liable for compensation or treatment.

Possible advantages of discovering an HIV positive status:	Possible disadvantages of discovering an HIV positive status:
- Access to specialised health care and counselling<br>- Preventing the transmission to your sexual partners<br>- Testing your partner for HIV<br>- Preventing mother-to-child HIV transmission<br>- Preventing transmission through blood donations	- Emotional stress, depression, and despair<br>- Stigmatisation on disclosure<br>- Discrimination on disclosure<br>- Rejection by family, friends, sexual partners, and/or spouse

Risks

Side effects from the blood draw are minimal. They include feeling faint, redness, pain, bruising, or bleeding at the puncture site.

Expenses and Payments

You will not be paid for your participation in this HIV testing. However, all associated costs of the HIV test, as well as the first post-test counselling, will be paid for by the Sponsor; Alvea, LLC. / Telis Bioscience Inc.

Confidentiality

Blood samples for this HIV test will not be stored. Your HIV testing information is strictly confidential and cannot be released to anyone without your written consent. Your HIV test result information will be recorded in your study health records for use by Alvea, LLC. / Telis Bioscience Inc. personnel and its designees. The general consent to healthcare and information release does not cover HIV-related

information. You are free to keep your HIV testing result to yourself. However, it is essential to notify your sexual partners and those who might have been exposed to your blood.

Contact details

If you have questions about this trial, you should first discuss them with your doctor or the South African Medical Association Research Ethics Committee (SAMAREC):

Address: Block F, Castle Walk office Park, Nossob Street, Erasmuskloof Ext 3 Pretoria
Tel: (012) 481 2082
Fax: (012) 481 2095
E-mail: samarec@samedical.org

After you have consulted your doctor or the Ethics Committee and if they have not provided you with answers to your satisfaction, you should write to the South African Health Products Regulatory Authority (SAHPRA) at:

The Chief Executive Officer
South African Health Products Regulatory Authority
Department of Health
Private Bag X828
PRETORIA
0001
E-mail: Boitumelo.Semete@sahpra.org.za
Tel: 012 842 7629/7626

Informed Consent

- I hereby confirm that I have been informed by the study doctor about the nature, conduct, benefits and risks of this clinical trial.
- I am aware that the results of the trial, including personal details regarding my sex, age, date of birth, initials and diagnosis will be anonymously processed into a trial report, but that some of my health information may be reasonably disclosed to the Sponsor and/or authorities under certain circumstances.
- I may, at any stage, without prejudice, withdraw my consent and end my participation in the trial.
- I have had sufficient opportunity to ask questions and (of my own free will) declare myself prepared to participate in the trial.
- I have read and understood the contents of the document.
- I understand that I shall receive a signed copy of this document.

**Participant:**		
**Printed name**	**Signature**	**Date**

I, Dr____ herewith confirm that the above participant has been informed fully about the nature, conduct and risks of the above trial.

**Study Doctor:**		
**Printed name**	**Signature**	**Date**

Verbal Participant Informed Consent
(This section is applicable when participants cannot read or write and should replace the previous Informed Consent section)

I, the undersigned study doctor, Dr________________________________ , hereby confirm that:

- I have read and explained fully, to the participant, named_____________________________________ as well as the witness who signed below, the content of this document, indicating the nature and purpose of the trial in which I have asked the participant to participate.
- Verbal consent of the participant was obtained for the witness to be present during the consenting process.
- I have explained both the possible risks and benefits of the trial and the alternative treatments available for his/her illness.
- The participant has indicated that he/she understands the contents of the document and also that he/she will be free to withdraw from the trial at any time without giving any reason or jeopardising his/her subsequent treatment.
- I have informed the participant on the existence of relevant compensation arrangements in case of an injury attributable to the drug(s) used in the clinical trial, to which he/she agrees.
- The participant has had sufficient opportunity to ask questions.
- The participant has voluntarily agreed to participate in this trial.

**Participant:**		

**Printed name**	**Signature (if possible)**	**Date**

**Study Doctor:**		
**Printed name**	**Signature**	**Date**

I, the witness who signed below, confirm that the study doctor has explained fully the content of this document to the participant.

**Witness:**		
**Printed name**	**Signature**	**Date**

(Witness' signature confirms that he/she has witnessed the relevant signatures at the time of signing. Witness name, signature and date must be completed by the witness at the same time that this document is signed and dated by the participant and the Study Doctor. A competent witness is a person 16 years or older and of sound mind and not involved with the trial.)
