# Supplementary material for: Safety and immunogenicity of DNA omicron booster Alveavax-v1.2 in Ad26.COV2.S-vaccinated adults
Source: iScience. 2025 Nov 10;28(12):113970. doi: 10.1016/j.isci.2025.113970 (PMC12704268; doi:10.1016/j.isci.2025.113970)
Supplement: Methods S5. Informed consent core [file mmc8.pdf]

Methods S5: Informed Consent Core

PARTICIPANT INFORMATION AND INFORMED CONSENT DOCUMENT

(Each participant must receive, read, and understand this document before the start of the study)

TRIAL NUMBER: Alvea-VAX-P00001

TRIAL TITLE: A Phase 1 open-label, active-controlled, randomized dose-finding study to evaluate safety, tolerability, and immunogenicity of intradermal and subcutaneous application of the plasmid DNA SARS-CoV-2 Omicron BA.2 vaccine Alveavax-v1.2 in primary Ad26.COV2.S vaccinated healthy individuals.

SPONSOR: Telis Bioscience Inc

19 Blackstone Street

Cambridge, Massachusetts USA 02139

PRINCIPAL INVESTIGATOR: ____

ADDRESS: ____

TELEPHONE #: ____

INTRODUCTION

You are invited to take part in a research study for a new vaccine. This document is needed to help you to decide if you would like to participate. You should fully understand what is involved before you agree to take part in this study. If you have any questions that are not fully explained in this document, do not hesitate to ask the study doctor. You should not agree to take part unless you are completely happy about all the procedures involved and possible risks. In the best interests of your health, it is strongly recommended that you discuss with or inform your personal doctor (general practitioner) of your possible participation in this study. The study doctor will also be notifying your personal doctor in this regard, unless you disagree that notification takes place.

THE PURPOSE OF THIS TRIAL

You are being invited to participate in the research of the new vaccine, Alveavax-v1.2 (“study vaccine”). Alveavax-v1.2 is an “investigational” vaccine meaning it has not yet been approved for use by the South African Health Products Regulatory Authority (SAHPRA). Alveavax-v1.2 is a COVID-19 vaccine that has DNA that carries the gene for SARS-CoV-2 spike protein for the Omicron BA.2 variant. You cannot get COVID from the Alveavax-v1.2 vaccine.
The trial will investigate different doses (amounts) of Alveavax-v1.2 and will compare it with the immune response of Ad26.COV2.S, a vaccine already approved by SAHPRA and Sponsored by Janssen (Johnson & Johnson).

The main purposes of this study are:

- See if Alveavax-v1.2 is safe for humans - it has not been administered to humans yet.
- Learn whether, and with which dose, Alveavax-v1.2 results in an adequate immune response.

An immune response is how your body recognises and defends itself against bacteria, viruses (such as COVID-19), and substances that appear foreign and harmful. Your immune system protects your body.

To participate, you must be a healthy adult between 18 to 65 years of age and have received Janssen Ad26.COV2.S vaccine at least 60 days prior to enrolling in this study and be eligible to receive a booster vaccine.

You will be randomly assigned (like rolling a die or flipping a coin) to receive either one (1) dose of Ad26.COV2.S (Janssen) vaccine or Alveavax-v1.2. If you are assigned to the Alveavax-v1.2 vaccine, you will receive either one (1) or four (4) injections of various doses (1/4 dose, 1 full dose, or 4 doses). The chances for receiving quarter, single and four doses of Alveavax-v1.2 are 16%, 30%, 24% (16% intradermal, 8% subcutaneous) respectively and the chance of receiving Janssen vaccine is 30%.
The injections are given in the skin of your arm (intradermal), under the skin of your arm (subcutaneous), or in your muscle (intramuscular) on your upper arm/shoulder area. For participants who cannot get an injection in your arm, your study doctor might decide to inject a different site such as your thigh or back. After any injection, you will be closely monitored for any reactions for at least 30 minutes and up to 4 hours at the study site. Alveavax-v1.2 is being injected into the skin of your arm because, as compared to muscles, skin has a higher concentration of cells that initiate the immune response (by identifying the vaccine). Although the intradermal injection might be more painful or have more local reactions, it is expected to generate a more robust and/or superior protective immunity. A more potent vaccine helps us give smaller effective doses making the vaccine more affordable and available to more people.

HOW IS THE STUDY VACCINE GIVEN?

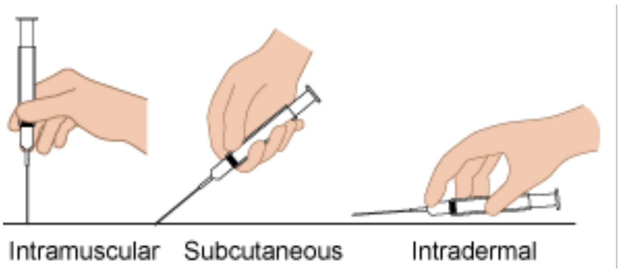

You will receive either one or four injections. Injection(s) will be made using a needle. Depending on where the needle is inserted, the injections can be either of three kinds. Intradermal is given in your skin, subcutaneous is given below your skin, and intramuscular is given deep in your muscle. Whenever possible, the vaccine will be injected into the arm you prefer.

Quarter and single doses are given through one injection intradermally. Participants receiving four doses of Alveavax-v1.2 will either receive one injection subcutaneously or four injections intradermally.

You will know which vaccine you received. If you receive Alveavax-v1.2, we will tell you what dose you have received. We will evaluate which dose has the best results and how it compares to the standard of care (Janssen).

Any vaccination can cause an allergic reaction. Allergic reactions can be immediate (begin within minutes) or delayed (appear several hours to days after). Allergic reactions can be mild or life-threatening. Severe allergic reactions are rare and have occurred in about 5 per one million vaccinations in the US.

The initial symptoms of allergic reactions include:

- A bad rash all over your body
- Itching all over your body
- Sweating
- Swelling of your face, tongue or throat
- Watering of eyes, running nose
- Sudden hoarseness of voice or wheezing
- Difficulty breathing
- A fast heartbeat
- A quick change in blood pressure (which can cause dizziness or light-headedness)

Please inform the study team if you have ever had a bad reaction to an injection or vaccine.
After receiving the injection, you will be monitored by the study doctor for symptoms of severe allergy. They can administer medications to treat severe allergic responses at the clinic. If you believe you are experiencing a severe allergic response after leaving the study site, call the emergency phone number and seek medical help immediately.

All vaccines have known or unforeseeable side effects. There is a small chance that you may have an adverse event to the vaccine. In addition, there is a slight possibility that you may have an unfavourable reaction to the vaccine, or it may make you sicker than if you were to contract COVID-19.

WHAT ARE THE SIDE EFFECTS AND RISKS ASSOCIATED WITH ALVEAVAX-V1.2?
Alveavax-v1.2 has not yet been tested in humans. However, we do not expect any potential discomfort, side effects, or risks to significantly differ from other well studied COVID-19 vaccines. Some are concerned that DNA from DNA vaccines could enter human genomes. But over 20 years of experience testing DNA vaccines in humans suggests that this is not the case.

There may be other possible risks or side effects of the Alveavax-v1.2 vaccine which are not fully known. You will be monitored for risks and side effects throughout your participation in the study. Therefore, it is very important that if you think you are having side effects or experiencing a change in your medical condition, you should contact and report them to your study doctor, regardless of whether or not you think it has to do with the study vaccine.

WHAT ARE SIDE EFFECTS OBSERVED IN OTHER COVID-19 VACCINES?
To date, most side effects reported by subjects have been mild to moderate in severity. In rare cases, severe side effects have been observed in participants.

The rare side effects have been reported in approximately only 3-40 cases per million COVID 19 doses administered. These include:

- <4 cases per million COVID 19 doses administered
    - Injury to the heart (myocarditis and pericarditis)
    - Skin disorders (cutaneous vasculitis)
    - Formation of blood clots leading to stroke, blood clots in the lung, bleeding disorder
    - Nervous system disorders like inflammation/injury to the brain (ADEM, aseptic meningitis, encephalitis), paralysis of muscles of the face (Facial nerve or Bell's palsy), seizures, sudden paralysis (Guillain-Barre syndrome), spinal cord inflammation, irreversible hearing loss
    - Sudden injury to the lungs (ARDS)

- Disease of lungs due to the vaccine (Vaccine Associated Enhanced Disease)
- Inflammation of more than one organ system (Multisystem Inflammatory Syndrome in Children and Adults [MIS-C/A])
- Vasculitis: Single organ cutaneous vasculitis
- Muscle breakdown (Rhabdomyolysis)
- Inflammation of thyroid (Subacute thyroiditis)
- Acute pancreatitis

- ~40 cases per million COVID 19 doses administered
  - Sudden paralysis (Guillain-Barre syndrome)

THE DURATION OF THIS TRIAL

If you decide to take part in this trial, you will be one of approximately 130 participants. Your participation will last for up to one (1) year and will include 7 study visits with your study doctor at the study doctor facilities and 1 study visit conducted by telephone.

After you consent to participate (sign this document), you will be screened to make sure you qualify to receive the vaccine injection.

YOUR RESPONSIBILITIES

For all study participants, the following responsibilities apply

1. Attend all study visits as instructed by the study staff.
2. Report your health status and medical problems to the study personnel.
3. Report any change or new medications, including over-the-counter drugs (for example, to treat side effects after the injection), you've taken to the study personnel.
4. Report all side effects or any medical problem you develop to the study personnel.
5. Complete the paper diary card daily for all/any side effects you experience for the first 7 days.
6. Inform the study doctor or staff, if you decide that you no longer want to be in the study.
7. Do not take part in any other medical research studies.
8. Do not receive COVID-19 vaccines other than the one provided through this study within the first 90 days.
9. Do not donate bone marrow, blood, and blood products from the time of the study vaccine administration until 3 months after receiving the study vaccine.

At each visit, you will undergo the following examinations and test:

Visit/Day	Procedures	Risks
Screening Visit<br>(Day -28 to Day -1)<br><br>Visit may take up to 4 hours	**Medical history** (includes asking questions about your prior medical status, contact information)	No foreseeable risks.
	**Physical examination:** A study doctor will perform a thorough examination of your organ systems, looking for any abnormal signs	

	**Vital signs:** Your blood pressure, heart rate, and temperature will be measured	
	**Blood draw for:**<br>Haematology, chemistry<br>(7ml/1.5 teaspoons)<br>Serology (HIV, HbsAG, and HCV)<br>(5ml/1 teaspoon)	See comment on blood sampling below this table.
	**Nasopharyngeal swab:** A swab will be inserted into your nose to collect a sample of your nasal secretions or cells from the nasopharynx (the uppermost part of your nose and throat). The nasal swab is a narrow stick made of a short plastic rod that is covered at one tip with adsorbing material such as cotton, polyester, or flocked nylon.	You may experience slight discomfort or tickling in the nose with this procedure. It may cause a nosebleed
	**Test for infections:** Your blood will be used to check for the presence of diseases, Hepatitis B, Hepatitis C, and HIV.	See comment on blood sampling below this table.<br><br>Positive tests will be reported to health authorities where applicable
Visit 1 Check-in<br>(Day 1)<br><br>Visit may take 3 to 4 hours	**Physical examination:** A study doctor will perform a thorough examination of your organ systems looking for any abnormal signs	No foreseeable risks
	**Vital signs:** Your blood pressure, heart rate, and temperature will be measured	
	**Blood draw for:**<br>Haematology and chemistry*<br>(7ml/1.5 teaspoons)<br>Humoral immunology<br>(5 ml/1 teaspoon)<br>Cellular immunology<br>(30 ml/6 teaspoons)	See comment on blood sampling below this table.
	**Nasopharyngeal swab:** same procedure as during screening.	You may experience slight discomfort or tickling in the nose with this procedure. It may cause a nosebleed
	**Urine pregnancy test:** If you are a female who could get pregnant, we will collect a urine sample from you to check for pregnancy.	No foreseeable risks.
Visit 1 Injection<br>(Day 1)	**Vital signs:** Your blood pressure, heart rate, and temperature will be measured	No foreseeable risks.

Visit may take from 1-6 hours	**Vaccine administration and monitoring:** You will be asked to remain on the study site for at least 30 minutes. The first five participants for each dose level will be asked to be observed for 4 hours after the injection. We will monitor vitals (includes blood pressure, pulse rate, and temperature) either once after 30 minutes or sequentially at 1, 2, 3, and 4 hours (±30 minutes) after the injection.	The main risks of the vaccine are local reactions (pain, redness, swelling at the injection site) and systemic reactions (fever, chills, tiredness, headache, sore throat, vomiting, diarrhoea, muscle pain, or joint pain).<br><br>The injection can cause pain due to the inserted needle and the injection itself. Depending on the volume injected the pain can be more severe. Injections can cause swelling, bruising, or redness. Risks also include faintness or loss of consciousness.
	**Diary instructions** (to be completed for the first 7 days): You will maintain a side effects diary according to the specific instructions provided. You will log oral temperature, measured dimensions of redness and swelling at the injection site, pain at the injection site, and related symptoms	No foreseeable risks.
Visit 2 Telephone Call (Day 3)<br><br>Telephone call may take 30 minutes	**Questions:** about how you are feeling and any side effects you may have.	No foreseeable risks.
	**Reminder of diary completion**	
Visit 3<br>(Day 7)<br><br>One week after injection<br><br>Visit may take 2 hours	**Physical examination:** A study doctor will perform a thorough examination of your organ systems looking for any abnormal signs	No foreseeable risks.
	**Vital signs:** Your blood pressure, heart rate, and temperature will be measured	
	**Blood draw for:**<br>Haematology, chemistry<br>(7ml/1.5 teaspoons)<br>Serology (HIV, HbsAG, and HCV)<br>(5ml/1 teaspoon)	See comment on blood sampling below this table.
	**Nasopharyngeal swab:** same procedure as during screening.	You may experience slight discomfort or tickling in the nose with this procedure. It may cause a nosebleed
	**Review and collection of diary card**	No foreseeable risks.

Visit 4, 5, 6 and 7 (Day 14, 28, 84 and 168 after your vaccination)<br><br>Visit may take 1 hour	**Questions:** Occurrence of pregnancy, COVID-19 infections and vaccinations	No foreseeable risks.
	**Blood draw for:**<br>Each visit: Humoral immunology (5ml/1 teaspoon)<br>Visit 5 and 7: Cellular immunology (30ml/6 teaspoons)	See comment on blood sampling below this table.
	Visit 4 & 5 only: **Nasopharyngeal swab:** same procedure as during screening.	You may experience slight discomfort or tickling in the nose with this procedure. It may cause a nosebleed
Visit 8<br>(Day 365)<br><br>One year after injection<br><br>Visit may take 1 hour	**Questions:** Occurrence of pregnancy, COVID-19 infections and vaccinations.	No foreseeable risks.
	**Physical examination:** A study doctor will perform a thorough examination of your organ systems looking for any abnormal signs	
	**Vital signs:** Your blood pressure, heart rate, and temperature will be measured	
	**Blood draw for:**<br>Humoral immunology (5ml/1 teaspoon)	See comment on blood sampling below this table.
	**Nasopharyngeal swab:** same procedure as during screening.	You may experience slight discomfort or tickling in the nose with this procedure. It may cause a nosebleed

Comment on risk by blood sampling: The study doctor or staff will draw blood from a vein in your arm. Blood collection may cause pain where the needle is inserted in the vein, and there is a small risk of bleeding, bruising, or irritation at the place of needle insertion. Rarely do some people experience dizziness or fainting when their blood is drawn. In rare cases, an infection may happen. Blood draw injection-site pain, swelling, bruising, or redness. Risks also include faintness or loss of consciousness.

As with any study, there may be unknown or unforeseeable risks to you. Adverse event assessment will happen at all study visits.

You do not have to take part in this study if you do not want to. Joining this study is not a part of your regular health care. If you take part, you can leave the study at any time without giving a reason. You will not lose access to medical care or other benefits that you are otherwise entitled to in either instance. In the best interests of your health, it is strongly recommended that you discuss with or inform your personal doctor (general practitioner) of your possible participation in this study. The study doctor will also be notifying your personal doctor in this regard, unless you disagree that notification takes place. If you choose not to participate, there will be no consequences. If you take part in this research study, you will be given a copy of this signed and dated document

ETHICS APPROVAL OF TRIAL

The Protocol of this clinical trial was submitted for approval to the South African Medical Association Research Ethics Committee (SAMAREC), a research ethics committee registered with the National Health Research Ethics Council. Written approval has been granted by SAMAREC for the conduct of the trial. The study has been structured in accordance with the Guidelines on Clinical Trials and Ethics in Health Research, published by the Department of Health and the Declaration of Helsinki (last updated October 2013), adopted by the World Medical Association (WMA), which deals with the recommendations guiding doctors in biomedical research involving human participants. Copies of these documents may be obtained from the study doctor should you wish to review them.

WHAT ARE THE BENEFITS OF PARTICIPATING IN THIS STUDY?

It is possible that you will not benefit from participating in this study, as we do not know if the vaccine will prevent COVID-19 disease. However, the vaccine you are given during this study may build up sufficient antibody levels for you to receive direct protection from severe infections with the Omicron strain or other strains of the SARS-CoV-2 virus. Your participation in this study may benefit the community and scientists and doctors by providing increased knowledge and information about the safety of this vaccine and its role in preventing infection with the SARS-CoV-2 virus.

You will benefit from boosted immunity if you receive the Janssen vaccine. If you receive Alveavax-v1.2, you may benefit if it proves to be effective. We will inform you 3 months after your injection if Alveavax-v1.2 does not prove to elicit the expected immune response so that you can get an approved COVID-19 booster vaccine. If Alveavax-v1.2 is not effective, postponing the receipt of the approved COVID-19 booster vaccine may place you at risk of acquiring COVID-19. All samples and information collected will be de-identified and securely stored for further analysis. There is no cost associated with participation in this study. You will receive financial compensation for the time and inconveniences that may arise from participation.

YOUR RIGHTS AS A PARTICIPANT IN THIS TRIAL

Your participation in this trial is entirely voluntary and you can refuse to participate or you can stop at any time without stating any reasons whatsoever. Your refusal to participate in or your withdrawal from this clinical trial will not affect your access to other medical care. The study doctor, however, retains the right to withdraw you from the study if it is considered to be in your best interest, in which event reasons will be provided for withdrawing you from the study. If it is detected that you did not give an accurate history or did not follow the guidelines of the trial and the prescriptions of the trial facility, you may be withdrawn from the trial at any time.

POPI ACT - DATA PROTECTION

POPIA (POPI Act) stands for the Protection of Personal Information Act (2013). The act was introduced to promote the protection of personal information (e.g. race, gender, address, telephone number – to name a

few) collected and processed by public and private bodies, amongst other reasons. The Sponsor is required to follow POPIA (POPI Act of 2013), for the processing of data collected for this research study.

CONSENT TO USE AND SHARE PERSONAL DATA

By signing this consent document, you consent to the use and sharing of your personal data for the purposes of this clinical trial. You are not obliged to give this permission. However, if you do not consent, you will not be able to participate in the clinical trial.

WILL YOUR CONSENT EVER EXPIRE?

This permission has no expiry date.

CAN YOU WITHDRAW YOUR CONSENT?

You have the right to withdraw your consent at any time by informing a member of the study team at telephone number and address at the top of this form.

WHAT HAPPENS IF YOU LEAVE THE CLINICAL TRIAL PREMATURELY?

If for any reason you terminate your participation in the clinical trial, site staff will inform the Sponsor that you are doing so. The site staff will ask you to return for a closing visit and if you agree, the site staff will also send the Sponsor details of that visit. Any information collected about you prior to your early withdrawal may be used and shared in accordance with this participant information and informed consent document. However, you may request that the data collected may be destroyed and / or records of your personal information must be deleted (in terms of Section 24 (1) of POPIA)

CAN THE STUDY STAFF REMOVE ME FROM THE STUDY?

The study staff and the Sponsor have the right to remove you from the study at any time without your consent. This may occur if:

- It is in your best medical interest to do so,
- You do not follow the study staff's instructions or follow the study requirements,
- The study is cancelled by the regulatory agency or the Sponsor,
- You received a SARS-CoV-2 vaccine < 3 months from Day 1,
- You received any live-virus vaccine (both licensed and investigational) within 4 weeks prior to and 4 weeks after Day 1, or
- You received any inactivated vaccine (both licensed and investigational), including influenza vaccine, within 2 weeks prior to or 2 weeks after Day 1.

The study staff will communicate why you were removed from the trial, as well as additional treatment, or research alternatives, and how they plan to monitor you for side effects.

CAN THE RESEARCH STUDY BE STOPPED BEFORE COMPLETION?

This research project may be stopped unexpectedly for a variety of reasons. These may include reasons such as:

- Unacceptable side effects or unacceptable risk to the participants enrolled in the clinical study;
- The treatment being shown not to be effective or need further testing;

Decisions made in the commercial interests of the Sponsor or by local regulatory/health authorities

In the interest of your safety, the investigator can inform your personal general practitioner as indicated by you that you are participating in this clinical trial.

The Sponsor and / or the clinical trial site will retain your personal data for 15 years after the investigation has ended (After this 15 year period the data will be destroyed). During this entire period, you may always:

- Ask for additional information about the processing of your personal data.
- Request access to the personal data held about you if this does not impede the scientific integrity of the clinical trial. To guarantee the scientific integrity of the clinical trial, you may only have access to certain personal data when the clinical trial has ended.
- Ask for corrections if the personal data is incorrect or incomplete
- Ask to transfer clinical trial-related personal data relating to you in a common format to yourself or someone else.
- If you feel any of your rights related to the collection and processing of your data have been violated, you should contact a member of the study team. If your concerns cannot be resolved to your satisfaction you can lodge a complaint in writing with the Information Regulator (South Africa), by writing to:

The Chief Executive Officer Information Regulator (South Africa)
P.O Box 31533
Braamfontein, Johannesburg, 2017
Tel: +27 (0) 10 023 5200
Email: complaints.IR@justice.gov.za
General enquiries Email: inforeg@justice.gov.za

ORGANISATIONAL AND TECHNICAL SECURITY MEASURES

The Sponsor has taken appropriate security measures to prevent accidental loss of your personal data, unauthorised use or access, changes or disclosure. For example, your personal data will be de-identified and anonymised (data will be processed in a way that cannot be tracked directly back to you) before it is stored, analysed or transferred. The Sponsor has also established procedures on how to deal with a suspected breach of data protection and will notify you and all designated supervisors of a suspected breach if it is required by law to do so.

TRANSFER OF YOUR DATA OUTSIDE SOUTH AFRICA

It is important to emphasise that some of the authorised users of your data may be located in countries that do not have the same standards as South Africa when it comes to the legal protection of personal data. Although the Sponsor, as the party responsible for the processing of personal data, makes every effort to respect the provisions of South African legislation on the protection of privacy, a transfer of personal data to

a country outside South Africa may pose a security risk. In addition, there is a risk that you may not be able to exercise certain rights or that it may be more difficult to exercise such rights against these recipients. To the extent possible, international recipients of your personal data will sign special contracts to ensure the security and protection of your rights. If the security and protection of your rights cannot be guaranteed if personal data is transferred to a country outside South Africa, your explicit consent for such transfers will be requested below. In all cases, all parties involved in the investigation are obliged to respect the confidentiality of your personal data.

WHERE WILL MY PERSONAL DATA BE PROCESSED AND WHO WILL HAVE ACCESS TO MY PERSONAL DATA?

Your study doctor and study staff will be responsible for collecting personal data, as required, for you to take part in the study. Along with medical data, including data from laboratory samples, other data collected may include your sex, age or date of birth, ethnicity, body weight and height. Your personal data related to your participation in the study will be replaced by a code so that you cannot be identified directly. Only your study doctor and study staff will be able to identify you from the code. The Sponsor and other companies working with the Sponsor on the study (the Sponsor representatives) will not be able to identify you directly.

The Sponsor and their representatives will be responsible for processing the information which will be stored under the code allocated to you and are responsible for ensuring your data remains confidential as required by the law in your country. Data collected about you for the purposes of this study will be transferred to a central location.

Some authorised Sponsor representatives (including the contract research organisation working with the Sponsor, laboratories testing your samples, monitors, auditors), National Health Authorities, Regulatory Authorities and Ethics Committees will have limited but direct access to your personal data (medical records and genetic data) held by the study site when required to check study procedures have been performed and data has been captured correctly. The information will remain confidential as required by the law in your country and remain the responsibility of the study doctor.

This access may include viewing your medical records remotely, from a location outside of the study site.

Once the study has been completed results of the study may be published. At no point will any personal information that can identify you be included in the results.

ALTERNATIVE TREATMENTS:

You may decide not to take part in this research study without any penalty. Instead of being in this research study, your choices may include:

- Vaccination with currently available South African Health Products Authority (SAHPRA)-authorised and / or approved COVID-19 vaccines.
- Enrolment in other COVID-19 vaccine studies.

TRIAL PROCEDURES MAY RESULT IN DISCOMFORT OR INCONVENIENCE

Venipunctures (i.e. drawing blood) are normally done as part of routine medical care and present a slight risk of discomfort. Drawing blood may result in a bruise at the puncture site, or less commonly fainting, swelling of the vein, infection and bleeding from the site. Procedures are performed under hygienic conditions by

experienced personnel. A total of approximately 150ml (~10 tablespoons) of blood will be taken over the course of this study.

The injection of the vaccine itself can cause pain due to the inserted needle and the injection itself. Depending on the volume injected the pain can be more severe. Injections can cause swelling, bruising, or redness. Risks also include faintness or loss of consciousness.

PREGNANCY / BIRTH CONTROL

Safety of the study medication in pregnancy and lactation and the effects during fertilisation of the egg cell has not been established. There might be unknown risks to the unborn child if a female participant is pregnant or becomes pregnant during the study or if a male participant fathers a child whilst on study medication. If you are a sexually active female who is not surgically sterile or less than two years post-menopausal, a urine pregnancy test will be done before you begin the study. It is mandatory to test negative on a pregnancy test before receiving the study vaccine. Furthermore, you must consent to use an authorised form of birth control prior to receiving the study vaccine and the following 84 days. Recommended methods of birth control include:

- The consistent use of an approved hormonal birth control (pill/patches, rings),
- An intrauterine device (IUD),
- Double barrier methods (Diaphragm with spermicidal gel or condoms with contraceptive foam),
- Sexual abstinence (no sexual intercourse until at least 84 days following the last study vaccination).

If you are a woman of child-bearing potential:

- You must not participate in this study if you are pregnant, or plan to become pregnant during the research study period, or are breastfeeding.
- You must use acceptable methods of birth control during this study (for example, a condom or a diaphragm plus spermicide; hormonal contraceptives that are injected, implanted or taken orally, or an intrauterine device.
- A pregnancy test will be done to confirm that you are not pregnant before your participation in this study.
- By signing this document, you confirm to the best of your knowledge that you are not pregnant now, breastfeeding and you do not intend to become pregnant during this study.

If at any time during this study you think you might be pregnant, or later learn during the study that you are pregnant, you must contact the study doctor immediately for further instructions regarding your participation in this study and follow-up.

Hormonal birth control, implants, and injections are only considered effective if used properly and started at least one month before you begin the study and at least 84 days after the study vaccination. You should ask your study doctor if you should continue birth control for longer than 84 days after the study vaccination. If you are unsure whether the method of birth control you use is acceptable to use while participating in this study, you should ask your study doctor before you begin the study.

No individual birth control is 100% effective. If you become pregnant, at any time during the one year that you participate in this study, it is important that you tell your study doctor immediately. A referral may be made to an obstetrician or gynaecologist for follow-up. Should you become pregnant, regardless of the outcome, the study doctor will collect information on your pregnancy, its outcome, and the health of the

child after birth even if you are withdrawn from the study and will share this information with the Sponsor. Your written consent will be obtained separately in the case that this happens.

MALE PARTICIPANTS

Since it is not known if Alveavax-v1.2 poses potential risks to a developing foetus, it is recommended that you use a condom and not impregnate a woman or donate sperm for at least an additional 84 days after you receive the study vaccination. This is because the study vaccine may be present in the sperm and/or seminal fluid even after study vaccination. You are encouraged to tell your female partner(s) and/or their doctor(s) that you are participating in a clinical study.

If your partner becomes pregnant during the study, it is important that the study team gathers information about the course and outcome of your partner's pregnancy so that any complications, including foetal abnormalities can be immediately identified. If your partner becomes pregnant during the study, you should tell the study doctor immediately. Your partner will be asked for permission to allow the study doctor to follow up and collect information about their pregnancy and the health of the baby. It is entirely voluntary. Your partner does not have to provide any information.

COSTS AND FINANCIAL ARRANGEMENTS

Neither you nor your medical scheme will be expected to pay for any study medication, study-related visits or trial procedures.

You will not be paid for being in this research study. You will not gain direct benefit from the study other than the potential benefit of the vaccine; however, the latter is not known and cannot be guaranteed in early phase research such as this. The Sponsor has arranged to offer you a fair reimbursement for daily loss of income for each study visit, any sundry items required, transportation to and from the research facility to participate in this study. You will be reimbursed following each completed visit. You should discuss this with the study doctor before commencing with the study.

INSURANCE AND COMPENSATION

The Sponsor has obtained insurance for you and the study doctor in the event of trial-related injuries. The Sponsor assumes no obligation to pay for the medical treatment of other injuries or illnesses not related to the studies. Further detailed information on the payment of medical treatment and compensation due to injury can be obtained from the study doctor should you wish to review it. Any compensation will be paid in accordance with the Association of the British Pharmaceutical Industry (ABPI) Guidelines on Compensation, which guidelines adequately cover the compensation aspects relating to clinical trials. A copy of these Guidelines is available from the study doctor on request.

Please note that if you have a life insurance policy, you should ask whether your insurance company requires notification of your intention to participate in a clinical trial. Our information to date is that it should not affect any life insurance policy taken out. Nevertheless, you are strongly advised to clarify this with the insurance company concerned.

Besides the above-mentioned compensation and medical care, no other compensation will be offered in case of an injury caused by this study. By signing this document, you do not waive any of your legal rights

should a research-related injury occur. You do not release the Sponsor, study doctor, study staff, or study site from liability for mistakes.

You must notify the study doctor immediately of any research or other related complications, side effects and/or injuries resulting from the trial, and the nature of the expenses to be covered.

By signing this document, you do not waive any of your legal rights should a research-related injury occur.

SOURCE OF ADDITIONAL INFORMATION

For the duration of the trial, you will be under the care of the study doctor, Dr__________________. If at any time between your visits you feel that any of your symptoms are causing you any problems, or you have any questions during the trial, please do not hesitate to contact him/her. The telephone number through which you can reach him/her or another authorised person is …………………… and/or………………………………

If you have questions about this trial, you should first discuss them with your doctor or the South African Medical Association Research Ethics Committee (SAMAREC):

CONTACT DETAILS OF SAMAREC

Address: Block F, Castle Walk office Park, Nossob Street, Erasmuskloof Ext 3 Pretoria
Tel: (012) 481 2082
Fax: (012) 481 2095
E-mail: samarec@samedical.org

After you have consulted your doctor or the Ethics Committee and if they have not provided you with answers to your satisfaction, you should write to the South African Health Products Regulatory Authority (SAHPRA) at:

The Chief Executive Officer
South African Health Products Regulatory Authority
Department of Health
Private Bag X828
PRETORIA
0001
E-mail: Boitumelo.Semete@sahpra.org.za
Tel: 012 842 7629/7626

CONFIDENTIALITY

All information obtained during the course of this trial is strictly confidential and will be maintained as such. Data that may be reported in scientific journals will not include any information that identifies you as a participant in this trial.

In connection with this trial, it might be important for domestic and foreign regulatory health authorities, such as the Department of Health, the National Health Research Ethics Council, the Food and Drug Administration of the United States of America, the South African Medical Association Research Ethics Committee (SAMAREC), the South African Health Products Regulatory Authority (SAHPRA), as well as authorised persons on behalf of the Sponsor, to be able to review your medical records pertaining to this

trial. Therefore, by signing this document, you authorise your study doctor to release your medical records in appropriate circumstances to the Sponsor, its employees or agents, domestic and foreign regulatory health authorities, the SAHPRA and the SAMAREC. You understand that these records will be utilised within reason by them only in connection with carrying out their obligations relating to this clinical trial.

Any information uncovered regarding your test results or state of health as a result of your participation in this trial will be held in strict confidence. You will be informed of any finding of importance to your health or continued participation in this trial but this information will not be disclosed to any other than those mentioned above without your written permission. The only exception to this rule will be in cases where a law exists compelling us to report incidences of communicable diseases. In this case, you will be informed of our intent to disclose such information to the authorised state agency.

The results of this research study may be presented at meetings or in publications. However, you will not be personally identified in any presentations or publications.

The information collected during this study may be added to research databases and used in the future by the Sponsor and its affiliated companies to study better measures of safety and effectiveness, study other therapies for subjects, develop a better understanding of disease included in the study or improve the efficiency, design and study methods of future clinical trials. Such information will not identify you by name.

INFORMED CONSENT

- I hereby confirm that I have been informed by the study doctor about the nature, conduct, benefits and risks of this clinical trial.
- I am aware that the results of the trial, including personal details regarding my sex, age, date of birth, initials and diagnosis will be anonymously processed into a trial report, but that some of my health information may be reasonably disclosed to the Sponsor and/or authorities under certain circumstances.
- I may, at any stage, without prejudice, withdraw my consent and end my participation in the trial.
- I have had sufficient opportunity to ask questions and (of my own free will) declare myself prepared to participate in the trial.
- I have read and understood the contents of the document.
- I understand that I shall receive a signed copy of this document.

**Participant:**		
Printed name	Signature	Date

I, Dr____ herewith confirm that the above participant has been informed fully about the nature, conduct and risks of the above trial.

**Study Doctor:**		
Printed name	Signature	Date

VERBAL PARTICIPANT INFORMED CONSENT
(This section is applicable when participants cannot read or write and should replace the previous Informed Consent section)

I, the undersigned study doctor, Dr__________________________________ , hereby confirm that:

- I have read and explained fully, to the participant, named _____________________________________ as well as the witness who signed below, with, the content of this document, indicating the nature and purpose of the trial in which I have asked the participant to participate.
- Verbal consent of the participant was obtained for the witness to be present during the consenting process.
- I have explained both the possible risks and benefits of the trial and the alternative treatments available for his/her illness.
- The participant has indicated that he/she understands the contents of the document and also that he/she will be free to withdraw from the trial at any time without giving any reason or jeopardising his/her subsequent treatment.
- I have informed the participant on the existence of relevant compensation arrangements in case of an injury attributable to the drug(s) used in the clinical trial, to which he/she agrees.
- The participant has had sufficient opportunity to ask questions.
- The participant has voluntarily agreed to participate in this trial.

**Participant:**		
Printed name	Signature	Date

**Study Doctor:**		
Printed name	Signature	Date

I, the witness who signed below, confirm that the study doctor has explained fully the content of this document to the participant.

**Witness:**		
Printed name	Signature	Date

(Witness' signature confirms that he/she has witnessed the relevant signatures at the time of signing. Witness name, signature and date must be completed by the witness at the same time that this document is signed and dated by the participant and the Study Doctor. A competent witness is a person 16 years or older and of sound mind and not involved with the trial.)
