# Supplementary material for: Safety and immunogenicity of DNA omicron booster Alveavax-v1.2 in Ad26.COV2.S-vaccinated adults
Source: iScience. 2025 Nov 10;28(12):113970. doi: 10.1016/j.isci.2025.113970 (PMC12704268; doi:10.1016/j.isci.2025.113970)
Supplement: Methods S6. Informed consent screening [file mmc9.pdf]

Methods S6: Informed Consent Screening

PRE-SCREENING INFORMATION AND INFORMED CONSENT DOCUMENT

Title: A Phase 1 open-label, active-controlled, randomized dose-finding study to evaluate safety, tolerability, and immunogenicity of intradermal and subcutaneous application of the plasmid DNA SARS-CoV-2 Omicron BA.2 vaccine Alveavax-v1.2 in primary Ad26.COV2.S vaccinated healthy individuals.

Sponsor: **Telis Bioscience Inc.**
19 Blackstone St, Cambridge, MA 02139

Principal Investigator: XXXXXXXXX Phone : XXXXXXXX

Please ask the study doctor to explain any words or information that you do not understand.

PURPOSE AND BACKGROUND

The purpose of this Pre-Screening Informed Consent Document is to inform you about collecting a blood sample for clinical laboratory testing. The tests carried out will check that your blood cells and blood chemistry are within the limits allowed for study Alvea-VAX-P00001. We will also use the sample to check Hepatitis B and C and HIV status. Testing for these viruses is important since they alter the bodies' immune system and can influence the effects of the drug studied.

The results of the blood tests will show whether or not you are eligible to take part in study Alvea-VAX-P00001.
You will have the opportunity to discuss your decision with your family and friends. You can also discuss it with your health care team (e.g., your personal /doctor). If you have any questions about the study, you can ask your study doctor for more explanation.
Allowing the collection of this blood sample is voluntary. If you decide not to allow collection of the sample, this will not impact the care that you would normally receive from your doctor.

By signing this Pre-Screening Consent document, you are agreeing to provide blood samples that can be used for routine blood and blood chemistry testing, and to check for HIV, Hepatitis B and C status.

Why Is This Testing Being Done?

You may be eligible for the Alvea-VAX-P00001 study. If the results of the tests show this is the case, you will then be asked to read, discuss, ask questions, and, if interested, sign an additional consent document explaining study Alvea-VAX-P00001 and its procedures in greater detail. Your study doctor will determine if you are eligible.

What will happen if I take part in this testing?

If you choose to take part in this testing, the study doctor or study staff will collect a blood sample from you of approximately 12ml (2 ½ teaspoons) to test for blood counts, blood chemistry and HIV, Hepatitis B and Hepatitis C status.

What are the possible risks or discomforts of this testing?

The main risks of blood tests are discomfort and bruising at the site where the needle goes in. These complications usually are minor and go away shortly after the tests are done.

Are there benefits to taking part in this testing?

No direct benefit to you can be promised as a result of your taking part in this testing.

Will I be paid if I take part in this testing?

You will not be paid for taking part in this testing.

Will it cost me anything to do this testing?

There are no costs to you or to your medical scheme to take part in this testing. The sponsor of study Alvea-VAX-P00001, Alvea LLC, will cover the cost of the testing.

What happens if I am injured because I took part in this testing?

This is a routine procedure; there are no research-related injuries anticipated from this testing. However, it is important that you tell your study doctor, Dr. XXXXXXXXXXXXXXX, if you feel that you have been injured as a result of taking part in this sample collection. You can tell the doctor in person or call him or her at Tel xxxxxxx.

The research study is covered by an insurance policy taken out by the study Sponsor in the event that you suffer a bodily injury as a result of taking part in the study.
The insurer will pay for all reasonable medical costs required to treat your bodily injury, in accordance with the SA Good Clinical Practice Guidelines (latest version), which are based on the Association of the British Pharmaceutical Industry Guidelines. You may request a copy of these guidelines from the study doctor.

The insurer will pay without you having to prove that the research was responsible for your bodily injury. The insurer will not pay for harm if, during the study you:

- Do not follow the study doctor's instructions
- Do not tell the study doctor that you have a bad side effect from the study medicine
- Suffer an injury arising from negligence on your part or do not take reasonable care of yourself and your study medicine.

If you are harmed and the insurer pays for the necessary medical costs, usually you will be asked to accept that insurance payment as full settlement of the claim for medical costs. However, accepting this offer of insurance cover does not mean you give up your right to make a separate claim for other losses based on negligence, in a South African court.

What are my rights if I take part in this testing?

If you eventually take part in the research study, all information related to sample management will be given to you in the additional informed consent document you will receive and sign. If you decide not to take part in this testing, you will not be able to take part in study Alvea-VAX-P00001. However, it will not affect your ability to enrol in another research study or to receive other treatment. You can discuss your other treatment options with your doctor. If you do not take part in the research study after this screening assessment is done, the samples collected during the screening assessment will be destroyed at the site. You may change your mind and revoke (take back) this authorisation at any time.

Will my medical information be kept private?

Your medical information will be kept as confidential as possible within the limits of the law.

How will my health information be used and disclosed?

If you sign this document, you give permission to Dr XXXXXX to use or disclose (share) your health information that identifies you only for the purposes of the testing described in this document.
The health information you are giving permission to be used and shared includes all health information about you relating to the analyses of your blood sample(s). This health information will only be used by and/or disclosed (shared) to the laboratory performing the testing.
Once coded, the result of the tests will be shared with Alvea LLC in order to confirm you are eligible to take part in study Alvea-VAX-P00001, and will become part of the study records. Under data protection law [Protection of Personal Information Act] your study site and the Sponsor shall be jointly responsible as 'controllers' for ensuring that your information is safeguarded. The Sponsor has appointed Clindev (PTY) Ltd (a wholly owned subsidiary of Micron Research Ltd) as its 'representative' in South Africa to fulfil its obligations under this law.

Who can answer my questions about the study?

If you have questions about this trial, you should first discuss them with your doctor or the Ethics Committee (contact details as provided on this document).
Contact your study doctor, Dr. xxxxxx, at tel xxxxxx. If you think you have been injured as a result of taking part in the study, contact Dr xxxxx, at tel xxxxxxxxxxx.

SAMAREC Research Ethics Committee
Address: Block F, Castle Walk office Park, Nossob Street, Erasmuskloof Ext 3 Pretoria
Tel: (012) 481 2082
Fax: (012) 481 2095
E-mail: samarec@samedical.org

After you have consulted your doctor or the Ethics Committee and if they have not provided you with answers to your satisfaction, you should write to the South African Health Products Regulatory Authority (SAHPRA) at:
The Chief Executive Officer
South African Health Products Regulatory Authority
Department of Health
Private Bag X828
PRETORIA
0001

E-mail: Boitumelo.Semete@sahpra.org.za
Tel: 012 842 7629/7626

Informed Consent

- I hereby confirm that I have been informed by the study doctor about the nature, conduct, benefits and risks of this clinical trial.
- I am aware that the results of the trial, including personal details regarding my sex, age, date of birth, initials and diagnosis will be anonymously processed into a trial report, but that some of my health information may be reasonably disclosed to the Sponsor and/or authorities under certain circumstances.
- I may, at any stage, without prejudice, withdraw my consent and end my participation in the trial.
- I have had sufficient opportunity to ask questions and (of my own free will) declare myself prepared to participate in the trial.
- I have read and understood the contents of the document.
- I understand that I shall receive a signed copy of this document.

Participant:		
Printed name	Signature	Date

I, Dr____ herewith confirm that the above participant has been informed fully about the nature, conduct and risks of the above trial.

Study Doctor:		
Printed name	Signature	Date

Verbal Participant Informed Consent

(This section is applicable when participants cannot read or write and should replace the previous Informed Consent section)

I, the undersigned study doctor, Dr_________________________________ , hereby confirm that:

- I have read and explained fully, to the participant, named _____________________________ as well as the witness who signed below, the content of this document, indicating the nature and purpose of the trial in which I have asked the participant to participate.
- Verbal consent of the participant was obtained for the witness to be present during the consenting process.
- I have explained both the possible risks and benefits of the trial and the alternative treatments available for his/her illness.
- The participant has indicated that he/she understands the contents of the document and also that he/she will be free to withdraw from the trial at any time without giving any reason or jeopardising his/her subsequent treatment.
- I have informed the participant on the existence of relevant compensation arrangements in case of an injury attributable to the drug(s) used in the clinical trial, to which he/she agrees.
- The participant has had sufficient opportunity to ask questions.
- The participant has voluntarily agreed to participate in this trial.

Participant:		
Printed name	Signature (if possible)	Date

Study Doctor:		

Printed name	Signature	Date

I, the witness who signed below, confirm that the study doctor has explained fully the content of this document to the participant.

Witness:		
Printed name	Signature	Date

(Witness' signature confirms that he/she has witnessed the relevant signatures at the time of signing. Witness name, signature and date must be completed by the witness at the same time that this document is signed and dated by the participant and the Study Doctor. A competent witness is a person 16 years or older and of sound mind and not involved with the trial.)
